# Maximizing the Potency of siRNA Lipid Nanoparticles for Hepatic Gene Silencing In Vivo**

**DOI:** 10.1002/anie.201203263

**Published:** 2012-07-10

**Authors:** Muthusamy Jayaraman, Steven M Ansell, Barbara L Mui, Ying K Tam, Jianxin Chen, Xinyao Du, David Butler, Laxman Eltepu, Shigeo Matsuda, Jayaprakash K Narayanannair, Kallanthottathil G Rajeev, Ismail M Hafez, Akin Akinc, Martin A Maier, Mark A Tracy, Pieter R Cullis, Thomas D Madden, Muthiah Manoharan, Michael J Hope

**Affiliations:** AlCana TechnologiesVancouver, BC (Canada); Alnylam PharmaceuticalsCambridge, MA (USA) E-mail: mjayaraman@alnylam.com; Department of Biochemistry and Molecular Biology, University of British ColumbiaVancouver, BC (Canada)

**Keywords:** drug delivery, gene silencing, ionizable amino lipids, liposomes, siRNA

RNA interference (RNAi) is a ubiquitous and highly effective biological mechanism to control gene expression. Over the last few years it has been established that synthetic double-stranded, small interfering RNA (siRNA) molecules can be applied as therapeutics that potently harness RNAi in humans and animal models, by demonstrating silencing of target mRNA transcripts in rodents and non-human primates (NHP).[1] Because of their high molecular weight and polyanionic nature, synthetic siRNAs fail to cross biological membranes by passive diffusion and therefore, generally require transmembrane drug delivery technologies to access the cytoplasm of target cells.[2, 3] Once in the cytoplasm, however, siRNAs readily load into the RNA-induced silencing complex (RISC) and direct sequence-specific cleavage of target mRNA. The resulting reduction in expression of protein, for example, can be profound and durable, lasting several weeks after administration of a single dose.[4] This new class of therapeutics, with its unique mechanism of action and ability to specifically inhibit previously “undruggable” disease-causing proteins, has prompted research into intracellular delivery technologies suitable for parenteral administration.

Lipid nanoparticles (LNPs) containing ionizable amino lipids that self-assemble into approximately 100 nm particles when mixed with polyanionic oligonucleotides, represent the most advanced delivery platforms for systemic administration of RNAi therapeutics. Substantial progress has been made in recent years to maximize efficacy and to achieve a favorable therapeutic index for human applications.[2, 4] The ionizable amino lipid plays a dual role in the drug delivery process. First, through electrostatic interaction with polyanionic nucleic acids, it promotes the self-assembly of formulation components into macromolecular nanoparticles encapsulating the siRNA. Second, following endocytosis of LNPs by target cells, it enables siRNA to escape the endosomal compartment and access the cell cytoplasm. In a recently published analysis of the structure–activity relationship of cationic lipid molecules we noted that permanently charged quaternary amine moieties in the hydrophilic head group region were consistently less effective in RNAi-mediated gene knockdown in vivo as compared to ionizable lipids.[5]

Herein, we report on a detailed study aimed at further investigating the role of the amine head group in the delivery of siRNA in vivo. Through systematic structural modifications to the hydrophilic head group we have modulated the apparent acid dissociation constant (p*K*_a_) of the ionizable amino lipids present in the LNPs and investigated the relationship between the p*K*_a_ value and activity, that is, the ability of the LNPs to deliver functionally active siRNAs into hepatocytes for eliciting gene silencing in vivo.

Amino lipid 2,2-dilinoleyl-4-dimethylaminoethyl-[1,3]-dioxolane (DLin-KC2-DMA, **1**), has been identified as a highly potent cationic lipid (Figure 1, Table 1)[5] when incorporated into LNPs encapsulating siRNA to target murine clotting factor VII (FVII) mRNA.[6] The hydrophobic dilinoleyl chain is known to be optimal for activity,[5, 7] hence this unsaturated alkyl chain configuration was maintained in all the lipids investigated herein and structural modifications were added to the head group region of the molecule. The formulations were prepared by a preformed vesicle method, as described in the Supporting Information (page 38), with the following lipid composition: amino lipid, distearoylphosphatidylcholine (DSPC), cholesterol and (*R*)-2,3-bis(octadecyloxy)propyl-1-(methoxy poly(ethylene glycol)2000)propylcarbamate (PEG-lipid) in the molar ratio 40/10/40/10, respectively, and a FVII siRNA/total lipid ratio of approximately 0.05 (*w*/*w*).[5] To ensure a narrow particle size distribution in the range of 70–90 nm and a low polydispersity index of 0.11±0.04 (*n*=56), the particles were extruded up to three times through 80 nm membranes prior to adding the siRNA.[5] Amino lipid p*K*_a_ values were determined for each LNP by measuring the fluorescence of 2-(*p*-toluidino)-6-napthalene sulfonic acid (TNS) during titration from pH 3 to 10 in increments of 0.5 pH units (Supporting Information, page 40).[8–10] The anionic TNS molecule fluoresces when associated with the surface of positively charged membranes, but is not fluorescent when free in solution, and this property has been used extensively to probe positive charge at membrane surfaces.[8–10]

**Figure 1:**
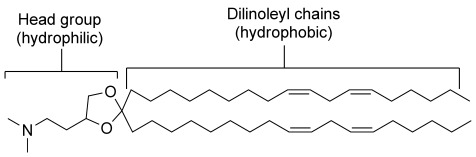
2,2-dilinoleyl-4-dimethylaminoethyl-[1,3]-dioxolane (DLin-KC2-DMA, **1**). No difference in activity between enantiomers (**4**, **5**) or the racemic mixture was observed (Table 1).

**Table 1:** Structures of the 56 amino lipids tested for gene silencing activity in vivo. ED_50_ is for FVII gene silencing in mice (mg siRNA kg^−1^) and the p*K*_a_ of the lipid was measured in situ. Lipids 1, 3, and 53 were reported earlier and used as references in this study.[5, 7]

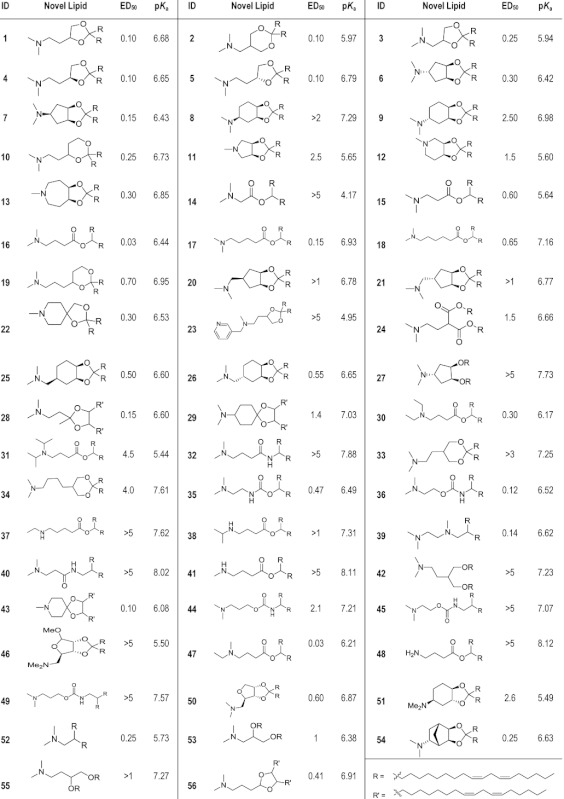

From the resulting fluorescence titration curve a pH value, at which the charge on the LNP is 50 % of maximum (p*K*_a_), can be calculated using curve-fit analysis (Figure 2). Importantly, this method enables a p*K*_a_ determination of amino lipids in situ, which accounts for potential influences of membrane structure and neighboring lipids on the dissociation properties of the amino group. Similar p*K*_a_ values were obtained for select formulations using zeta potential measurements over the same pH titration range, but TNS was found to be the more sensitive method (data not shown).

**Figure 2:**
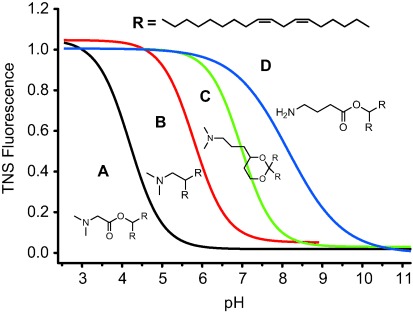
Determination of p*K*_a_ by in situ TNS fluorescence titration. The four amino lipids shown are examples illustrating the full p*K*_a_ range evaluated. Sigmoidal best-fit analyses were applied with p*K*_a_ defined as the pH at half-maximal fluorescence intensity. A) **14**, p*K*_a_=4.17; B) **52**, p*K*_a_=5.73; C) **19**, p*K*_a_=6.95; D) **48**, p*K*_a_=8.12 (see Supporting Information page 40 for details).

The hydrophilic head-group region of **1** was extensively modified, producing 53 novel amino lipids with p*K*_a_ values ranging from 4.17 to 8.12 (Table 1; see the Supporting Information for full experimental details on lipid synthesis). Lipids **1**, **3**, and **53** have been reported earlier and were used as references in this study.[5],[7] The structural modifications introduced were sufficient to affect the p*K*_a_ value without significantly altering the molecular dimensions of the hydrophilic region. This is important because having a small head group is a characteristic that enables amino lipids to adopt inverted, non-bilayer structures, which are hypothesized to be responsible for destabilizing the endosomal membrane, resulting in efficient release of siRNA into the cytoplasm of target cells.[5, 11]

A plot of p*K*_a_ value versus ED_50_ (median effective dose for FVII gene silencing in female C57BL/6 mice by i.v. administration) for all 56 amino lipids tested reveals a strong correlation between the in vivo activity and the acid dissociation constant of the amine, with a p*K*_a_ optimum seen between 6.2 and 6.5, on either side of which LNP potency rapidly decreases (Figure 3). On the other hand, significant potency differences were found for lipids with similar p*K*_a_ values. These results indicate that an optimal p*K*_a_ value is a necessary but not sufficient requirement for good in vivo activity and other structural features, such as the nature of the linker between the head group and the lipid tails, can also contribute substantially to the in vivo activity.

**Figure 3:**
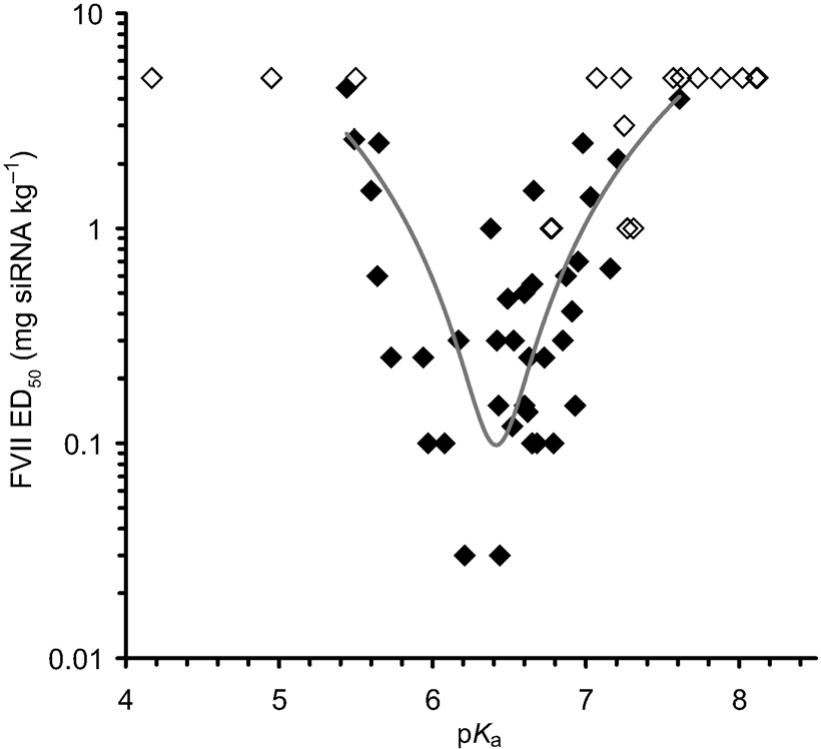
Plot of in vivo hepatic gene silencing activity vs. p*K*_a_ in mice. The 56 amino lipids were formulated in LNPs and subjected to an ED_50_ analysis (described in Supporting Information, page 39) and plotted against their p*K*_a_. For 15 lipids the ED_50_ dose was not achieved, these are indicated by the open diamonds representing the highest dose tested for that lipid. For the remaining lipids (closed diamonds), a polynomial best-fit curve highlights the most active compounds, which exhibit an optimal p*K*_a_ between 6.2 and 6.5. Each data point is derived from a four-dose response curve with groups of *n*=4 mice per dose.

To further investigate the p*K*_a_–activity relationship, a selected group of lipids were studied in more detail. These novel lipids were designed around the structure of dilinoleylmethyl-4-dimethylaminobutyrate (**16**, ED_50_=0.03 mg kg^−1^, p*K*_a_=6.44) also referred to as DLin-MC3-DMA (Figure 4), one of the most active cationic lipids from the group of 56 lipids. In the first group of lipids (**31**, **30**, **38**, and **37**), the distance between ester and amine is maintained at three methylene units and the substitution on the *N*-atom altered to create a p*K*_a_ range between 5.44 and 7.62.

**Figure 4:**
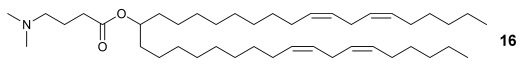
(6*Z*,9*Z*,28*Z*,31*Z*)-Heptatriaconta-6,9,28,31-tetraen-19-yl 4-(dimethylamino)butanoate (**16**, Table 1).

In the second group (**14**, **15**, **17**, and **18**), the dimethylamino moiety is maintained but the distance between the ester and the amine is varied from one to five methylene units to generate lipids with p*K*_a_ values ranging from 4.17 to 7.16. The plot of ED_50_ as a function of p*K*_a_ value shows that the highest potency is achieved at a p*K*_a_ of 6.44 for the siRNA LNP formulation containing lipid **16** (Figure 5, blue line). Activity substantially decreases as the p*K*_a_ value shifts to either the acidic or basic side, underlining the importance of the ionization behavior of LNPs in the functional delivery of siRNA to hepatocytes in mice.

**Figure 5:**
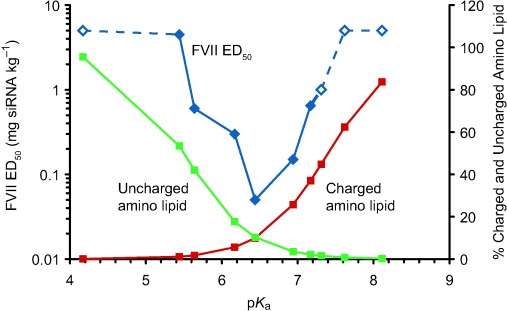
Plot of hepatic gene silencing activity in mice, p*K*_a_ and calculated protonated/unprotonated amino lipid (% mole fractions) for the set of novel lipids between pK_a_ 4 and 8.5. Each FVII ED_50_ data point (blue) is derived from a four-dose response (*n*=4 mice per dose level). Open diamonds represent lipids for which the ED_50_ was not achieved and are shown at the highest dose tested. The mole % of each amino lipid estimated to carry a positive charge in blood, assuming pH 7.4, is shown in red and the mole % amino lipid estimated to be uncharged in acidified endosomes at pH 5.5 is shown in green.

The surprisingly sharp p*K*_a_ value dependence of LNP activity provides more insight into the complex in vivo mechanism for this highly potent class of LNPs for siRNA delivery. In brief, it has been proposed that intravenous administration of LNPs with low to zero surface charge will minimize nonspecific binding of LNPs to tissues and proteins thus increasing the likelihood of particles reaching hepatocytes.[5] In addition, we recently demonstrated that uptake of ionizable LNPs by hepatocytes in vitro and in vivo is mediated by apolipoprotein E (ApoE),[12] which is also known to preferentially associate with neutral membrane surfaces.[13]

On the other hand, LNPs must acquire sufficient positive surface charge following endocytosis to promote an electrostatic interaction with the negative surface of the endosome lumen. The resulting close proximity of LNP and membrane allows lipid mixing to occur, leading to the formation of ion pairs between the exogenous amphipathic amine and endogenous anionic membrane lipids.[11, 14] The ion pairs prefer to adopt inverted, non-bilayer configurations that disrupt the integrity of the endosome membrane and the siRNA is subsequently released into the cytoplasm.[5] The ED_50_–p*K*_a_ profile may, therefore, represent a balance between two opposing delivery requirements, namely minimum charge at pH 7.4 to maximize hepatocyte uptake by ApoE-mediated endocytosis and maximum positive charge in the acidified lumen of endosomes to promote membrane disruption.

If potency differences of structurally similar lipids are predominantly a function of p*K*_a_ value, we hypothesized that the activity of LNPs containing two or more cationic lipids should reflect the average p*K*_a_ of the particle and not the average activity measured for the individual amino lipids. To test this, LNP formulations encapsulating FVII siRNA were prepared in which the 40 mole % amino lipid component consisted of different ratios of lipids **15**, **16**, and **17** thus producing LNPs with average surface p*K*_a_ values that increased incrementally from 5.64 (**15**) to 6.93 (**17**). The measured p*K*_a_ value for mixed lipid LNPs was shown to be the same (within experimental error) as that calculated from the mole ratio of the mixed amino lipids (Supporting Information, page 41). Each LNP was administered at the same dose (0.1 mg kg^−1^ siRNA) and the concentration of murine plasma FVII protein was determined 24 h later and expressed as a percentage of that measured in a saline-treated control (Figure 6).

**Figure 6:**
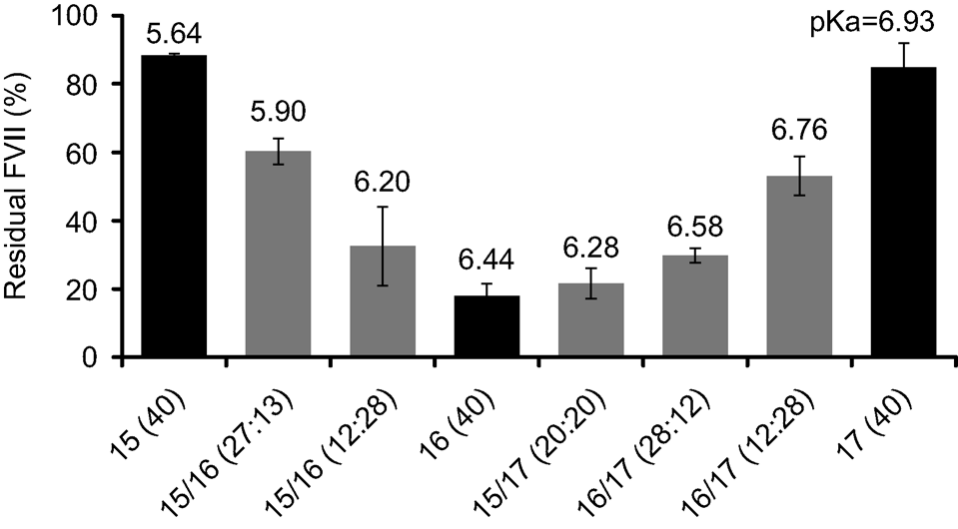
Lipid mixing supports p*K*_a_ value as the dominant factor in determining in vivo hepatic gene-silencing activity for a given set of amino lipids. Mixtures of amino lipids **15**, **16**, and **17** were made to incrementally shift the average surface p*K*_a_ value of the resulting LNP between 5.64 and 6.93. Numbers in parenthesis indicate the amount of each lipid in the mixture to make up the 40 mole % amino lipid component of the LNP. Bars represent group mean (*n*=4)±s.d.

As the average p*K*_a_ value increases from 5.64 (**15**) or decreases from 6.93 (**17**) by mixing with **16**, LNP activity increases. We did not identify a mixture with greater activity than **16** alone, but importantly, LNPs containing an equimolar mixture of **15** (ED_50_=0.6) and **17** (ED_50_=0.15), lipids that individually are 20- and 5-fold less potent than **16**, respectively, showed an average apparent p*K*_a_ value similar to **16** and equivalent activity. These data further confirm that for amino lipids of similar structure, in vivo hepatic gene silencing is driven by p*K*_a_ rather than specific head group structure or linker length (the distance between the ester and the amine). We have subsequently shown this to be true for mixtures of other cationic lipids with different head group structures (data not shown).

The pH-dependent surface charge for each LNP in the blood or endosomal compartment can be calculated from their p*K*_a_, using the Henderson–Hasselbach equation, assuming blood pH 7.4 and acidified endosome pH 5.5.[15] The amount of fully or partially charged LNPs carrying positive charge for each chosen lipid (**14**–**18**, **30**, **31**, **37**, and **38**) is expressed as a fraction of charged amino lipids plotted red in Figure 5 along with the fraction of amino lipids that would be uncharged at pH 5.5 in the endosome, plotted green. This demonstrates that the optimal p*K*_a_ value for in vivo hepatic gene silencing coincides with the p*K*_a_ giving rise to LNPs that exhibit minimum surface charge in the blood at pH 7.4 and maximum charge in acidified endosomes at pH 5.5. It should be noted, however, that the p*K*_a_-value trend found for this class of ionizable lipids with apoE-dependent hepatic delivery may not apply to other cationic and potentially ApoE-independent delivery systems.[6, 12]

To identify potent and safe siRNA delivery systems for clinical development, it is important that the potency in mice translates to non-human primates. For the following experiments LNPs containing the highly potent amino lipid **16** were used, in which the molar ratio of the four lipid components **16**, DSPC, cholesterol and PEG-lipid (50/10/38.5/1.5) had been further optimized to enhance in vivo activity. In the murine FVII model, an ED_50_ of 0.005 mg kg^−1^ siRNA was achieved in the optimized composition, which represented a sixfold improvement relative to the 40/10/40/10 molar ratio composition (Figure 7, inset). The same lipid composition was administered to cynomolgus monkeys but with an encapsulated siRNA targeting hepatic transthyretin (TTR), a gene of therapeutic interest in the treatment of familial amyloidotic polyneuropathy.[16] Animals received a 15 min intravenous infusion at siRNA doses of 0.03, 0.1, or 0.3 mg kg^−1^ and from the dose response curve an ED_50_ less than 0.03 mg kg^−1^ was obtained (Figure 7).

**Figure 7:**
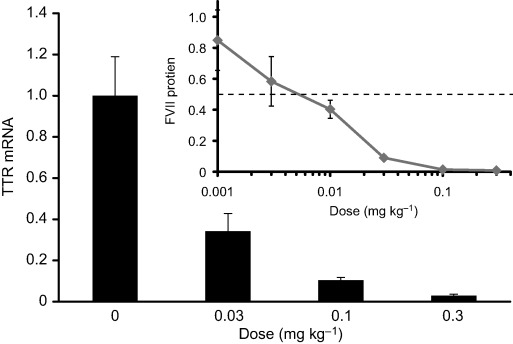
Efficacy of siRNA-LNP containing amino lipid **16** in mice and non-human primates. An optimized formulation composition containing 50 mole % of **16** was employed in this study (see text and Supporting Information page 41 for details). Inset: residual FVII activity in mouse serum relative to a saline control group with a median effective siRNA dose (ED_50_) of about 0.005 mg kg^−1^; data points represent group mean (*n*=5)±s.d. Cynomolgus monkeys were administered siRNA targeting the TTR gene at doses of 0.03, 0.1 and 0.3 mg kg^−1^ (bar graph). Data represent group mean (*n*=3)±s.d., expressed as TTR mRNA relative to GAPDH mRNA levels determined in liver samples, with the ED_50_ estimated to be <0.03 mg kg^−1^.

In conclusion, we have identified the lipid p*K*_a_ value as an important molecular characteristic of amino lipids, which determines their ability to mediate potent hepatic gene silencing in vivo. Combined with our current understanding of the optimal hydrocarbon chain length, lipid-chain unsaturation and linker chemistry for these molecules, the tight correlation between activity and ionization behavior, with a sharp pK_a_ optimum between 6.2–6.5, serves as a guide for the design of LNPs for efficient delivery of therapeutic siRNAs. In fact, a LNP formulation based on DLin-MC3-DMA (**16**), one of the most potent lipids identified in this work, is currently being utilized in a clinical trial for treating hypercholesterolemia; the target is PCSK9 and the data (not shown) indicate decreases in LDL-C after a single low dose.[17, 18]

## Supplementary material

Detailed facts of importance to specialist readers are published as ”Supporting Information”. Such documents are peer-reviewed, but not copy-edited or typeset. They are made available as submitted by the authors.
