# Supplementary material for: Maximizing the Potency of siRNA Lipid Nanoparticles for Hepatic Gene Silencing In Vivo
Source: Angew Chem Int Ed Engl. 2012 Jul 10;51(34):8529–33. doi: 10.1002/anie.201203263 (PMC3470698; doi:10.1002/anie.201203263)
Supplement: Supplementary file 1 [file anie0051-8529-SD1.pdf]

Angewandte Chemie
Eine Zeitschrift der Gesellschaft Deutscher Chemiker

Supporting Information

© Wiley-VCH 2012

69451 Weinheim, Germany

Maximizing the Potency of siRNA Lipid Nanoparticles for Hepatic Gene Silencing In Vivo**

Muthusamy Jayaraman, Steven M. Ansell, Barbara L. Mui, Ying K. Tam, Jianxin Chen, Xinyao Du, David Butler, Laxman Eltepu, Shigeo Matsuda, Jayaprakash K. Narayanannair, Kallanthottathil G. Rajeev, Ismail M. Hafez, Akin Akinc, Martin A. Maier, Mark A. Tracy, Pieter R. Cullis, Thomas D. Madden, Muthiah Manoharan, and Michael J. Hope**

anie_201203263_sm_miscellaneous_information.pdf

DOI: 10.1002/anie.201203263

Supporting Information

General Information

^{1}H and ^{13}C NMR spectra were measured on a JEOL JMTC-500 or JNM-FX 400 NMR instrument (500 MHz or 400 MHz for ^{1}H NMR, 125 MHz or 100 MHz for ^{13}C NMR, 200 MHz for ^{31}P NMR). Tetramethylsilane (TMS) served as the internal standard (0 ppm) for 1H NMR, and $CDCl_3$ served as the internal standard (77.0 ppm) for ^{13}C NMR. The following abbreviations were used to express the multiplicities: s = singlet; d = doublet; t = triplet; q = quartet; m = multiplet; br = broad. High performance liquid chromatography (HPLC) was performed on Shimadzu 10A instruments using a Daicel Chiralpac AS-H, AD-H, IA, Chiralcel OZ-H, or OJ-H 4.6 mm × 25 mm column. High-resolution mass spectra (HRMS) were performed on Bruker microTOF focus–KR. All reactions were monitored by thin-layer chromatography carried out on Sigma Aldrich precoated TLC plates (silica gel 60GF-254, 0.25 mm), visualization by using either UV (254 nm) or phosphomolybdic acid stain. The products were purified by flash column chromatography on silica gel or using an automated Teledyne ISCO combiflash Rf chromatography system. All simple chemicals were purchased and used as received.

METHODS

Synthesis of cationic lipids.

The lipids **1, 3** and **53** were synthesized as reported earlier[5,7] and the spectral data were matching with that of reported values. The lipids **4** and **5** were synthesized using a similar procedure to the synthesis of **2** by using the corresponding commercially available optically pure butane triols.

Synthesis of 2

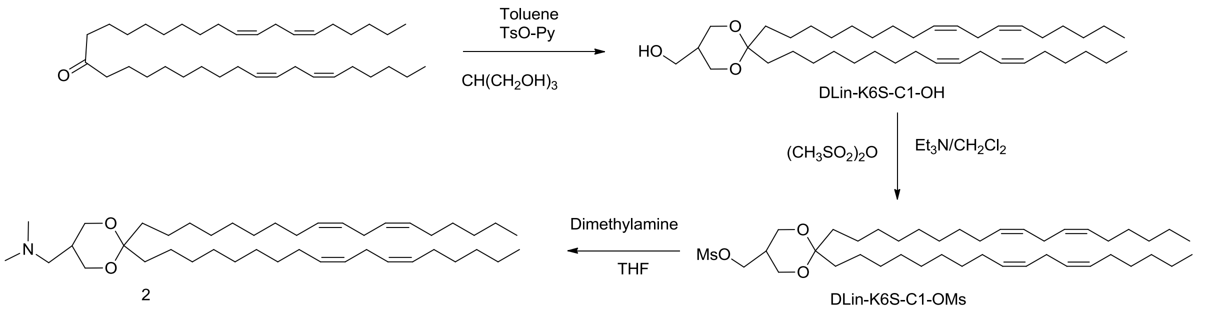

Synthesis of {2,2-bis[(9Z,12Z)-Octadeca-9,12-dien-1-yl]-1,3-dioxan-5-yl}methanol. (**DLin-K6S-C1-OH**). A mixture of (**DLin-ketone**, 1.05 g, 2.0 mmol), 2-hydroxymethyl-1,3-propanediol (490 mg, 4.2 mmol) and pyridinium p-toluenesulfonate (100 mg, 0.4 mmol) in 150 mL of toluene was refluxed under nitrogen overnight with a Dean-Stark tube to remove water. The resulting mixture was cooled to room temperature. The organic phase was washed with water (2 x 100 mL), brine (100 mL), and dried over anhydrous sodium sulfate. Evaporation of the solvent resulted in pale oil (1.2 g). The crude product was purified by column chromatography on silica gel (230-400 mesh, 100 mL) with 0-5% methanol gradient in dichloromethane as eluent. This afforded 0.93 g of pure **DLin-K6S-C1-OH** as pale oil.

Synthesis of {2,2-bis[(9Z,12Z)-Octadeca-9,12-dien-1-yl]-1,3-dioxan-5-yl}methyl methane sulfonate. (**DLin-K6S-C1-OMs**). To a solution of (**DLin-K6S-C1-OH**, 0.93 g, 1.5 mmol) and dry triethylamine (290 mg, 2.9 mmol) in 50 mL of anhydrous dichloromethane was added methanesulfonyl anhydride (400 mg, 2.3 mmol) under nitrogen. The resulting mixture was stirred at room temperature overnight. The organic phase was washed with water (2 x 75 mL), brine (75 mL), and dried over anhydrous sodium sulfate. The solvent was evaporated to afford 1.0 g of pale oil. The crude product was used in the following step without further purification.

Synthesis of ({2,2-bis[(9Z,12Z)-Octadeca-9,12-dien-1-yl]-1,3-dioxan-5-yl}methyl) dimethylamine. 2

To the above crude material (**DLin-K6S-C1-OMs**, 1.0 g) under nitrogen was added 20 mL of dimethylamine in tetrahydrofuran (2.0 M). The resulting mixture was stirred at room temperature for 7 days. An oily residual was obtained upon evaporation of the solvent. Column chromatography on silica gel (230-400 mesh, 100 mL) with 0-3% methanol gradient in chloroform as eluent resulted in 150 mg of the product **2** as pale oil. ^1H NMR (400 MHz, $CDCl_3$) δ: 5.24-5.51 (8, m, 4x CH=CH), 4.04 (2H, dd, 2 x OCH)), 3.75 (2H, dd OCH), 2.7-2.9 (2H, br, NCH_2), 2.78 (4H, t, 2 x C=C-CH_2-C=C), 2.57 (6H, s, 2 x NCH_3), 1.95-2.17 (9H, q, 4 x allylic CH_2 and CH), 1.67-1.95 (2H, m, CH_2), 1.54-1.65 (4H, m, 2 x CH_2), 1.22-1.45 (32H, m), 0.90 (6H, t, 2 x CH_3) ppm.

General procedure for the synthesis of 6-9, 11-13, 20-22, 25-29, 39, 43, 46, 50-52 and 54

The detailed synthesis of precursor Cbz compounds is described elsewhere.[17]

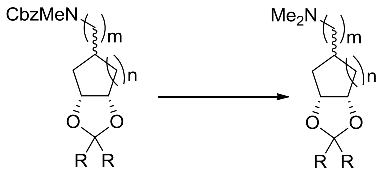

R = linoleyl

To a stirred suspension of the aminomethyl Cbz protected lipids (1 eq.) in THF (60 mL) $LiAlH_4$ (2 eq.) in 60 ml anhydrous THF was added slowly at 0 ^oC under nitrogen atmosphere. After the completion of the addition, reaction mixture was warmed to room temperature and then heated to reflux for 4 h. Progress of the reaction was monitored by TLC. After completion of reaction (by TLC) the mixture was cooled to 0 ^oC and quenched with careful addition of saturated Na_2SO_4 solution. Reaction mixture was stirred for 4 h at room temperature and filtered off. Residue was washed well with THF. The filtrate and washings were mixed and the volatilities were stripped off under vacuum to furnish the crude product was purified by flash column chromatography to obtain the pure product.

Synthesis of (3aR,5s,6aS)-N,N-dimethyl-2,2-di((9Z,12Z)-octadeca-9,12-dien-1-yl)tetrahydro-3aH-cyclopenta[d][1,3]dioxol-5-amine 6

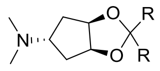

R = linoleyl

6

Using the general procedure compound **6** (1.3 g, 68%) was obtained as a colorless oil. ^{1}H NMR (500 MHz, CDCl3) δ 5.49 – 5.21 (m, 8H), 4.68 – 4.53 (m, 2H), 2.83 – 2.66 (m, 5H), 2.26 (d, J = 16.3 Hz, 6H), 2.16 – 1.95 (m, 10H), 1.72 – 1.59 (m, 2H), 1.56 – 1.45 (m, 2H), 1.46 – 1.15 (m, 39H), 0.94 – 0.82 (m, 6H). ^{13}C NMR (126 MHz, CDCl3) δ 130.15, 130.11, 130.09, 127.92, 127.91, 127.89, 127.88, 112.30, 79.30, 77.25, 76.99, 76.74, 64.40, 44.69, 38.32, 36.09, 35.38, 31.50, 29.93, 29.86, 29.68, 29.63, 29.60, 29.53, 29.50, 29.45, 29.32, 29.27, 27.22, 27.20, 27.17, 25.60, 24.52, 23.29, 22.55, 14.06. HRMS Calc for $C_{44}H_{80}NO_2$ $(MH)^+$ 654.6111; Found 654.6111.

Synthesis of (3aR,5r,6aS)-N,N-dimethyl-2,2-di((9Z,12Z)-octadeca-9,12-dien-1-yl)tetrahydro-3aH-cyclopenta[d][1,3]dioxol-5-amine 7

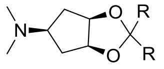

R = linoleyl

7

Prepared the compound **7** by following the general procedure using the corresponding *N*-Cbz-compound (0.28 g, 0.36 mmol, 1.0 eq) and 1M solution of LAH in THF (0.73 mL, 0.73 mmol,

2.0 eq), to get 0.14 g (62%) of the pure product **7**. ^{1}H NMR (400 MHz, CDCl3) δ 5.48 – 5.21 (m, 8H), 4.59 – 4.43 (m, 2H), 2.76 (tt, J = 14.4, 7.2, 4H), 2.49 – 2.32 (m, 1H), 2.24 (s, 6H), 2.04 (qt, J = 16.2, 8.1, 8H), 1.70 – 1.56 (m, 4H), 1.56 – 1.47 (m, 2H), 1.47 – 1.15 (m, 38H), 0.98 – 0.77 (m, 6H). . HRMS Calc for $C_{44}H_{80}NO_2$ $(MH)^+$ 654.6111; Found 654.6112.

Synthesis of (3aR,5R,7aS)-N,N-dimethyl-2,2-di((9Z,12Z)-octadeca-9,12-dien-1-yl)hexahydrobenzo[d][1,3]dioxol-5-amine 8

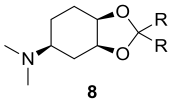

8

R = linoleyl

The *N*-Cbz-compound (1.8 g, 2.28 mmol) and 1M solution of LAH in THF (2.28 mL, 2.28 mmol, 2.0 eq), gave 0.65 g (85%) of the pure product **8.** . ^{1}H NMR (400 MHz, $CDCl_3$) δ 5.42 – 5.25 (m, 8H), 4.13 – 4.00 (m, 1H), 2.80 (t, *J* = 6.4, 4H), 2.30 (s, 6H), 2.18 – 1.95 (m, 10H), 1.87 – 1.59 (m, 9H), 1.45 – 1.16 (m, 37H), 0.98 – 0.79 (m, 6H). ^{13}C NMR (101 MHz, $CDCl_3$) δ 130.37, 130.35, 130.33, 128.13, 111.77, 77.54, 77.23, 74.68, 72.52, 60.20, 41.49, 38.60, 36.87, 32.29, 31.72, 30.21, 30.16, 29.86, 29.71, 29.54, 27.44, 27.42, 27.39, 25.94, 25.82, 24.66, 24.46, 22.77, 22.42, 14.39. Calc. mass for the $C_{45}H_{81}NO_2$: 668.13, found 668.5.

Synthesis of (3aS,5R,7aR)-N,N-dimethyl-2,2-di((9Z,12Z)-octadeca-9,12-dien-1-yl)hexahydrobenzo[d][1,3]dioxol-5-amine 9

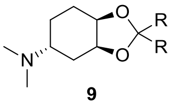

9

R = linoleyl

The *N*-Cbz-compound (0.9 g, 1.14 mmol) and 1M solution of LAH in THF (2.28 mL, 2.28 mmol, 2.0 eq), gave 0.65 g (85%) of the pure product **9**. ^{1}H NMR (400 MHz, $CDCl_3$) δ 5.48 – 5.22 (m, 8H), 4.33 (dd, *J* = 9.5, 4.1, 1H), 4.23 – 4.00 (m, 1H), 2.77 (t, *J* = 6.4, 4H), 2.65 (s, 1H), 2.34 (s, 6H), 2.18 – 1.95 (m, 9H), 1.86 (dd, *J* = 11.7, 5.7, 3H), 1.78 – 1.59 (m, 4H), 1.54 (d, *J* = 8.3, 2H), 1.45 – 1.16 (m, 37H), 0.98 – 0.79 (m, 6H). ^{13}C NMR (101 MHz, $CDCl_3$) δ 130.40, 130.35, 128.16, 128.13, 111.45, 77.54, 77.23, 76.91, 73.11, 72.94, 57.93, 54.76, 41.69, 38.22, 36.23, 32.09, 31.74, 30.21, 29.91, 29.74, 29.71, 29.57, 29.54, 29.02, 27.45, 27.41, 26.89, 25.84, 24.71, 24.38, 23.34, 22.79, 21.42, 14.30. Calc. mass for the $C_{45}H_{81}NO_2$: 668.13, found 668.5.

Pentane-1,3,5-triol. (**PTO**). Diethyl 3-hydroxyglutarate (1.0 g, 4.9 mmol) in anhydrous THF (10 mL) was added dropwise to a suspension of lithium aluminum hydride in anhydrous tetrahydrofuran (110 mL) under nitrogen with a cold water bath. Upon addition, the bath was removed and the suspension was stirred at room temperature for 2 days. The resulting mixture was quenched by adding 13 mL of brine very slowly with an ice-water bath. A white

suspension was resulted, and the mixture was stirred at room temperature overnight. The solid was filtered, and washed with tetrahydrofuran. The filtrate and wash were combined, and solvent evaporated to give 0.70 g of pale oil. Column chromatography of the crude product (230-400 mesh silica gel, 100 mL, 0-12% methanol gradient in chloroform) afforded 0.54 g of **PTO** as colourless oil.

2-{2,2-bis[(9Z,12Z)-Octadeca-9,12-dien-1-yl]-1,3-dioxan-4-yl}ethan-1-ol. (**DLin-K6A-C2-OH**). A mixture of **DLin-ketone** (0.80 g, 1.5 mmol), **PTO** (0.54 g, 4.5 mmol) and pyridinium p-toluenesulfonate (60 mg, 0.24 mmol) in 150 mL of toluene was refluxed under nitrogen overnight with a Dean-Stark tube to remove water. The resulting mixture was cooled to room temperature. The organic phase was washed with water (2 x 75 mL), brine (75 mL), and dried over anhydrous sodium sulphate. Evaporation of the solvent resulted in pale oil (1.1 g). The crude product was purified by column chromatography on silica gel (230-400 mesh, 75 mL) with 0-3% methanol gradient in dichloromethane as eluent. This afforded 0.75 g (79%) of pure **DLin-K6A-C2-OH** as colourless oil.

2-{2,2-bis[(9Z,12Z)-Octadeca-9,12-dien-1-yl]-1,3-dioxan-4-yl}ethyl methanesulfonate. (**DLin-K6A-C2-OMs**). To a solution of (**DLin-K6A-C2-OH**, 0.75 g, 1.2 mmol) and dry triethylamine (0.58 g, 5.7 mmol) in 40 mL of anhydrous dichloromethane was added methanesulfonyl anhydride (0.50 g, 2.9 mmol) under nitrogen. The resulting mixture was stirred at room temperature overnight. The organic phase was washed with water (2 x 50 mL), brine (50 mL), and dried over anhydrous sodium sulfate. The solvent was evaporated to afford 0.80 g of pale oil as a crude product. The crude product was used in the following step without further purification.

(2-{2,2-bis[(9Z,12Z)-Octadeca-9,12-dien-1-yl]-1,3-dioxan-4-yl}ethyl)dimethylamine 10

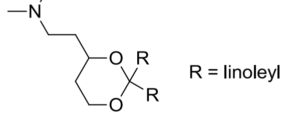

10

To the above crude material (**DLin-K6A-C2-OMs,** 0.80 g) under nitrogen was added 15 mL of dimethylamine in tetrahydrofuran (2.0 M). The resulting mixture was stirred at room temperature for 6 days. The solid was filtered. An oily residual was obtained upon evaporation of the solvent. Column chromatography on silica gel (230-400 mesh, 100 mL) with 0-6% methanol gradient in dichloromethane as eluent resulted in 0.70 g of the product **10** as pale oil. ^{1}H NMR (400 MHz, $CDCl_3$) δ: 5.28-5.45 (8, m, 4x CH=CH), 3.85-4.0 (2H, m, 2 x OCH), 3.78 (1H, dd, OCH), 2.78 (4H, t, 2 x C=C-CH_2-C=C), 2.55-2.90 (2H, br, NCH_2),

2.47 (6H, s, 2 x NCH_3), 2.05 (8H, q, 4 x allylic CH_2), 1.65-1.90 (4H, m, CH_2), 1.47-1.65 (4H, m, CH_2), 1.1-1.65 (36H, m), 0.90 (6H, t, 2 x CH_3).

Synthesis of (3aR,6aS)-5-methyl-2-((6Z,9Z)-octadeca-6,9-dien-1-yl)-2-((9Z,12Z)-octadeca-9,12-dien-1-yl)tetrahydro-3aH-[1,3]dioxolo[4,5-c]pyrrole 11

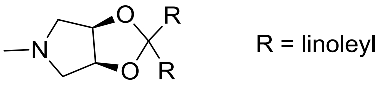

11

The *N*-Cbz-compound (2 g, 2.68 mmol) and 1M solution of LAH in THF (5.4 mL, 5.4 mmol, 2.0 eq), gave 1.32 g (79%) of the pure product **11** as a colorless oil. ^{1}H NMR ($CDCl_3$, 400 MHz) δ = 5.43 – 5.29 (m, 8H), 4.61 (s, 2H), 2.97 (d, *J* = 10.9 Hz, 2H), 2.77 (t, *J* = 6.4 Hz, 4H), 2.28 (s, 3H), 2.12 – 2.01 (m, 10H), 1.72 (m, 2H), 1.52 (br, 2H), 1.44 – 1.23 (m, 36H), 0.89 (t, *J* = 6.8 Hz, 6H); ^{13}C NMR δ = 130.2 (x3), 128.0, 127.9, 115.1, 80.2, 82.3, 41.9, 36.8, 35.9, 31.5, 30.0, 29.9, 29.7 (x2), 29.6, 29.5, 29.4, 29.3, 27.3, (x2), 27.2, 25.7, 24.3, 23.9, 22.6, 14.0; Electrospray MS (+ve): Molecular weight for $C_{42}H_{76}NO_2$ $(M + H)^+$ Calc. 626.6, Found 626.6.

Synthesis of (3aS,7aR)-5-methyl-2,2-di((9Z,12Z)-octadeca-9,12-dien-1-yl)hexahydro-[1,3]dioxolo[4,5-c]pyridine 12

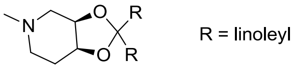

12

Using a procedure analogous to that described for the synthesis of compound **6**, compound **12** (0.90 g, 69%) was obtained as a colorless oil. ^{1}H NMR (400 MHz, $CDCl_3$) δ 5.49 – 5.23 (m, 8H), 4.16 (t, *J* = 5.2, 2H), 2.77 (t, *J* = 6.3, 4H), 2.70 (dd, *J* = 11.6, 5.0, 1H), 2.44 – 2.33 (m, 1H), 2.31 – 2.14 (m, 5H), 2.04 (dd, *J* = 13.6, 6.8, 8H), 1.99 – 1.88 (m, 1H), 1.78 – 1.63 (m, 2H), 1.56 (m, 2H), 1.48 – 1.15 (m, 37H), 0.88 (t, *J* = 6.7, 6H). ^{13}C NMR δ = 130.2, 130.1 (x2), 127.9 (x2), 72.1, 70.6, 68.1, 51.1, 46.3, 38.5, 36.5, 31.5, 30.0, 29.7, 29.6, 29.5 (x2), 29.3 (x3), 27.7, 27.2 (x2), 25.6, 24.4, 22.6, 14.1; Electrospray MS (+ve): Molecular weight for $C_{43}H_{78}NO_2$ $(M + H)^+$ Calc. 640.6, Found 640.6.

Synthesis of (3aR,8aS)-6-methyl-2,2-di((9Z,12Z)-octadeca-9,12-dien-1-yl)hexahydro-3aH-[1,3]dioxolo[4,5-d]azepine 13

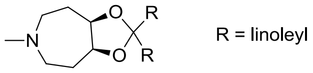

13

Using a procedure analogous to that described for the synthesis of compound **6**, compound **13** (1.04 g, 88 %) was isolated as a colorless oil. ^{1}H NMR (400 MHz, $CDCl_3$) δ 5.48 – 5.23 (m, 8H), 4.43 – 4.24 (m, 2H), 2.85 – 2.64 (m, 6H), 2.30 (s, 3H), 2.27 – 2.17 (m, 2H), 2.04 (q, *J* = 6.6, 8H), 2.00 – 1.84 (m, 4H), 1.65 (dd, *J* = 10.1, 6.0, 2H), 1.60 – 1.49 (m, 2H), 1.47 – 1.16 (m, 36H), 0.88 (t, *J* = 6.8, 6H); ^{13}C NMR (101 MHz, $CDCl_3$) δ 130.39, 130.34, 128.16, 128.12, 111.09, 53.90, 47.34, 36.84, 36.30, 31.74, 31.25, 30.23, 30.12, 29.90, 29.88, 29.86, 29.79, 29.74, 29.70, 29.57, 29.55, 29.52, 27.47, 27.44, 27.41, 25.84, 24.72, 23.63, 22.79, 14.30; Electrospray MS (+ve): Molecular weight for $C_{44}H_{79}NO_2$ $(M + Na)^+$ Calc. 654.6, Found 654.4.

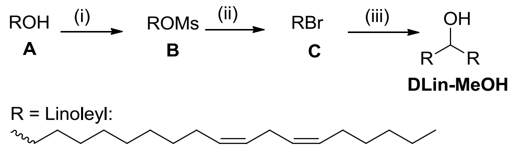

(i) MsCl, NEt_3, DCM; (ii) $MgBr.Et_2O$, Et_2O (iii) a) Mg, Et_2O then HCOOEt; b) NaOMe

Synthesis of methanesulfonic acid octadeca-9, 12-dienyl ester B

To a solution of the alcohol **1** (26.6 g, 100 mmol) in dichloromethane (100 mL), triethylamine (13.13 g, 130 mmol) was added and this solution was cooled in an ice-bath. To this cold solution, a solution of mesyl chloride (12.6 g, 110 mmol) in dichloromethane (60 mL) was added dropwise and after the completion of the addition, the reaction mixture was allowed to warm to ambient temperature and stirred overnight. The TLC of the reaction mixture showed the completion of the reaction. The reaction mixture was diluted with dichloromethane (200 mL), washed with water (200 mL), satd. $NaHCO_3$ (200 mL), brine (100 mL) and dried ($NaSO_4$). The organic layer was concentrated to get the crude product which was purified by column chromatography (silica gel) using 0-10% Et_2O in hexanes. The pure product fractions were combined and concentrated to obtain the pure product **B** as colorless oil (30.6 g, 89%). ^{1}H NMR ($CDCl_3$, 400 MHz) δ 5.42-5.21 (m, 4H), 4.20 (t, 2H), 3.06 (s, 3H), 2.79 (t, 2H), 2.19-2.00 (m, 4H), 1.90-1.70 (m, 2H), 1.06-1.18 (m, 18H), 0.88 (t, 3H). ^{13}C NMR ($CDCl_3$) δ 130.76, 130.54, 128.6, 128.4, 70.67, 37.9, 32.05, 30.12, 29.87, 29.85, 29.68, 29.65, 29.53, 27.72, 27.71, 26.15, 25.94, 23.09, 14.60. MS. Molecular weight calculated for $C_{19}H_{36}O_3S$, 344.53, Found 343.52 $(M-H^-)$.

Synthesis of 18-Bromo-octadeca-6, 9-diene C

The mesylate **B** (13.44 g, 39 mmol) was dissolved in anhydrous ether (500 mL) and to it the MgBr.Et_2O complex (30.7 g, 118 mmol) was added under argon and the mixture was refluxed under argon for 26 h after which the TLC showed the completion of the reaction. The reaction mixture was diluted with ether (200 mL) and ice-cold water (200 mL) was added to this mixture and the layers were separated. The organic layer was washed with 1% aqueous K_2CO_3 (100 mL), brine (100 mL) and dried (anhyd. Na_2SO_4). Concentration of the organic layer provided the crude product which was further purified by column chromatography (silica gel) using 0-1% Et_2O in hexanes to isolate the bromide **C** (12.6 g, 94 %) as a colorless oil. ^{1}H NMR ($CDCl_3$, 400 MHz) δ 5.41-5.29 (m, 4H), 4.20 (d, 2H), 3.40 (t, J = 7 Hz, 2H), 2.77 (t, J = 6.6 Hz, 2H), 2.09-2.02 (m, 4H), 1.88-1.00 (m, 2H), 1.46-1.27 (m, 18H), 0.88 (t, J = 3.9 Hz, 3H). ^{13}C NMR ($CDCl_3$) δ 130.41, 130.25, 128.26, 128.12, 34.17, 33.05, 31.75, 29.82, 29.57, 29.54, 29.39, 28.95, 28.38, 27.42, 27.40, 25.84, 22.79, 14.28.

Synthesis of (6Z,9Z,28Z,31Z)-heptatriaconta-6,9,28,31-tetraen-19-ol. DLin-MeOH

To a flame dried 500 mL RB flask, freshly activated Mg turnings (2.4 g, 100 mmol) were added and the flask was equipped with a magnetic stir bar, an addition funnel and a reflux condenser. This set-up was degassed and flushed with argon and 10 mL of anhydrous ether was added to the flask via syringe. The bromide **C** (26.5 g, 80.47 mmol) was dissolved in anhydrous ether (50 mL) and added to the addition funnel. About 5 mL of this ether solution was added to the Mg turnings while stirring vigorously. An exothermic reaction was noticed (to confirm/accelerate the Grignard reagent formation, 5 mg of iodine was added and immediate decolorization was observed confirming the formation of the Grignard reagent) and the ether started refluxing. The rest of the solution of the bromide was added dropwise while keeping the reaction under gentle reflux by cooling the flask in water. After the completion of the addition the reaction mixture was kept at 35 ^{o}C for 1 h and then cooled in ice bath. Ethyl formate (2.68 g, 36.2 mmol) was dissolved in anhydrous ether (40 mL) and transferred to the addition funnel and added dropwise to the reaction mixture with stirring. An exothermic reaction was observed and the reaction mixture started refluxing. After the initiation of the reaction the rest of the ethereal solution of formate was quickly added as a stream and the reaction mixture was stirred for a further period of 1 h at ambient temperature. The reaction was quenched by adding 10 mL of acetone dropwise followed by ice cold water (60 mL). The reaction mixture was treated with aq. H_2SO_4 (10 % by volume, 300 mL) until the solution became homogeneous and the layers were separated. The aq. phase was extracted

with ether (2x100 mL). The combined ether layers were dried (Na_2SO_4) and concentrated to get the crude product which was treated with 1 g of sodium in methanol (200 mL) at room temperature overnight. Upon completion of the reaction, most of the solvent was evaporated. The resulting mixture was poured into 150 mL of 5% hydrochloric acid solution. The aqueous phase was extracted with ether (2 x 150 mL). The combined ether extract was washed with water (2 x 100 mL), brine (100 mL), and dried over anhydrous sodium sulfate. Evaporation of the solvent gave the crude product which was purified by column (silica gel, 0-10% ether in hexanes) chromatography and the pure product fractions were evaporated to provide the product **6b** as a colorless oil (16.4 g, 85%). NMR (400 MHz,$CDCl_3$) δ 5.47 – 5.24 (m, 8H), 3.56 (dd, *J* = 6.8, 4.2, 1H), 2.85 – 2.66 (m, 4H), 2.12 – 1.91 (m, 9H), 1.50 – 1.17 (m, 46H), 0.98 – 0.76 (m, 6H). ^{13}C NMR (101 MHz, $CDCl_3$) δ 130.41, 130.37, 128.18, 128.15, 77.54, 77.22, 76.91, 72.25, 37.73, 31.75, 29.94, 29.89, 29.83, 29.73, 29.58, 29.53, 27.46, 27.43, 25.89, 25.86, 22.80, 14.30.

Synthesis of (6Z,9Z,28Z,31Z)-heptatriaconta-6,9,28,31-tetraen-19-yl 2-(dimethylamino)acetate 14

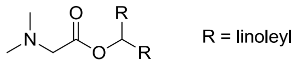

14

A solution of **DLin-MeOH** (0.50 g), dimethylglycine hydrochloride (0.53 g), 4-*N*,*N*-dimethylaminopyridine (0.60g) and 1-ethyl-3-(3-dimethylaminopropyl)carbodiimide hydrochloride (0.50 g) in dichloromethane (5 mL) was stirred at room temperature overnight. The solution was washed with dilute hydrochloric acid followed by dilute aqueous sodium bicarbonate. The organic fractions were dried over anhydrous magnesium sulphate, filtered and the solvent removed on a rotovap. The residue was passed down a silica gel column (20 g) using a 0-3% methanol/dichloromethane elution gradient. Fractions containing the purified product were combined and the solvent removed, yielding **14** as a colorless oil (0.35 g). ^{1}H NMR (400MHz, $CDCl_3$) δ: 5.36 (m; 8H); 4.97 (p; J=6.2Hz, 1H); 3.22 (s; 2H); 2.79 (t; J=6.2Hz, 4H); 2.42 (s; 6H); 2.07 (m; 8H); 0.91 (t; J=6.8Hz, 6H)

Synthesis of (6Z,9Z,28Z,31Z)-heptatriaconta-6,9,28,31-tetraen-19-yl 3-(dimethylamino)propanoate 15

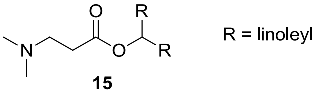

15

A solution of **DLin-MeOH** (0.51 g), 4-N,N-dimethylaminopropanoic acid (0.25 g), 4-N,N-dimethylaminopyridine (0.48g) and 1-ethyl-3-(3-dimethylaminopropyl)carbodiimide

hydrochloride (0.21 g) in dichloromethane (10 mL) was stirred at room temperature overnight. The solution was washed with dilute hydrochloric acid followed by dilute aqueous sodium bicarbonate. The organic fractions were dried over anhydrous magnesium sulphate, filtered and the solvent removed on a rotovap. The residue was passed down a silica gel column (20 g) using a 0-3% methanol/dichloromethane elution gradient. Fractions containing the purified product were combined and the solvent removed, yielding **15** as a colorless oil (0.40 g). ^{1}H NMR (400MHz, $CDCl_3$) δ: 5.37 (m, 8H); 4.90 (p, *J*=6.2Hz, 1H); 2.79 (t, *J*=6.4Hz, 4H); 2.68 (t, *J*=7.2Hz, 2H); 2.52 (t, *J*=7.2Hz, 2H); 2.30 (s, 6H); 2.07 (m, 8H); 0.91 (t, *J*=6.7Hz; 6H)

Synthesis of [6*Z*,9*Z*,28*Z*,31*Z*)-heptatriaconta-6,9,28,31-tetraen-19-yl-4-(dimethylamino) butanoate] 16

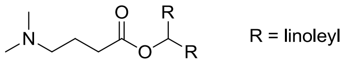

16

The DLin-MeOH (144 g, 272 mmol) was dissolved in 1 L of dichloromethane and to it the hydrochloride salt of dimethylaminobutyric acid **7** (55 g, 328 mmol) was added followed by diisopropylethylamine (70 mL) and DMAP (4 g). After stirring for 5 min. at ambient temperature, EDCI (80 g, 417 mmol) was added and the reaction mixture was stirred at room temperature overnight after which the TLC (silica gel, 5% MeOH in CH_2Cl_2) analysis showed complete disappearance of the starting alcohol. The reaction mixture was diluted with CH_2Cl_2 (500 mL) and washed with saturated $NaHCO_3$ (400 mL), water (400 mL) and brine (500 mL). The combined organic layers were dried over anhyd. Na_2SO_4 and solvents were removed *in vacuo*. The crude product (180 g) thus obtained was purified by Flash column chromatography [2.5 Kg silica gel, Using the following eluents i) column packed with 6L of 0.1% NEt_3 in DCM; after loading ii) 4 L of 0.1% NEt_3 in DCM; iii) 16L of 2% MeOH – 98% of 0.1% NEt_3 in DCM; iv) 4L of 2.5% MeOH – 97.5% of 0.1% NEt_3 in DCM; v) 12L of 3% MeOH – 97% of 0.1% NEt_3 in DCM] to isolate the pure product **16** (159 g, 91%) as a colorless oil. ^{1}H NMR (400 MHz, $CDCl_3$): δ 5.46 – 5.23 (m, 8H), 4.93 – 4.77 (m, 1H), 2.83 – 2.66 (m, 4H), 2.37 – 2.22 (m, 4H), 2.20 (s, 6H), 2.10 – 1.96 (m, 9H), 1.85 – 1.69 (m, 2H), 1.49 (d, *J* = 5.4, 4H), 1.39 – 1.15 (m, 39H), 0.95 – 0.75 (m, 6H). ^{13}C NMR (101 MHz, $CDCl_3$): δ 173.56, 130.38, 130.33, 128.17, 128.14, 77.54, 77.22, 76.90, 74.44, 59.17, 45.64, 34.36, 32.69, 31.73, 29.87, 29.76, 29.74, 29.70, 29.56, 29.50, 27.44, 27.41, 25.84, 25.55, 23.38, 22.78, 14.27. EI-MS (+ve): MW calc. for $C_{43}H_{79}NO_2$ $(M + H)^+$: 642.6, found: 642.6.

Synthesis of 5-(dimethylamino)pentanoic acid. **(DMAPA)**. 5-Bromovaleric acid (2 g) was dissolved in a 20% aqueous solution of dimethylamine and stirred at room temperature overnight. The solvent was removed on a rotovap and the residue dissolved in water (10 mL). Sodium bicarbonate (1.2 g) was added and the solvent removed on a rotovap. The residue was suspended in ethanol, filtered and the solvent removed. The remaining residue was then dissolved in methylene chloride, filtered and the solvent removed, yielding **(DMAPA)** as a yellow viscous oil (1.3 g).

Synthesis of (6Z,9Z,28Z,31Z)-heptatriaconta-6,9,28,31-tetraen-19-yl 5-(dimethylamino) pentanoate 17

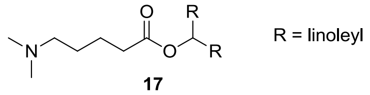

17

A solution of **DLin-MeOH** (0.50 g), **(DMAPA)** (1.3 g), 4-N,N-dimethylaminopyridine (1.35 g) and 1-ethyl-3-(3-dimethylaminopropyl)carbodiimide hydrochloride (0.53 g) in dichloromethane (40 mL) was stirred at room temperature overnight. The solution was washed with dilute hydrochloric acid followed by dilute aqueous sodium bicarbonate. The organic fractions were dried over anhydrous magnesium sulphate, filtered and the solvent removed on a rotovap. The residue was passed down a silica gel column (20 g) using a 0-4% methanol/dichloromethane elution gradient. Fractions containing the purified product were combined and the solvent removed, yielding **17** as a colorless oil (0.36 g). ^{1}H NMR (400MHz, $CDCl_3$) δ: 5.37 (m, 8H); 4.88 (p, *J*=6.1Hz, 4H); 2.79 (t, *J*=6.4Hz, 4H); 2.38 (m, 2H); 2.34 (m, 2H); 2.31 (s, 6H); 2.07 (m, 8H); 0.91 (t, *J*=6.8Hz; 6H)

Synthesis of 6-(dimethylamino)hexanoic acid. **(DMAHA)**. 6-Bromohexanoic acid (2.3 g) was dissolved in a 20% aqueous solution of dimethylamine and stirred at room temperature overnight. The solvent was removed on a rotovap and the residue dissolved in water (10 mL). Sodium bicarbonate (1.0 g) was added and the solvent removed on a rotovap. The residue was suspended in ethanol, filtered and the solvent removed. The remaining residue was then dissolved in toluene, filtered and the solvent removed, yielding **(DMAHA)** as a semicrystalline solid (1.5 g).

Synthesis of (6Z,9Z,28Z,31Z)-heptatriaconta-6,9,28,31-tetraen-19-yl 6-(dimethylamino) hexanoate 18.

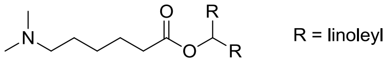

18

A solution of **DLin-MeOH** (0.50 g), **(DMAHA)** (0.58 g), 4-N,N-dimethylaminopyridine (0.60 g) and 1-ethyl-3-(3-dimethylaminopropyl)carbodiimide hydrochloride (0.59 g) in dichloromethane (25 mL) was stirred at room temperature overnight. The solution was washed with dilute hydrochloric acid followed by dilute aqueous sodium bicarbonate. The organic fractions were dried over anhydrous magnesium sulphate, filtered and the solvent removed on a rotovap. The residue was passed down a silica gel column (20 g) using a 0-6% methanol/dichloromethane elution gradient. Fractions containing the purified product were combined and the solvent removed, yielding **18** as a colorless oil (0.41 g). ^{1}H NMR (400MHz, $CDCl_3$) δ: 5.37 (m; 8H); 4.88 (p; J=6.1Hz; 1H); 2.79 (t; J=6.4Hz; 4H); 2.47 (m; 2H); 2.40 (s; 6H); 2.31 (t; J=7.4Hz; 2H); 2.07 (m; 8H); 0.91 (t; J=6.8Hz; 6H).

Synthesis of hexane-1,3,6-triol. (**HTO**).

Diethyl β-ketoadipate (1.86 g, 8.6 mmol) was added dropwise to a suspension of lithium aluminum hydride in anhydrous tetrahydrofuran (90 mL) under argon with an ice-water bath. Upon addition, the bath was removed and the suspension was stirred at room temperature overnight. The resulting mixture was quenched by adding 10 mL of brine very slowly with an ice-water bath. A white suspension was resulted, and the mixture was stirred at room temperature overnight. The solid was filtered, and washed with tetrahydrofuran followed by ethanol (2 x 50 mL). The filtrate and wash were combined, and solvent evaporated to give 0.90 g of pale oil. Column chromatography of the crude product (230-400 mesh SiO_2, 100 mL, 0-10% methanol gradient in dichloromethane) afforded 0.70 g of **HTO** as colourless oil.

Synthesis of 6Z,9Z,28Z,31Z)-heptatriaconta-6,9,28,31-tetraen-19-one. (**DLin-ketone**).

To a mixture of **DLin-MeOH** (4.0 g, 7.2 mmol) and anhydrous potassium carbonate (0.4 g) in 100 mL of dichloromethane was added pyridinium chlorochromate (4.0 g, 19 mmol). The resulting suspension was stirred at room temperature for 2 hours. Ether (300 mL) was then added into the mixture, and the resulting brown suspension was filtered through a pad of silica gel (150 mL). The silica gel pad was further washed with ether (3 x 75 mL). The ether filtrate and washes were combined. Evaporation of the solvent gave 5.1 g of an oily residual as a crude product. The crude product was purified by column chromatography on silica gel (230-400 mesh, 200 mL) eluted with 0-4% ethyl acetate in hexanes. This afforded 3.0 g (79%) of (**DLin-ketone**). ^{1}H NMR ($CDCl_3$, 400 MHz) δ 5.33-5.21 (m, 8H), 2.69 (t, 4H), 2.30 (t, 4H), 2.05-1.95 (m, 8H), 1.55-1.45 (m, 2H), 1.35-1.15 (m, 18H), 0.82 (t, 3H). ^{13}C NMR ($CDCl_3$) δ 211.90, 130.63, 130.54, 128.47, 128.41, 43.27, 33.04, 32.01, 30.93, 29.89, 29.86,

29.75, 29.74, 27.69, 26.11, 24.35, 23.06, 14.05. MS. Molecular weight calculated for $C_{37}H_{66}O$, Cal. 526.92, Found 528.02 ($M+H^+$).

Synthesis of 3-{2,2-bis[(9Z,12Z)-Octadeca-9,12-dien-1h-yl]-1,3-dioxan-4-yl}propan-1-ol. (**DLin-K6A-C3-OH**).

A mixture of dilinoleyl ketone (**DLin-ketone**, 1.80 g, 3.4 mmol), **HTO** (0.50 g, 3.7 mmol) and pyridinium p-toluenesulfonate (100 mg, 0.40 mmol) in 120 mL of toluene was refluxed under argon for 3 hours with a Dean-Stark tube to remove water. The resulting mixture was cooled to room temperature. The organic phase was washed with water (2 x 50 mL), brine (50 mL), and dried over anhydrous sodium sulfate. Evaporation of the solvent resulted in pale oil (2.0 g). The crude product was purified by column chromatography on silica gel (230-400 mesh, 50 mL) with 0-3% methanol gradient in dichloromethane as eluent. This afforded 0.90 g (41%) of pure **DLin-K6A-C3-OH** as colourless oil.

Synthesis of 3-{2,2-bis[(9Z,12Z)-Octadeca-9,12-dien-1-yl]-1,3-dioxan-4-yl}propyl methanesulfonate. (**DLin-K6A-C3-OMs**).

To a solution of **DLin-K6A-C3-OH** (0.97 g, 1.5 mmol) and dry triethylamine (0.44 g, 4.3 mmol) in 60 mL of anhydrous dichloromethane was added methanesulfonyl anhydride (0.60 g, 3.5 mmol) under argon. The resulting mixture was stirred at room temperature overnight. The organic phase was washed with water (2 x 30 mL), brine (30 mL), and dried over anhydrous magnesium sulfate. The solvent was evaporated to afford 1.1 g of pale oil as a crude product. The crude product was used in the following step without further purification.

Synthesis of (3-{2,2-bis[(9Z,12Z)-Octadeca-9,12-dien-1-yl]-1,3-dioxan-4-yl}propyl) dimethylamine. 19.

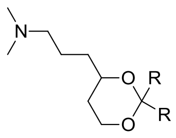

R = linoleyl

19

To the above crude material (**DLin-K6A-C3-OMs,** 1.1 g) under argon was added 20 mL of dimethylamine in tetrahydrofuran (2.0 M). The resulting mixture was stirred at room temperature for 5 days. The solid was filtered. An oily residual was obtained upon evaporation of the solvent. Column chromatography on silica gel (230-400 mesh, 40 mL) with 0-7% methanol gradient in dichloromethane as eluent resulted in 0.85 g of the product **19** as pale oil. ^1H NMR (400 MHz, $CDCl_3$) δ: 5.25-5.45 (8, m, 4 x CH=CH), 3.7-4.0 (3H, m, 3x OCH), 2.77 (4H, t, 2 x C=C-CH_2-C=C), 2.5-2.8 (2H, br, NCH_2), 2.5 (6H, s, 2 x NCH_3),

2.05 (8H, q, 4 x allylic CH_2), 1.65-1.90 (4H, m, 2 x CH_2), 1.40-1.65 (4H, m, 2 x CH_2), 1.1-1.65 (38H, m), 0.90 (6H, t, 2 x CH_3).

Synthesis of 1-((3aR,5r,6aS)-2,2-di((9Z,12Z)-octadeca-9,12-dien-1-yl)tetrahydro-3aH-cyclopenta[d][1,3]dioxol-5-yl)-N,N-dimethylmethanamine 20

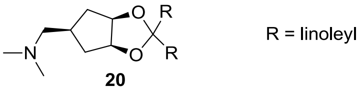

20

Prepared the compound **20** by following similar procedure as compound **7**, using *N*-Cbz-compound (0.21 g, 0.27 mmol, 1.0 eq) and 1M solution of LAH in THF (0.4 mL, 0.4 mmol, 1.5 eq), gave 0.09 g (50%) of the pure product **20**. ^{1}H NMR (400 MHz, $CDCl_3$) δ 5.53 – 5.16 (m, 8H), 4.68 – 4.46 (m, 2H), 2.79 (t, *J* = 6.6, 4H), 2.37 (d, *J* = 7.1, 2H), 2.24 (d, *J* = 5.1, 6H), 2.16 – 1.94 (m, 11H), 1.73 – 1.64 (m, 2H), 1.58 (ddd, *J* = 20.2, 10.4, 6.8, 4H), 1.48 – 1.21 (m, 37H), 0.91 (dd, *J* = 8.8, 5.1, 6H). ^{13}C NMR (101 MHz, $CDCl_3$) δ 130.19, 130.17, 130.15, 127.95, 127.94, 127.93, 115.27, 80.90, 77.34, 77.02, 76.70, 65.01, 45.83, 37.40, 37.10, 31.55, 31.54, 31.53, 29.99, 29.70, 29.68, 29.58, 29.56, 29.50, 29.36, 29.35, 29.32, 27.26, 27.24, 27.21, 25.64, 24.42, 23.83, 22.59, 14.10. Calc. mass for the $C_{45}H_{81}NO_2$: 668.1, found 668.5.

Synthesis of 1-((3aR,5s,6aS)-2,2-di((9Z,12Z)-octadeca-9,12-dien-1-yl)tetrahydro-3aH-cyclopenta[d][1,3]dioxol-5-yl)-N,N-dimethylmethanamine 21

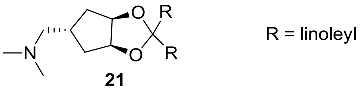

21

Prepared the compound **21** by following similar procedure as compound **7**, using *N*-Cbz-compound (2.1 g, 2.66 mmol, 1.0 eq) and 1M solution of LAH in THF (4.0 mL, 4.0 mmol, 1.5 eq), gave 1.2 g (68%) of the pure product **21**. ^{1}H NMR (400 MHz, $CDCl_3$) δ 5.57 – 5.21 (m, 8H), 4.67 – 4.53 (m, 2H), 2.79 (t, *J* = 6.5, 4H), 2.52 – 2.33 (m, 1H), 2.23 (m, 8H), 2.14 – 1.94 (m, 10H), 1.66 (dd, *J* = 10.1, 6.1, 2H), 1.58 – 1.22 (m, 39H), 1.22 – 1.07 (m, 2H), 0.91 (t, *J* = 6.9, 6H). ^{13}C NMR (101 MHz, $CDCl_3$) δ 130.19, 130.17, 130.14, 127.96, 127.94, 127.93, 112.24, 80.36, 77.34, 77.02, 76.70, 64.27, 45.80, 38.75, 36.15, 35.85, 34.67, 31.54, 30.01, 29.88, 29.71, 29.69, 29.68, 29.58, 29.53, 29.49, 29.37, 29.36, 29.31, 27.27, 27.24, 27.21, 25.64, 24.63, 23.17, 22.59, 22.58, 14.10. Calc. mass for the $C_{45}H_{81}NO_2$: 668.1, found 668.5.

Synthesis of 8-methyl-2,2-di((9Z,12Z)-octadeca-9,12-dien-1-yl)-1,3-dioxa-8-azaspiro[4.5]decane 22

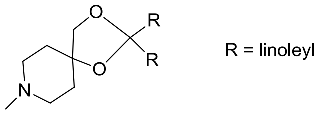

22

Using our general procedure using *N*-Cbz-compound (1.44 g, 1.86 mmol, 1.0 eq), 1M LAH in THF (3.72 mL, 3.72 mmol, 2.0 eq), which gave 1.04 g (86%) of the pure produc. ^{1}H NMR (400 MHz, $CDCl_3$) δ 5.51 – 5.16 (m, 8H), 3.75 (s, 2H), 2.77 (t, *J* = 6.4, 4H), 2.55 (s, 2H), 2.33 (s, 1H), 2.28 (s, 3H), 2.04 (q, *J* = 6.8, 8H), 1.88 – 1.74 (m, 2H), 1.74 – 1.63 (m, 2H), 1.57 (dd, *J* = 10.2, 5.4, 4H), 1.45 – 1.15 (m, 37H), 0.88 (t, *J* = 6.8, 6H). ^{13}C NMR (101 MHz, $CDCl_3$) δ 130.40, 130.36, 128.15, 128.13, 112.67, 78.12, 77.55, 77.23, 76.91, 73.59, 53.33, 46.31, 37.93, 36.66, 31.74, 30.17, 29.90, 29.79, 29.73, 29.57, 29.53, 27.45, 27.41, 25.84, 24.32, 22.80, 14.31. Calc. mass for the $C_{44}H_{79}NO_2$: 653.6, found 654.5.

Synthesis of 2-(2,2-di((9Z,12Z)-octadeca-9,12-dien-1-yl)-1,3-dioxolan-4-yl)-N-methyl-N-(pyridin-3-ylmethyl)ethanamine 23

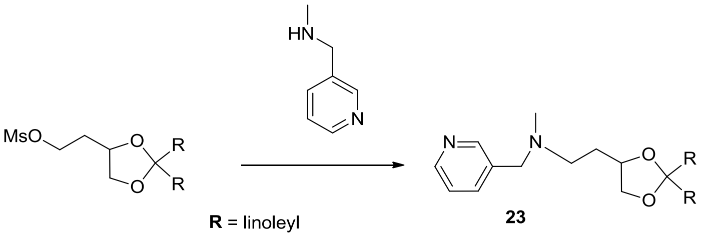

To a solution of the mesylate (0.693 g, 1 mmol) in ethanol (3 mL) the pyridinium amine (0.122 g, 1 mmol) was added and the mixture was microwaved at 130°C for 1 hour. The reaction mixture was concentrated and the thus crude product was purified by column chromatography (24 g silica cardridge in combifalsh Rf purification system) to obtain the pure product **23** (310 mg, 43%) as a light yellow oil. ^{1}H NMR (400 MHz, CDCl3) δ 8.61 – 8.35 (m, 2H), 7.65 (d, J = 7.8 Hz, 1H), 7.24 (dd, J = 7.5, 4.6 Hz, 1H), 5.52 – 5.16 (m, 8H), 4.24 – 3.95 (m, 2H), 3.57 – 3.37 (m, 4H), 2.86 – 2.64 (m, 4H), 2.62 – 2.30 (m, 2H), 2.18 (s, 3H), 2.10 – 1.92 (m, 9H), 1.78 (dtd, J = 65.4, 13.8, 6.4 Hz, 3H), 1.57 (dd, J = 15.9, 9.1 Hz, 5H), 1.44 – 1.05 (m, 40H), 0.88 (t, J = 6.8 Hz, 6H). ^{13}C NMR (101 MHz, CDCl3) δ 150.53, 148.81, 136.70, 134.65, 130.40, 130.37, 128.15, 128.13, 123.51, 112.23, 77.54, 77.23, 76.91, 74.89, 70.17, 59.90, 54.27, 42.25, 38.01, 37.69, 31.74, 31.67, 30.20, 30.15, 29.90, 29.84, 29.76, 29.57, 29.55, 27.46, 27.42, 25.84, 24.26, 23.95, 22.80, 14.32. Calc. mass for the $C_{48}H_{82}N_2O_2$: 719.18, found 719.5.

Synthesis of 1,3-bis(9Z,12Z)-Octadeca-9,12-dien-1-yl propanedioate. (**DLin-mal**). To a solution of linoleyl alcohol (**Lin-OH,** 5.0 g, 19 mmol) in anhydrous dichloromethane (70 mL) was added dropwise malonyl dichloride (1.36 g, 9.3 mmol) under argon at 0-5°C. The resulting mixture was stirred at room temperature for 6 hours. The mixture was diluted with 50 mL of dichloromethane. The organic phase was washed with water (3 x 75 mL), brine (75 mL) and dried over anhydrous sodium sulfate. Evaporation of the solvent gave a brownish

oily residual (5.8 g). The crude product was purified by column chromatography on silica gel (230-400 mesh, 200 mL) with 0-4% ethyl acetate gradient in hexanes as eluent. This afforded 3.1 g (55%) of pure **DLin-mal** as colourless oil. ^{1}H NMR (400 MHz, $CDCl_3$) δ 5.25-5.45 (8, m, 4x CH=CH), 4.13 (4H, t, 2 x OCH_2), 3.35 (2H, s, CO-CH_2-CO), 2.78 (4H, t, 2 x C=C-CH_2-C=C), 2.05 (8H, q, 4 x allylic CH_2), 1.55-1.65 (4H, m, CH_2), 1.2-1.4 (32H, m), 0.90 (6H, t, 2 x CH_3).

Synthesis of 1,3-bis(9Z,12Z)-Octadeca-9,12-dien-1-yl 2-[2-(dimethylamino)ethyl] propanedioate. **24.**

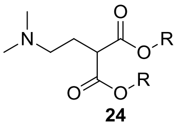

24

R = linoleyl

To a suspension of sodium hydride (0.17 g, 60%, 4.1 mmol) in anhydrous benzene (40 mL) was added **DLin-mal** (0.50 g, 0.83 mmol**)** under argon. The resulting suspension was stirred at room temperature 60 min. To the resulting mixture was added N,N-dimethylaminoethyl chloride hydrochloride (0.12 g, 0.83 mmol) in one portion, and the resulting mixture was refluxed under argon for 2 days. The organic phase was washed with water (3 x 20 mL), brine (2 x 25 mL) and dried over anhydrous sodium sulfate. Evaporation of the solvent gave a pale oily residual (0.50 g). Column chromatography on silica gel (230-400 mesh, 40 mL) with 0-4% methanol in dichloromethane as eluent resulted in 0.13 g of the product **24** as pale oil. ^{1}H NMR (400 MHz, $CDCl_3$) δ: 5.25-5.40 (8, m, 4 x CH=CH), 4.05-4.20 (4H, m, 2 x OCH_2), 3.47 (1H, t, CO-CH-CO), 2.75 (4H, t, 2 x C=C-CH_2-C=C), 2.35-2.9 (6H, br, 2 x NCH_3), 2.15-2.35 (2H, br, NCH_2), 2.05 (8H, q, 4 x allylic CH_2), 1.55-1.65 (4H, m, CH_2), 1.2-1.45 (32H, m), 0.90 (6H, t, 2 x CH_3).

Synthesis of N,N-dimethyl-1-((3aR,5R,7aS)-2-((8Z,11Z)-octadeca-8,11-dien-1-yl)-2-((9Z,12Z)-octadeca-9,12-dien-1-yl)hexahydrobenzo[d][1,3]dioxol-5-yl)methanamine 25

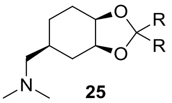

25

R = linoleyl

Prepared the compound **25** by following similar procedure as described above, using *N*-Cbz-compound (0.28 g, 0.35 mmol, 1.0 eq) and 1M solution of LAH in THF (0.52 mL, 0.52 mmol, 1.5 eq), gave 0.15 g (66%) of the pure product **25.** ^{1}H NMR (400 MHz, $CDCl_3$) δ 5.48 – 5.18 (m, 8H), 4.29 – 4.16 (m, 1H), 4.06 (dt, *J* = 8.3, 5.6, 1H), 2.76 (t, *J* = 6.4, 4H), 2.18 (s, 6H), 2.14 – 1.91 (m, 11H), 1.90 – 1.60 (m, 5H), 1.60 – 1.46 (m, 3H), 1.46 – 1.13 (m, 38H), 0.94 – 0.76 (m, 6H). ^{13}C NMR (101 MHz, $cdcl_3$) δ 130.40, 130.38, 128.16, 111.29, 77.54, 77.23,

76.91, 74.02, 73.13, 66.44, 46.15, 38.44, 36.58, 32.07, 31.75, 30.25, 29.92, 29.90, 29.78, 29.75, 29.72, 29.57, 29.55, 29.54, 28.82, 28.52, 27.47, 27.43, 26.03, 25.86, 24.74, 24.42, 22.79, 14.29 Calc. mass for the $C_{46}H_{83}NO_2$: 682.1, found 682.5.

Synthesis of N,N-dimethyl-1-((3aR,5S,7aS)-2-((8Z,11Z)-octadeca-8,11-dien-1-yl)-2-((9Z,12Z)-octadeca-9,12-dien-1-yl)hexahydrobenzo[d][1,3]dioxol-5-yl)methanamine 26

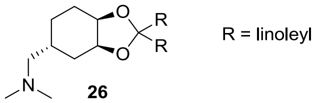

26

Prepared the compound **26** by following a similar procedure using *N*-Cbz-compound (0.21 g, 0.26 mmol, 1.0 eq) and 1M solution of LAH in THF (0.4 mL, 0.4 mmol, 1.5 eq). The pure product **26** (0.08, 50%) was isolated as an oil**.** ^{1}H NMR (400 MHz, $CDCl_3$) δ 5.50 – 5.19 (m, 8H), 4.16 (dd, *J* = 8.2, 4.2, 1H), 4.05 (dt, *J* = 9.7, 6.1, 1H), 2.76 (t, *J* = 6.4, 4H), 2.16 (d, *J* = 10.3, 6H), 2.10 – 1.97 (m, 10H), 1.95 – 1.83 (m, 1H), 1.76 – 1.48 (m, 7H), 1.48 – 1.19 (m, 39H), 0.88 (t, *J* = 6.8, 6H). ^{13}C NMR (101 MHz, $cdcl_3$) δ 130.40, 130.38, 128.17, 111.50, 77.55, 77.23, 76.91, 74.33, 73.21, 66.44, 46.17, 38.75, 36.87, 34.64, 32.36, 31.75, 30.25, 29.92, 29.90, 29.77, 29.75, 29.72, 29.58, 29.54, 27.48, 27.47, 27.43, 26.70, 25.86, 25.12, 24.72, 24.54, 22.80, 14.29. Calc. mass for the $C_{46}H_{83}NO_2$: 682.1, found 682.5.

Synthesis of (1s,3R,4S)-N,N-dimethyl-3,4-bis((9Z,12Z)-octadeca-9,12-dien-1-yloxy) cyclopentan amine 27

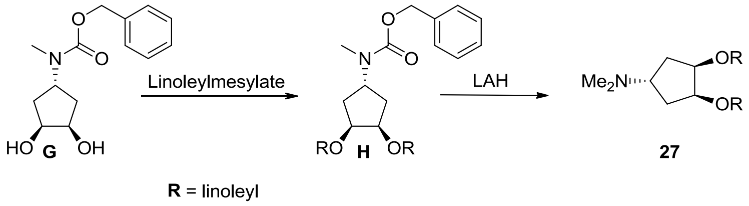

Synthesis of benzyl ((1s,3R,4S)-3,4-bis((9Z,12Z)-octadeca-9,12-dien-1-yloxy)cyclopentyl) (methyl)carbamate H:

To a solution of **G** (0.2 g, 0.000754 mol) in 10 mL of anhydrous benzene in a 25 mL two neck RBF was added (60 % oil suspension) NaH (0.22 g, 0.00453 mol) at once and reaction mixture was heated at 50 ^{o}C for 1.5 h under nitrogen atmosphere to ensure the formation of dianion. Reaction mixture was then cooled to room temperature and a solution of linoleyl mesylate (0.65 g, 0.00188 mol) in 10 ml anhydrous benzene was added slowly into it. Resulting mixture was heated to reflux for 20 h. Reaction was monitored by TLC. Upon completion of the reaction, mixture was cooled to room temperature and then poured onto crushed ice (30g). Aqueous phase was then extracted well with ethyl acetate (3 x 20ml) and

separated out. Organic phase was dried and concentrated on rota vapor to give crude liquid which was purified by flash silica gel column chromatography using 3% ethyl acetate in n-hexane as eluting system to get the title compound as pale yellow liquid. **Yield**: 0.33g (57%). ^{1}H NMR (400MHz, $CDCl_3$) δ 7.34-7.26 (m, 5H), 5.40-5.28 (m, 8H), 5.10 (s, 2H), 4.71 (m, 1H), 3.85 (m, 2H), 3.46-3.40 (m, 4H), 2.80 (s,3H), 2.77-2.74 (t, 4H), 2.05-2.00 (m,9H), 1.73 (m, 2H), 1.55 (m, 3H), 1.37-1.24 (m, aliphatic protons), 0.89-0.85 (m, 6H). **HPLC** 92.60%.

Synthesis of (1s,3R,4S)-N,N-dimethyl-3,4-bis((9Z,12Z)-octadeca-9,12-dien-1-yloxy) cyclopentan amine 27

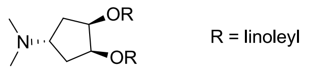

27

Prepared the compound **27** by following the general procedure using *N*-Cbz-compound **H** of the corresponding product (0.32 g, 0.42 mmol, 1.0 eq) and 1M solution of LAH in THF (0.63 mL, 0.63 mmol, 1.5 eq), gave 0.16 g (60%) of the pure product **27.** ^{1}H NMR (400 MHz, $CDCl_3$) δ 5.49 – 5.18 (m, 8H), 3.85 (t, *J* = 3.9, 2H), 3.53 – 3.31 (m, 4H), 2.76 (t, *J* = 6.4, 4H), 2.20 (s, 6H), 2.11 – 1.88 (m, 10H), 1.74 – 1.62 (m, 2H), 1.62 – 1.50 (m, 4H), 1.43 – 1.14 (m, 33H), 0.95 – 0.76 (m, 6H). ^{13}C NMR (101 MHz, $cdcl_3$) δ 130.40, 130.34, 128.17, 128.14, 80.06, 77.54, 77.22, 76.91, 69.91, 63.22, 43.85, 33.90, 31.74, 30.19, 29.99, 29.91, 29.77, 29.71, 29.57, 29.52, 27.46, 27.42, 26.42, 25.85, 22.79, 14.28. Calc. mass for the $C_{43}H_{79}NO_2$: 642.0, found 642.5.

Synthesis of DLin-diol

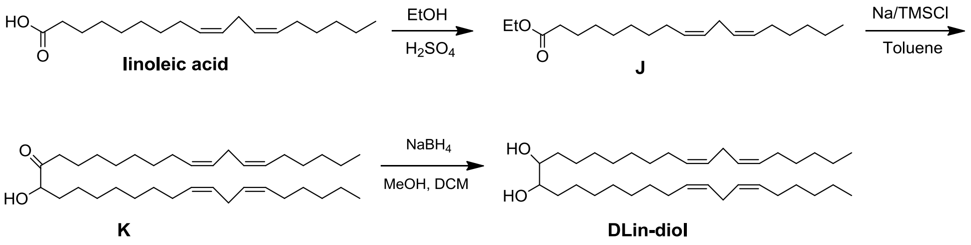

Synthesis of (9Z,12Z)-ethyl octadeca-9,12-dienoate J

To 500ml of ethanol cooled below 0°C using ice-salt mixture was added 10ml of Conc.H_2SO_4 slowly. Linoleic acid (100 g, 357 mmol) in 500ml of ethanol was added to the above solution slowly by maintaining the temperature below 0°C. After addition the reaction mass was warmed to RT and then refluxed for 5hrs (TLC). It was then cooled to room temperature and neutralized by sat. $NaHCO_3$ solution. The resulting solution was

concentrated to remove excess of solvent. The residue was diluted with water (1000 mL) and extracted with DCM (6x500 mL). The combined organic layer was washed with brine (1000 mL), and dried over sodium sulfate, filtered and evaporated at reduced pressure to obtain pure product (109.80 g, 99%) as a pale yellow liquid , which was taken as such for the next stage. ^{1}H NMR ($CDCl_3$) δ 0.89 (t, 3H, J = 6.8Hz), 1.24-1.31 (m, 17H), 1.62 (m, 2H, J = 10Hz), 2.04 (q, 4H, J_1 = 6.8Hz, J_2 = 6.8Hz), 2.29 (t, 2H, J = 7.6Hz), 2.76 (t, 2H, J = 6.4Hz), 4.13 (q, 2H, J_1 = 7.2Hz, J_2 = 7.2Hz), 5.34(m, 4H). ^{13}C NMR ($CDCl_3$) δ 13.9, 14.1, 22.5, 24.9, 25.5, 27.1, 29.0, 29.1, 29.3, 29.5, 31.4, 34.2, 60.0, 127.8, 127.9, 129.9, 130.0, 173.6.

Synthesis of (6Z,9Z,27Z,30Z)-19-hydroxyhexatriaconta-6,9,27,30-tetraen-18-one K

To 660ml of freshly distilled toluene in a 2L multineck RB flask fitted with reflux condenser under argon was added sodium pieces (41.1 g, 1.785 mol). To this was added TMSCl (192 mL, 1.499 mol) slowly and heated to 40°C after addition. Then a solution of **J**(110 g, 0.357 mol) in 275 mL of freshly distilled toluene was added slowly by maintaining the reaction temperature at 40°C over a period of 1hr. It was then refluxed for 2-3hrs. After 3hrs the reaction mass turned pale purple in color (TLC). Heating was stopped and the reaction mass was cooled to room temperature, filtered through a pad of celite and washed with toluene. The filtrate obtained was stirred with 3L of sat. NH_4Cl solution for 15 – 20 minutes until the silyl ether converted to the required α-keto alcohol. The organic layer was separated and the aqueous layer was washed with ethyl acetate (3x1000ml). The combined organic layer was washed with brine (1L), dried over sodium sulfate, filtered and evaporated at reduced pressure to obtain the crude material, which was purified by silica gel chromatography using hexane/ethyl acetate as eluent. The product got eluted at 3% ethyl acetate in hexane to get 2002 (44 g, 47%) as a pale yellow liquid. ^{1}H NMR (400MHz, $CDCl_3$) δ 0.89 (t, 6H, J = 7.2Hz), 1.2-1.3 (m, 30H), 1.53 (m, 1H), 1.62 (m,2H), 1.8 (m,1H), 2.04 (q, 8H, J_1=6.8Hz, J_2=6.8Hz), 2.43 (m, 2H), 2.76 (t, 4H, J = 6.4Hz), 3.49 (d, 1H, J=4.8Hz) 4.16 (m, 1H), 5.34 (m, 8H).^{13}C NMR (100MHz, $CDCl_3$) δ 14.1, 22.6, 23.6, 24.8, 25.6, 27.2, 29.1, 29.2, 29.24, 29.3, 29.4, 29.56, 29.6, 31.5, 33.7, 37.8, 76.3, 127.8, 127.9, 129.9, 130.0, 212.4.

Synthesis of (6Z,9Z,27Z,30Z)-hexatriaconta-6,9,27,30-tetraene-18,19-diol DLin-diol

A solution of 2002 (44 g, 83 mol) in methanol/DCM mixture (490 mL, DCM was added to make the solution homogeneous) under argon was cooled below 0°C using ice-salt mixture. Sodium borohydride (4.7 g, 125 mmol)was added in one lot to the reaction mass. The suspension was stirred for 2hrs, and the mass temperature slowly raised to RT. After 2hrs the reaction mass became homogeneous. TLC showed the absence of starting material. The

reaction was quenched with 100ml of water, and concentrated to remove excess solvent. The residue obtained was again diluted with water (500 mL), and extracted with DCM (4x500 mL). The combined organic layer was washed with brine (500 mL), dried over sodium sulfate, filtered and evaporated at reduced pressure to obtain the crude product, which was purified by silica gel chromatography using hexane/ethyl acetate as eluent. The product eluted from 4% to 20% of ethyl acetate in hexane to get **DLin-diol** (36g, 82%) as a white semisolid. ^{1}H NMR ($CDCl_3$): δ 0.89 (t, 6H, J = 6.8Hz), 1.2-1.5 (m, 36H), 1.78(d, 1H, J = 4Hz), 1.95 (d, 1H, J = 4Hz), 2.04 (q, 8H, J_1 = 6.8 Hz, J_2 = 6.8Hz), 2.77 (t, 4H, J = 6.4Hz), 3.40 (m, 1H), 3.61 (m, 1H), 5.34 (m, 8H). ^{13}C NMR ($CDCl_3$): δ 13.7, 22.2, 25.2, 25.3, 25.7, 26.8, 28.4, 28.9, 29.0, 29.1, 29.3, 30.8, 31.1, 33.2, 74.1, 74.3, 127.5, 127.6, 129.7, 129.8. MS: Molecular weight calculated for $C_{36}H_{66}O_2$ 530.51, Found: 531.52(M+H).

Synthesis of 2-(4,5-di((8Z,11Z)-heptadeca-8,11-dien-1-yl)-2-methyl-1,3-dioxolan-2-yl)-*N,N*-dimethylethanamine 28

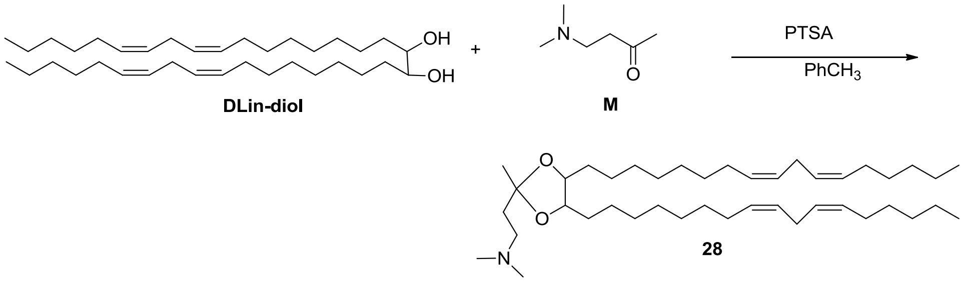

To a solution of **DLin-diol** (2.1 g, 4.0 mmol), ketone **M** (0.50 g, 4.3 mmol) in toluene was added the PTSA (0.86 g, 5 mmol) and refluxed under Dean-Stock apparatus until there is no starting material left. Cooled the reaction mixture, evaporated, directly loaded on column chromatography and purified using 0-10% MeOH in CH_2Cl_2 to get 1.25 g of the pure compound **28** in 50% yields. ^{1}H NMR (400 MHz, $CDCl_3$) δ 5.46 – 5.24 (m, 8H), 4.09 – 3.93 (m, 1H), 3.65 – 3.42 (m, 2H), 2.84 – 2.68 (m, 5H), 2.47 – 2.30 (m, 3H), 2.27 – 2.16 (m, 7H), 2.04 (q, *J* = 6.8, 9H), 1.82 (qd, *J* = 12.4, 7.8, 2H), 1.46 (t, *J* = 19.0, 7H), 1.40 (s, 38H), 0.99 – 0.76 (m, 6H). Calc. mass for the $C_{42}H_{77}NO_2$: 628.07; found 628.5.

Synthesis of 29

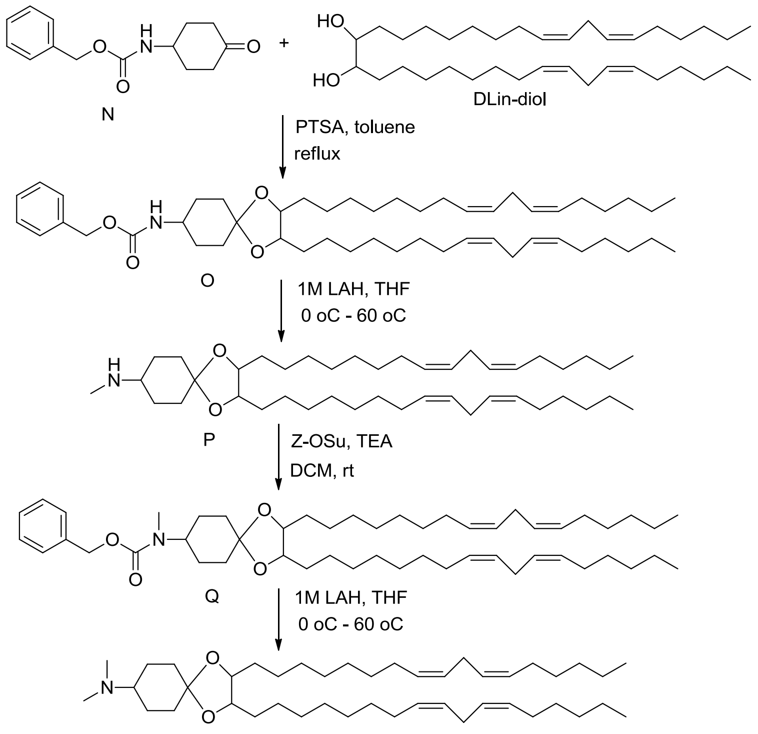

29

Synthesis of benzyl (2,3-di((8Z,11Z)-heptadeca-8,11-dien-1-yl)-1,4-dioxaspiro[4.5]decan-8-yl)carbamate O

Using the general ketalyzation procedure using *N*-Cbz-4-amino cyclohexanone **N** (1.5 g, 6.0 mmol, 1.0 eq), dilinoleyl diol **DLin-diol** (3.38 g, 6.0 mmol, 1.0 eq) and PTSA (0.11 g, 0.6 mmol, 0.1 eq), which gave 3.31 g (69%) of the pure corresponding ketal **O.** ^{1}H NMR (400 MHz, $CDCl_3$) δ 7.45 – 7.24 (m, 5H), 5.47 – 5.24 (m, 8H), 5.06 (d, *J* = 10.8, 1H), 4.63 (d, *J* = 7.4, 1H), 3.97 (s, 1H), 3.55 (d, *J* = 3.7, 2H), 2.76 (t, *J* = 6.4, 4H), 2.04 (q, *J* = 6.7, 8H), 1.91 (s, 2H), 1.81 – 1.59 (m, 3H), 1.56 – 1.42 (m, 8H), 1.42 – 1.15 (m, 31H), 0.88 (t, *J* = 6.8, 6H). Calc. mass for the $C_{50}H_{81}NO_4Na$: 759.62; found 786 (+Na).

Synthesis of 2,3-di((8Z,11Z)-heptadeca-8,11-dien-1-yl)-N-methyl-1,4-dioxaspiro[4.5]decan-8-amine P

Using the general experimental procedure described earlier, using *N*-Cbz ketal **O** (2.42 g, 3.0 mmol, 1.0 eq), 1M LAH in THF (4.6 mL, 4.6 mmol, 1.5 eq), this gave 2.17 g of the compound **P** in quantitative yields. ^{1}H NMR (400 MHz, $CDCl_3$) δ 5.49 – 5.12 (m, 8H), 4.69 (s, 1H), 3.98 (d, *J* = 5.9, 1H), 3.56 (d, *J* = 3.7, 1H), 2.76 (t, *J* = 6.2, 4H), 2.37 (s, 3H), 2.13 – 1.93 (m, 8H), 1.92 – 1.63 (m, 5H), 1.60 – 1.40 (m, 8H), 1.40 – 1.14 (m, 34H), 0.88 (t, *J* = 6.8, 6H). Calc. mass for the $C_{43}H_{77}NO_2$: 639.6; found 640.3.

Synthesis of benzyl (2,3-di((8Z,11Z)-heptadeca-8,11-dien-1-yl)-1,4-dioxaspiro[4.5]decan-8-yl)(methyl)carbamate Q

To a stirred solution of *N*-methyl ketal **O** (2.0 g, 3.0 mmol, 1.0 eq), triethyl amine (1.3 mL, 9.0 mmol, 3.0 eq) in dichloromethane at 0 ^{o}C was added solid Z-OSu portions wise and continued the reaction at room temperature under argon for overnight. After completion of the reaction, diluted with dichloromethane, washed with water, brine, dried on $MgSO_4$, concentrated and purified by column chromatography using hexane : ethylacetate (20%) as gradients to get 1.94 g (80%) of the pure Cbz-protected ketal **Q**. ^{1}H NMR (400 MHz, $CDCl_3$) δ 7.50 – 7.16 (m, 5H), 5.49 – 5.24 (m, 8H), 5.15 (d, *J* = 15.4, 2H), 4.04 (dd, *J* = 59.3, 9.4, 1H), 3.72 – 3.43 (m, 1H), 2.77 (dd, *J* = 13.2, 6.5, 8H), 2.04 (q, *J* = 6.7, 8H), 1.93 – 1.68 (m, 4H), 1.66 – 1.55 (m, 3H), 1.48 (d, *J* = 5.1, 6H), 1.38 – 1.17 (m, 30H), 0.88 (t, *J* = 6.7, 6H). Calc. mass for the $C_{51}H_{83}NO_4$: 773.6; found 774.3.

Preparation of 2,3-di((8Z,11Z)-heptadeca-8,11-dien-1-yl)-N,N-dimethyl-1,4-dioxaspiro[4.5] decan-8-amine 29.

Following the general procedure as described earlier, using *N*-Cbz-ketal **Q** (1.48 g, 1.85 mmol, 1.0 eq), 1M LAH in THF (2.77 mL, 2.77 mmol, 1.5 eq), this gave 1.0 g of the compound **29** in 79% yield. ^{1}H NMR (400 MHz, $CDCl_3$) δ 5.48 – 5.13 (m, 8H), 3.97 (d, *J* = 5.8, 1H), 3.55 (d, *J* = 4.2, 1H), 2.76 (t, *J* = 6.3, 4H), 2.27 (s, 6H), 2.23 – 2.12 (m, 1H), 2.04 (q, *J* = 6.7, 8H), 1.80 (dt, *J* = 16.5, 10.8, 3H), 1.65 (t, *J* = 10.2, 1H), 1.60 – 1.42 (m, 8H), 1.42 – 1.16 (m, 32H), 0.88 (t, *J* = 6.8, 6H). ^{13}C NMR (101 MHz, $CDCl_3$) δ 130.41, 130.32, 128.20, 128.13, 107.86, 107.39, 81.06, 80.95, 78.03, 77.98, 77.54, 77.22, 76.90, 62.74, 62.69, 42.07, 42.02, 37.20, 35.80, 35.18, 34.08, 33.59, 33.43, 31.74, 30.12, 29.98, 29.91, 29.87, 29.78, 29.69, 29.64, 29.57, 29.50, 29.47, 27.45, 27.42, 26.55, 26.43, 26.41, 26.37, 26.13, 25.85, 25.80, 22.79, 14.29. Calc. mass for the $C_{44}H_{79}NO_2$: 653.6; found 654.3.

Synthesis of 4-Bromo-butyric acid-1-octadeca-9, 12-dienyl-nondeca-10, 13-dienyl ester DLin-MC3-Br

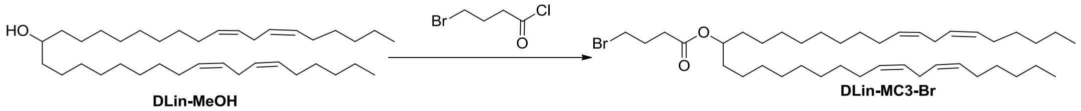

To a solution of 4-bromo butyric acid (22.14g, 0.1325 mol) in anhydrous DCM (300 mL) was added slowly and carefully oxalyl chloride (23 mL, 0.2651 mol) at 0° C under inert atmosphere followed by catalytic amount of dry DMF (0.05 mL). Resulting solution was then allowed to warm to room temperature over the period of time and stirred for 3.0 h. The pale

yellow solution was then concentrated under vacuum to get the crude acid chloride **2** which was dried well under high vacuum. Crude acid chloride **2** was dissolved in anhydrous DCM (300 mL) and cooled to 0° C. To this stirring solution was slowly added 200ml of dilinoleyl alcohol **1** (35g, 0.06628 mol) in DCM. The reaction mixture was warmed to room temperature, stirred for 20 h and monitored by TLC (silica gel, 10% ethyl acetate in hexanes). Upon completion of the reaction, mixture was diluted with water (400 mL) and organic layer was separated out. Organic phase was then washed with sat. solution of $NaHCO_3$ (1 x 400 mL) followed by brine (1 x 100 mL) and concentrated under vacuum. Crude product was then purified by silica gel (100-200 mesh) column, eluted with 2-3% ethyl acetate in hexane solution to give 38 g (84.5%) of desired product as pale yellow liquid. ^{1}H NMR (400MHz, $CDCl_3$): δ 5.37-5.28 (m, 8H), 4.88-4.83 (m, 1H), 3.46-3.43 (t, 2H, J= 6.44Hz), 2.76-2.74 (m, 4H), 2.49-2.45(m, 2H), 2.19-2.12(m, 2H), 2.06-2.01 (m, 8H), 1.51-1.49 (m, 4H), 1.37-1.28 (m, aliphatic protons), 0.89-0.86 (m,6H). Purity by HPLC(ELSD)-100%

Synthesis of (6Z,9Z,28Z,31Z)-heptatriaconta-6,9,28,31-tetraen-19-yl 4-(diethylamino) butanoate 30

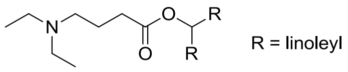

30

A solution of **(DLin-MC3-Br)** (0.65 g) in dichloromethane (20 mL) was treated with diethylamine (5 mL) at room temperature for three days. The solvent was removed on a rotovap. The residue was partitioned between dichloromethane and dilute hydrochloric acid. The organic fractions were washed with dilute aqueous sodium bicarbonate solution, dried over anhydrous magnesium sulphate, filtered and the solvent removed. The residue was passed down a silica gel column (20 g) using a 0-2% methanol/dichloromethane elution gradient. Fractions containing the purified product were combined and the solvent removed, yielding **30** as a colorless oil (0.58 g). ^{1}H NMR (400MHz, $CDCl_3$) δ: 5.32 (m; 8H); 4.83 (p; J=6.1Hz; 1H); 2.92 (m; 4H); 2.85 (m; 2H); 2.74 (t; J=6.5Hz; 4H); 2.37 (J=6.6Hz; 2H); 2.02 (m; 10H); 0.87 (t; J=6.7Hz; 6H)

Synthesis of (6Z,9Z,28Z,31Z)-heptatriaconta-6,9,28,31-tetraen-19-yl 4-[bis(propan-2-yl)amino]butanoate 31.

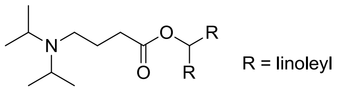

31

A solution of **(DLin-MC3-Br)** (0.51 g) in dichloromethane (20 mL) was treated with diisopropylamine (5 mL) at room temperature for a week. The solvent was removed on a

rotovap and the residue partitioned between dichloromethane and dilute hydrochloric acid. The organic fractions were washed with dilute aqueous sodium bicarbonate solution, dried over anhydrous magnesium sulphate, filtered and the solvent removed. The residue was passed down a silica gel column (20 g) using a 0-5% methanol/dichloromethane elution gradient. Fractions containing the purified product were combined and the solvent removed, yielding **31** as a colorless oil (0.24 g). ^{1}H NMR (400MHz, $CDCl_3$) δ: 5.33 (m; 8H); 4.84 (p; J=6.1Hz; 1H); 2.97 (m; J=6.5Hz; 2H); 2.75 (t; J=6.4Hz; 4H); 2.40 (t; J=7.2Hz; 2H); 2.28 (t; J=7.2Hz; 2H); 2.03 (m; 8H); 1.67 (p; J=7.2Hz; 2H); 0.96 (d; J=6.7Hz; 12H); 0.87 (t; J=6.7Hz; 6H).

Synthesis of dilinoleyl azide

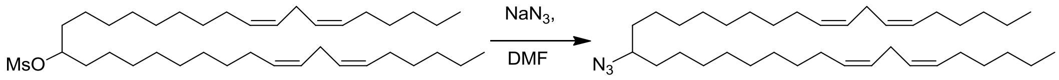

DLin-M-Ms DLin-M-N3

To a solution of the mesylate (50 g, 0.08 mol) in DMF (500 mL) under argon atmosphere, was added NaN_3 (27 g, 0.41 mol) and heated to 70°C and maintained the temperature for four hours (TLC). The mixture was diluted with water and extracted with ethyl acetate (3x250 ml). The organic layer was washed with water, brine, dried over $Na_2 SO_4$ and evaporated at reduced pressure to give crude product, which was purified by silica gel chromatography using hexane / ether as eluent. The product was eluted at 2% ether hexane to yield 36 g (86%) pure azide. ^{1}H NMR (400 MHz, $CDCl_3$): **δ** 0.90 (t, 8H), 1.30 (m, 36H), 1.49 (t, 4H, J = 6.4 Hz) 2.04 (q, 8H, J1 =7.6, J2 = 14Hz), 2.77 (t, 4H, J = 6.4 Hz), 3.22 (m, 1H), 5.34 (m, 8H). ^{13}C NMR (400 MHz, $CDCl_3$): **δ** 14.1, 22.5, 25.6, 26.1, 27.2, 29.2, 29.3, 29.45, 29.65, 31.5, 34.1, 63.1, 127.9, and 130.1.

Synthesis of DLin-methylamine

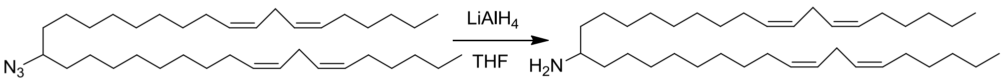

DLin-MN$_3$ DLin-MA

Lithium aluminiumhydride (2.75 g, 0.072 mol) was suspended in dry THF (200 mL) at room temperature under argon atmosphere. It was then cooled to 0°C and to this was added the azide in THF solution drop wise. After addition, the reaction temperature was allowed to warm to room temperature and stirred for 20 hrs (TLC). It was then cooled to 0°C and quenched with saturated solution of sodium sulfate. It was filtered through celite bed and washed with ethyl acetate. The combined filtrate was dried over Na_2SO_4 and evaporated at

reduced pressure to get pure product. Yield: 18g (74%). ^{1}H NMR (400 MHz, $CDCl_3$): δ 0.89 (t, 6H), 1.2-1.5 (m, 40H), 2.05 (q, 8H, J = 6.8Hz) 2.66 (m, 1H), 2.77 (t, 4H, J = 6.4Hz), 5.35 (m, 8H). ^{13}C NMR (400 MHz, CDCl3): δ 13.9, 22.5, 25.5, 26.1, 27.1, 29.2, 29.3, 29.4, 29.6, 29.8, 30.2, 30.3, 31.4, 38.1, 51.1, 127.9, and 130.0.

Synthesis of *N*-(4-N,N-dimethylamino)butanoyl-(6Z,9Z,28Z,31Z)-heptatriaconta-6,9,28,31-tetraen-19-amine 32.

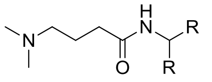

R = linoleyl

32

A solution of the crude (6Z,9Z,28Z,31Z)-heptatriaconta-6,9,28,31-tetraen-19-amine (0.51 g), 4-dimethylaminobutanoic acid (0.56 g), 4-N,N-dimethylaminopyridine (0.67 g) and 1-ethyl-3-(3-dimethylaminopropyl)carbodiimide hydrochloride (0.55 g) in dichloromethane (20 mL) was stirred at room temperature overnight. The solution was washed with dilute hydrochloric acid followed by dilute aqueous sodium bicarbonate. The organic fractions were dried over anhydrous magnesium sulphate, filtered and the solvent removed on a rotovap. The residue was passed down a silica gel column (20 g) using a 0-6% methanol/dichloromethane elution gradient. Fractions containing the purified product were combined and the solvent removed, yielding **32** as a colorless oil (0.55 g). ^{1}H NMR (400MHz, $CDCl_3$) δ 6.06 (d; J=9.1Hz; 1H); 5.33 (m; 8H); 3.85 (m; 1H); 2.75 (t; J=6.4Hz; 4H); 2.31 (t; J=6.7Hz; 2H); 2.23 (t; J=6.7Hz; 2H); 2.21 (s; 6H); 2.03 (m; 8H); 1.77 (p; J=6.7Hz; 2H); 0.87 (t; J=6.7Hz; 6H)

Synthesis of (2-{2,2-bis[(9Z,12Z)-Octadeca-9,12-dien-1-yl]-1,3-dioxan-5-yl}ethyl) dimethylamine 33.

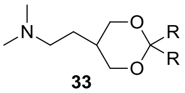

R = linoleyl

33

This compound was synthesized as pale oil in a manner similar to that described for **2**, but 2-hydroxymethyl-1,3-propanediol was replaced with 2-hydroxyethyl-1,3-propanediol. ^{1}H NMR (400 MHz, $CDCl_3$) δ 5.25-5.45 (8, m, 4x CH=CH), 3.87 (2H, dd, 2 x OCH)), 3.55 (2H, dd OCH), 2.75 (4H, t, 2 x C=C-CH_2-C=C), 2.45-2.60 (2H, br, NCH_2), 2.40 (6H, s, 2 x NCH_3), 2.03 (8H, q, 4 x allylic CH_2), 1.73-1.86 (1H, m), 1.56-1.72 (6H, m, 2 x CH_2), 1.22-1.45 (32H, m), 0.90 (6H, t, 2 x CH_3)

Synthesis of (4-{2,2-bis[(9Z,12Z)-Octadeca-9,12-dien-1-yl]-1,3-dioxan-5-yl}butyl) dimethylamine. **34.**

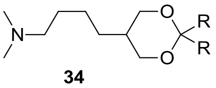

R = linoleyl

34

This compound was synthesized as pale oil in a manner similar to that described for **2**, but 2-hydroxymethyl-1,3-propanediol was replaced with 2-hydroxybutyl-1,3-propanediol. ^{1}H NMR (400 MHz, $CDCl_3$) δ: 5.24-5.45 (8H, m, 4x CH=CH), 3.79 (2H, dd, 2 x OCH)), 3.50 (2H, dd OCH), 2.76 (4H, t, 2 x C=C-CH_2-C=C), 2.37 (2H, t, NCH_2), 2.31 (6H, s, 2 x NCH_3), 2.04 (8H, q, 4 x allylic CH_2), 1.63-1.90 (3H, m,), 1.45-1.62 (4H, m, 2 x CH_2), 1.22-1.45 (36H, m), 0.90 (6H, t, 2 x CH_3).

Synthesis of (6Z,9Z,28Z,31Z)-heptatriaconta-6,9,28,31-tetraen-19-yl (2-(dimethylamino) ethyl)carbamate 35

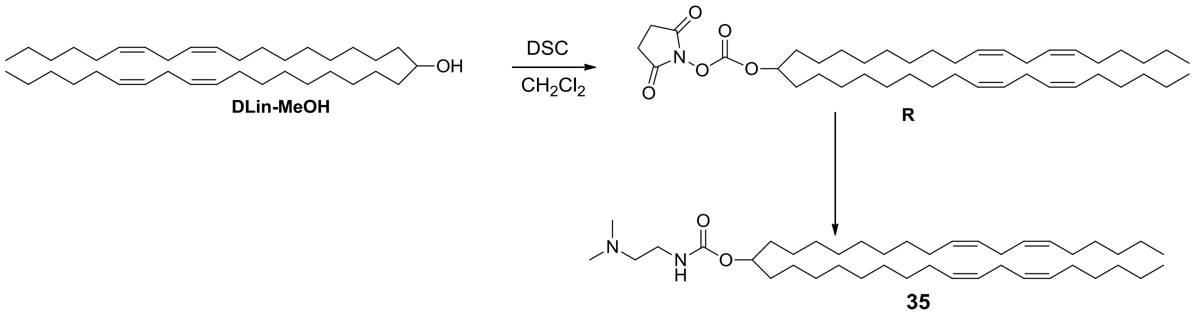

The treatment of the NHS ester of the **DLin-MeOH** (1.34 g, 3 mmol) with the dimethylethylenediamine (0.5 mL) provided the carbamate **35** (1.08 g, 90%). ^{1}H NMR (400 MHz, $CDCl_3$) δ 5.51 – 5.25 (m, 8H), 5.07 (s, 1H), 4.85 – 4.65 (m, 1H), 3.24 (d, J = 5.1, 2H), 2.77 (t, J = 6.4, 4H), 2.39 (t, J = 5.9, 2H), 2.22 (s, 6H), 2.04 (t, J = 6.4, 8H), 1.48 (s, 4H), 1.33 (ddd, J = 21.1, 13.7, 8.6, 37H), 0.89 (t, J = 6.8, 6H). Molecular weight for $C_{42}H_{79}N_2O_2$ $(M+H)^+$ Calc. 643.61, Found 643.5.

Synthesis of 2-(dimethylamino)ethyl (6Z,9Z,28Z,31Z)-heptatriaconta-6,9,28,31-tetraen-19-ylcarbamate 36

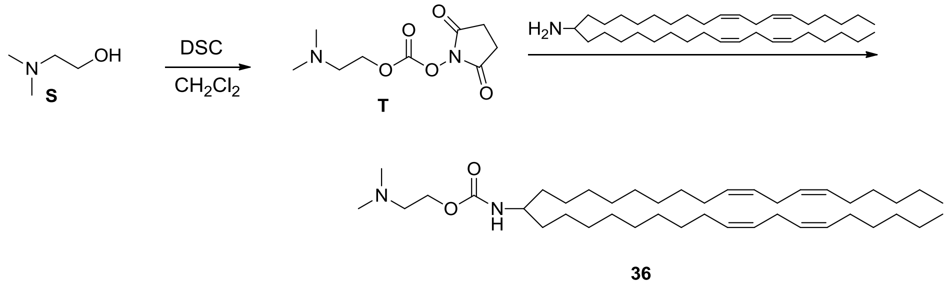

To a solution of *N,N'*-disuccinimidyl carbonate (5.50 g, 21.5 mmol) in CH_2Cl_2 (200 mL), 2-dimethylaminoethanol **S** (2.37 mL, 23.6 mmol) was added dropwise. The resulting mixture was stirred at room temperature overnight. To this solution, Et_3N (0.822 mL, 5.90 mmol) and dilinoleylamine (2.07 g, 3.92 mmol) were added and the reaction mixture was stirred at room temperature overnight. The reaction mixture was diluted with CH_2Cl_2 and washed with saturated $NaHCO_3$ aq. The organic layer was dried over anhydrous $MgSO_4$, filtered and

concentrated. The crude was purified by silica gel column chromatography (0-5% MeOH in CH_2Cl_2) to give compound **36** (1.78 g, 2.77 mmol, 71%, 2 steps, R_f = 0.26 developed with 5% MeOH in CH_2Cl_2). ^{1}H NMR (400 MHz, $CDCl_3$) δ 5.29-5.41 (m, 8 H), 4.50 (d, *J* = 9.2 Hz, 1 H), 4.13 (t, *J* = 5.4 Hz, 2 H), 3.56 (s, 1 H), 2.76 (t, *J* = 6.4 Hz, 4 H), 2.53 (t, *J* = 5.4 Hz, 2 H), 2.27 (s, 6 H), 2.04 (q, *J* = 6.8 Hz, 8 H), 1.26-1.44 (m, 40 H), 0.88 (t, *J* = 6.8 Hz, 6 H). ^{13}C NMR (100 MHz, $CDCl_3$) δ 156.44, 130.38, 130.35, 128.16, 128.15, 62.19, 58.58, 51.50, 45.81, 35.64, 31.73, 30.66, 29.89, 29.82, 29.77, 29.72, 29.56, 29.52, 28.86, 27.45, 27.41, 26.00, 25.84, 22.78, 14.40, 14.15. Molecular weight for $C_{42}H_{79}N_2O_2$ $(M+H)^+$ Calc. 643.61, Found 643.5.

Synthesis of (6Z,9Z,28Z,31Z)-heptatriaconta-6,9,28,31-tetraen-19-yl 3-(ethylamino) propanoate 37

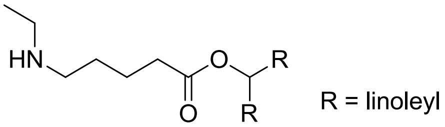

37

The bromide **DLin-M-C3-Br** (1.54 g) was treated with a 15% solution of ethylamine in tetrahydrofuran (70 mL) overnight. The solvent was removed on a rotary evaporator and the residue dissolved in dichloromethane. The solution was washed first with dilute hydrochloric acid, and then with aqueous sodium bicarbonate solution. The organic phase was dried over anhydrous magnesium sulphate, filtered and the solvent removed. The residue was passed down a silica gel column (20 g) using a 0-12% methanol/dichloromethane elution gradient. Fractions containing the purified product were combined and the solvent removed, yielding **37** as a colorless oil (1.25 g). ^{1}H NMR (400MHz, $CDCl_3$) δ 5.33 (m; 8H); 4.84 (p; J=6.1Hz; 1H); 2.75 (t; J=6.4Hz; 4H); 2.65 (m; 4H); 2.33 (t; J=7.4Hz; 2H); 2.03 (m; 8H); 1.81 (p; J=7.3Hz; 2H); 1.09 (t; J=7.1Hz; 3H); 0.87 (t; J=6.8Hz; 6H).

Synthesis of (6Z,9Z,28Z,31Z)-heptatriaconta-6,9,28,31-tetraen-19-yl 4-(propan-2-ylamino) butanoate 38

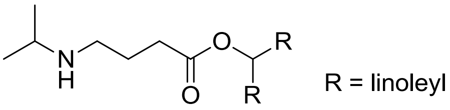

38

A solution of **DLin-M-C3-Br** (0.52 g) in dichloromethane (20 mL) was treated with isopropylamine (5 mL) at room temperature for three days. The solvent was removed on a rotovap and the residues partitioned between dichloromethane and dilute hydrochloric acid. The organic fractions were washed with dilute aqueous sodium bicarbonate solution, dried

over anhydrous magnesium sulphate, filtered and the solvent removed. The residue was passed down a silica gel column (20 g) using a 0-8% methanol/dichloromethane elution gradient. Fractions containing the purified product were combined and the solvent removed, yielding **38** as a colorless oil (0.36 g). ^{1}H NMR (400MHz, $CDCl_3$) δ 5.32 (m; 8H); 4.84 (p; J=6.2Hz; 1H); 2.75 (m; 5H); 2.60 (t; J=7.3Hz; 2H); 2.32 (t; J=7.3Hz; 2H); 2.03 (m; 8H); 1.78 (p; J=7.2Hz; 2H); 1.03 (d; J=6.3Hz; 3H); 0.87 (t; J=6.8Hz; 6H)

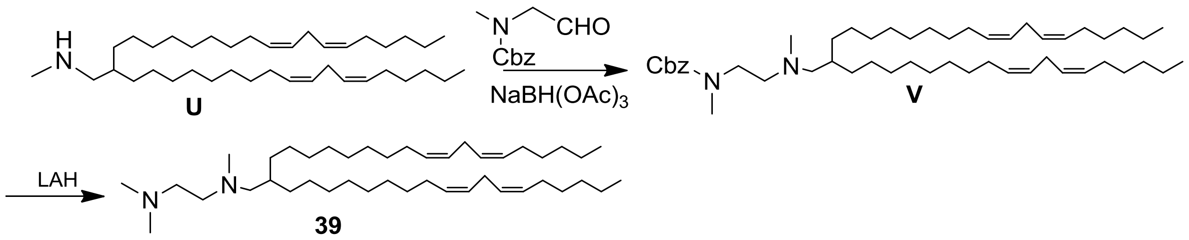

Synthesis of benzyl methyl(2-(methyl((11Z,14Z)-2-((9Z,12Z)-octadeca-9,12-dien-1-yl)icosa-11,14-dien-1-yl)amino)ethyl)carbamate V: To a solution of **U** (5 g, 0.0089 mol) in 100 mL of DCM under argon at 0^oC was added $NaBH(OAc)_3$ (2.30 g, 0.0106 mol) and stirred for 20 minutes. Aldehyde (1.70 g, 0.0082 mol) in 700ml of DCM was added slowly to the reaction mass over a period of 45 minutes. After the completion of addition the reaction mass was allowed to stir at RT for 15 – 20 minutes. The reaction mass was washed with sat. $NaHCO_3$ (2x500 mL) and water (500 mL). The aqueous layer was re-extracted with DCM (500 mL). The combined organic layer was washed with brine (500 mL). The organic layer was dried over Na_2SO_4, filtered and concentrated. The crude obtained was purified by silica gel chromatography and Hexane/ Diethyl ether as eluent. The product got eluted at 8% of ether in hexane as brown liquid (yield, 6.40g, 96%). ^{1}H NMR: (400MHz, $CDCl_3$): δ 0.89 (t, 6H, J= 7.2Hz), 1.26 – 1.43 (m, 40H), 1.85 (m, 1H), 2.06 (q, 8H, J1=6.8Hz, J2=6.8Hz), 2.15 (s, 2H), 2.20 (s, 3H), 2.45 (m, 2H), 2.77 (t, 4H, J=6Hz), 2.95 (s, 3H), 3.35 (m,2H), 5.12 (s, 2H), 5.32 (m, 8H), 7.35 (m,5H).

Synthesis of N1,N1,N2-trimethyl-N2-((11Z,14Z)-2-((9Z,12Z)-octadeca-9,12-dien-1-yl)icosa-11,14-dien-1-yl)ethane-1,2-diamine 39: To a suspension of lithium aluminiumhydride (0.751 g, 0.0198 mol) in THF under argon atmosphere at 0^0C, was added a solution of **V** (5.7g, 0.0076 mol) in THF drop-wise. After addition, it was allowed to warm to room temperature and stirred for 15 hours (TLC). The reaction mass was cooled to 0°C and quenched with saturated solution of sodium sulfate (50ml) followed by ethyl acetate (100ml). It was filtered through celite bed and washed with ethyl acetate. Combined filtrates were evaporated at reduced pressure to obtain crude product, which was purified silica gel

chromatography using DCM / Ethylacetate / Chloroform/ Methanol as eluent. The product **39** eluted at 3% chloroform in methanol as brown liquid (3.80g, 80%) ^{1}H NMR: (400MHz, $CDCl_3$) δ 0.89 (t, 6H, J=6.8Hz), 1.26-1.37 (m, 40H), 1.42 (m,1H), 2.06 (q, 8H, J1= 6.8Hz, J2= 6.8Hz), 2.15 (d, 2H, J= 7.2Hz), 2.20 (s, 3H), 2.29 (s, 6H), 2.45 (s, 4H), 2.78 (t, 4H, J=6.4Hz), 5.36 (m, 8H). ^{13}C NMR: (100MHz, $CDCl_3$) δ 14.1, 22.6, 25.6, 26.6, 27.2, 27.22, 28.9, 29.3, 29.6, 29.7, 30.1, 31.5, 32.2, 35.8, 43.2, 45.7, 56.2, 57.2, 63.3, 127.9, 130.2. HPLC ELSD: 100% Mass: 627.53.

Synthesis of 3-(dimethylamino)-N-((11Z,14Z)-2-((9Z,12Z)-octadeca-9,12-dien-1-yl)icosa-11,14-dien-1-yl)propanamide 40

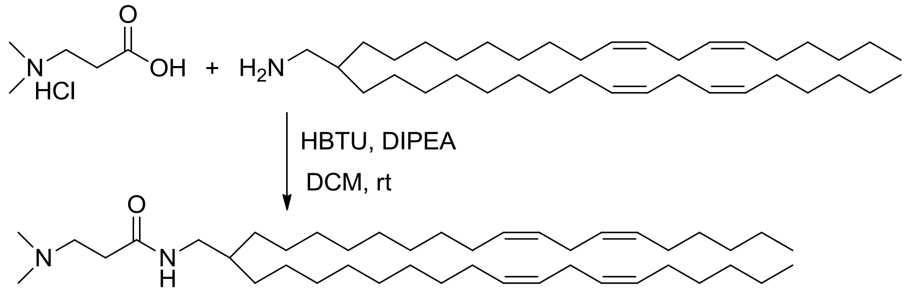

40

To a stirred suspension of *N*, *N*-dimethylamino propionic acid hydrochloride (0.198 g, 1.3 mmol, 1.0 eq) in DCM was added HBTU (0.59 g, 1.56 mmol, 1.2 eq) and DIPEA (0.71 mL, 3.9 mmol, 3.0 eq) at room temperature. After stirred for 10 minutes, a solution of amine (0.7 g. 1.3 mmol, 1.0 eq) in DCM was added drop wise at room temperature and continued the stirring until completion of the reaction. Reaction mixture was diluted with DCM, washed with saturated $NaHCO_3$ solution followed by brine, organic layer was separated and dried over $MgSO_4$, concentrated and purified by the silica gel column chromatography using DCM:MeOH (5%) as gradients to get pure oily compound **40** in 70% yield. ^{1}H NMR (400 MHz, $CDCl_3$) δ 7.18 (brs, 1H), 5.47 – 5.19 (m, 8H), 3.18 – 3.07 (m, 4H), 2.76 (t, *J* = 6.5, 4H), 2.70 (s, 6H), 2.60 (t, *J* = 6.0, 2H), 2.04 (q, *J* = 6.8, 9H), 1.48 (brs, 1H), 1.40 – 1.14 (m, 43H), 0.88 (t, *J* = 6.8, 6H). ^{13}C NMR (101 MHz, $CDCl_3$) δ 172.26, 130.41, 130.36, 128.17, 128.15, 77.54, 77.22, 76.90, 55.70, 43.85, 43.02, 37.90, 31.99, 31.74, 30.25, 29.92, 29.86, 29.81, 29.57, 27.47, 27.42, 26.84, 25.85, 22.79, 14.29. Calc. mass for the $C_{43}H_{80}N_2O$: 640.6, found 641.5.

Synthesis of (6Z,9Z,28Z,31Z)-heptatriaconta-6,9,28,31-tetraen-19-yl 4-(methylamino) butanoate 41

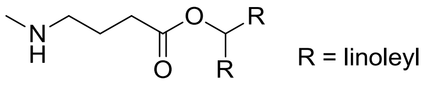

41

The bromide **DLin-M-C3-Br** (0.51 g) was treated with a 10% solution of methylamine in a 3:2 dichloromethane/tetrahydrofuran (50 mL) overnight. The solvent was removed on a rotovap and the residues partitioned between dichloromethane and dilute hydrochloric acid. The organic fractions were washed with dilute aqueous sodium bicarbonate solution, dried over anhydrous magnesium sulphate, filtered and the solvent removed. The residue was passed down a silica gel column (20 g) using a 0-4% methanol/dichloromethane elution gradient. Fractions containing the purified product were combined and the solvent removed, yielding **41** as a colorless oil (0.31 g). ^{1}H NMR (400MHz, $CDCl_3$) δ 5.37 (m; 8H); 4.84 (p; J=6.1Hz; 1H); 2.75 (t; J=6.4Hz; 4H); 2.62 (t; J=7.1Hz; 2H); 2.43 (s; 3H); 2.33 (t; J=7.4Hz; 2H); 2.03 (m; 8H); 1.82 (p; J=7.2Hz; 2H); 0.87 (t; J=6.8Hz; 6H)

1,3-Diethyl 2-[2-(dimethylamino)ethyl]propanedioate. (**001-80).** {Ref. J.Chem. Soc. Perkin Trans I, (7), 1787 (1988)}

To a solution of sodium ethoxide (30 mmol, 12.2 mL from 21 wt% solution in ethanol) in ethanol (15 mL) was added a solution of malonate (4.80 g, 30 mmol) in ethanol (5 mL), followed by addition of 2-(dimethylamino)ethyl chloride hydrochloride (2.16 g, 15 mmol) at room temperature under argon. The resulting mixture was stirred at 50-60 °C under argon for 20 hours. The reaction mixture was allowed to cool to room temperature and water (75 mL) was added. The mixture was extracted with ethyl acetate (3 x 60 mL). The combined organic extract was washed with water (50 mL), brine (50 mL) and dried over sodium sulphate. Concentration gave the crude product which was purified by column chromatography on silica gel (0-6% methanol in dichloromethane). The desired product (**001-80**) was obtained as a yellow oil (1.50 g, 6.5 mmol, 43%).

2-[2-(Dimethylamino)ethyl]propane-1,3-diol. (**001-85).** To a suspension of lithium aluminum hydride (1.75 g, 43 mmol) in tetrahydrofuran (60 mL) was added **001-80** (1.5 g, 6.5 mmol) at 5 °C (ice-water). The resulting mixture was stirred at room temperature overnight. The excess of lithium aluminum hydride was consumed by addition of brine (7 mL) slowly at 5 °C. The solid was filtered off and was washed with 10% methanol in tetrahydrofuran (2 X 15 ml). The filtrate was concentrated. The desired product was obtained as a pale viscous oil (0.74 g, 5.0 mmol, 77%) and used directly for the next step without further purification.

Dimethyl({4-[(9Z,12Z)-octadeca-9,12-dien-1-yloxy]-3-{[(9Z,12Z)-octadeca-9,12-dien-1-yloxy]methyl}butyl})amine 42

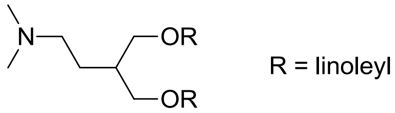

42

To a solution of **001-85** (0.40 g, 2.7 mmol) in benzene (60 mL) was added sodium hydride (1.1 g of 60% sodium hydride in mineral oil, 27.5 mmol) under argon at room temperature. The resulting mixture was stirred at the same temperature for 60min, followed by addition of linoleyl methanesulfonate (2.06 g, 6.0 mmol). The mixture was continued to stir for another 30 min and then was heated to reflux overnight under argon. The reaction was quenched by addition of ethanol (15 mL). The mixture was washed with water (40 mL), brine (50 mL) and dried over sodium sulphate. Concentration gave the crude product which was purified by column chromatography on silica gel (0-6% methanol in dichloromethane). The desired product **42** was obtained as a pale oil (0.59 g, 0.92 mmol, 34%). ^{1}H NMR (400 MHz, $CDCl_3$) δ 5.43-5.30 (m, 8H), 3.42-3.33 (m, 8H), 2.78 (t, J=6.4 Hz, 4H), 2.33 (m, 2H), 2.23 (s, 6H), 2.05 (q, J=6.8 Hz, 8H), 1.87 (m, 1H), 1.59-1.50 (m, 6H), 1.41-1.24 (m, 32H), 0.90 (t, J=6.8 Hz, 6H).

Preparation of 43

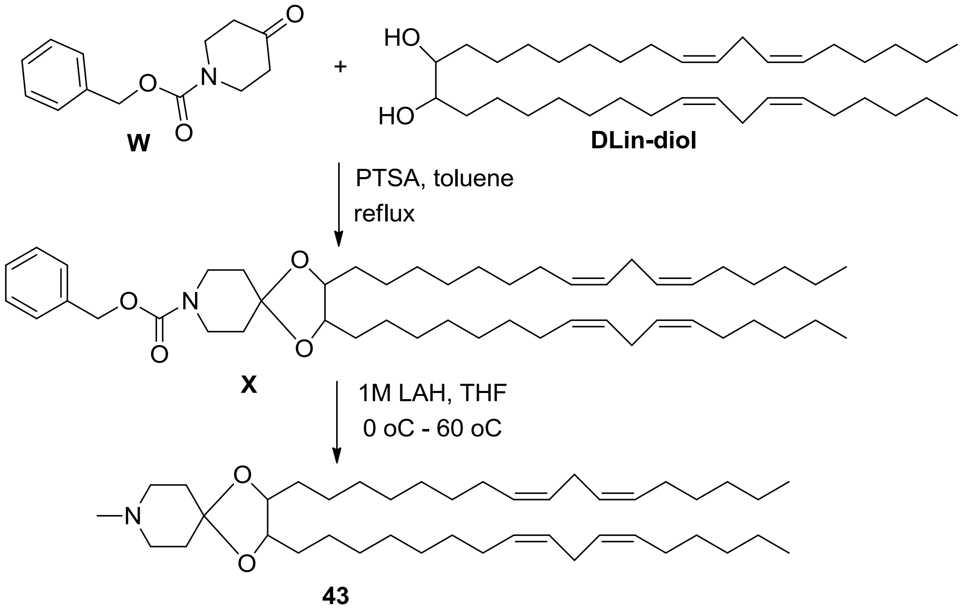

43

Preparation of benzyl 2,3-di((8Z,11Z)-heptadeca-8,11-dien-1-yl)-1,4-dioxa-8-azaspiro[4.5]decane-8-carboxylate : Using our general similar ketalyzation procedure, using 1-*N*-Cbz-piperidone **W** (1.0 g, 4.29 mmol, 1.0 eq), **DLin-diol** (2.4 g, 4.29 mmol, 1.0

eq) and PTSA (0.08 g, 0.43 mmol, 0.1 eq), which gave 2.74 g (71%) of the pure corresponding ketal **X**. ^{1}H NMR (400 MHz, $CDCl_3$) δ 7.42 – 7.21 (m, 5H), 5.46 – 5.22 (m, 8H), 5.11 (s, 2H), 4.01 (d, *J* = 7.1, 1H), 3.68 – 3.43 (m, 5H), 2.81 – 2.66 (m, 4H), 2.04 (q, *J* = 6.7, 8H), 1.76 – 1.40 (m, 9H), 1.40 – 1.15 (m, 32H), 0.88 (t, *J* = 6.8, 6H). Calc. mass for the $C_{49}H_{79}NO_4$: 745.6; found 746.3.

Preparation of 2,3-di((8Z,11Z)-heptadeca-8,11-dien-1-yl)-8-methyl-1,4-dioxa-8-azaspiro[4.5]decane 43

Used similar experimental procedure as described earlier, using *N*-Cbz-ketal **X** (2.72 g, 3.52 mmol, 1.0 eq), 1M LAH in THF (5.27 mL, 5.27 mmol, 1.5 eq), this gave 2.13 g of the compound **43** in 93% yield. ^{1}H NMR (400 MHz, $CDCl_3$) δ 5.52 – 5.20 (m, 8H), 4.09 – 3.92 (m, 1H), 3.61 (s, 1H), 2.79 (t, *J* = 6.5, 4H), 2.49 (s, 4H), 2.31 (s, 3H), 2.07 (q, *J* = 6.8, 8H), 1.75 (dd, *J* = 11.5, 5.8, 4H), 1.52 (m, 5H), 1.43 – 1.19 (m, 31H), 0.91 (t, *J* = 6.9, 6H). ^{13}C NMR (101 MHz, $CDCl_3$) δ 130.22, 130.12, 128.01, 127.92, 105.99, 105.51, 80.73, 77.82, 77.35, 77.23, 77.03, 76.71, 53.80, 53.61, 53.54, 45.98, 38.07, 36.53, 35.24, 33.27, 31.54, 29.74, 29.70, 29.66, 29.46, 29.45, 29.36, 29.27, 27.24, 27.21, 26.24, 26.14, 25.64, 22.59, 14.10. Calc. mass for the $C_{42}H_{75}NO_2$: 625.5; found 626.2.

Synthesis of 3-(dimethylamino)propyl (6Z,9Z,28Z,31Z)-heptatriaconta-6,9,28,31-tetraen-19-ylcarbamate 44

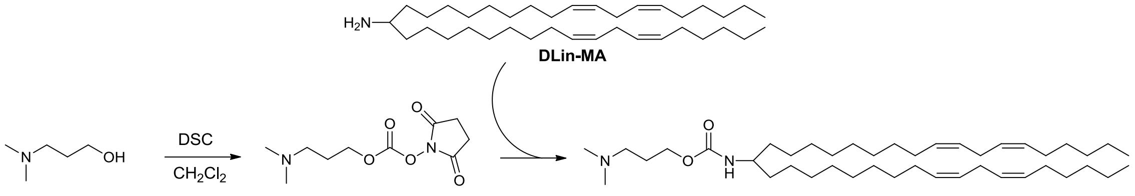

44

To a solution of *N,N'*-disuccinimidyl carbonate (5.50 g, 21.5 mmol) in CH_2Cl_2 (200 mL), 3-dimethylamino-1-propanol (2.43 g, 23.6 mmol) was added dropwise. The resulting mixture was stirred at room temperature overnight. Taken up 50 mL of the solution, Et_3N (0.822 mL, 5.90 mmol) and **DLin-MA** (2.08 g, 3.93 mmol) were added and the reaction mixture was stirred at room temperature overnight. The reaction mixture was diluted with CH_2Cl_2 and washed with saturated $NaHCO_3$ aq. The organic layer was dried over anhydrous $MgSO_4$, filtered and concentrated. The crude was purified by silica gel column chromatography (0-5% MeOH in CH_2Cl_2) to give **44** (1.66 g, 2.53 mmol, 64%, R_f = 0.22 with 5% MeOH in CH_2Cl_2). ^{1}H NMR (400 MHz, $CDCl_3$) δ 5.30-5.41 (m, 8 H), 4.37 (d, *J* = 8.0 Hz, 1 H), 4.09 (t, *J* = 6.0 Hz, 2 H), 3.57 (brs, 1 H), 2.78 (t, *J* = 6.0 Hz, 4 H), 2.33 (t, *J* = 8.0 Hz, 2 H), 2.23 (s, 6 H), 2.02-2.06 (m, 8 H), 1.76-1.80 (m, 2 H), 1.27-1.45 (m, 40 H), 0.89 (t, *J* = 8.0 Hz, 6 H). ^{13}C

NMR (100 MHz, $CDCl_3$) δ 156.53, 130.38, 130.34, 128.16, 128.14, 63.16, 56.56, 51.41, 45.69, 35.71, 31.74, 29.88, 29.83, 29.77, 29.72, 29.56, 29.52, 27.71, 27.45, 27.41, 26.03, 25.84, 22.78, 14.28. Molecular weight for $C_{43}H_{81}N_2O_2$ $(M+H)^+$ Calc. 657.63, Found 657.5.

Synthesis of 2-(dimethylamino)ethyl ((11Z,14Z)-2-((9Z,12Z)-octadeca-9,12-dien-1-yl)icosa-11,14-dien-1-yl)carbamate 45

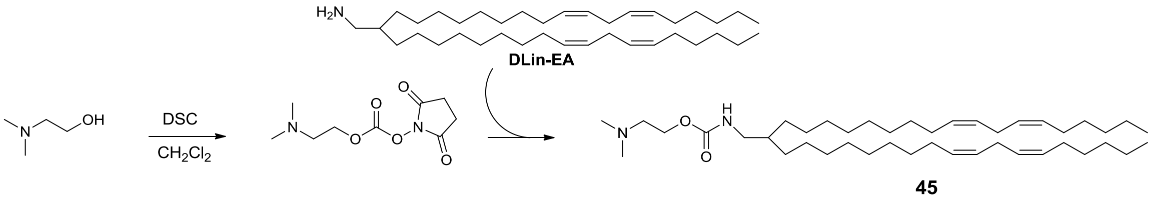

45

To a solution of *N,N'*-disuccinimidyl carbonate (5.50 g, 21.5 mmol) in CH_2Cl_2 (200 mL), 2-dimethylaminoethanol (2.37 mL, 23.6 mmol) was added dropwise. The resulting mixture was stirred at room temperature overnight. Taken up 50 mL of the solution, Et_3N (0.697 mL, 5.00 mmol) and **DLin-EA** (440 mg, 0.812 mmol) were added and the reaction mixture was stirred at room temperature overnight. The reaction mixture was diluted with CH_2Cl_2 and washed with saturated $NaHCO_3$ aq. The organic layer was dried over anhydrous $MgSO_4$, filtered and concentrated. The crude was purified by silica gel column chromatography (0-5% MeOH in CH_2Cl_2) to give **45** (332 mg, 0.505 mmol, 62%, R_f = 0.30 with 5% MeOH in CH_2Cl_2).

^1H NMR (400 MHz, $CDCl_3$) δ 5.27-5.39 (m, 8 H), 4.73 (t, *J* = 5.4 Hz, 1 H), 4.12 (t, *J* = 5.6 Hz, 2 H), 3.08 (t, *J* = 5.9 Hz, 2 H), 2.75 (t, *J* = 6.4 Hz, 4 H), 2.51 (t, *J* = 5.6 Hz, 2 H), 2.25 (s, 6 H), 2.02 (q, *J* = 6.8 Hz, 8 H), 1.22-1.43 (m, 41 H), 0.86 (t, *J* = 6.8 Hz, 6 H). ^{13}C NMR (100 MHz, $CDCl_3$) δ 156.90, 130.39, 130.37, 128.16, 62.26, 58.59, 45.78, 44.40, 38.40, 31.91, 31.74, 30.21, 29.89, 29.81, 29.77, 29.56, 29.54, 27.46, 27.41, 26.88, 25.85, 22.78, 14.28. Molecular weight for $C_{43}H_{81}N_2O_2$ $(M+H)^+$ Calc. 657.63, Found 657.5.

Synthesis of 1-((3aR,4R,6aR)-6-methoxy-2,2-di((9Z,12Z)-octadeca-9,12-dien-1-yl)tetrahydrofuro[3,4-d][1,3]dioxol-4-yl)-N,N-dimethylmethanamine 46

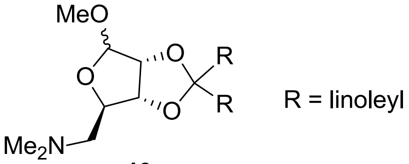

46

Prepared the compound **46** by following similar procedure as compound **6**, using *N*-Cbz-compound of the corresponding product (0.5 g, 0.6 mmol, 1.0 eq) and 1M solution of LAH in THF (0.9 mL, 0.9 mmol, 1.5 eq), gave 0.32 g (76%) of the pure product **46.** ^1H NMR (400 MHz, $CDCl_3$) δ 5.51 – 5.19 (m, 8H), 4.95 (s, 1H), 4.60 (dd, *J* = 29.4, 6.0, 2H), 4.25 (t, *J* = 7.5, 1H), 3.33 (s, 3H), 2.77 (t, *J* = 6.4, 4H), 2.38 (dd, *J* = 7.5, 5.2, 2H), 2.27 (s, 6H), 2.05 (q, *J* =

6.7, 8H), 1.65 (dd, J = 10.1, 5.9, 2H), 1.50 (d, J = 8.9, 2H), 1.33 (ddd, J = 18.9, 12.2, 7.1, 36H), 0.89 (t, J = 6.8, 6H). ^{13}C NMR (101 MHz, cdcl$_3$) δ 130.40, 130.36, 128.17, 128.14, 116.25, 109.69, 85.68, 85.09, 83.54, 77.54, 77.23, 76.91, 63.06, 54.95, 46.17, 37.20, 36.95, 31.75, 30.11, 30.05, 29.91, 29.89, 29.78, 29.71, 29.58, 29.55, 29.53, 27.48, 27.46, 27.42, 25.85, 24.38, 23.33, 22.80, 14.30. Calc. mass for the $C_{45}H_{81}NO_4$: 700.1, found 700.5.

Synthesis of (6Z,9Z,28Z,31Z)-heptatriaconta-6,9,28,31-tetraen-19-yl 4-[ethyl(methyl) amino]butanoate 47

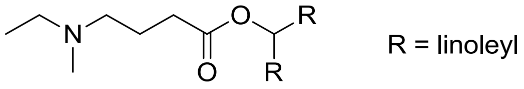

47

A solution of **(DLin-M-C3-Br)** (0.51 g) in tetrahydrofuran (3 mL) was treated with ethylmethylamine (1 g) at room temperature overnight. The solvent was removed on a rotovap and the residue partitioned between dichloromethane and dilute hydrochloric acid. The organic fractions were washed with dilute aqueous sodium bicarbonate solution, dried over anhydrous magnesium sulphate, filtered and the solvent removed. The residue was passed down a silica gel column (20 g) using a 0-6% methanol/dichloromethane elution gradient. Fractions containing the purified product were combined and the solvent removed, yielding **47** as a colorless oil (0.44 g). ^{1}H NMR (400MHz, CDCl$_3$) δ: 5.33 (m; 8H); 4.84 (p; J=6.1Hz; 1H); 2.75 (t; J=6.4Hz; 4H); 2.39 (q; J=7.1Hz; 2H); 2.31 (m; 4H); 2.19 (s; 3H); 2.03 (m; 8H); 1.77 (p; J=7.4Hz; 2H); 1.02 (t; J=7.1Hz; 3H); 0.87 (t; J=6.8Hz; 6H).

Synthesis of (6Z,9Z,28Z,31Z)-heptatriaconta-6,9,28,31-tetraen-19-yl 4-aminobutanoate 48

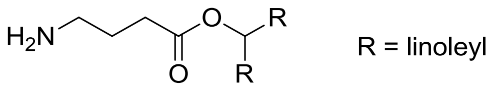

48

A solution of **DLin-MeOH** (0.51 g), N-Boc-4-aminobutanoic acid (0.63 g), 4-N,N-dimethylaminopyridine (0.39 g) and 1-ethyl-3-(3-dimethylaminopropyl)carbodiimide hydrochloride (0.30 g) in dichloromethane (15 mL) was stirred at room temperature overnight. The solution was washed with dilute hydrochloric acid. The organic fractions were dried over anhydrous magnesium sulphate, filtered and the solvent removed on a rotovap. The residue was treated with triflouroacetic acid (2 mL) for an hour. The solution was diluted with methylene chloride, washed with water followed by excess aqueous sodium bicarbonate solution. The organic phase was dried over anhydrous magnesium sulphate, filtered, and the solvent removed. The residue was passed down a silica gel column (20 g) using a 0-10%

methanol/dichloromethane elution gradient. Fractions containing the purified product were combined and the solvent removed, yielding **48** as a colorless oil (0.44 g). ^{1}H NMR (400MHz, $CDCl_3$) δ: 5.32 (m; 8H); 4.84 (p; J=6.2Hz; 1H); 2.75 (t; J=6.3Hz; 4H); 2.72 (m; 2H); 2.32 (t; J=7.4Hz; 2H); 2.03 (m; 8H); 1.75 (p; J=7.1Hz; 2H); 0.87 (t; J=6.8Hz; 6H).

Synthesis of 3-(dimethylamino)propyl ((11Z,14Z)-2-((9Z,12Z)-octadeca-9,12-dien-1-yl)icosa-11,14-dien-1-yl)carbamate 49

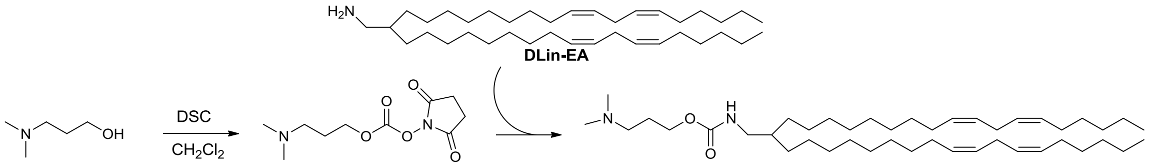

49

To a solution of *N,N'*-disuccinimidyl carbonate (5.50 g, 21.5 mmol) in CH_2Cl_2 (200 mL), 3-dimethylamino-1-propanol (2.43 g, 23.6 mmol) was added dropwise. The resulting mixture was stirred at room temperature overnight. Taken up 50 mL of the solution, Et_3N (0.697 mL, 5.00 mmol) and **DLin-EA** (1.71 g, 3.15 mmol) were added and the reaction mixture was stirred at room temperature overnight. The reaction mixture was diluted with CH_2Cl_2 and washed with saturated $NaHCO_3$ aq. The organic layer was dried over anhydrous $MgSO_4$, filtered and concentrated. The crude was purified by silica gel column chromatography (0-5% MeOH in CH_2Cl_2) to give **49** (1.14 g, 1.70 mmol, 54%, R_f = 0.13 with 5% MeOH in CH_2Cl_2). ^{1}H NMR (400 MHz, $CDCl_3$) δ 5.30-5.42 (m, 8 H), 4.62 (s, 1 H), 4.10 (t, *J* = 6.4 Hz, 2 H), 3.10 (t, *J* = 5.8 Hz, 2 H), 2.77 (t, *J* = 6.4 Hz, 4 H), 2.34 (t, *J* = 7.4 Hz, 2 H), 2.23 (s, 6 H), 2.05 (q, *J* = 6.8 Hz, 8 H), 1.77-1.82 (m, 2 H), 1.27-1.44 (m, 41 H), 0.89 (t, *J* = 6.8 Hz, 6 H). ^{13}C NMR (100 MHz, $CDCl_3$) δ 156.75, 130.19, 130.15, 127.95, 127.94, 63.10, 56.30, 45.47, 44.22, 38.24, 31.69, 31.54, 30.03, 29.69, 29.62, 29.57, 29.36, 29.34, 27.46, 27.25, 27.21, 26.69, 25.64, 22.59, 14.09. Molecular weight for $C_{44}H_{83}N_2O_2$ $(M+H)^+$ Calc. 671.65, Found 671.5.

Synthesis of 1-((3aR,4R,6aS)-2,2-di((9Z,12Z)-octadeca-9,12-dien-1-yl)tetrahydrofuro[3,4-d][1,3]dioxol-4-yl)-N,N-dimethylmethanamine 50

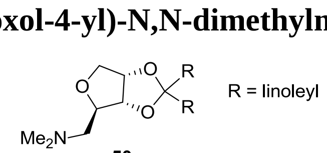

50

Prepared the compound **25a** by following a similar procedure as compound **9**, using *N*-Cbz-compound **24a** (0.3 g, 0.38 mmol, 1.0 eq) and 1M solution of LAH in THF (0.76 mL, 0.76 mmol, 1.5 eq). The pure product **25a** (0.17 g, 69%) was isolated as a thick oil. ^{1}H NMR (400 MHz, $CDCl_3$) δ 5.54 – 5.22 (m, 8H), 4.76 (dd, *J* = 7.5, 3.0, 1H), 4.50 (dd, *J* = 6.4, 1.8, 1H),

4.18 – 4.08 (m, 1H), 3.95 (dd, J = 10.6, 1.4, 1H), 3.84 (dd, J = 10.6, 4.3, 1H), 2.77 (t, J = 6.4, 4H), 2.27 (s, 6H), 2.04 (q, J = 6.8, 8H), 1.68 (dd, J = 9.9, 6.2, 2H), 1.53 (d, J = 8.7, 2H), 1.49 – 1.17 (m, 38H), 0.89 (t, J = 6.8, 6H). Calc. mass for the $C_{44}H_{79}NO_3$: 670.1, found 670.5.

Synthesis of (3aR,5R,7aR)-N,N-dimethyl-2,2-di((9Z,12Z)-octadeca-9,12-dien-1-yl)hexahydrobenzo[d][1,3]dioxol-5-amine 51

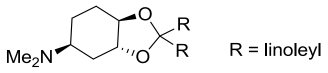

51

Prepared **51** by following similar procedure as compound **9**, using *N*-Cbz-compound (0.52 g, 0.66 mmol) and 1M solution of LAH in THF (1.32 mL, 1.32 mmol), gave 0.4 g (91%) of the pure product **19**. ^{1}H NMR (400 MHz, $CDCl_3$) δ 5.50 – 5.22 (m, 8H), 3.67 (ddd, J = 12.1, 8.9, 3.3, 1H), 3.39 – 3.16 (m, 1H), 2.77 (t, J = 6.4, 4H), 2.44 (dd, J = 12.7, 2.2, 1H), 2.29 (s, 1H), 2.24 (s, 6H), 2.06 (dt, J = 13.5, 10.9, 8H), 1.99 – 1.87 (m, 1H), 1.63 (dd, J = 16.1, 9.8, 5H), 1.51 – 1.14 (m, 39H), 0.89 (t, J = 6.7, 6H). ^{13}C NMR (101 MHz, $CDCl_3$) δ 130.40, 128.14, 112.25, 81.03, 77.54, 77.22, 76.91, 75.60, 61.55, 44.11, 38.19, 38.14, 31.74, 30.24, 30.21, 29.89, 29.80, 29.77, 29.74, 29.57, 29.54, 27.46, 27.41, 25.84, 24.86, 24.36, 24.23, 22.80, 14.31. Calc. mass for the $C_{45}H_{81}NO_2$: 668.1, found 668.5.

Synthesis of (11*Z*,14*Z*)-*N*,*N*-dimethyl-2-((9*Z*,12*Z*)-octadeca-9,12-dien-1-yl)icosa-11,14-dien-1-amine 52

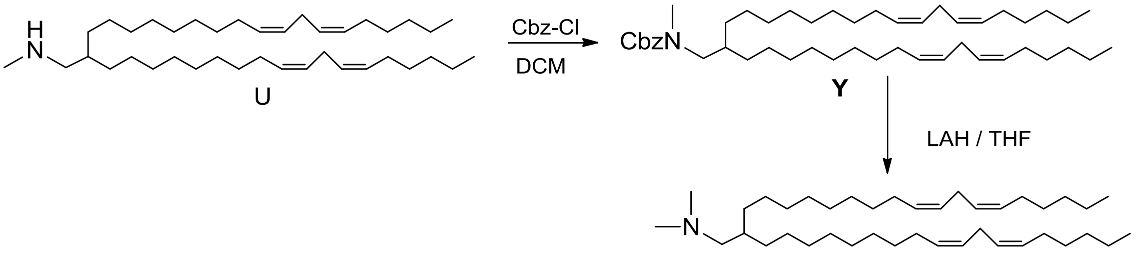

52

Synthesis of benzyl methyl((11Z,14Z)-2-((9Z,12Z)-octadeca-9,12-dien-1-yl)icosa-11,14-dien-1-yl)carbamate Y: To a suspension of lithium aluminiumhydride (4.05 g, 0.1066 mol) in THF (360 mL) under argon atmosphere at 0^{0}C, was added a solution of **U** (36 g, 0.0533 mol) in THF drop-wise. After addition, it was allowed to warm to toom temperature and stirred for 15 hours (TLC). The reaction mass was cooled to 0°C and quenched with saturated solution of sodium sulfate followed by ethyl acetate. It was filtered through celite bed and washed with ethyl acetate. Combined filtrates were evaporated and purified by silica gel using 100% methanol to get the final product **Y** (26g, 87%). ^{1}H NMR (400MHz, $CDCl_3$): δ 0.87 (t, 6H, J = 6.8Hz), 1.27 (m, 42H), 2.03 (q, 8H, J_1 = 6.8Hz, J_2 = 6.8Hz), 2.45 (s, 3H), 2.49 (d, 2H, J = 6Hz), 2.76 (t, 4H, J = 6.4Hz), 5.30 (m, 8H).

Synthesis of (11Z,14Z)-N,N-dimethyl-2-((9Z,12Z)-octadeca-9,12-dien-1-yl)icosa-11,14-dien-1-amine 52: Compound **Y** (4 g, 0.0072 mol) was dissolved in DCM (40 mL) under argon atmosphere and cooled to 0°C. To this solution 2,6-Lutidine (1.7 mL, 0.0144 mol) was added drop-wise followed by benzyl chloroformate (1.0 mL, 0.0074 mol). It was then allowed to warm to 20°C and stirred for one hour (TLC). Then it was diluted with DCM (200 mL), washed with 10% citric acid (2x200 mL), water and brine. The organic layer was dried over anhydrous sodium sulfate and evaporated at reduced pressure to obtain crude product, which was purified by silica gel using 3% ether/hexane to get the final product **52** (3.80 g, 76%). ^{1}H NMR (400MHz, $CDCl_3$) δ 0.87 (t, 6H, J=6.8Hz), 1.20 (m, 44H), 2.02 (q, 8H, J_1 = 6.8Hz, J_2 = 6.8Hz), 2.76 (t, 4H, J = 6.4Hz), 2.89 (d, 3H, J = 6Hz), 3.14 (m, 2H), 5.12 (s, 2H), 5.30 (m, 8H), 7.26 (m, 4H).

Synthesis of (3aS,4S,5R,7R,7aR)-N,N-dimethyl-2-((7Z,10Z)-octadeca-7,10-dien-1-yl)-2-((9Z,12Z)-octadeca-9,12-dien-1-yl)hexahydro-4,7-methanobenzo[d][1,3]dioxol-5-amine 54:

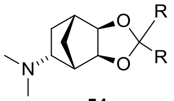

R = linoleyl

54

Prepared the compound **54** by following similar procedure as compound **6**, using *N*-Cbz-compound (1.4 g, 1.75 mmol, 1.0 eq) and 1M solution of LAH in THF (2.62 mL, 2.62 mmol, 1.5 eq), gave 1.0 g (84%) of the pure product **54**. ^{1}H NMR (400 MHz, $CDCl_3$) δ 5.50 – 5.20 (m, 8H), 3.91 (dd, *J* = 15.3, 5.4, 2H), 2.77 (t, *J* = 6.3, 4H), 2.43 (s, 1H), 2.29 (d, *J* = 4.0, 1H), 2.18 (s, 6H), 2.04 (q, *J* = 6.7, 8H), 1.64 (dd, *J* = 10.4, 5.7, 4H), 1.50 (s, 2H), 1.45 – 1.13 (m, 39H), 0.89 (t, *J* = 6.7, 6H). ^{13}C NMR (101 MHz, $cdcl_3$) δ 130.41, 130.39, 130.38, 130.34, 128.19, 128.15, 113.17, 81.76, 81.21, 77.54, 77.22, 76.90, 65.76, 44.14, 43.40, 40.30, 36.17, 35.25, 32.05, 31.75, 30.21, 29.89, 29.77, 29.74, 29.71, 29.57, 29.52, 29.11, 27.46, 27.42, 25.85, 24.82, 24.08, 22.79, 14.29. Calc. mass for the $C_{46}H_{81}NO_2$: 680.1, found 680.5.

Synthesis of N,N-dimethyl-3,4-bis((9Z,12Z)-octadeca-9,12-dien-1-yloxy)butan-1-amine 55

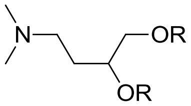

R = linoleyl

55

Using a similar procedure used for the synthesis of **42** treatment of NaH (0.6 g, 15 mmol) with 1-dimethylaminobutan-3,4-diol (0.5 g, 3.75 mmol) in anhydrous benzene followed by

linoleyl mesylate (3.22 g, 9.37 mmol) in refluxing conditions for 10 h followed by usual work-up and purification provided the pure product (0.56 g, 43%) as clear liquid. 1H NMR (400 MHz, CDCl3) δ 5.48 – 5.20 (m, 8H), 3.57 (dt, J = 9.2, 6.6 Hz, 1H), 3.50 – 3.30 (m, 7H), 2.82 – 2.68 (m, 4H), 2.40 (dd, J = 23.0, 15.8 Hz, 2H), 2.34 – 2.14 (m, 7H), 2.12 – 1.84 (m, 9H), 1.78 – 1.59 (m, 2H), 1.59 – 1.43 (m, 5H), 1.43 – 1.12 (m, 39H), 0.88 (dd, J = 8.7, 5.0 Hz, 6H).

Synthesis of 3-(4,5-di((8Z,11Z)-heptadeca-8,11-dien-1-yl)-1,3-dioxolan-2-yl)-N,N-dimethylpropan-1-amine 56

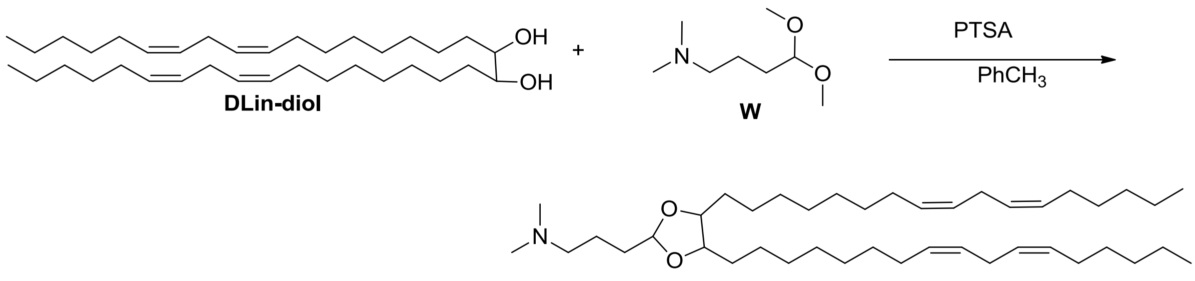

56

Using a similar procedure used for the synthesis of **28**, using 1.06 g (2 mmol) of **DLin-diol** and 400 mg (2.5 mmol) of the amine **W**, 0.7 g (56%) the ketalized product **56** was isolated after column purification. ^{1}H NMR (400 MHz, $CDCl_3$) δ 5.44 – 5.24 (m, 8H), 5.02 – 4.83 (m, 1H), 3.87 (dd, *J* = 17.2, 14.0, 1H), 3.51 (dd, *J* = 15.2, 8.5, 1H), 2.72 (dt, *J* = 9.5, 5.0, 4H), 2.32 – 2.25 (m, 2H), 2.22 (s, 6H), 2.11 – 1.94 (m, 9H), 1.68 – 1.38 (m, 11H), 1.38 – 1.18 (m, 35H), 0.84 (dt, *J* = 10.7, 5.9, 6H). Calc. mass for the $C_{42}H_{77}NO_2$: 628.07; found 628.5.

siRNA synthesis. All siRNAs were synthesized by Alnylam and were characterized by electrospray mass spectrometry and anion exchange HPLC. The sequences for the sense and antisense strands of FVII has been reported elsewhere (see reference 6). The sequences for the sense and antisense strands of the TTR siRNA is as follows:

siTTR sense: 5'- GuAAccAAGAGuAuuccAudTdT-3' antisense: 5'-AUGGAAuACUCUUGGUuACdTdT-3'. 2'-O-Me modified nucleotides are in lower case, siRNAs were generated by annealing equimolar amounts of complementary sense and antisense strands.

Preformed vesicle method to formulate LNP-siRNA systems. LNP-siRNA systems were made using the preformed vesicle method. Cationic lipid, DSPC, cholesterol and PEG-lipid were solubilized in ethanol at a molar ratio of 40/10/40/10, respectively. The lipid mixture was added to an aqueous buffer (50 mM citrate, pH 4) with mixing to a final ethanol and lipid

concentration of 30% (vol/vol) and 6.1 mg/mL respectively and allowed to equilibrate at room temperature for 2 min before extrusion. The hydrated lipids were extruded through two stacked 80 nm pore-sized filters (Nuclepore) at 22°C using a Lipex Extruder (Northern Lipids, Vancouver, Canada) until a vesicle diameter of 70-90 nm, as determined by dynamic light scattering analysis, was obtained. This generally required 1-3 passes. The siRNA (solubilized in a 50 mM citrate, pH 4 aqueous solution containing 30% ethanol) was added to the vesicles (pre-equilibrated to 35°C) at a rate of ~5 mL/min with mixing. After a final target siRNA/lipid ratio of 0.06 (wt/wt) was achieved, the mixture was incubated for a further 30 min at 35°C to allow vesicle re-organization and encapsulation of the siRNA. The ethanol was then removed and the external buffer replaced with PBS (155 mM NaCl, 3 mM Na_2HPO_4, 1 mM KH_2PO_4, pH 7.5) by dialysis.

In vivo screening of cationic lipids for FVII activity. Eight to 10 week old, female C57BL/6 mice were obtained from Harlan. Mice were held in a pathogen-free environment and all procedures involving animals were performed in accordance with guidelines established by the Canadian Council for Animal Care (CCAC). LNP-siRNA systems containing Factor VII siRNA were diluted to the appropriate concentrations in sterile PBS immediately prior to use and the formulations were administered intravenously via the lateral tail vein in a total volume of 10 ml/kg. After 24 hours, animals were anesthetized with Ketamine/Xylazine and blood was collected by cardiac puncture and processed to serum (Microtainer Serum Separator Tubes; Becton Dickinson, Franklin Lakes, NJ). Serum was tested immediately or stored at -70oC for later analysis for Factor VII levels.

Measurement of FVII protein in serum. Serum Factor VII levels were determined using the colorimetric Biophen VII assay kit (Anaira, USA). Briefly, serially diluted pooled control serum (200% - 3.125%) and appropriately diluted serum samples from treated animals (n=4-5 animals per dose level) were analyzed in 96-well, flat bottom, non-binding polystyrene assay plates (Corning, Corning, NY) using the Biophen VII kit according to manufacturer's instructions. Absorbance was measured at 405 nm and a calibration curve was generated using the serially diluted control serum to determine levels of Factor VII in serum from treated animals, relative to the saline-treated control animals. ED_{50} values for each formulation were derived from linear interpolation of the Factor VII activity profile, and included data points within 10-90% residual Factor VII activity (typically three to six points). Formulations containing novel lipids were always screened with one or more benchmark

formulations to control and assess assay variability over time, and formulations with promising activity were repeated, with an expanded number of dose levels.

In situ determination of pK_a using TNS. The pKa of each cationic lipid was determined in LNP using TNS and PFV comprised of cationic lipid/DSPC/cholesterol/PEG-lipid (40/10/40/10 mol%) in PBS at a concentration of ~6 mM total lipid. TNS was prepared as a 100 μM stock solution in distilled water. Vesicles were diluted to 100 μM lipid in 2 mL of buffered solutions containing , 10 mM HEPES, 10 mM MES, 10 mM ammonium acetate, 130 mM NaCl, where the pH ranged from 2.5 to 11. An aliquot of the TNS solution was added to give a final concentration of 1 μM and following vortex mixing fluorescence intensity was measured at room temperature in a SLM Aminco Series 2 Luminescence Spectrophotometer using excitation and emission wavelengths of 321 nm and 445 nm. A sigmoidal best fit analysis was applied to the fluorescence data and the pK_a was measured as the pH giving rise to half-maximal fluorescence intensity. The ratio of charged to uncharged cationic lipid at pH 5.5 and pH 7.4 for the different amino lipid pK_a values (plotted in Figure 4 of the manuscript as % charged or % uncharged) was calculated using the Henderson-Hasselbach equation.

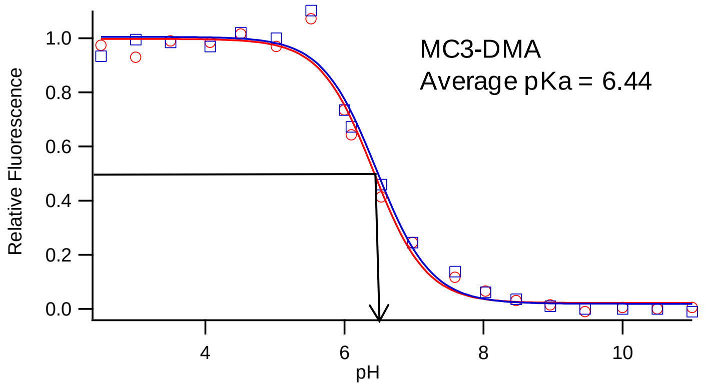

Figure S1. Fluorescence titration of MC3-DMA (**16**), conducted in duplicate giving an average pKa = 6.44.

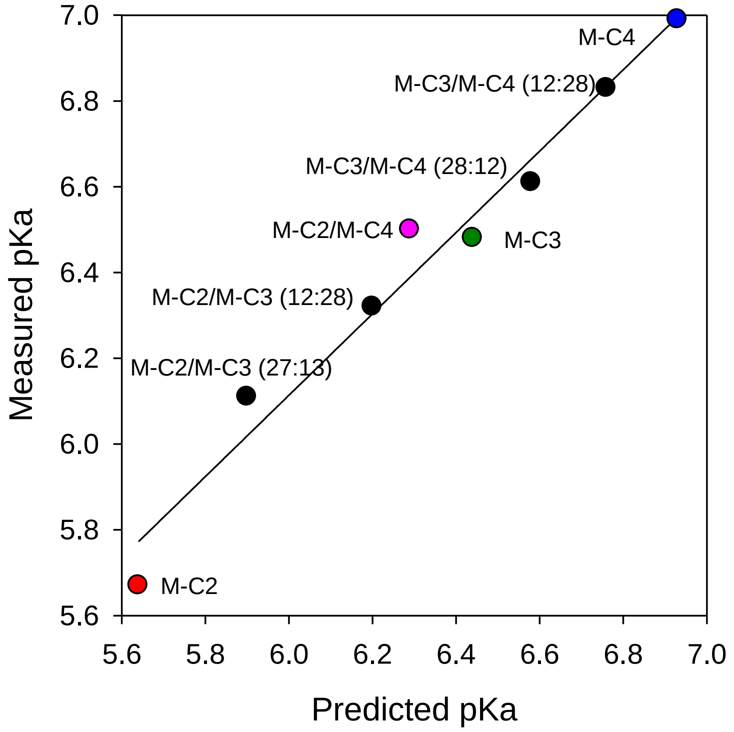

Figure S2. Correlation between measured and predicted pKa values for lipid mixtures. Mixtures of MC2-DMA (**15**), MC3-DMA (**16**) and MC4-DMA (**17**) were made to incrementally move the average LNP pK_a between 5.64 and 6.93. Lipids were mixed at mole ratios calculated to achieve a desired average pKa for the lipid mixture. Data plotted in Figure S2 show a strong correlation between predicted and measured pK_a values. The numbers in parenthesis indicate the amount of each lipid in each mixture to make up the 40 mole % amino lipid component. Lipids used above all contain the dimethylamine (DMA) moiety (Chart 1B of manuscript) consequently, for clarity, DMA has been dropped from the data point labels in the graph. Each point represents the average of two titrations.

***In vivo* non-human primate experiments.** All procedures using cynomolgus monkeys were conducted by a certified contract research organization using protocols consistent with local, state, and federal regulations, as applicable, and approved by the IACUC. LNP were prepared using a spontaneous formation procedure previously described (see references 5 and 12 in manuscript) and were composed of DLin-MC3-DMA/DSPC/Cholesterol/PEG-lipid (50/10/38.5/1.5 mol/mol) with a final siRNA-to-lipid ratio of 0.9 (wt/wt). Cynomolgus monkeys (n = 3 per group) received either 0.03, 0.1 or 0.3 mg/kg siTTR encapsulated in LNP as 15 minute intravenous infusions (5 mL/kg) via the cephalic vein. Animals were sacrificed at 48 hours post-administration, and a 0.15-0.20 g sample of the left lateral lobe of the liver

was collected and snap-frozen in liquid nitrogen. Prior studies have established uniformity of silencing activity throughout the liver 6. TTR mRNA levels, relative to GAPDH mRNA levels, were determined in liver samples using a branched DNA assay (QuantiGene Assay, Affymetrix, CA) 6. Clinical chemistry and hematology parameters were analyzed at pre-dose and 48 hours post-administration. P-values were calculated for comparison of K2C-SNALP-treated animals with PBS-treated animals using analysis of variance (ANOVA, single-factor) with an alpha value of 0.05. P-values less than 0.05 were considered significant.

Complete References 4, 5 and 12

[4]. Zimmermann, T. S.; Lee, A. C.; Akinc, A.; Bramlage, B.; Bumcrot, D.; Fedoruk, M. N.; Harborth, J.; Heyes, J. A.; Jeffs, L. B.; John, M.; Judge, A. D.; Lam, K.; McClintock, K.; Nechev, L. V.; Palmer, L. R.; Racie, T.; Rohl, I.; Seiffert, S.; Shanmugam, S.; Sood, V.; Soutschek, J.; Toudjarska, I.; Wheat, A. J.; Yaworski, E.; Zedalis, W.; Koteliansky, V.; Manoharan, M.; Vornlocher, H. P.; MacLachlan, I. RNAi-mediated gene silencing in non-human primates. *Nature* **2006**, *441* (7089), 111-114.

[5]. Semple, S. C.; Akinc, A.; Chen, J.; Sandhu, A. P.; Mui, B. L.; Cho, C. K.; Sah, D. W.; Stebbing, D.; Crosley, E. J.; Yaworski, E.; Hafez, I. M.; Dorkin, J. R.; Qin, J.; Lam, K.; Rajeev, K. G.; Wong, K. F.; Jeffs, L. B.; Nechev, L.; Eisenhardt, M. L.; Jayaraman, M.; Kazem, M.; Maier, M. A.; Srinivasulu, M.; Weinstein, M. J.; Chen, Q.; Alvarez, R.; Barros, S. A.; De, S.; Klimuk, S. K.; Borland, T.; Kosovrasti, V.; Cantley, W. L.; Tam, Y. K.; Manoharan, M.; Ciufolini, M. A.; Tracy, M. A.; de, F. A.; MacLachlan, I.; Cullis, P. R.; Madden, T. D.; Hope, M. J. Rational design of cationic lipids for siRNA delivery. *Nat. Biotechnol.* **2010**, *28* (2), 172-176.

[12]. Akinc, A.; Querbes, W.; De, S.; Qin, J.; Frank-Kamenetsky, M.; Jayaprakash, K. N.; Jayaraman, M.; Rajeev, K. G.; Cantley, W. L.; Dorkin, J. R.; Butler, J. S.; Qin, L.; Racie, T.; Sprague, A.; Fava, E.; Zeigerer, A.; Hope, M. J.; Zerial, M.; Sah, D. W.; Fitzgerald, K.; Tracy, M. A.; Manoharan, M.; Koteliansky, V.; Fougerolles, A.; Maier, M. A. Targeted delivery of RNAi therapeutics with endogenous and exogenous ligand-based mechanisms. *Mol. Ther.* **2010**, *18* (7), 1357-1364.

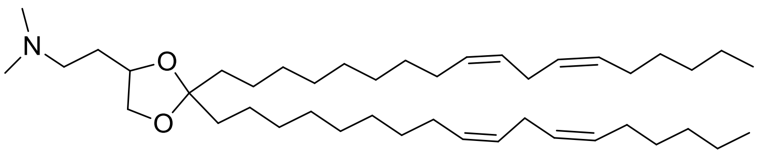
N
O
O

1

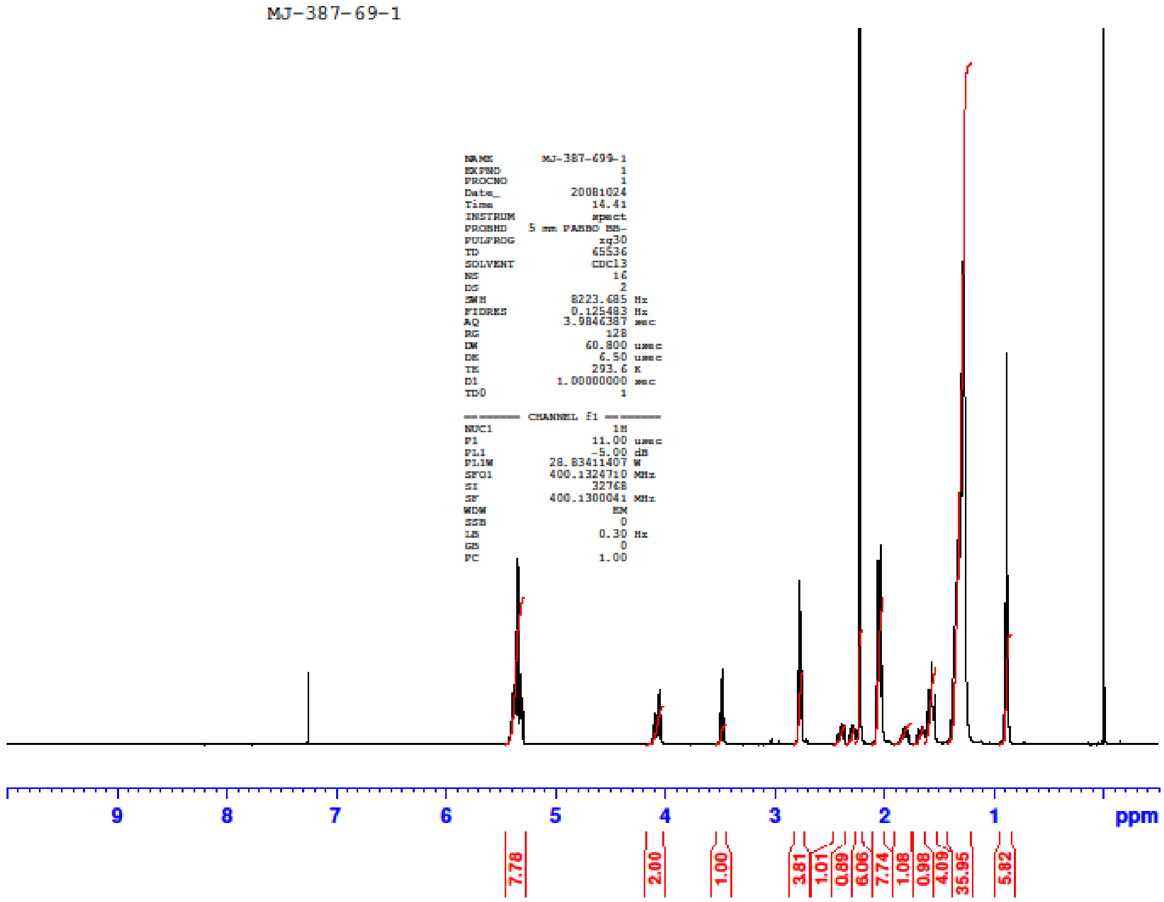
MJ-387-69-1
NAME MJ-387-699-1
EXPNO 1
PROCNO 1
Date_ 20081024
Time 14.41
INSTRUM spect
PROBHD 5 mm PABBO BB-
PULPROG zg30
TD 65536
SOLVENT CDCl3
NS 16
DS 2
SWH 8223.685 Hz
FIDRES 0.125483 Hz
AQ 3.9846387 sec
RG 128
DW 60.800 usec
DE 6.50 usec
TE 293.6 K
D1 1.00000000 sec
TD0 1
======== CHANNEL f1 ========
NUC1 1H
P1 11.00 usec
PL1 -5.00 dB
PL1W 28.83411407 W
SFO1 400.1324710 MHz
SI 32768
SF 400.1300041 MHz
WDW EM
SSB 0
LB 0.30 Hz
GB 0
PC 1.00
9
8
7
6
5
4
3
2
1
ppm
7.78
2.00
1.00
3.81
1.01
0.89
6.06
7.74
1.08
0.98
4.09
35.95
5.82

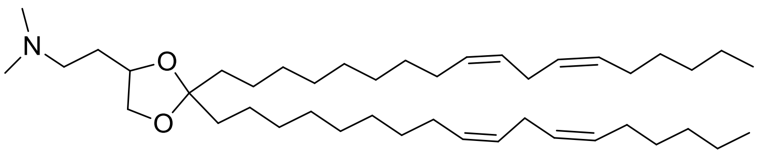

1

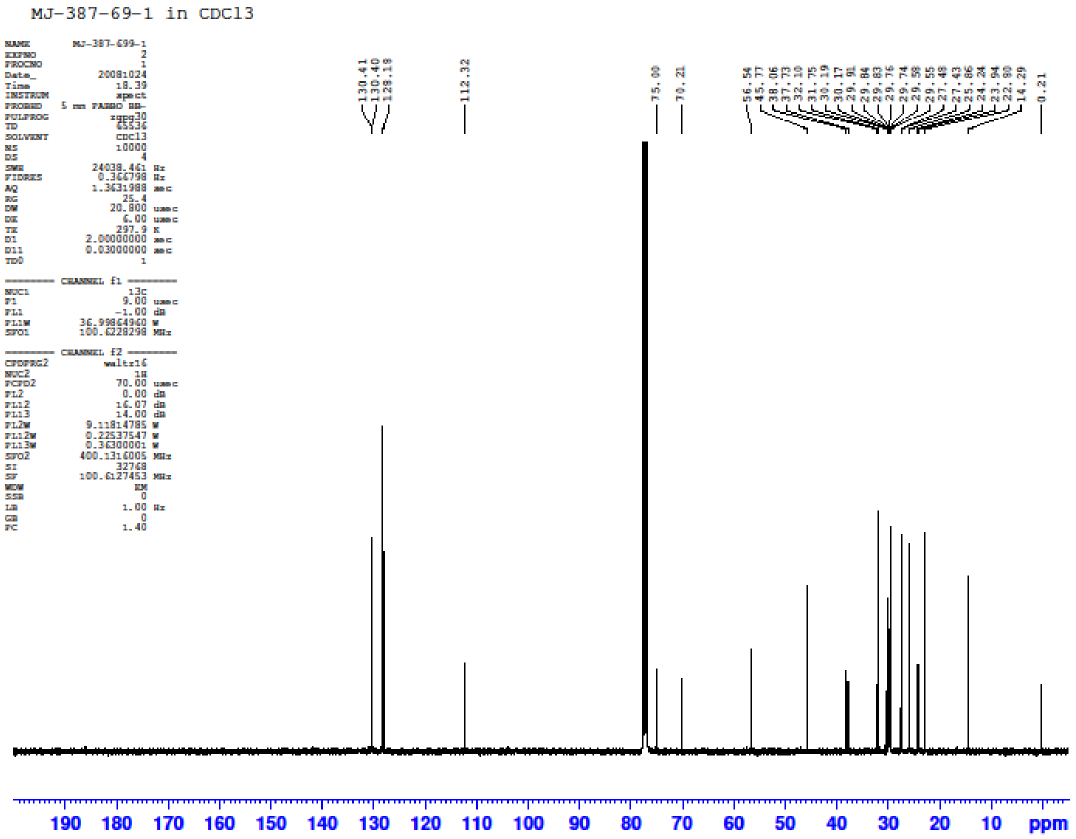
MJ-387-69-1 in CDCl3
130.41
130.40
128.19
112.32
75.00
70.21
56.54
45.77
38.06
37.73
32.10
31.75
30.19
30.17
29.91
29.84
29.83
29.76
29.74
29.58
29.55
27.48
27.43
25.86
24.24
23.94
22.90
14.29
0.21
190 180 170 160 150 140 130 120 110 100 90 80 70 60 50 40 30 20 10 ppm

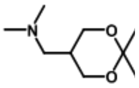

N
O
O

2

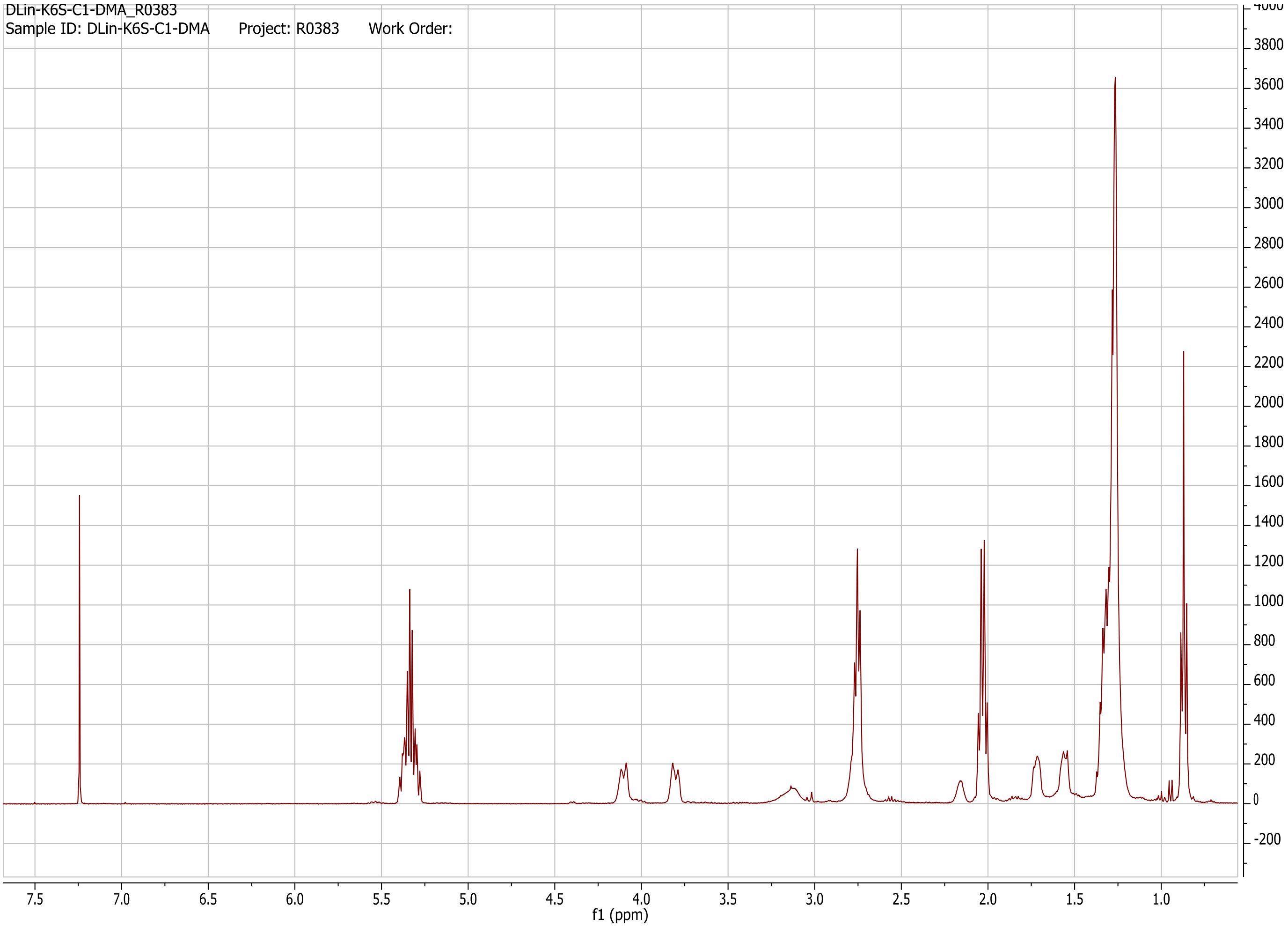

DLin-K6S-C1-DMA_R0383
Sample ID: DLin-K6S-C1-DMA Project: R0383 Work Order:
f1 (ppm)
7.5
7.0
6.5
6.0
5.5
5.0
4.5
4.0
3.5
3.0
2.5
2.0
1.5
1.0
3800
3600
3400
3200
3000
2800
2600
2400
2200
2000
1800
1600
1400
1200
1000
800
600
400
200
0
-200

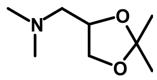

3

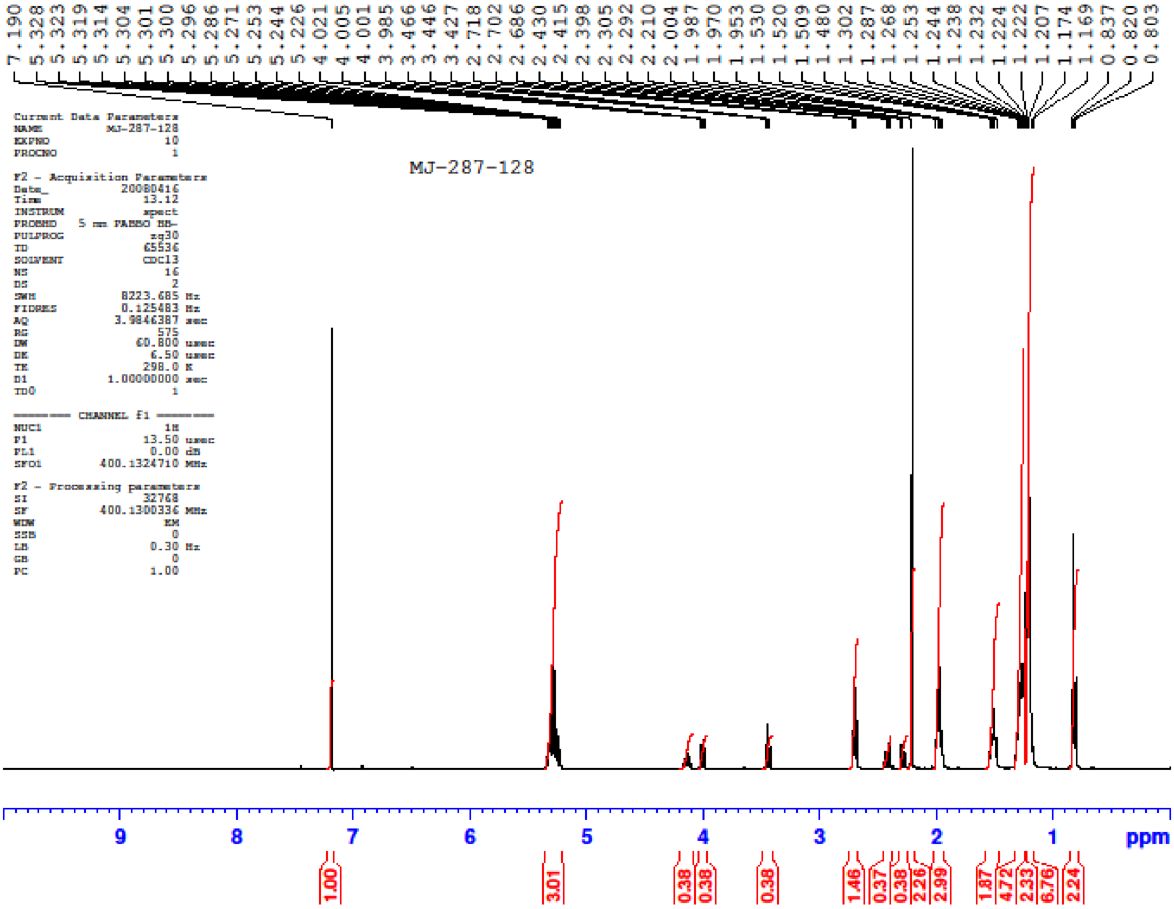

MJ-287-128
Current Data Parameters
NAME MJ-287-128
EXPNO 10
PROCNO 1
F2 - Acquisition Parameters
Date_ 20080416
Time 13.12
INSTRUM spect
PROBHD 5 mm PABBO BB-
PULPROG zg30
TD 65536
SOLVENT CDCl3
NS 16
DS 2
SWH 8223.685 Hz
FIDRES 0.125483 Hz
AQ 3.9846387 sec
RG 575
DW 60.800 usec
DE 6.50 usec
TE 298.0 K
D1 1.00000000 sec
TD0 1
======== CHANNEL f1 ========
NUC1 1H
P1 13.50 usec
PL1 0.00 dB
SFO1 400.1324710 MHz
F2 - Processing parameters
SI 32768
SF 400.1300336 MHz
WDW EM
SSB 0
LB 0.30 Hz
GB 0
PC 1.00
ppm

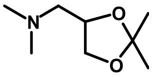
N
O
O

3

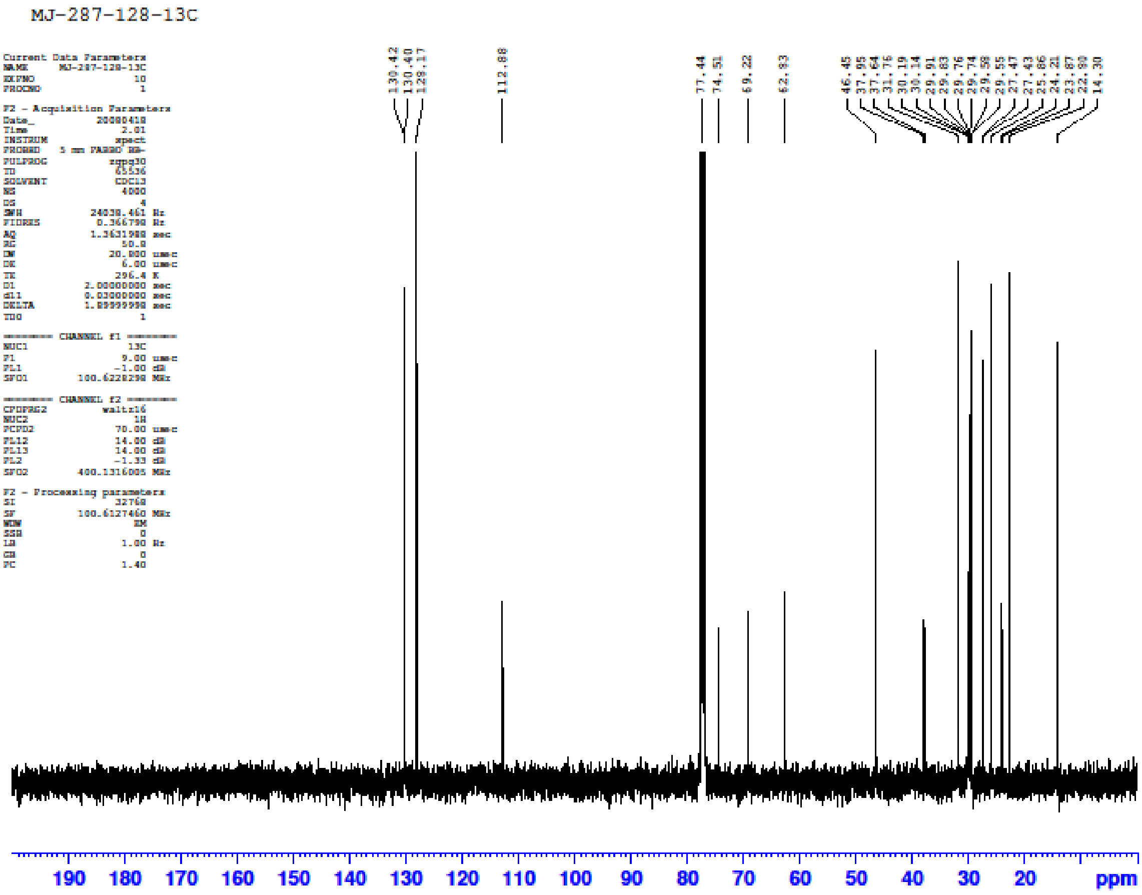
MJ-287-128-13C
Current Data Parameters
NAME MJ-287-128-13C
EXPNO 10
PROCNO 1
F2 - Acquisition Parameters
Date_ 20080418
Time 2.01
INSTRUM spect
PULPROG zgpg30
TD 65536
SOLVENT CDCl3
NS 4000
DS 4
SWH 24038.461 Hz
FIDRES 0.366798 Hz
AQ 1.3631988 sec
RG 50.8
DW 20.800 usec
DE 6.00 usec
TE 296.4 K
D1 2.00000000 sec
d11 0.03000000 sec
DELTA 1.89999998 sec
TD0 1
======== CHANNEL f1 ========
NUC1 13C
P1 9.00 usec
PL1 -1.00 dB
SFO1 100.6228298 MHz
======== CHANNEL f2 ========
CPDPRG2 waltz16
NUC2 1H
PCPD2 70.00 usec
PL12 14.00 dB
PL13 14.00 dB
PL2 -1.33 dB
SFO2 400.1316005 MHz
F2 - Processing parameters
SI 32768
SF 100.6127460 MHz
WDW EM
SSB 0
LB 1.00 Hz
GB 0
PC 1.40
130.42
130.40
129.17
112.88
77.44
74.51
69.22
62.83
46.45
37.95
37.64
31.76
30.19
30.14
29.91
29.83
29.76
29.74
29.58
29.55
27.47
27.43
25.86
24.21
23.87
22.90
14.30
190
180
170
160
150
140
130
120
110
100
90
80
70
60
50
40
30
20
ppm

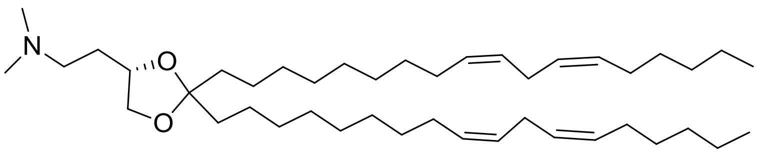

4

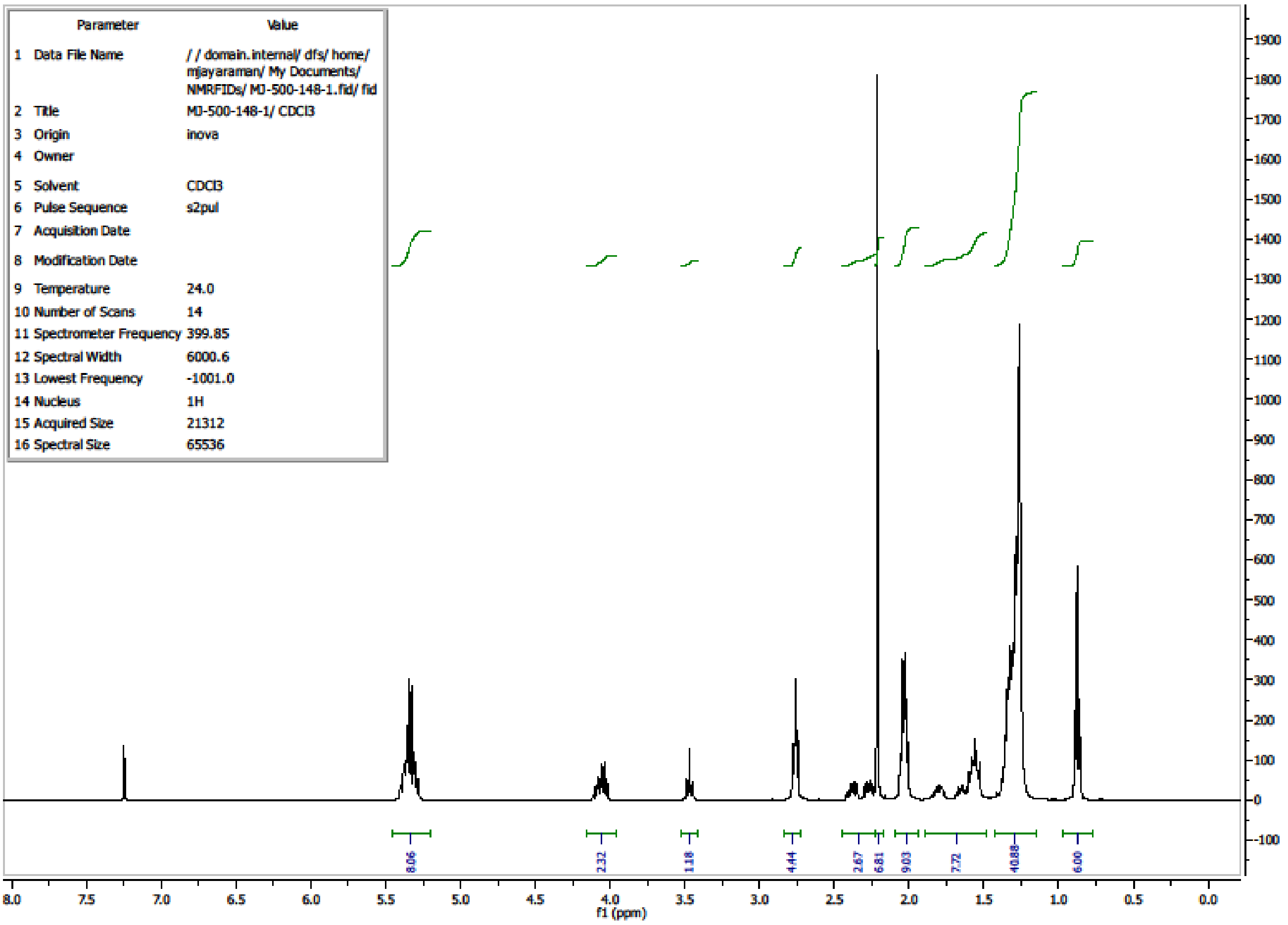

Parameter
Value
1 Data File Name
/ / domain.internal/ dfs/ home/ mjayaraman/ My Documents/ NMRFIDs/ MJ-500-148-1.fid/ fid
2 Title
MJ-500-148-1/ CDCl3
3 Origin
inova
4 Owner
5 Solvent
CDCl3
6 Pulse Sequence
s2pul
7 Acquisition Date
8 Modification Date
9 Temperature
24.0
10 Number of Scans
14
11 Spectrometer Frequency
399.85
12 Spectral Width
6000.6
13 Lowest Frequency
-1001.0
14 Nucleus
1H
15 Acquired Size
21312
16 Spectral Size
65536
8.06
2.32
1.18
4.44
2.67
6.81
9.03
7.72
40.88
6.00
f1 (ppm)

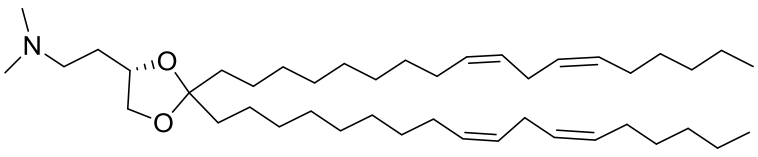
N
O
O

4

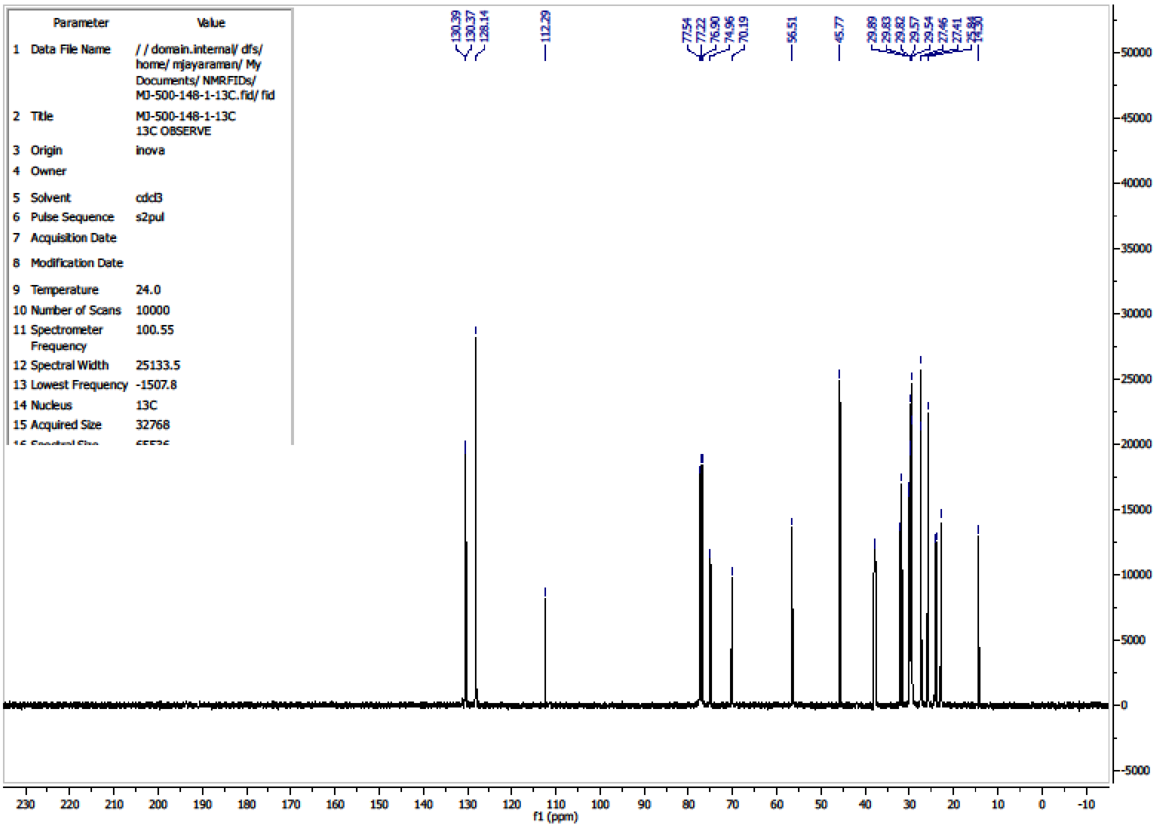
Parameter | Value
1 Data File Name | / / domain.internal/ dfs/ home/ mjayaraman/ My Documents/ NMRFIDs/ MJ-500-148-1-13C.fid/ fid
2 Title | MJ-500-148-1-13C 13C OBSERVE
3 Origin | inova
4 Owner
5 Solvent | cdcl3
6 Pulse Sequence | s2pul
7 Acquisition Date
8 Modification Date
9 Temperature | 24.0
10 Number of Scans | 10000
11 Spectrometer Frequency | 100.55
12 Spectral Width | 25133.5
13 Lowest Frequency | -1507.8
14 Nucleus | 13C
15 Acquired Size | 32768
16
130.39
130.37
128.14
112.29
77.54
77.22
76.90
74.96
70.19
56.51
45.77
29.89
29.83
29.82
29.57
29.54
27.46
27.41
50000
45000
40000
35000
30000
25000
20000
15000
10000
5000
0
-5000
230 220 210 200 190 180 170 160 150 140 130 120 110 100 90 80 70 60 50 40 30 20 10 0 -10
f1 (ppm)

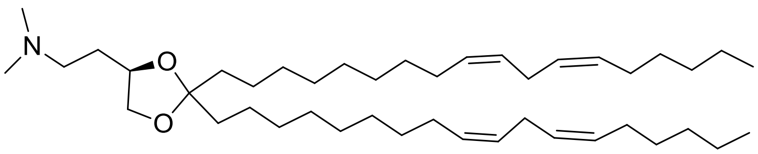

5

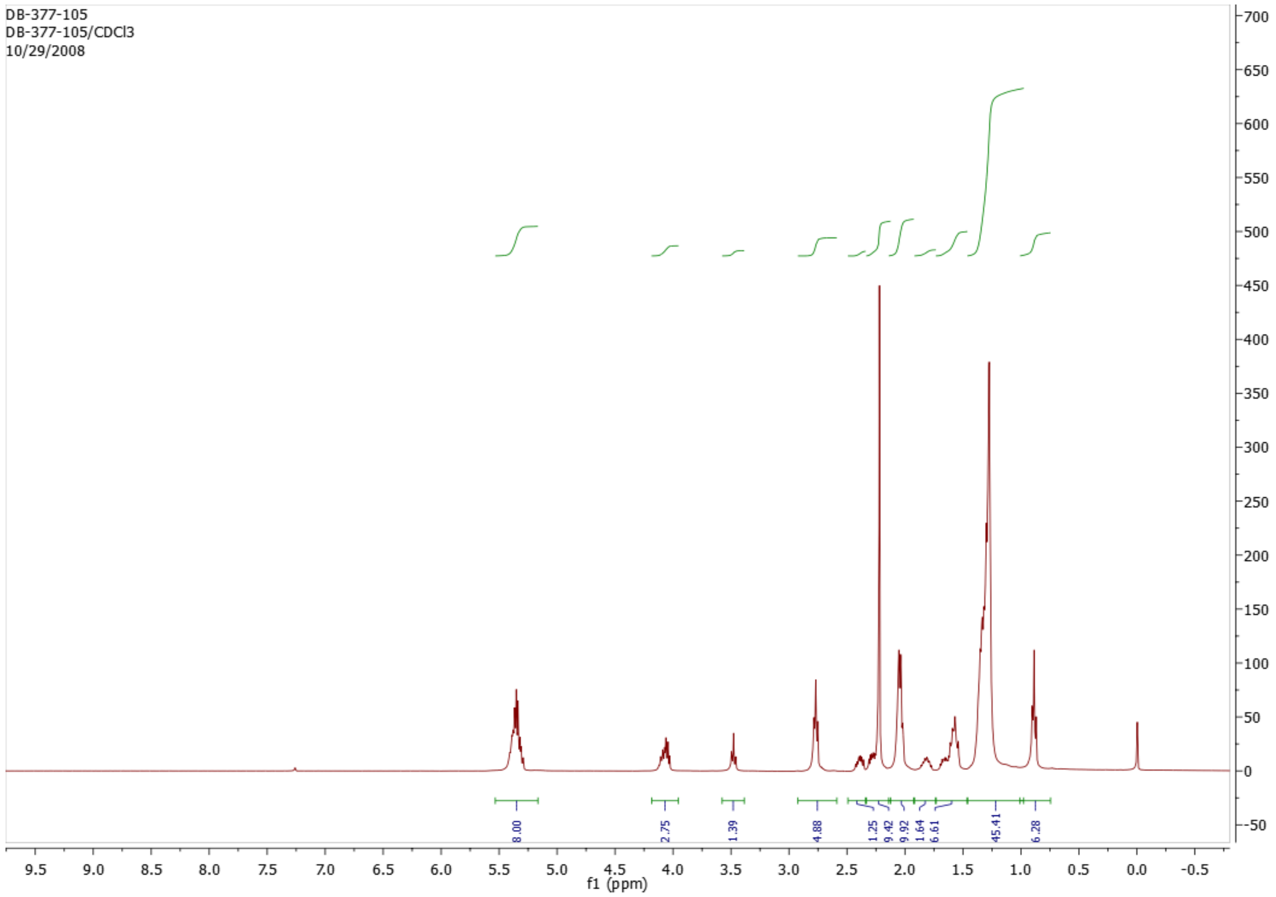

DB-377-105
DB-377-105/CDCl3
10/29/2008
8.00
2.75
1.39
4.88
1.25
9.42
9.92
1.64
6.61
45.41
6.28
f1 (ppm)

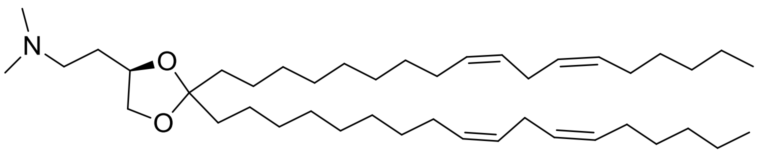

5

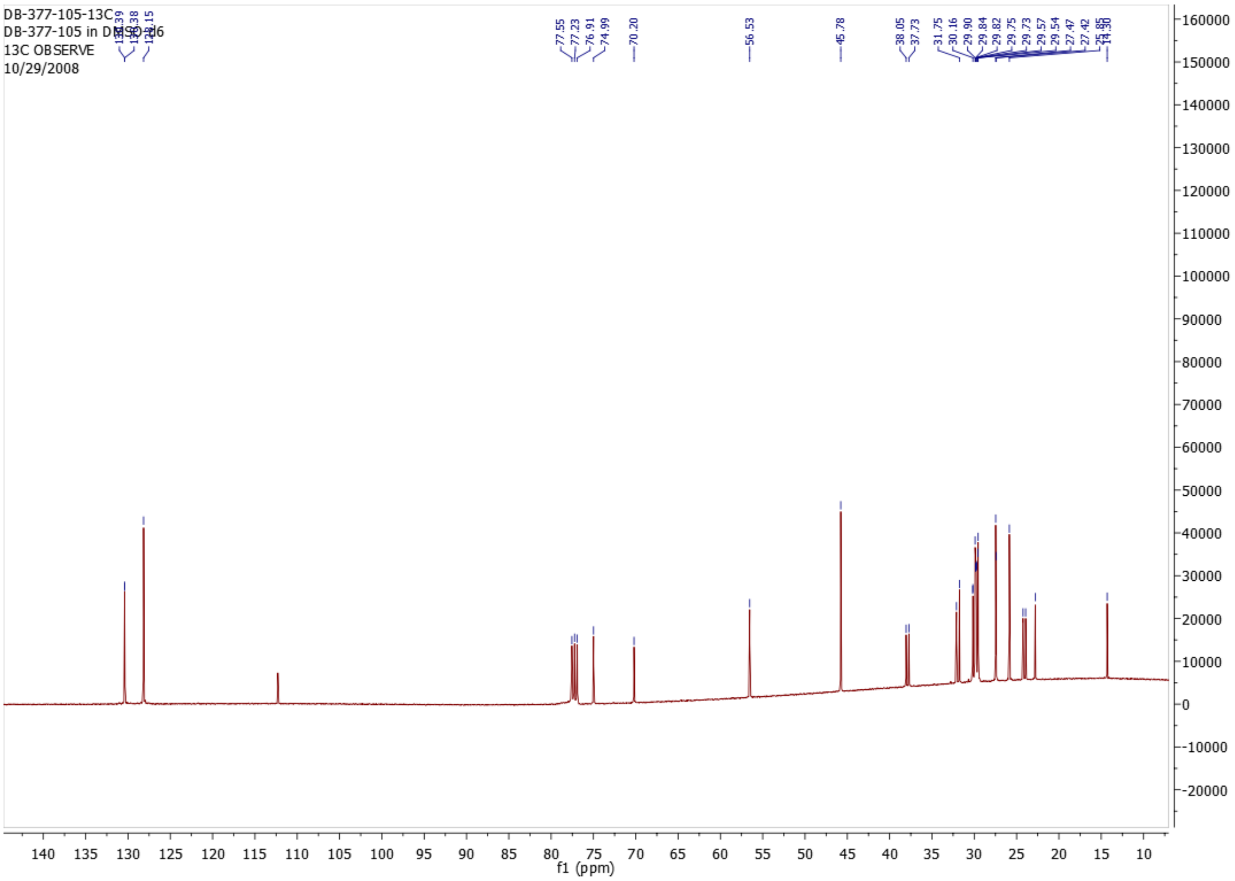

DB-377-105-13C
DB-377-105 in DMSO-d6
13C OBSERVE
10/29/2008
f1 (ppm)

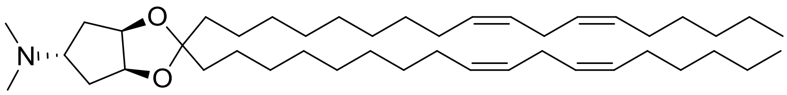
N
O
O

6

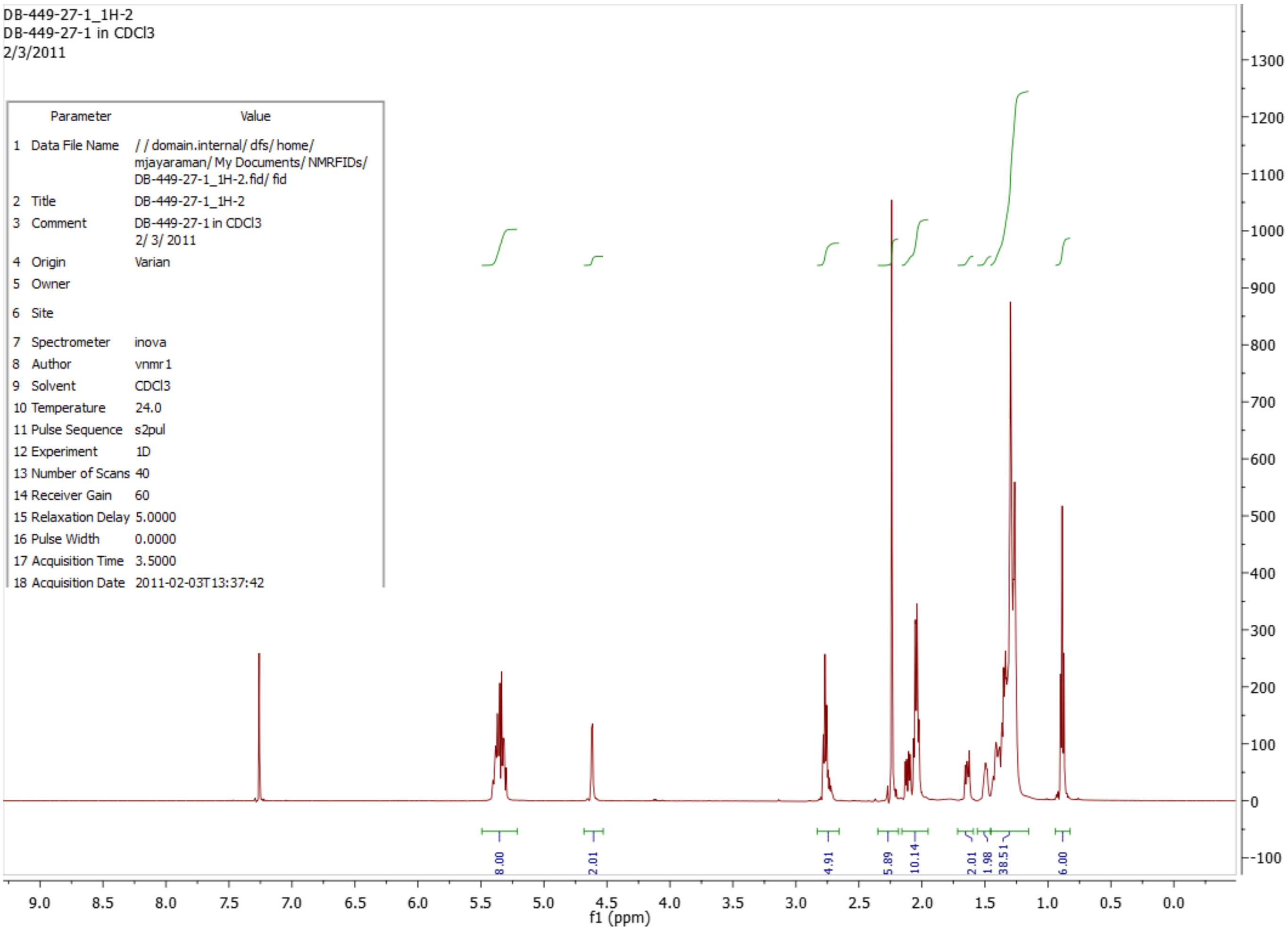
DB-449-27-1_1H-2
DB-449-27-1 in CDCl3
2/3/2011
Parameter
Value
1 Data File Name //domain.internal/dfs/home/mjayaraman/My Documents/NMRFIDs/DB-449-27-1_1H-2.fid/fid
2 Title DB-449-27-1_1H-2
3 Comment DB-449-27-1 in CDCl3 2/3/2011
4 Origin Varian
5 Owner
6 Site
7 Spectrometer inova
8 Author vnmr1
9 Solvent CDCl3
10 Temperature 24.0
11 Pulse Sequence s2pul
12 Experiment 1D
13 Number of Scans 40
14 Receiver Gain 60
15 Relaxation Delay 5.0000
16 Pulse Width 0.0000
17 Acquisition Time 3.5000
18 Acquisition Date 2011-02-03T13:37:42
8.00
2.01
4.91
5.89
10.14
2.01
1.98
38.51
6.00
9.0
8.5
8.0
7.5
7.0
6.5
6.0
5.5
5.0
4.5
4.0
3.5
3.0
2.5
2.0
1.5
1.0
0.5
0.0
f1 (ppm)
1300
1200
1100
1000
900
800
700
600
500
400
300
200
100
0
-100

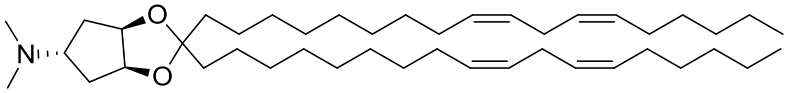

6

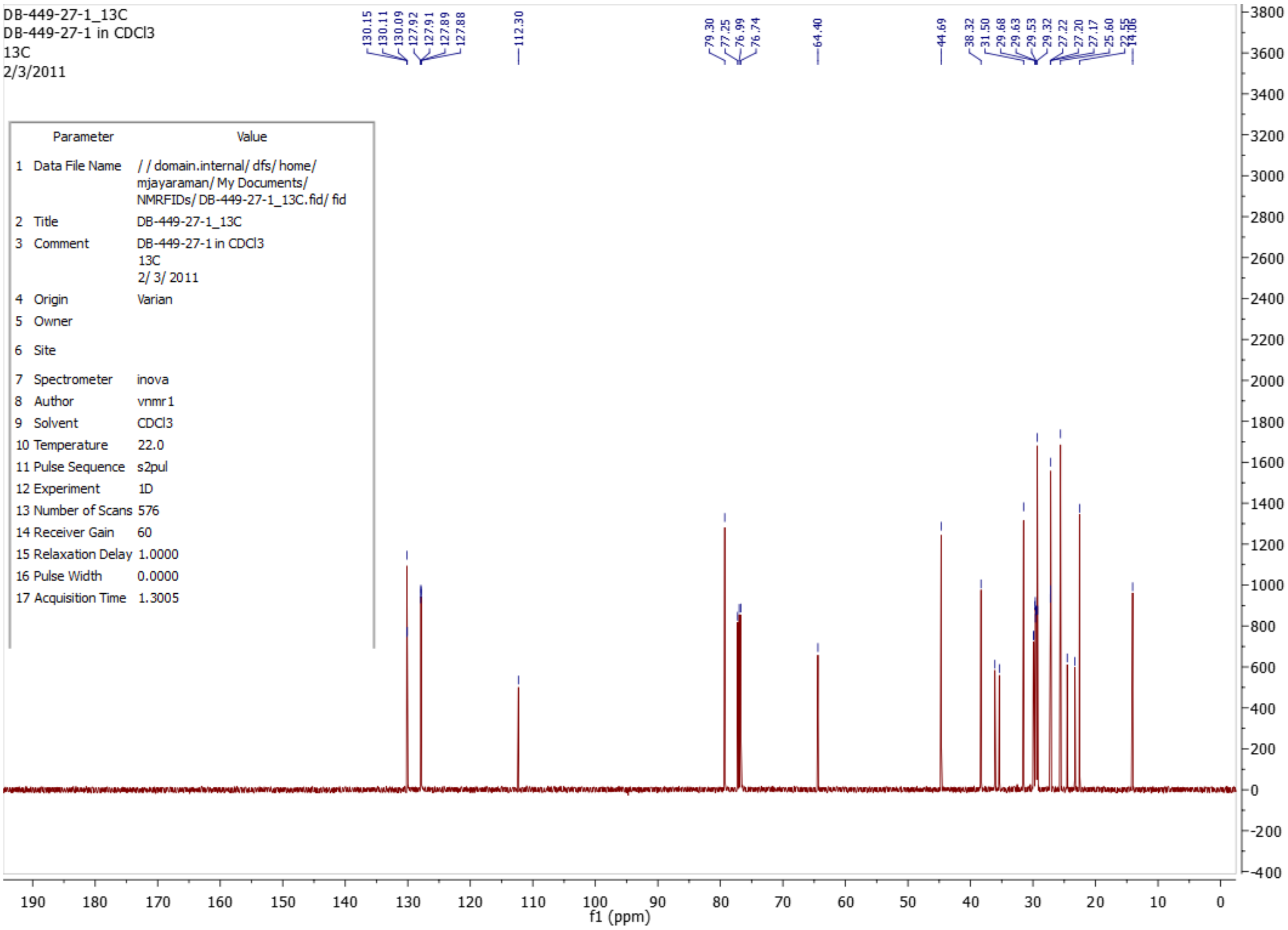

DB-449-27-1_13C
DB-449-27-1 in CDCl3
13C
2/3/2011
130.15
130.11
130.09
127.92
127.91
127.89
127.88
112.30
79.30
77.25
76.99
76.74
64.40
44.69
38.32
31.50
29.68
29.63
29.53
29.32
27.22
27.20
27.17
25.60
22.55
14.06
Parameter
Value
1 Data File Name
//domain.internal/dfs/home/mjayaraman/My Documents/NMRFIDs/DB-449-27-1_13C.fid/fid
2 Title
DB-449-27-1_13C
3 Comment
DB-449-27-1 in CDCl3
13C
2/3/2011
4 Origin
Varian
5 Owner
6 Site
7 Spectrometer
inova
8 Author
vnmr1
9 Solvent
CDCl3
10 Temperature
22.0
11 Pulse Sequence
s2pul
12 Experiment
1D
13 Number of Scans
576
14 Receiver Gain
60
15 Relaxation Delay
1.0000
16 Pulse Width
0.0000
17 Acquisition Time
1.3005
f1 (ppm)

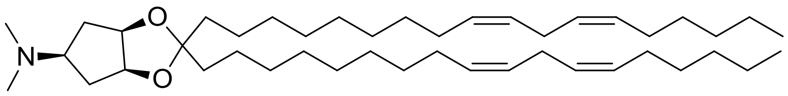

7

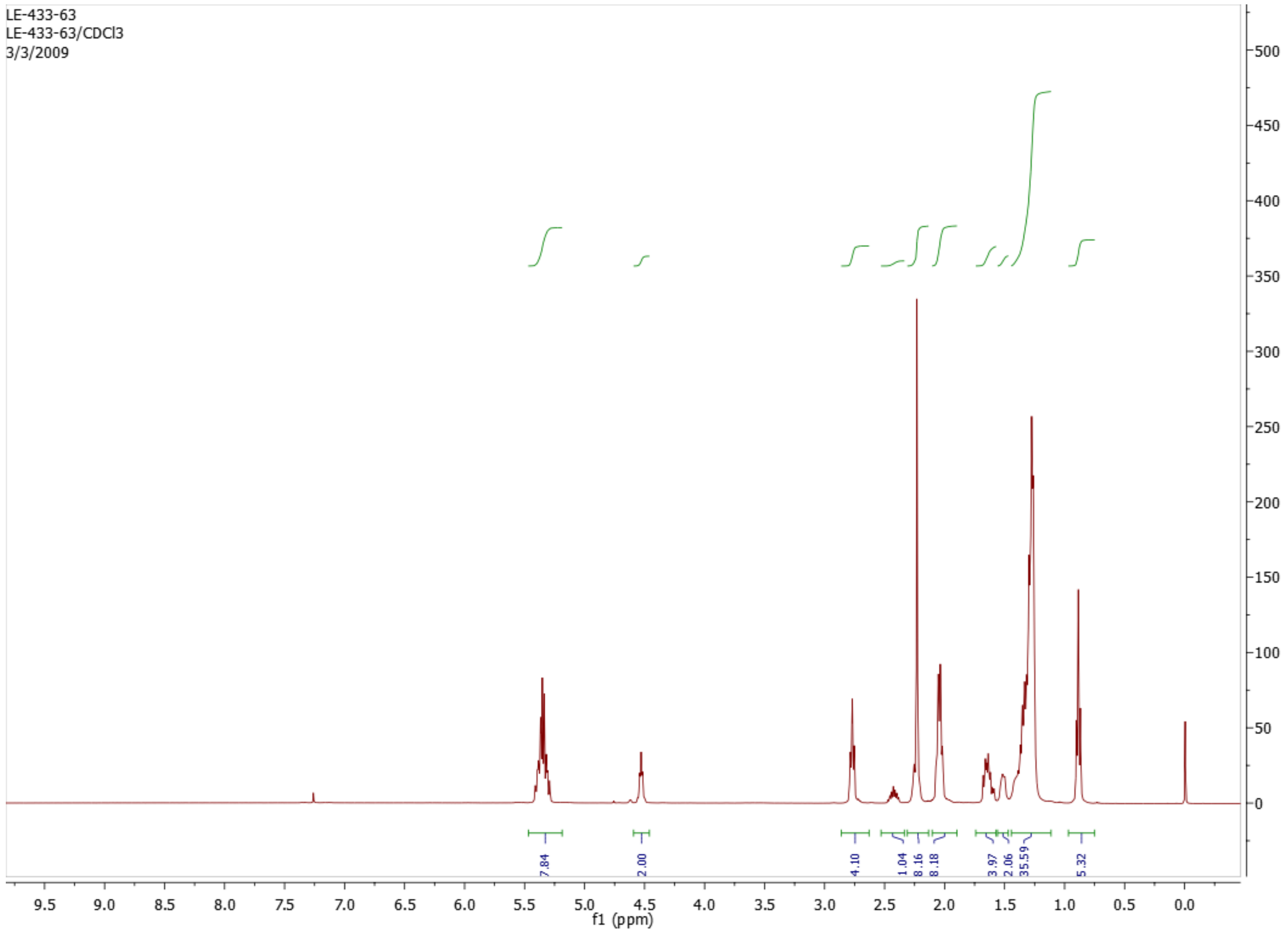

LE-433-63
LE-433-63/CDCl3
3/3/2009
7.84
2.00
4.10
1.04
8.16
8.18
3.97
2.06
35.59
5.32
f1 (ppm)

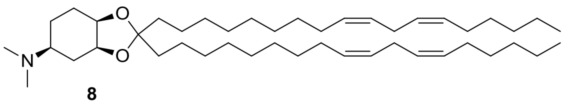

8

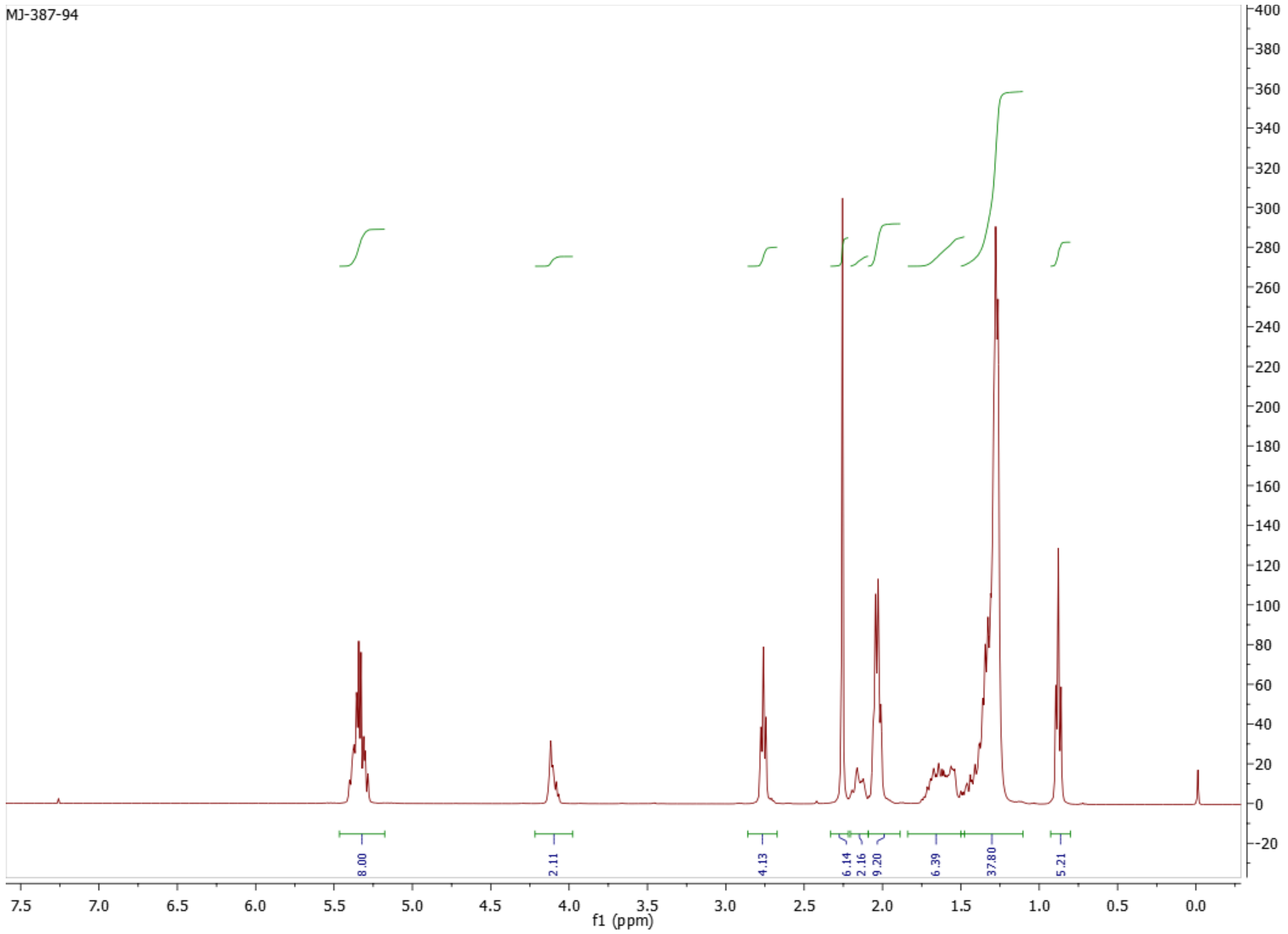

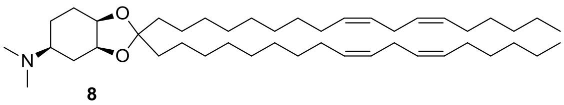

8

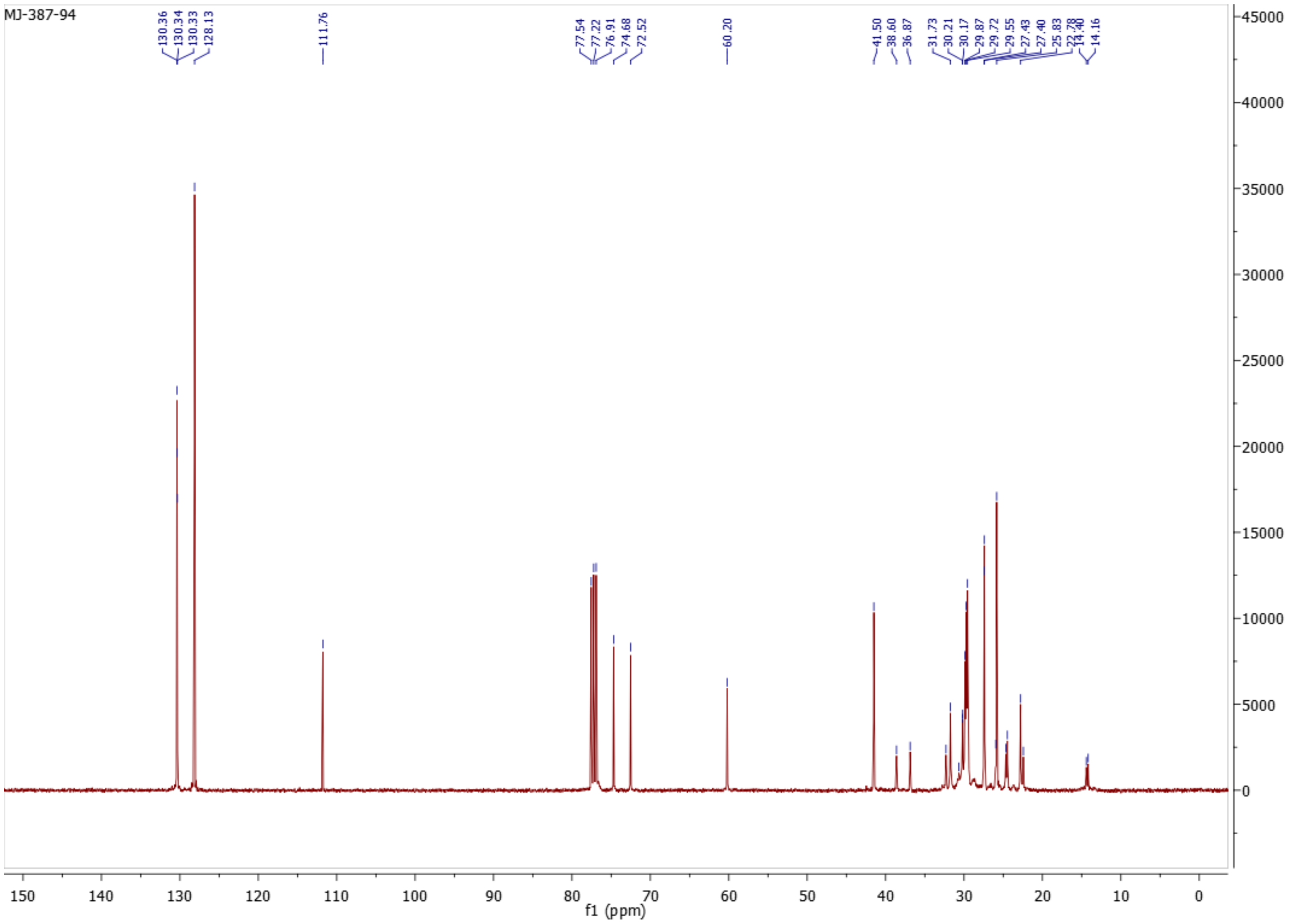
MJ-387-94
130.36
130.34
130.33
128.13
111.76
77.54
77.22
76.91
74.68
72.52
60.20
41.50
38.60
36.87
31.73
30.21
30.17
29.87
29.72
29.55
27.43
27.40
25.83
14.16
45000
40000
35000
30000
25000
20000
15000
10000
5000
0
150
140
130
120
110
100
90
80
70
60
50
40
30
20
10
0
f1 (ppm)

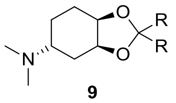
R
R
O
O
N

R = linoleyl

9

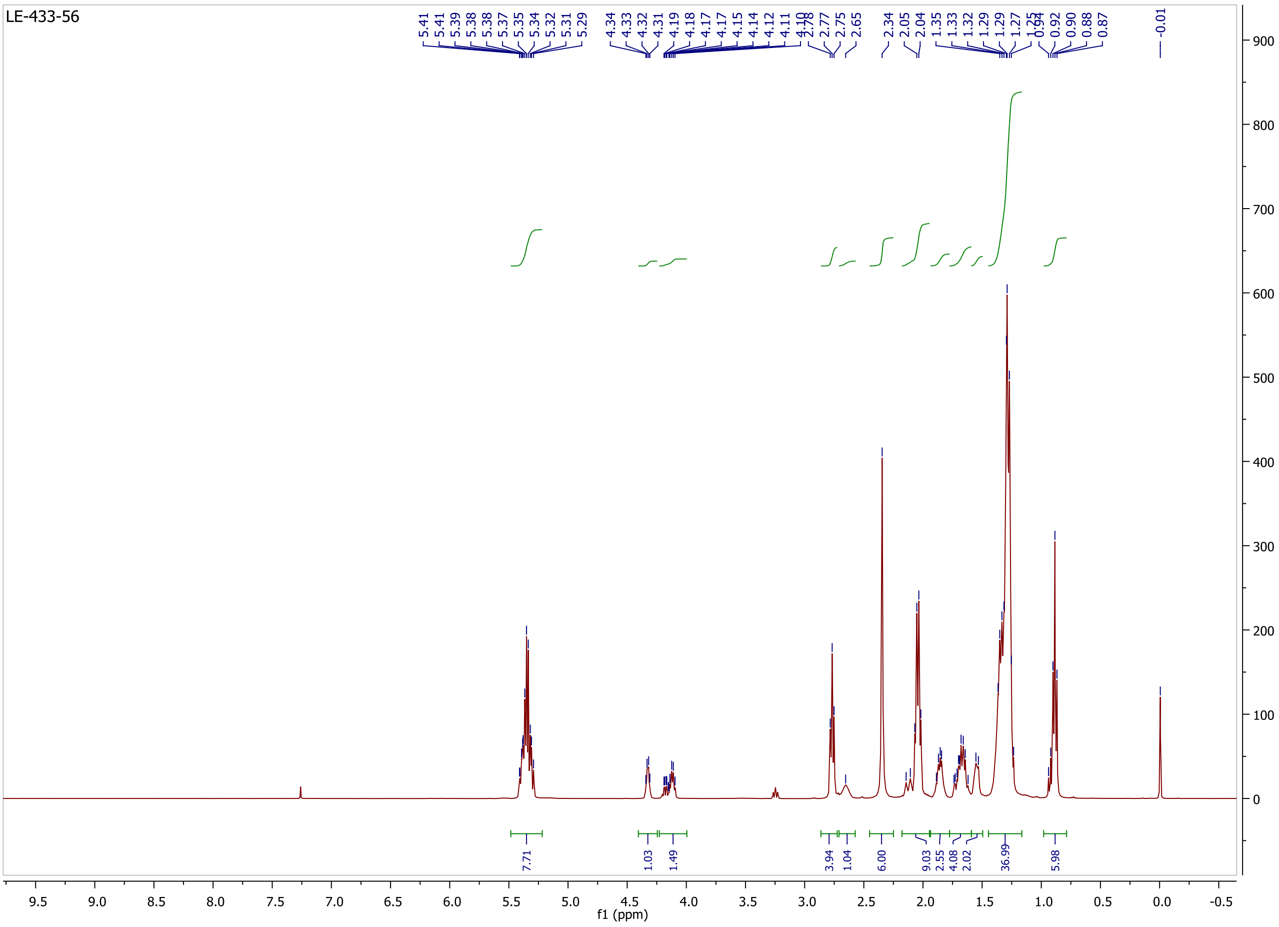
LE-433-56
5.41 5.41 5.39 5.38 5.38 5.37 5.35 5.34 5.32 5.31 5.29 4.34 4.33 4.32 4.31 4.19 4.18 4.17 4.17 4.15 4.14 4.12 4.11 4.10 2.78 2.77 2.75 2.65 2.34 2.05 2.04 1.35 1.33 1.32 1.29 1.29 1.27 1.25 0.94 0.92 0.90 0.88 0.87 -0.01
7.71
1.03
1.49
3.94
1.04
6.00
9.03
2.55
4.08
2.02
36.99
5.98
9.5 9.0 8.5 8.0 7.5 7.0 6.5 6.0 5.5 5.0 4.5 4.0 3.5 3.0 2.5 2.0 1.5 1.0 0.5 0.0 -0.5
f1 (ppm)
900 800 700 600 500 400 300 200 100 0

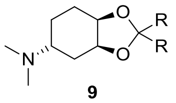

R = linoleyl

9

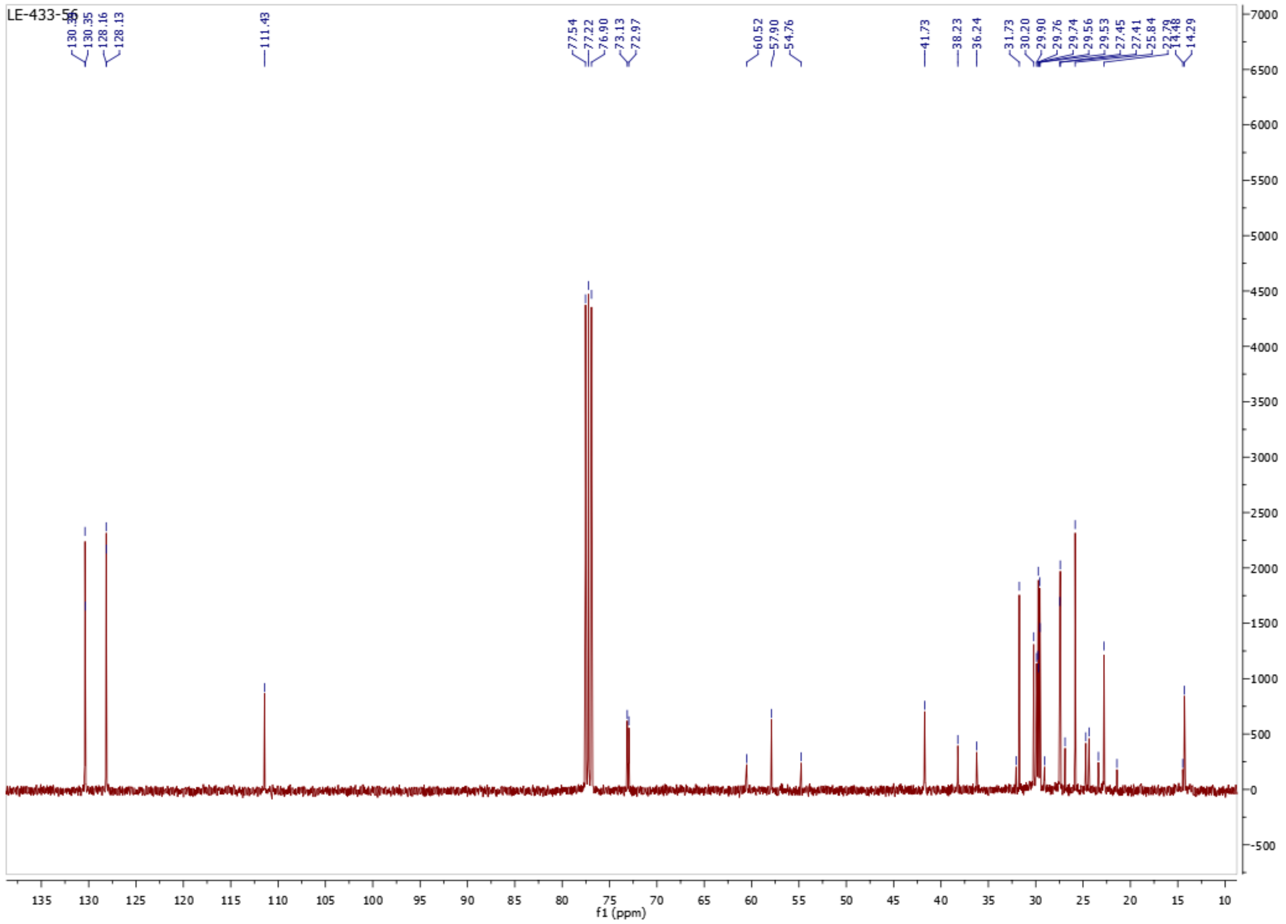
LE-433-56
130.35
128.16
128.13
111.43
77.54
77.22
76.90
73.13
72.97
60.52
57.90
54.76
41.73
38.23
36.24
31.73
30.20
29.90
29.76
29.74
29.56
29.53
27.45
27.41
25.84
22.79
14.48
14.29
f1 (ppm)

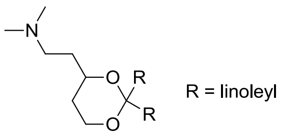
N
O
O
R
R
R = linoleyl

10

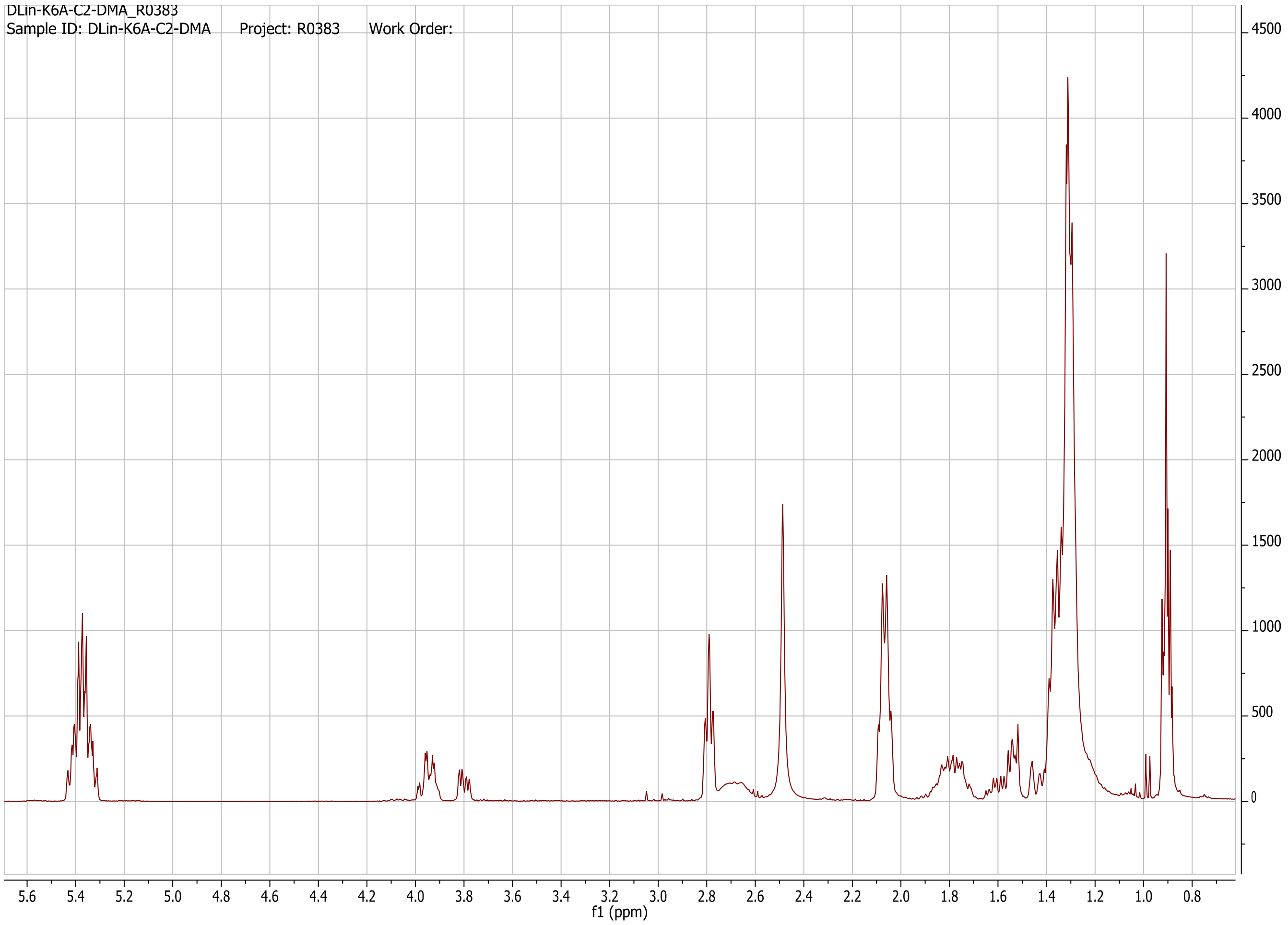
DLin-K6A-C2-DMA_R0383
Sample ID: DLin-K6A-C2-DMA
Project: R0383
Work Order:
f1 (ppm)

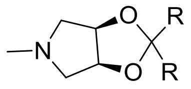

R = linoleyl

11

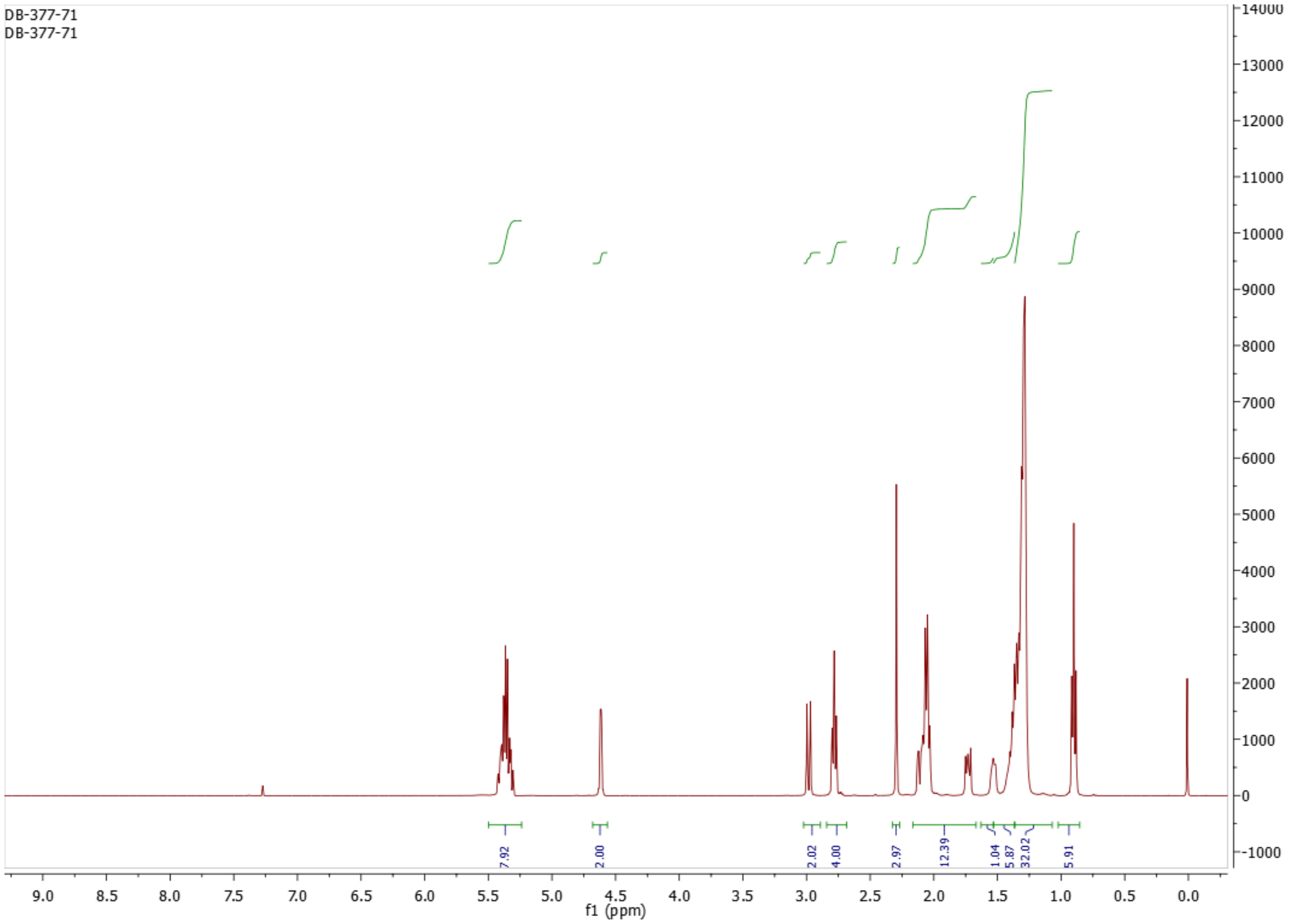

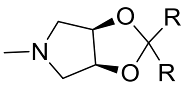

R = linoleyl

11

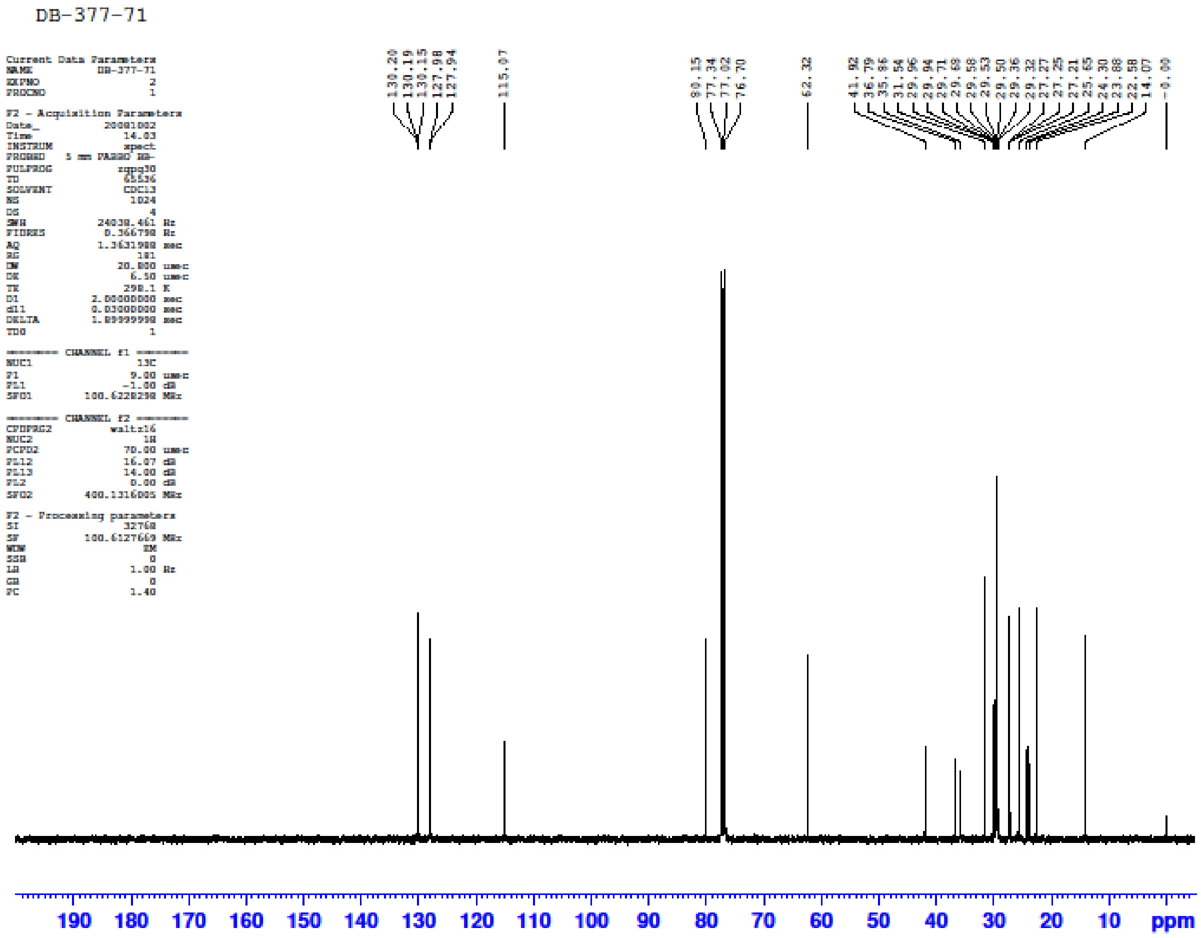

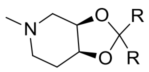

R = linoleyl

12

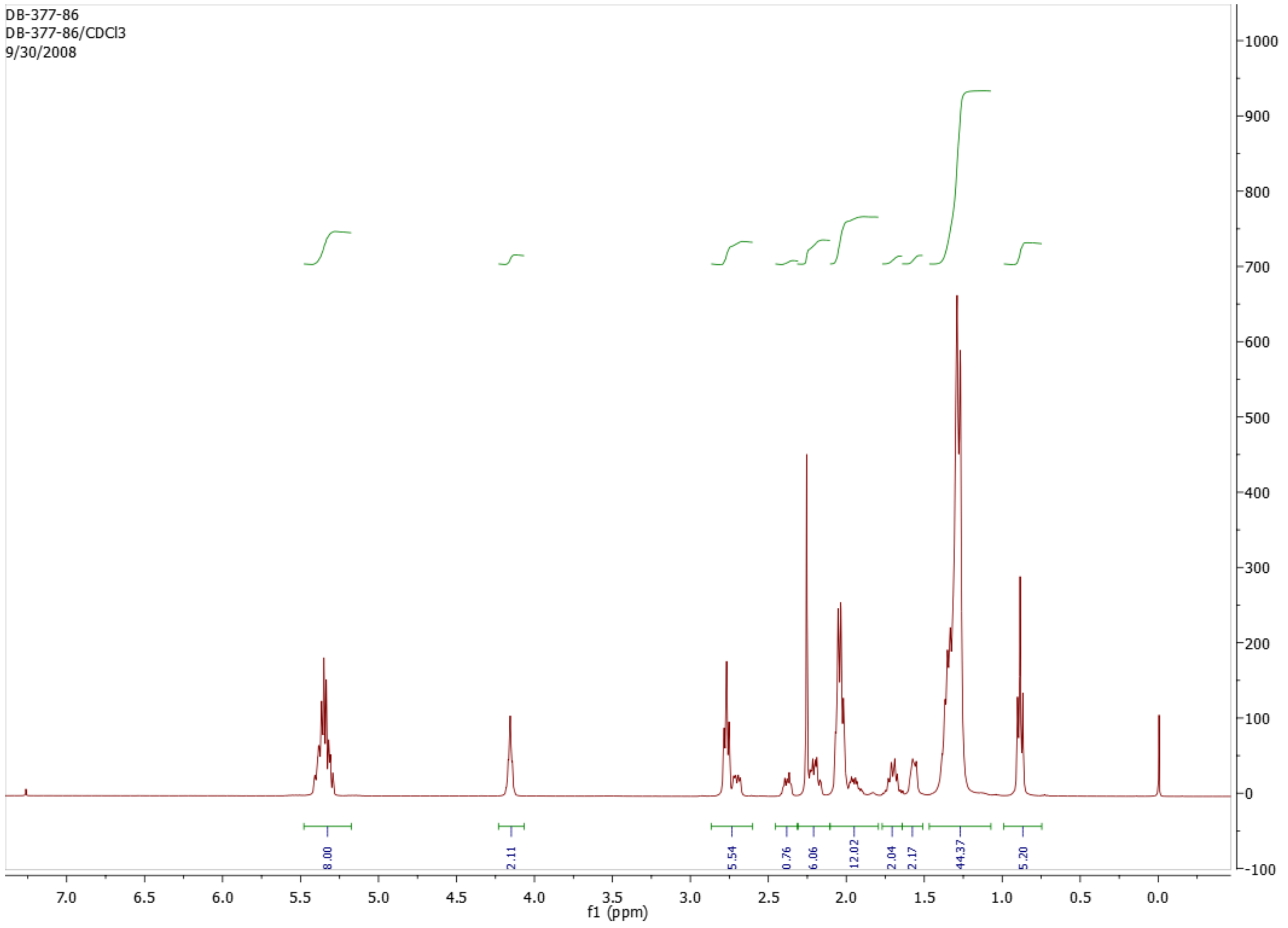

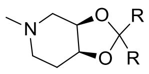

R = linoleyl

12

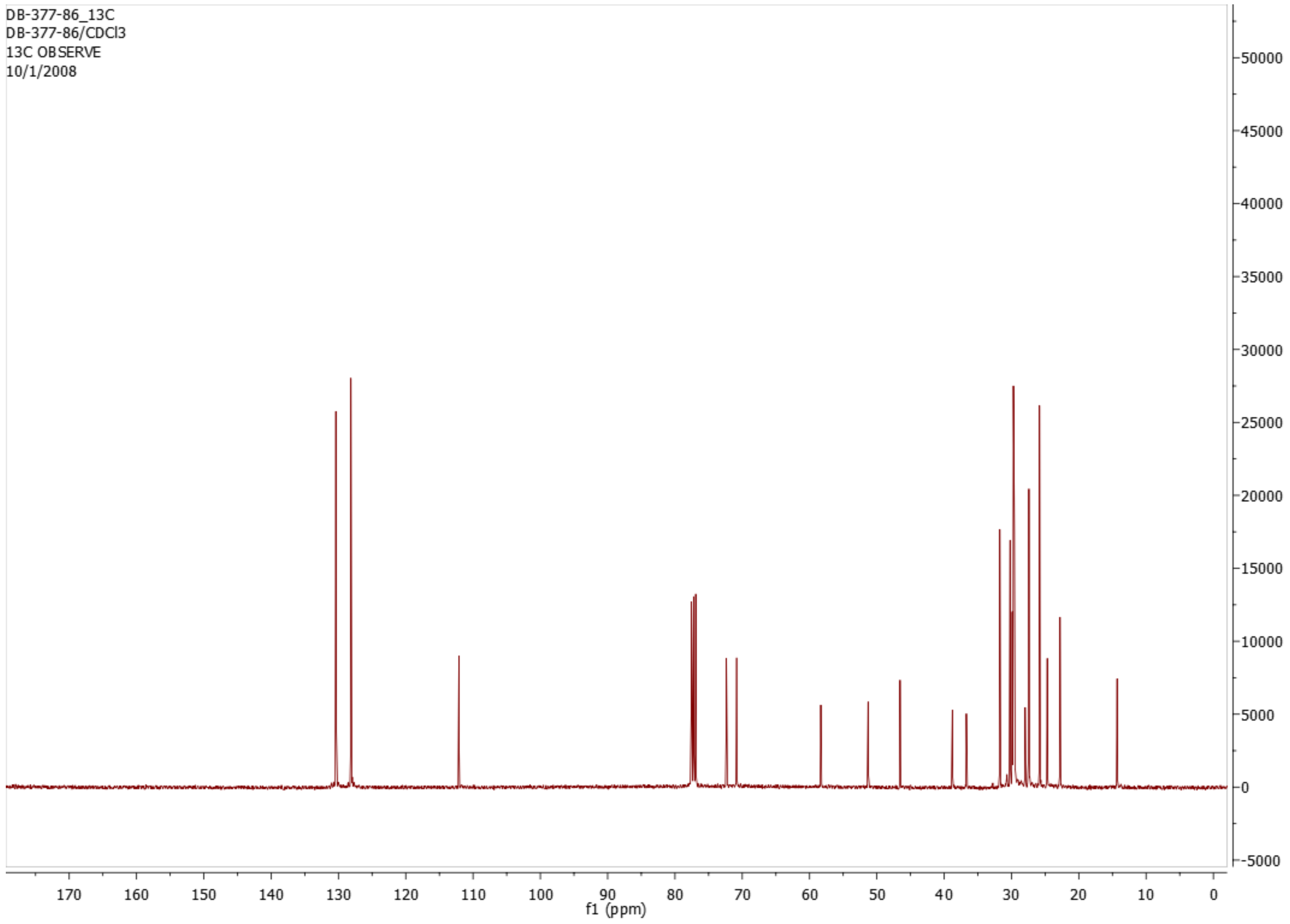

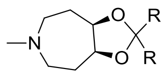

R = linoleyl

13

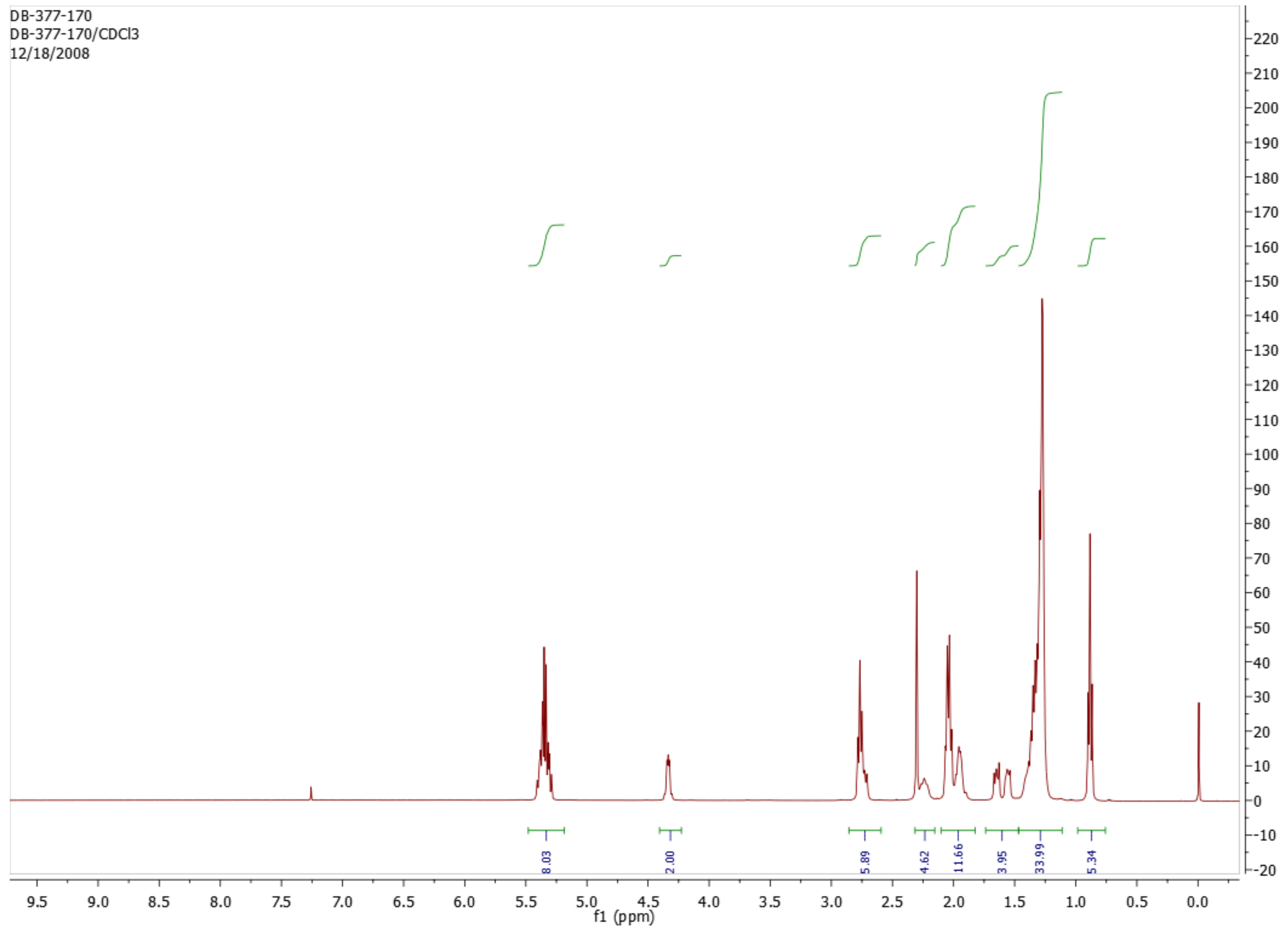

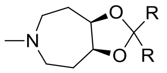

R = linoleyl

13

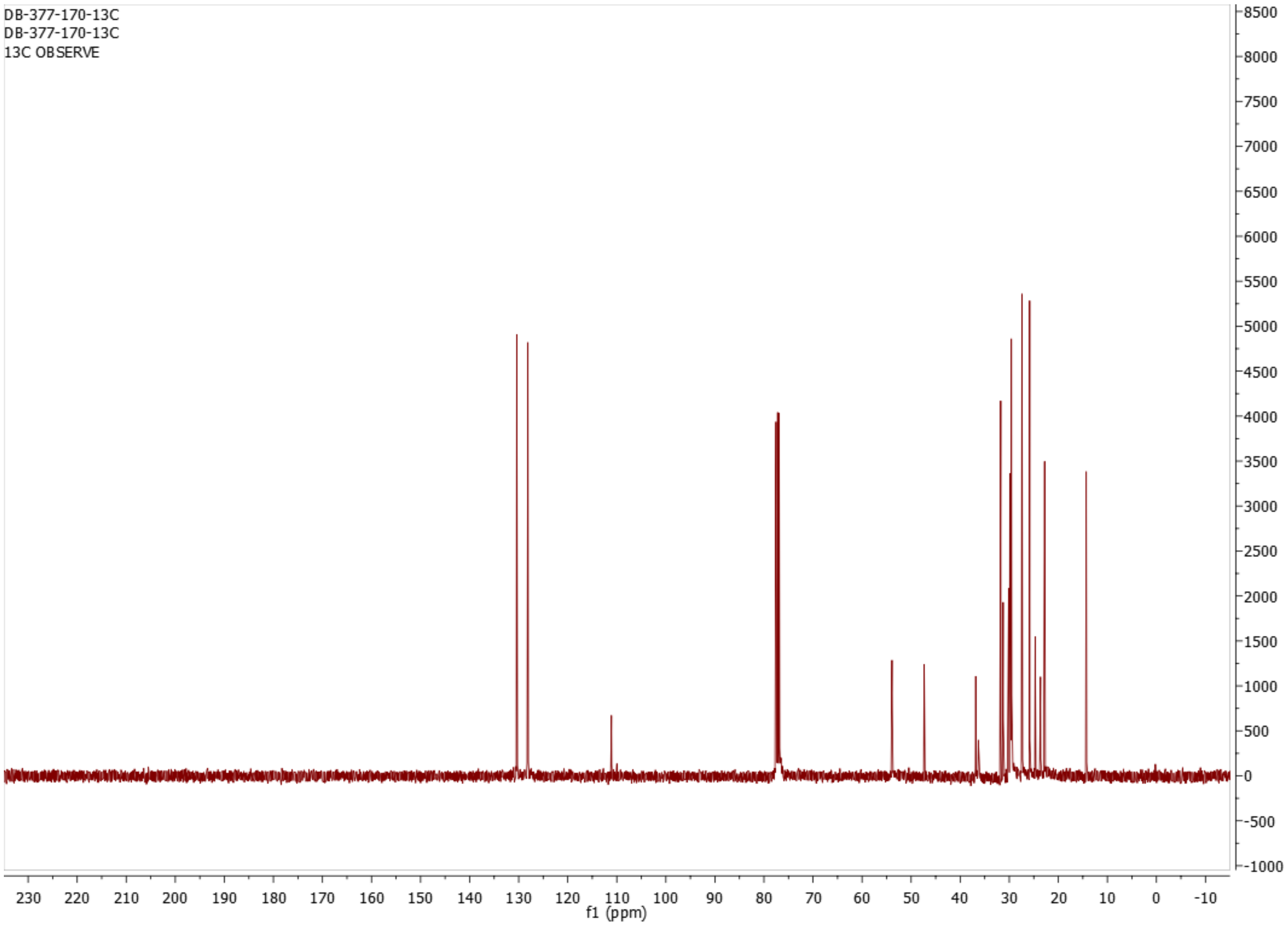

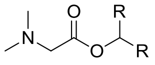

R = linoleyl

14

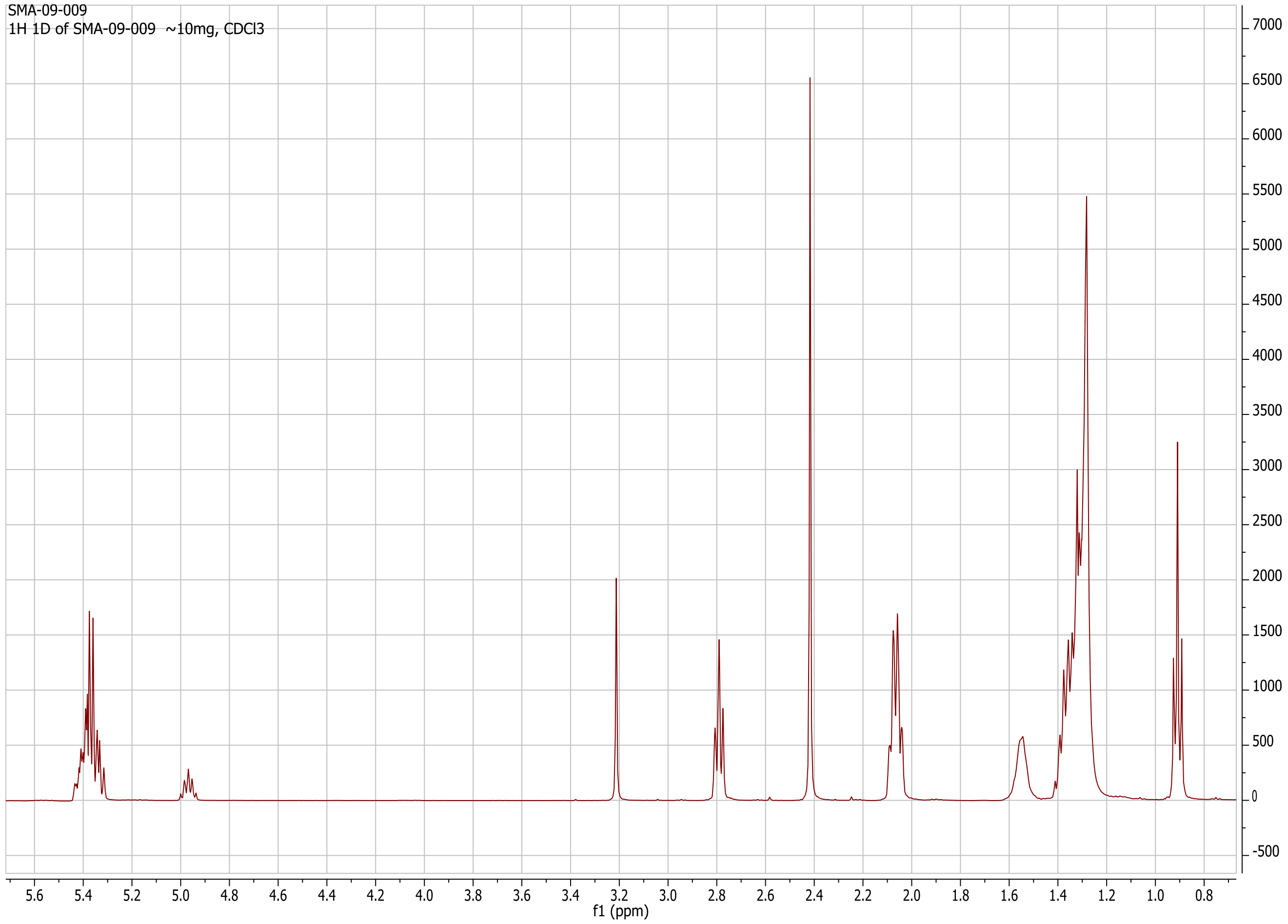

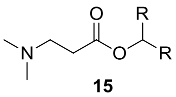

R = linoleyl

15

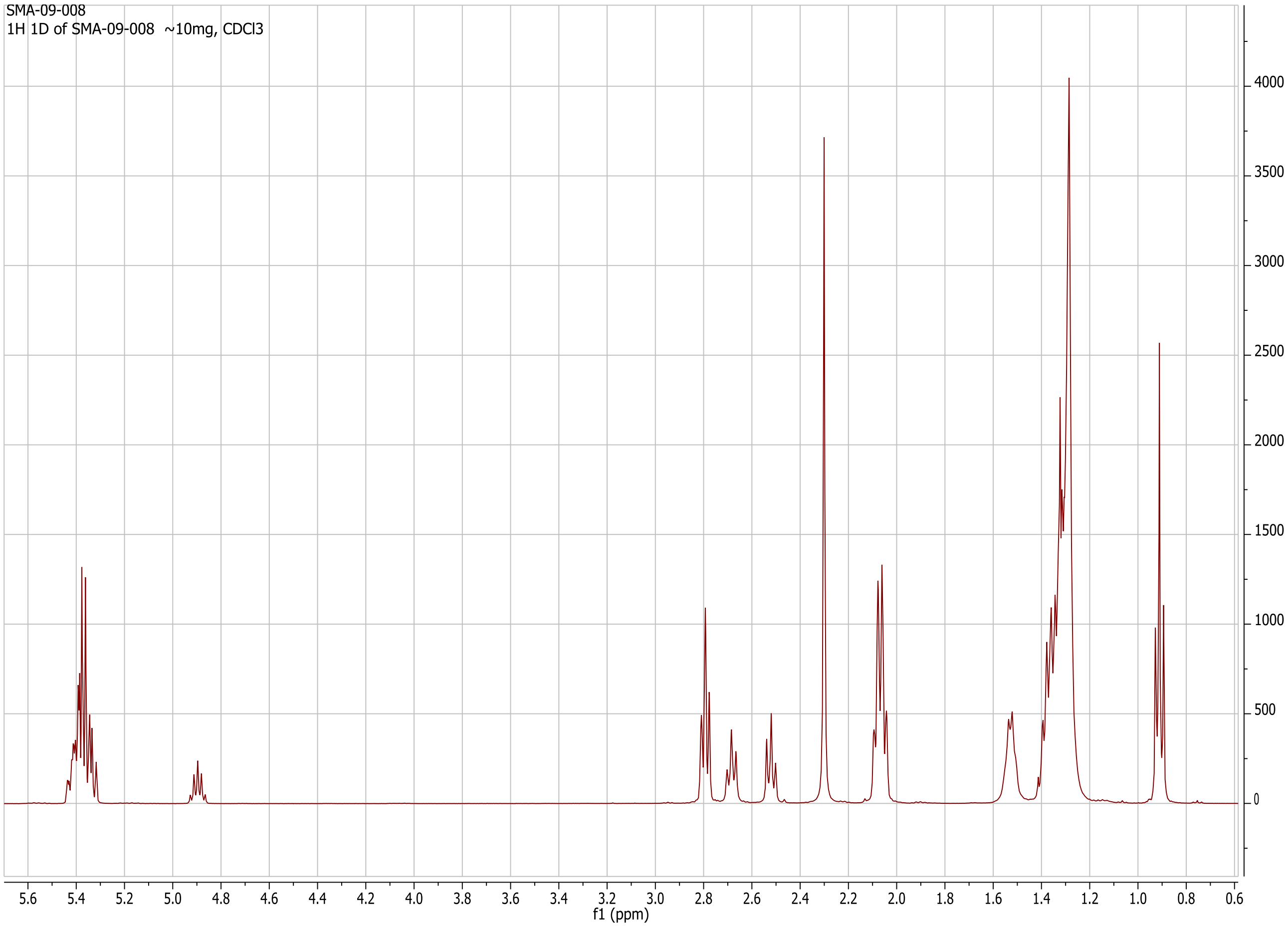

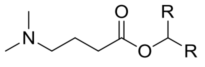

R = linoleyl

16

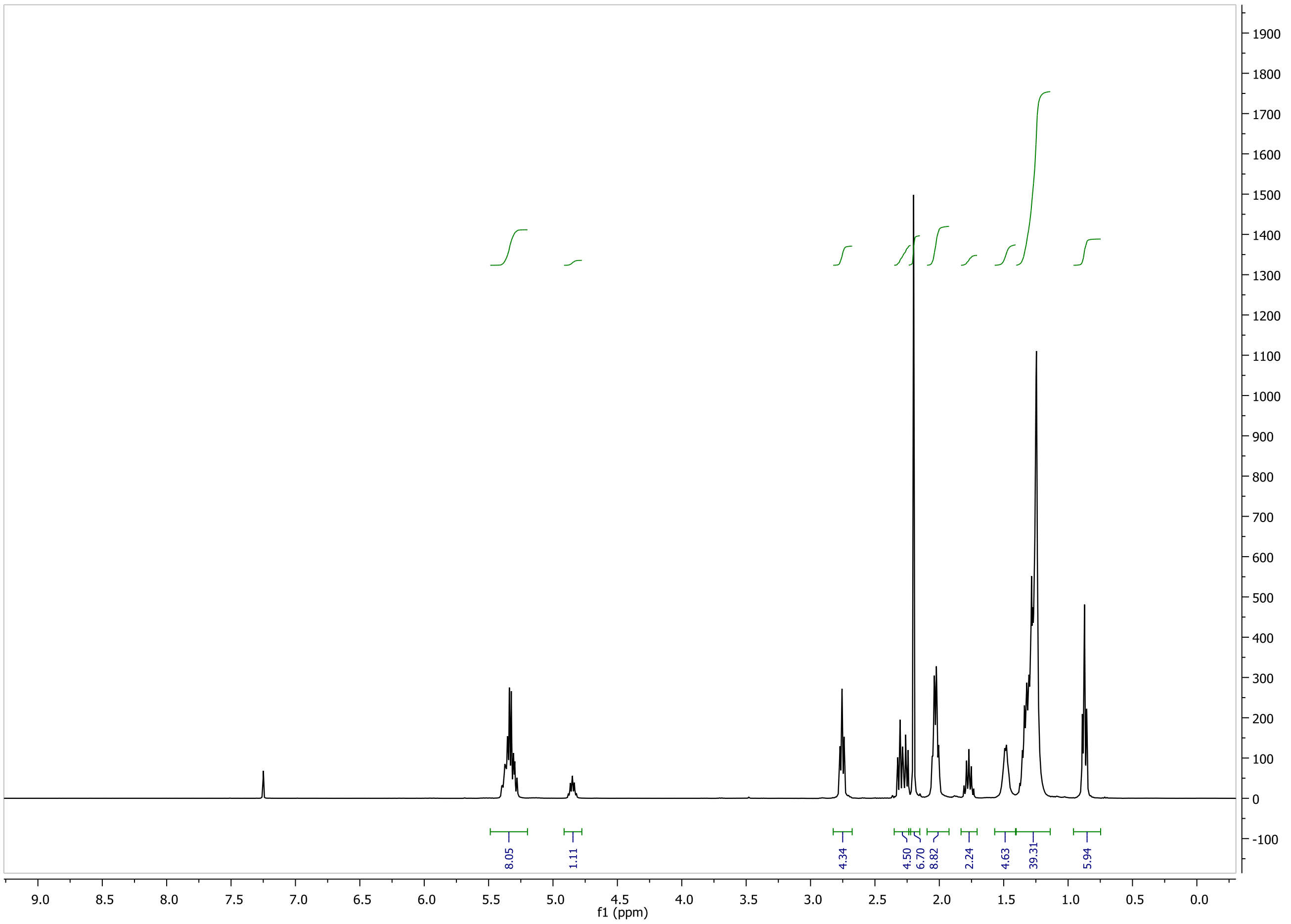

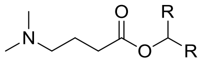

16

R = linoleyl

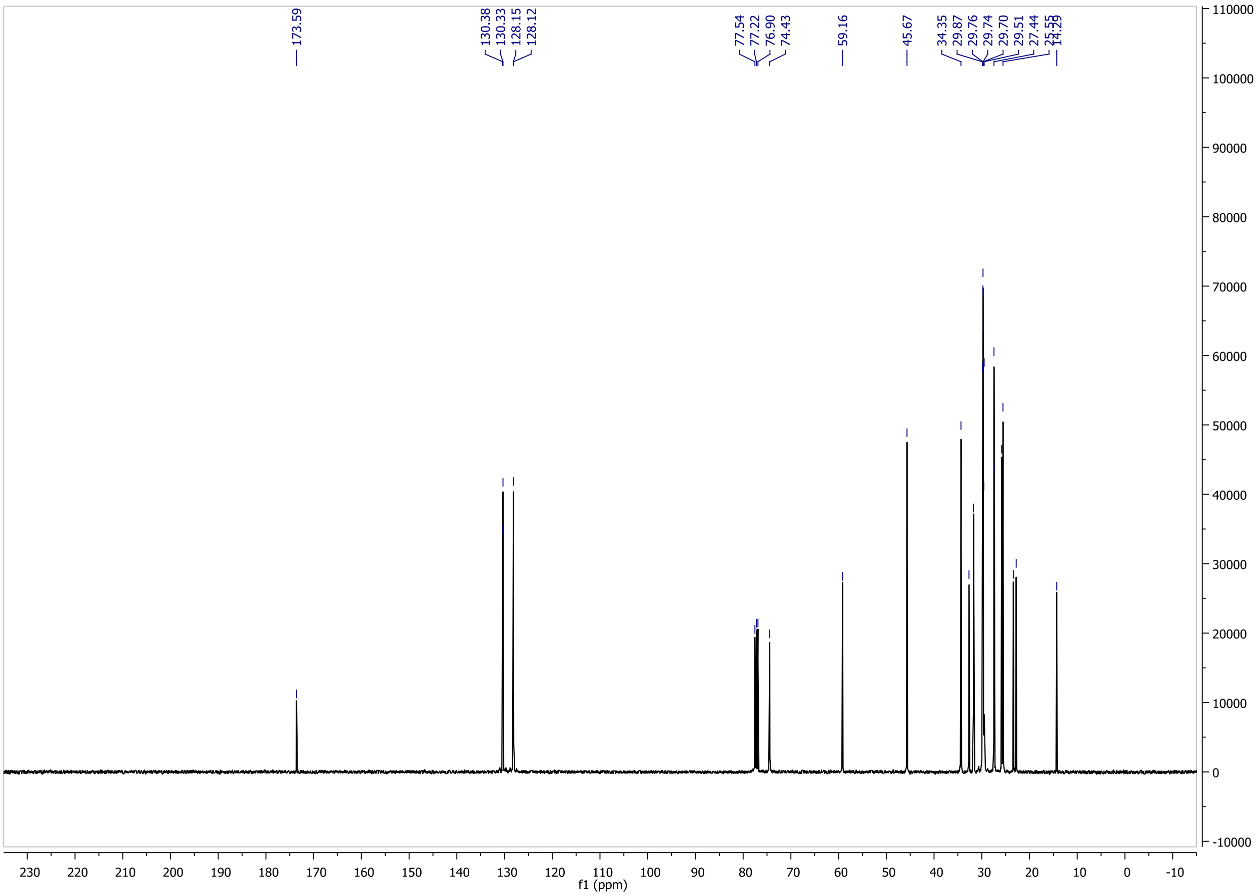

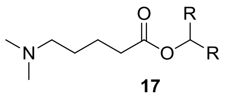

17

R = linoleyl

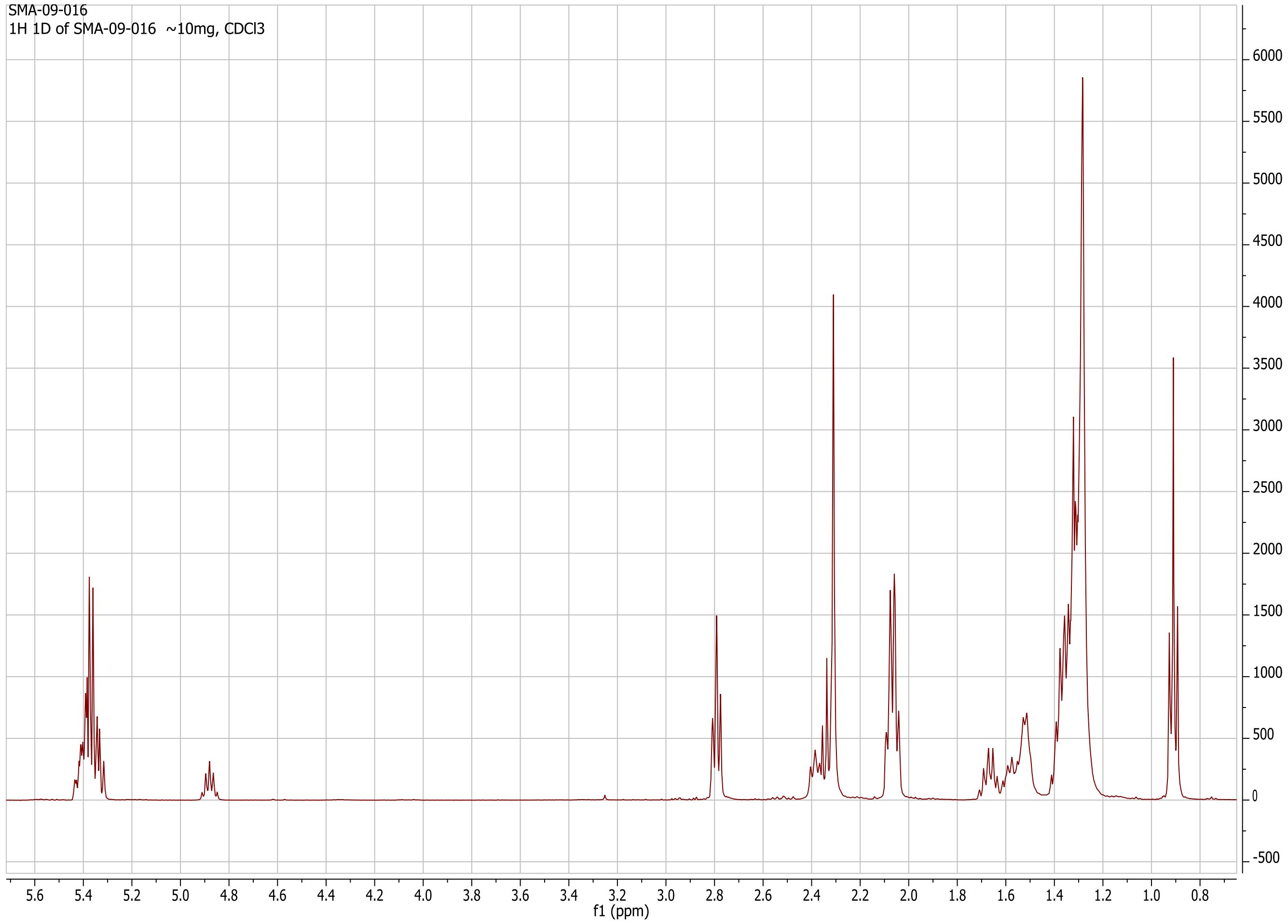

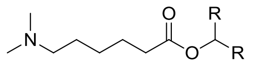

R = linoleyl

18

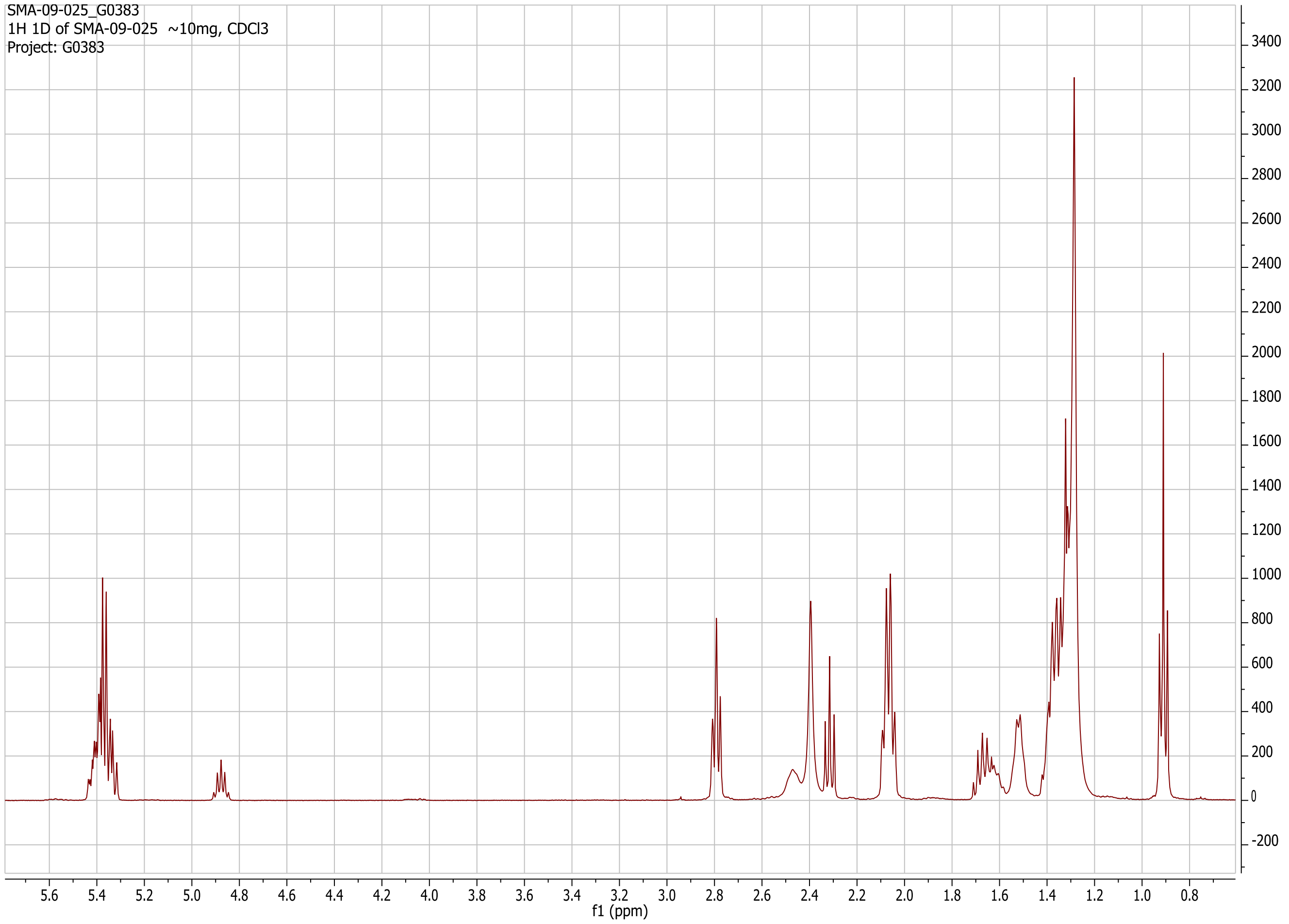

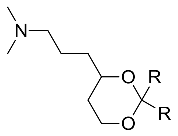

R = linoleyl

19

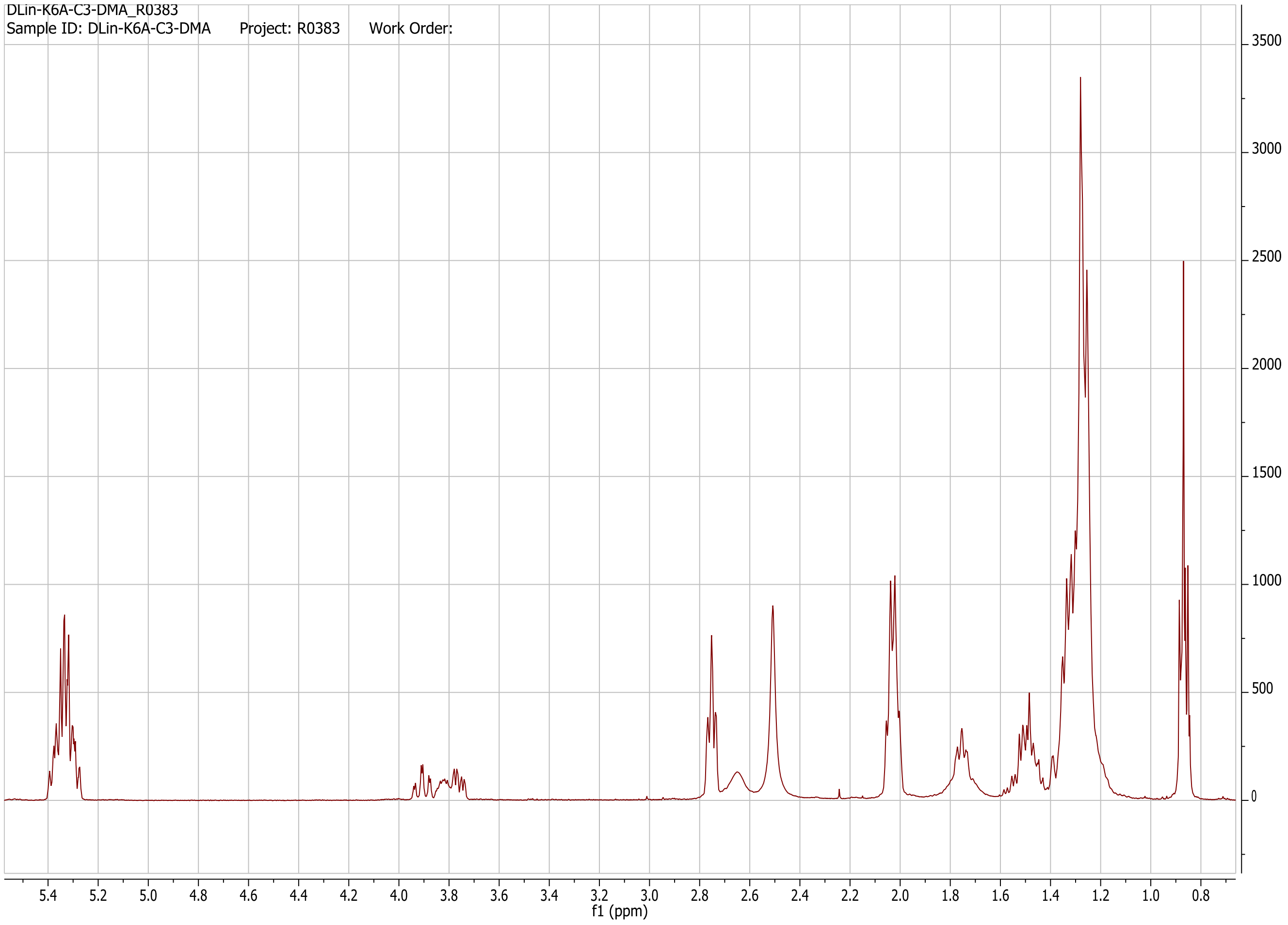

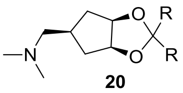

20

R = linoleyl

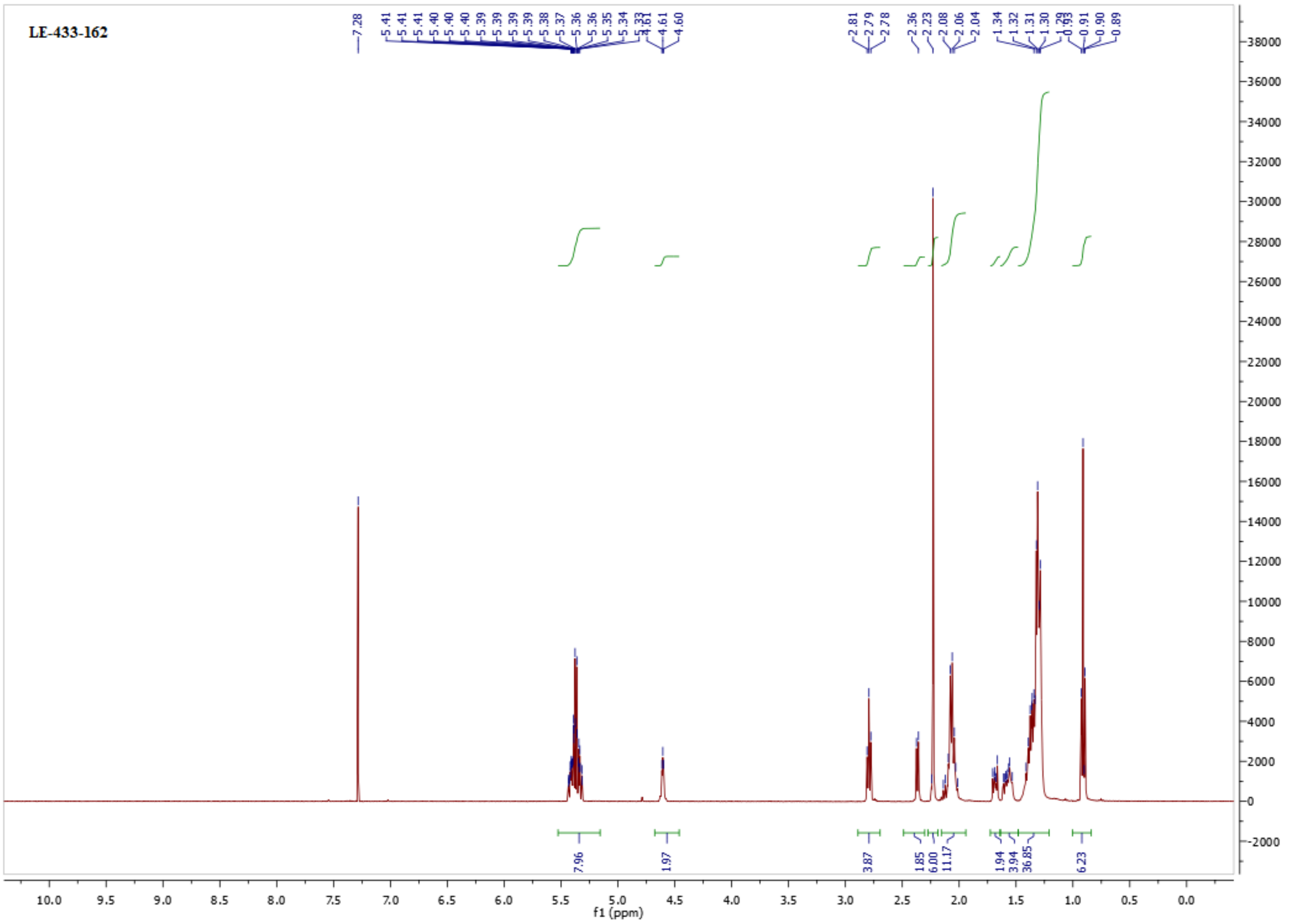

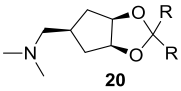

20

R = linoleyl

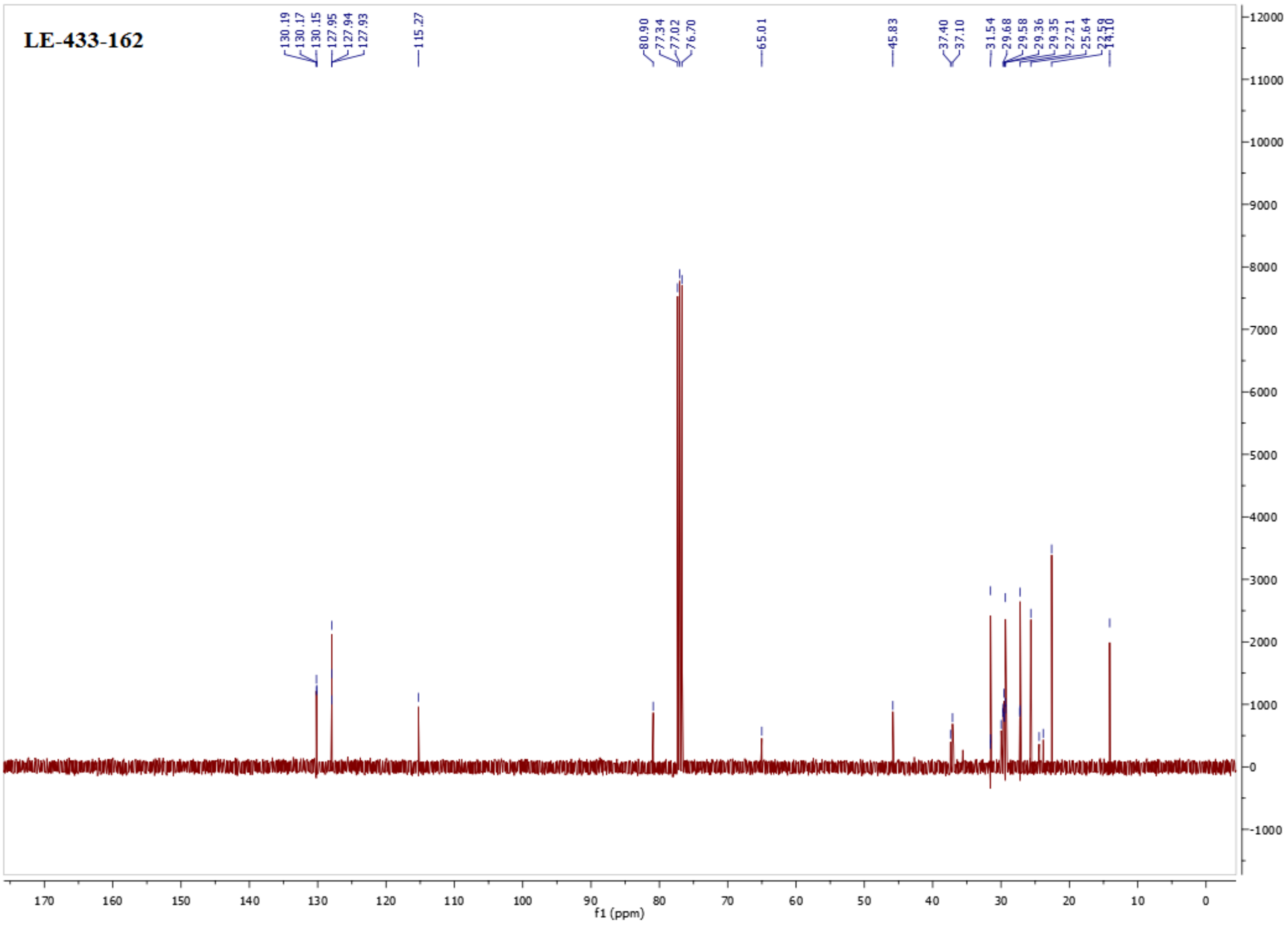

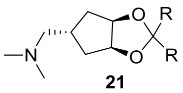

21

R = linoleyl

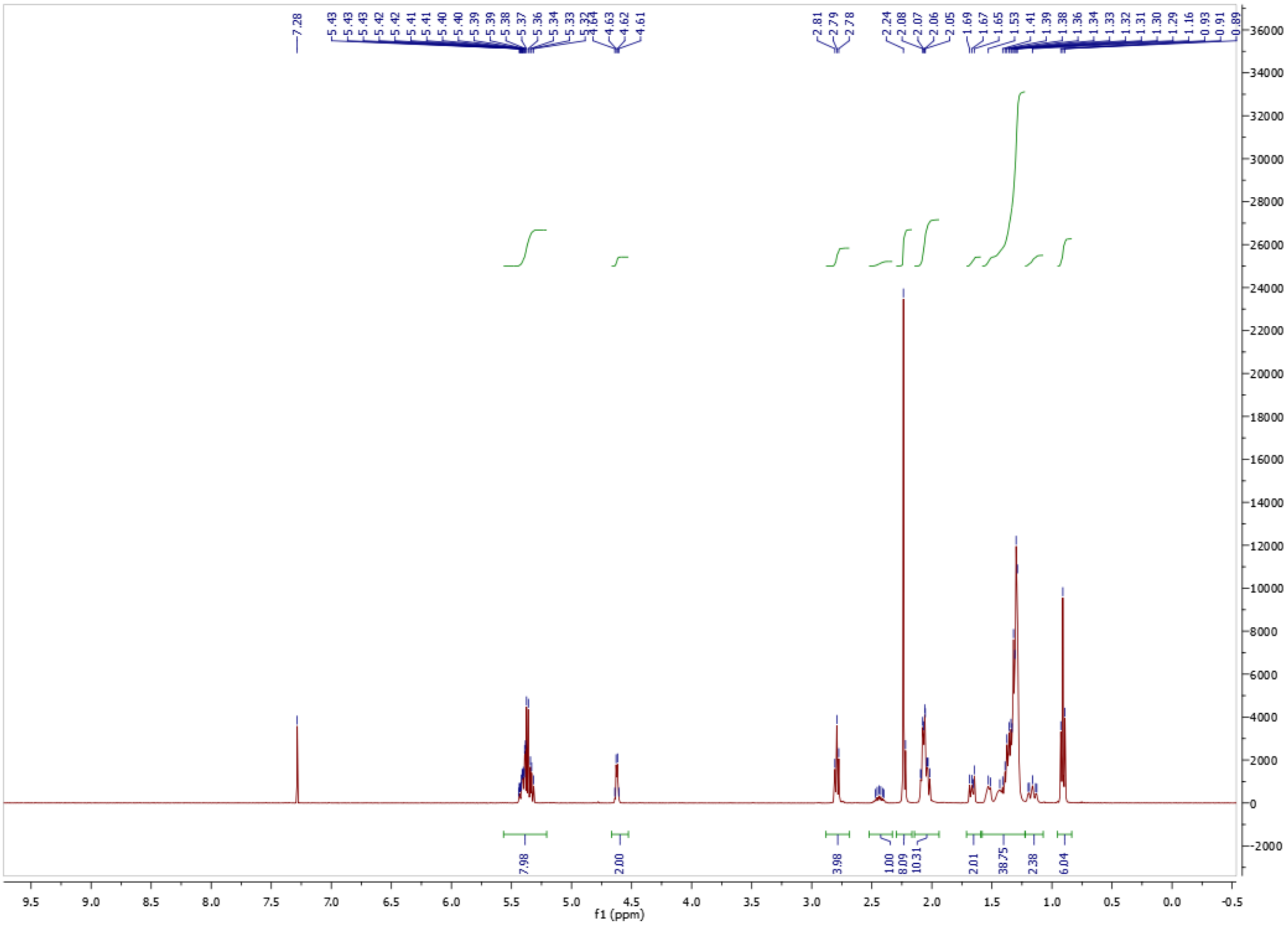

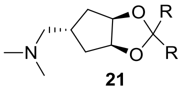

21

R = linoleyl

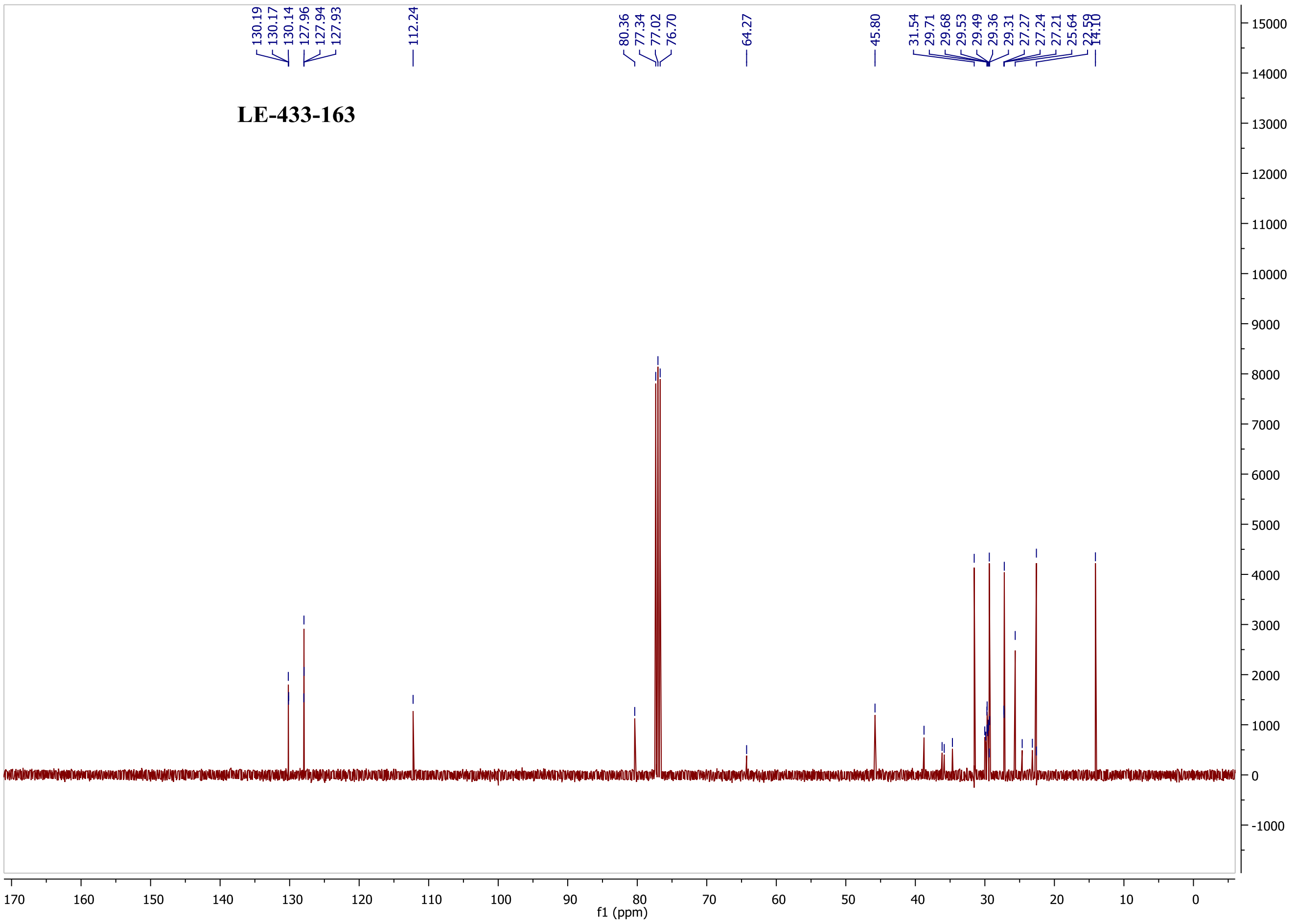

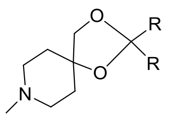

22

R = linoleyl

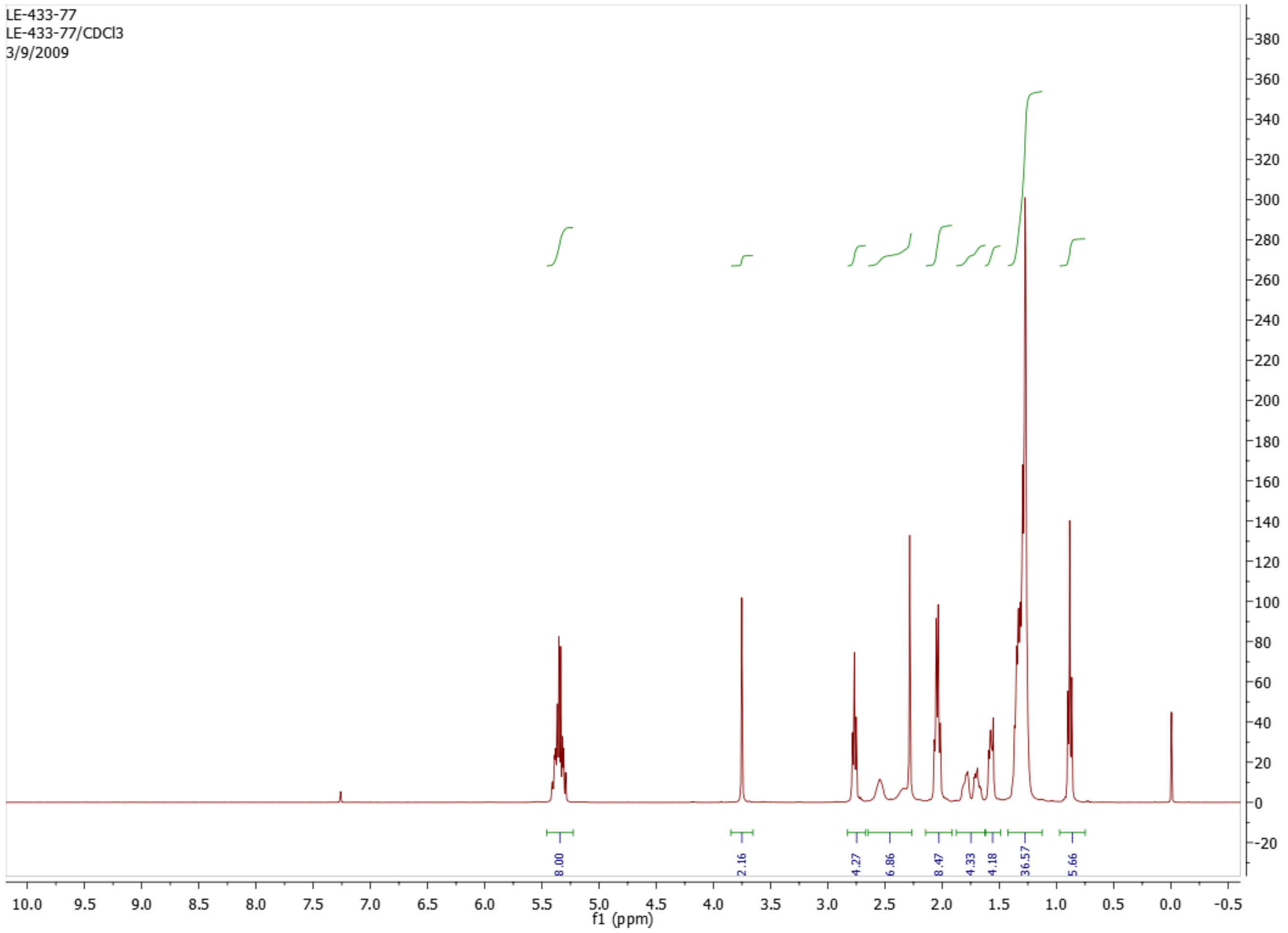

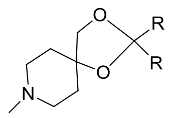

22

R = linoleyl

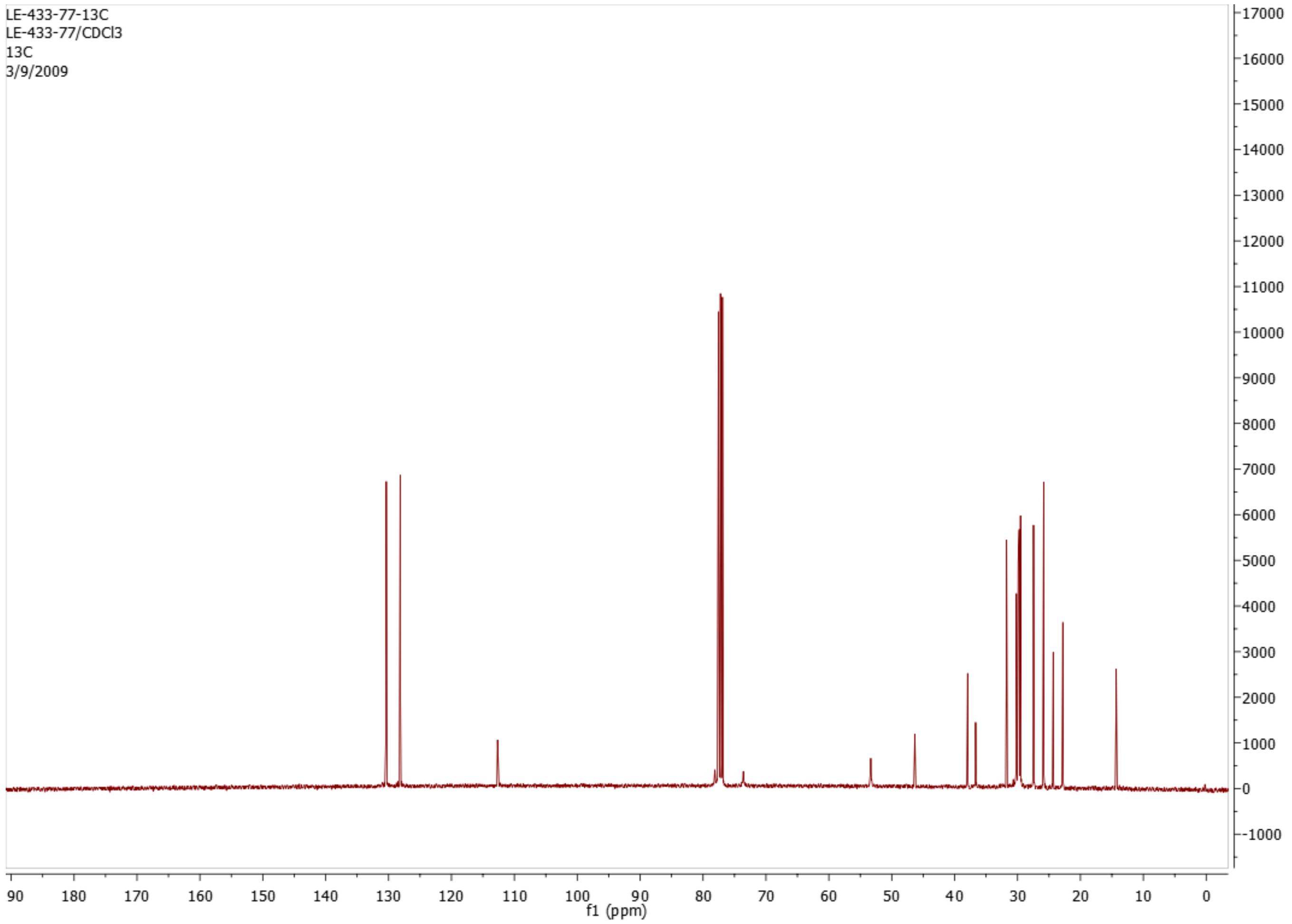

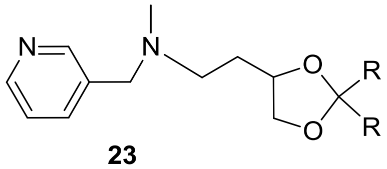

23

R = linoleyl

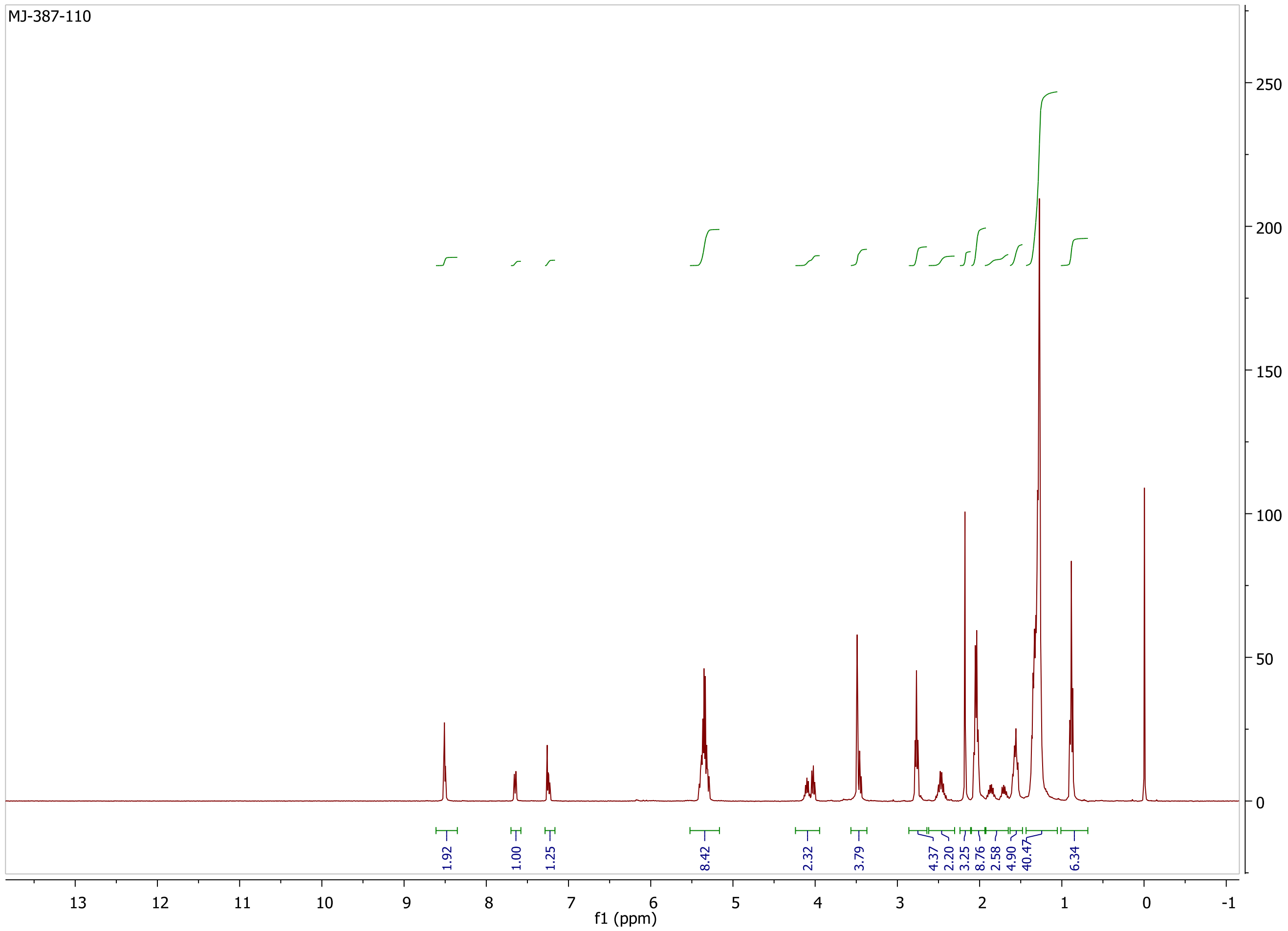

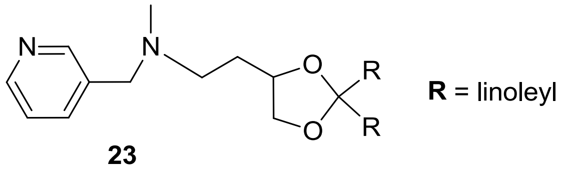
N
N
O
O
R
R
23
R = linoleyl

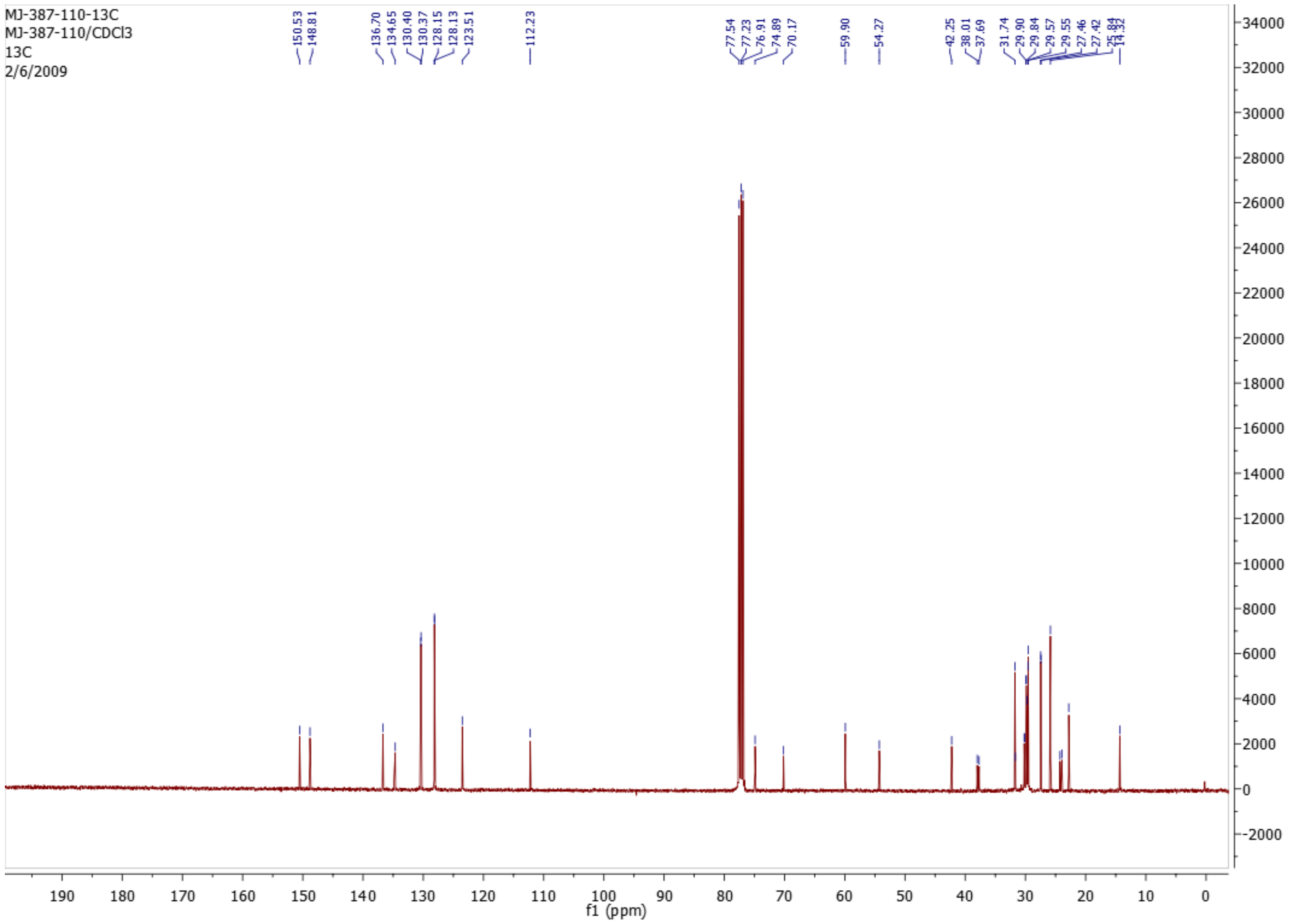
MJ-387-110-13C
MJ-387-110/CDCl3
13C
2/6/2009
150.53
148.81
136.70
134.65
130.40
130.37
128.15
128.13
123.51
112.23
77.54
77.23
76.91
74.89
70.17
59.90
54.27
42.25
38.01
37.69
31.74
29.90
29.84
29.57
29.55
27.46
27.42
f1 (ppm)

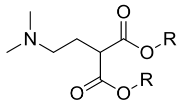

24

R = linoleyl

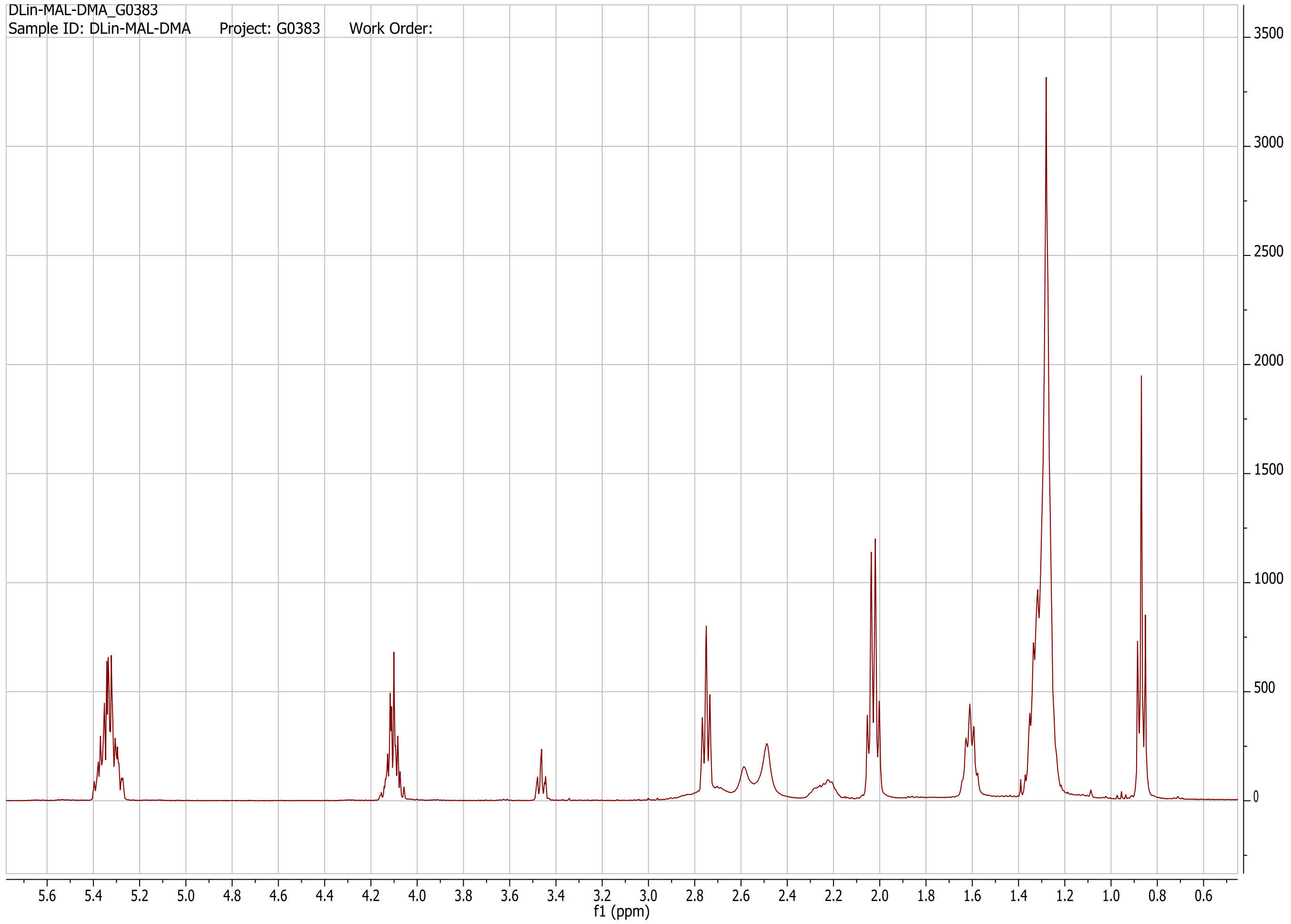

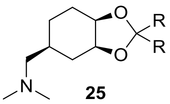

25

R = linoleyl

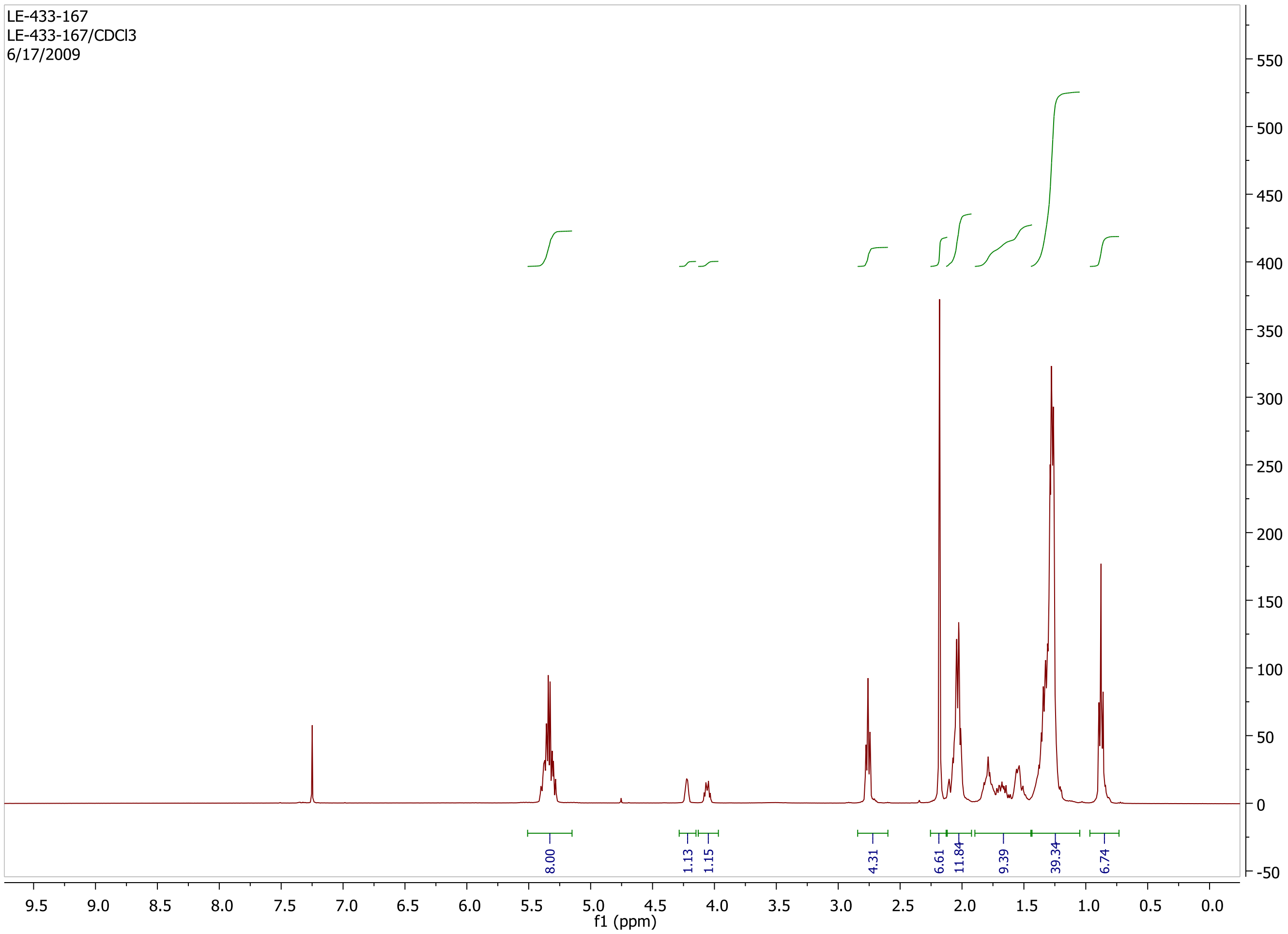

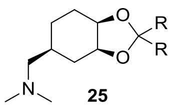

25

R = linoleyl

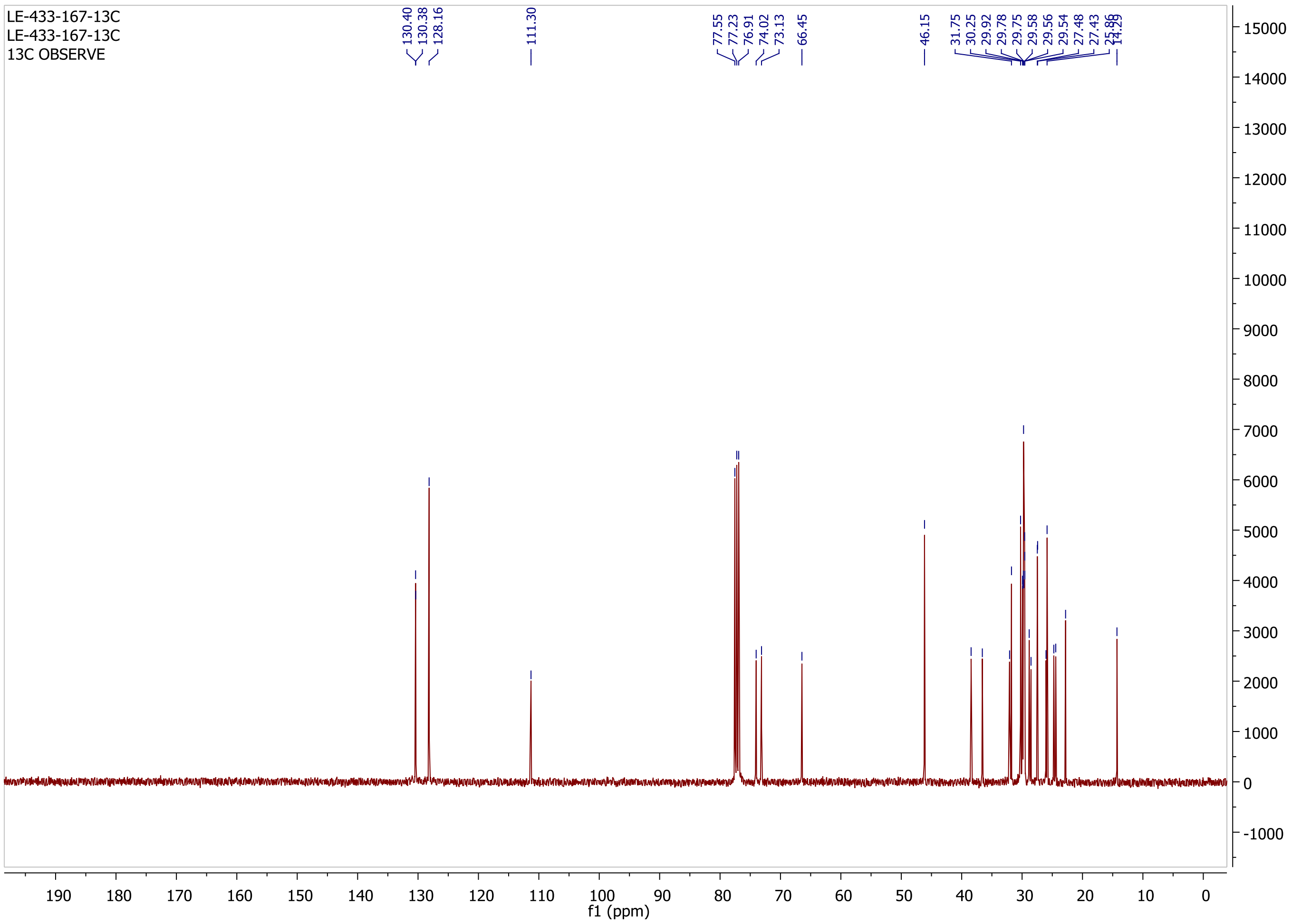

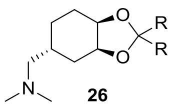

26

R = linoleyl

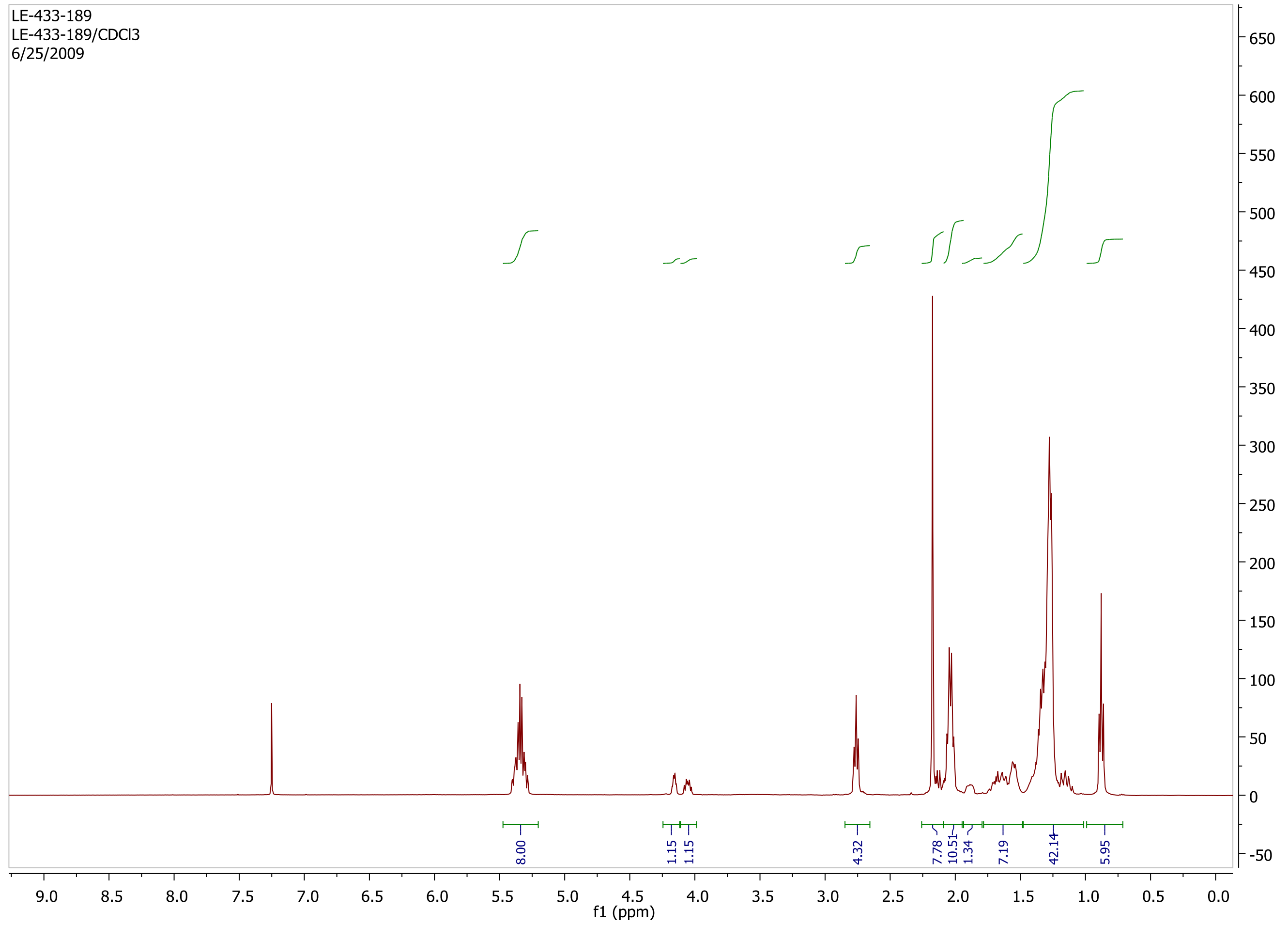

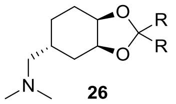

26

R = linoleyl

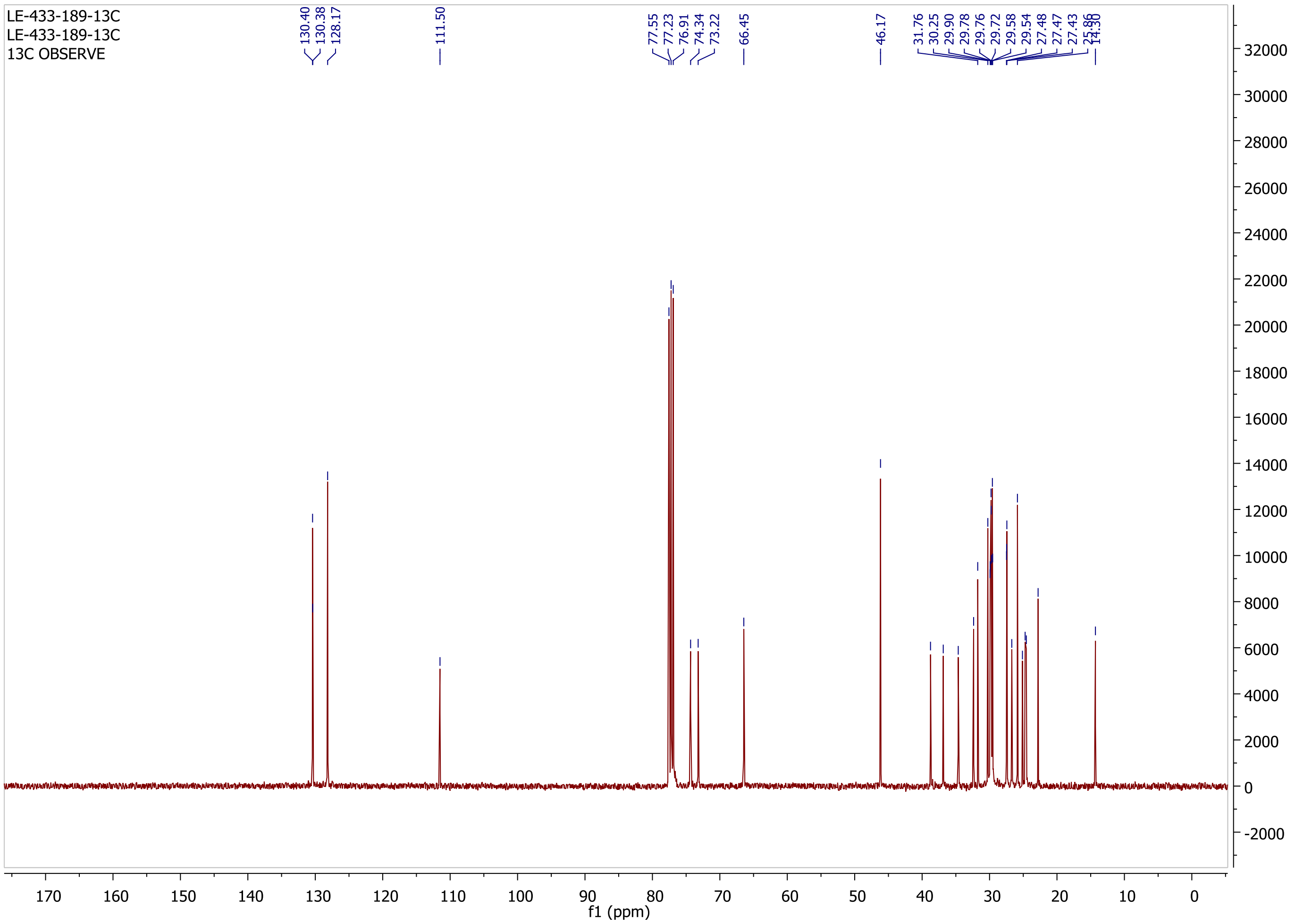

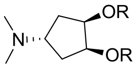

R = linoleyl

27

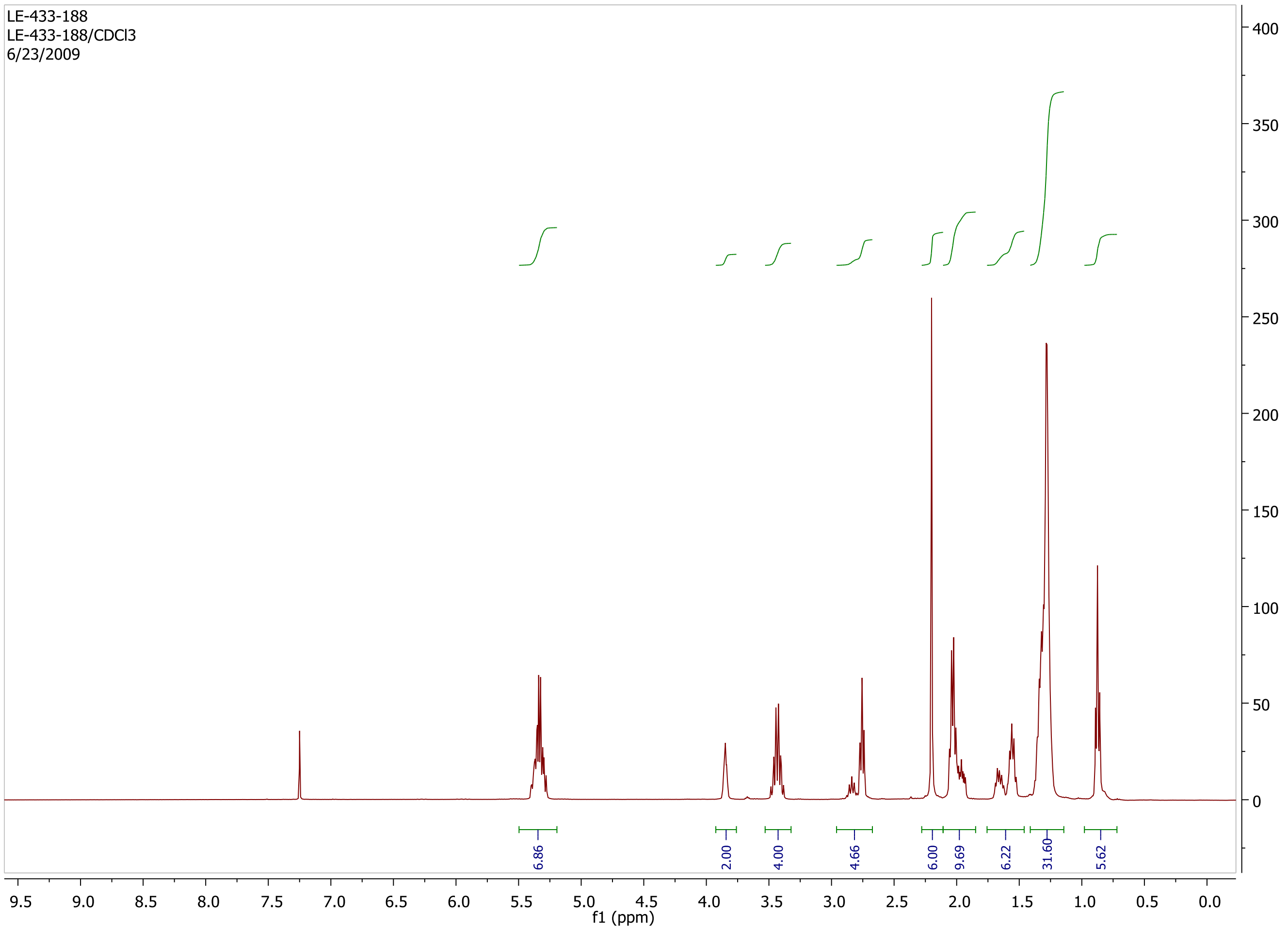

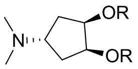

R = linoleyl

27

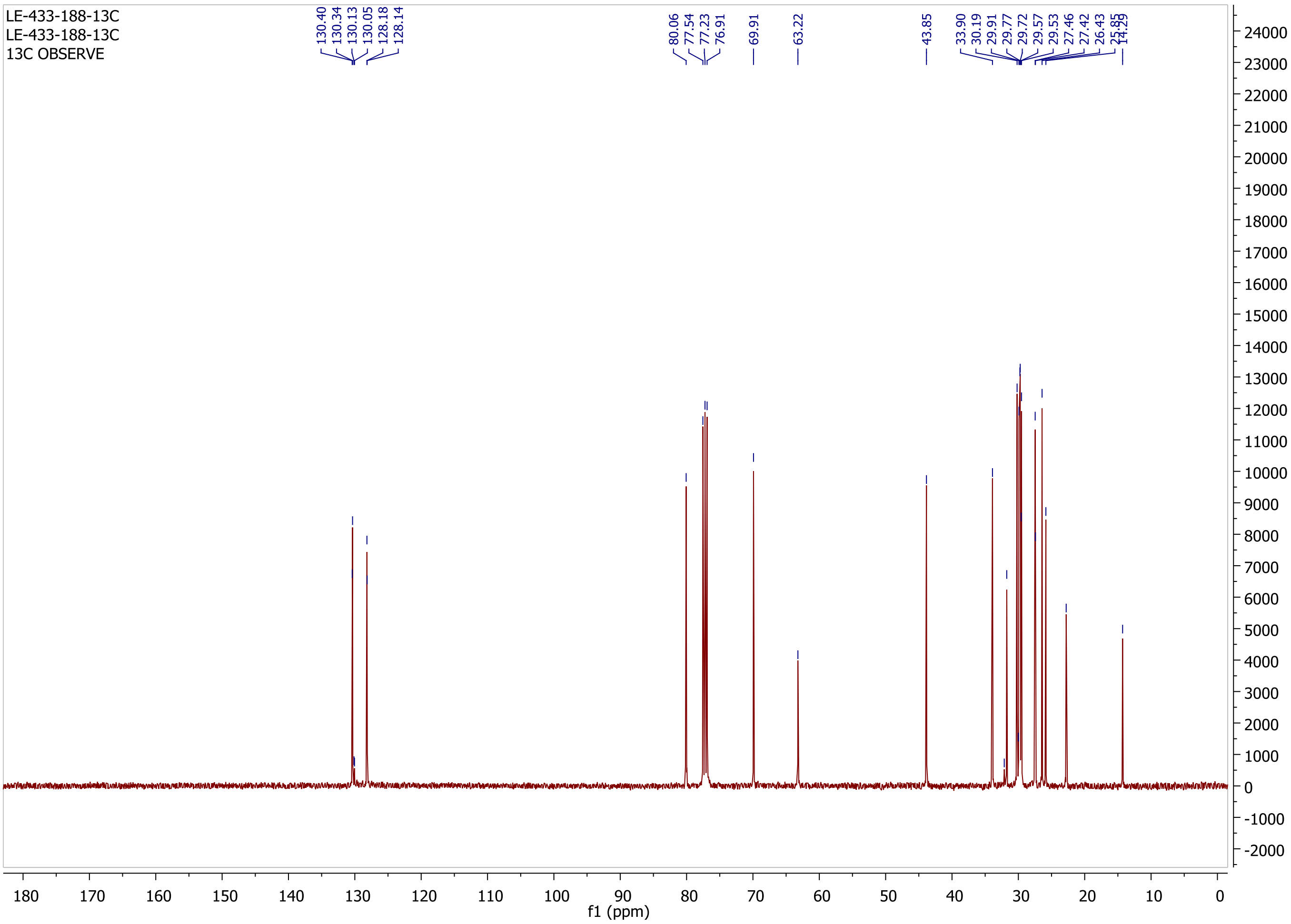

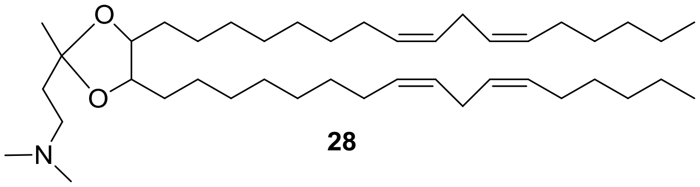

28

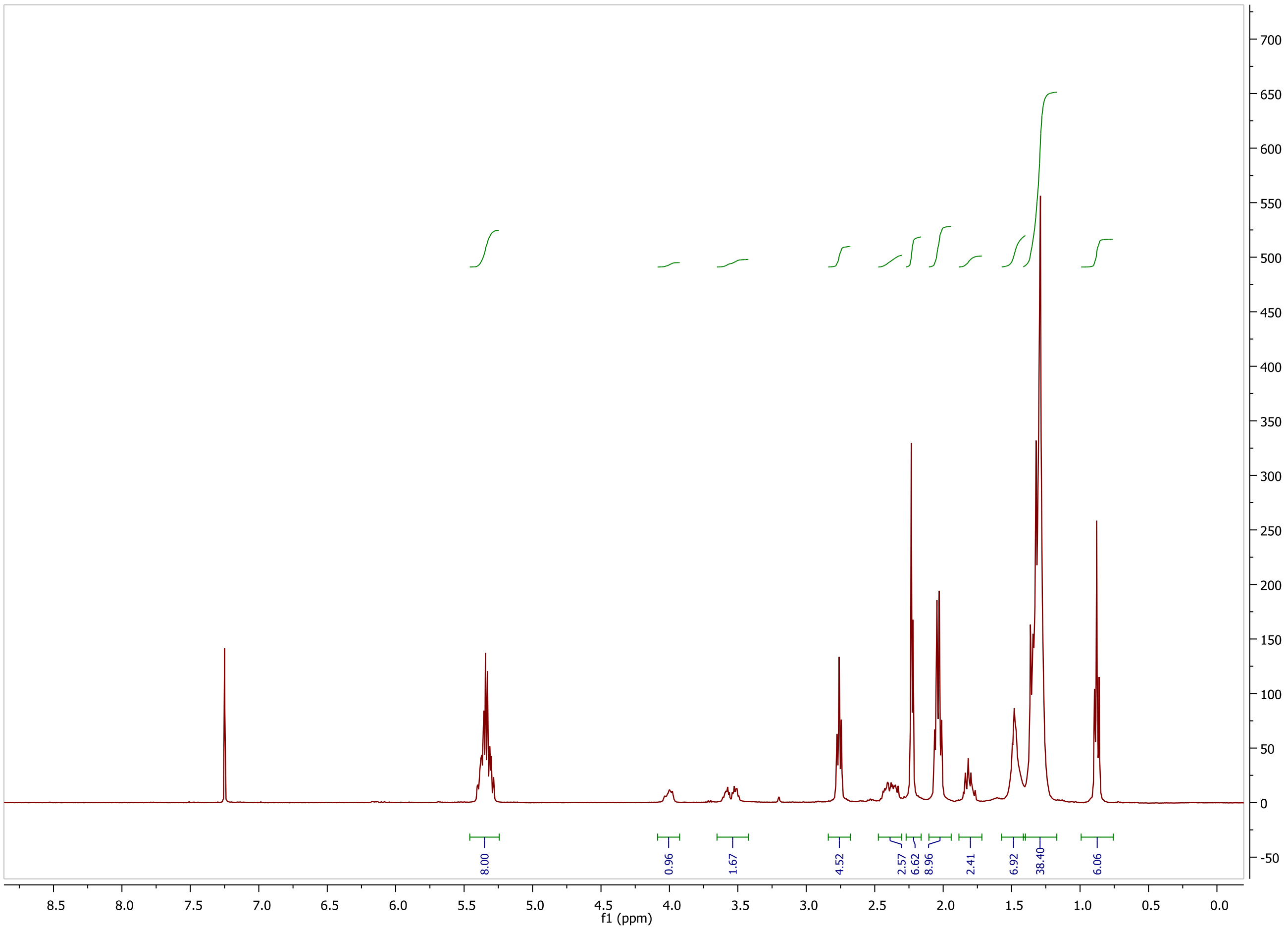

8.00
0.96
1.67
4.52
2.57
6.62
8.96
2.41
6.92
38.40
6.06
8.5 8.0 7.5 7.0 6.5 6.0 5.5 5.0 4.5 4.0 3.5 3.0 2.5 2.0 1.5 1.0 0.5 0.0
f1 (ppm)
700 650 600 550 500 450 400 350 300 250 200 150 100 50 0 -50

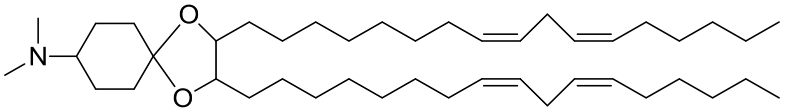
N
O
O

29

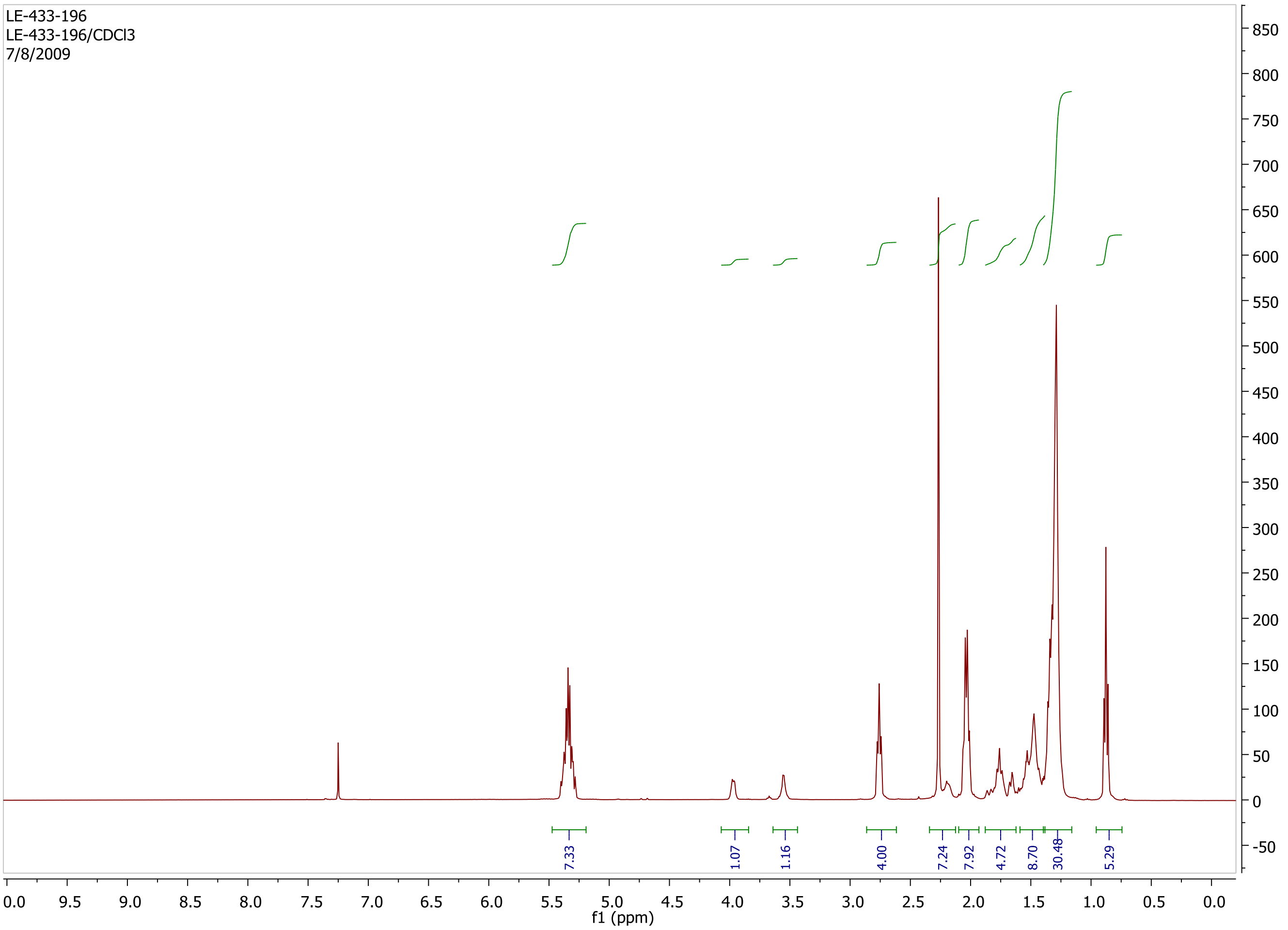
LE-433-196
LE-433-196/CDCl3
7/8/2009
7.33
1.07
1.16
4.00
7.24
7.92
4.72
8.70
30.48
5.29
f1 (ppm)

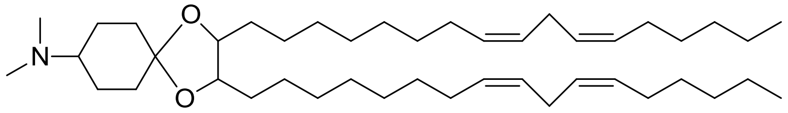

29

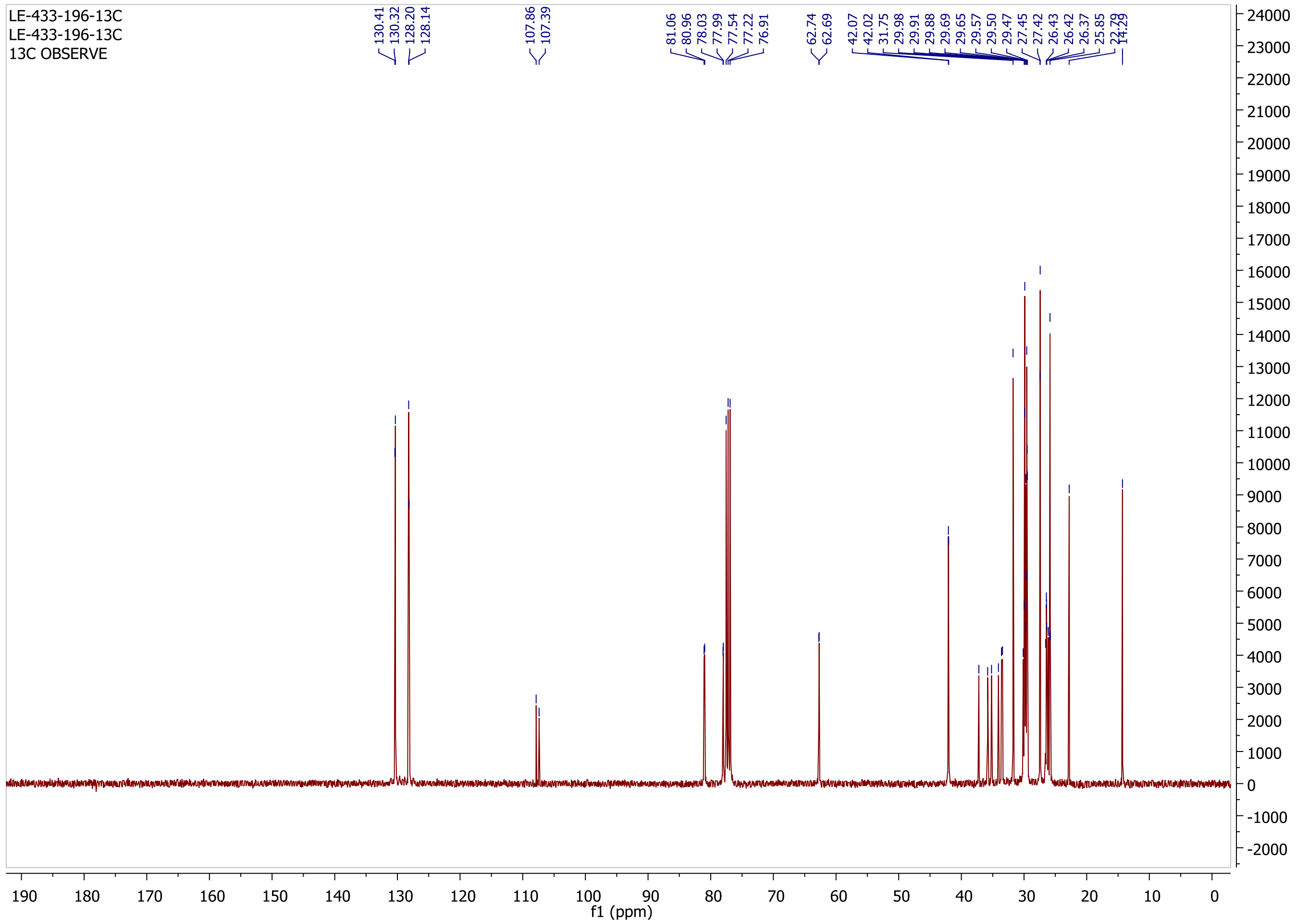

LE-433-196-13C
LE-433-196-13C
13C OBSERVE
130.41
130.32
128.20
128.14
107.86
107.39
81.06
80.96
78.03
77.99
77.54
77.22
76.91
62.74
62.69
42.07
42.02
31.75
29.98
29.91
29.88
29.69
29.65
29.57
29.50
29.47
27.45
27.42
26.43
26.42
26.37
25.85
22.79
14.29
f1 (ppm)

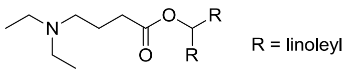

N
O
O
R
R
R = linoleyl

30

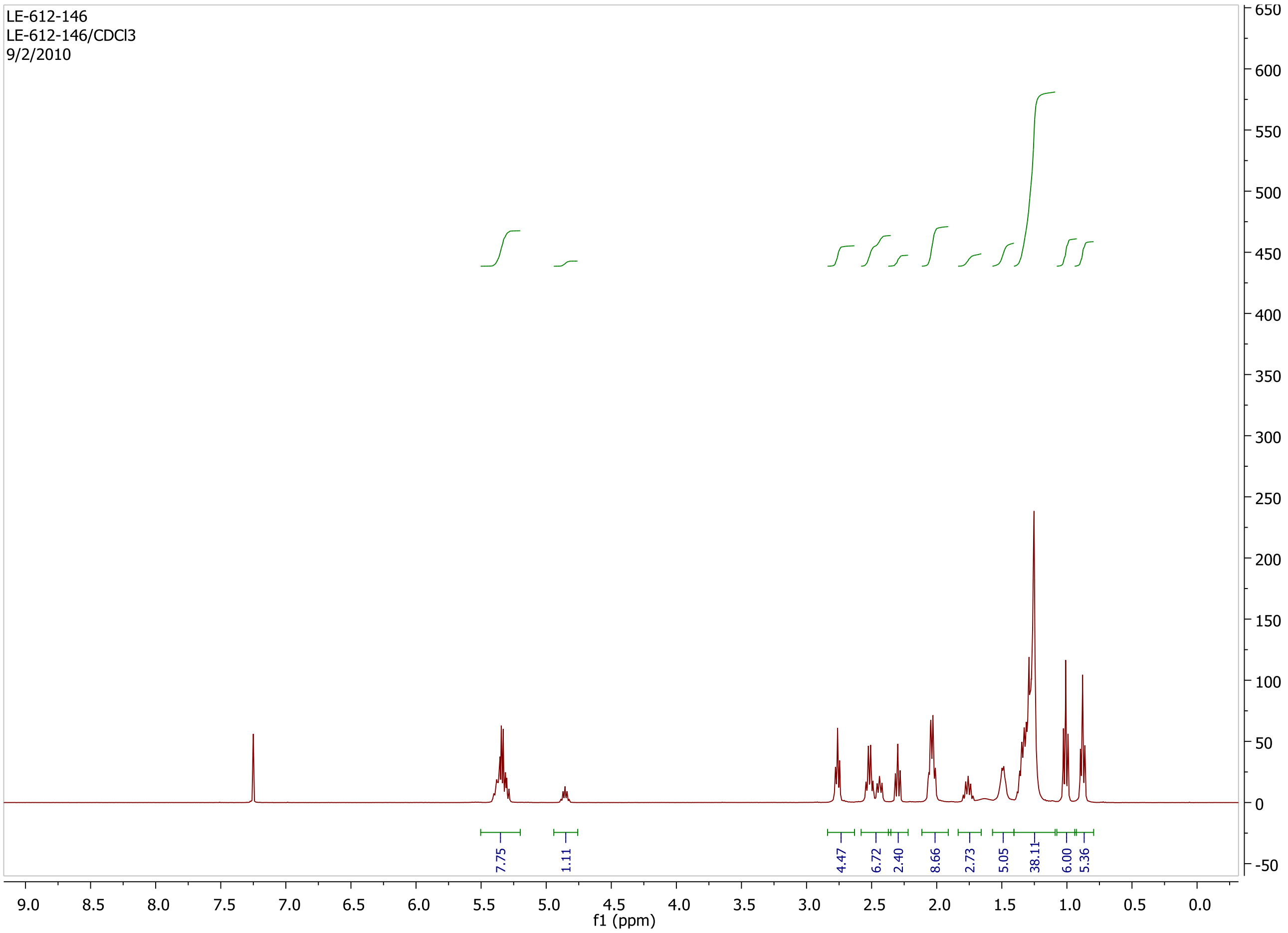

LE-612-146
LE-612-146/CDCl3
9/2/2010
7.75
1.11
4.47
6.72
2.40
8.66
2.73
5.05
38.11
6.00
5.36
f1 (ppm)

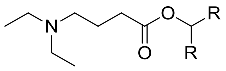

R = linoleyl

30

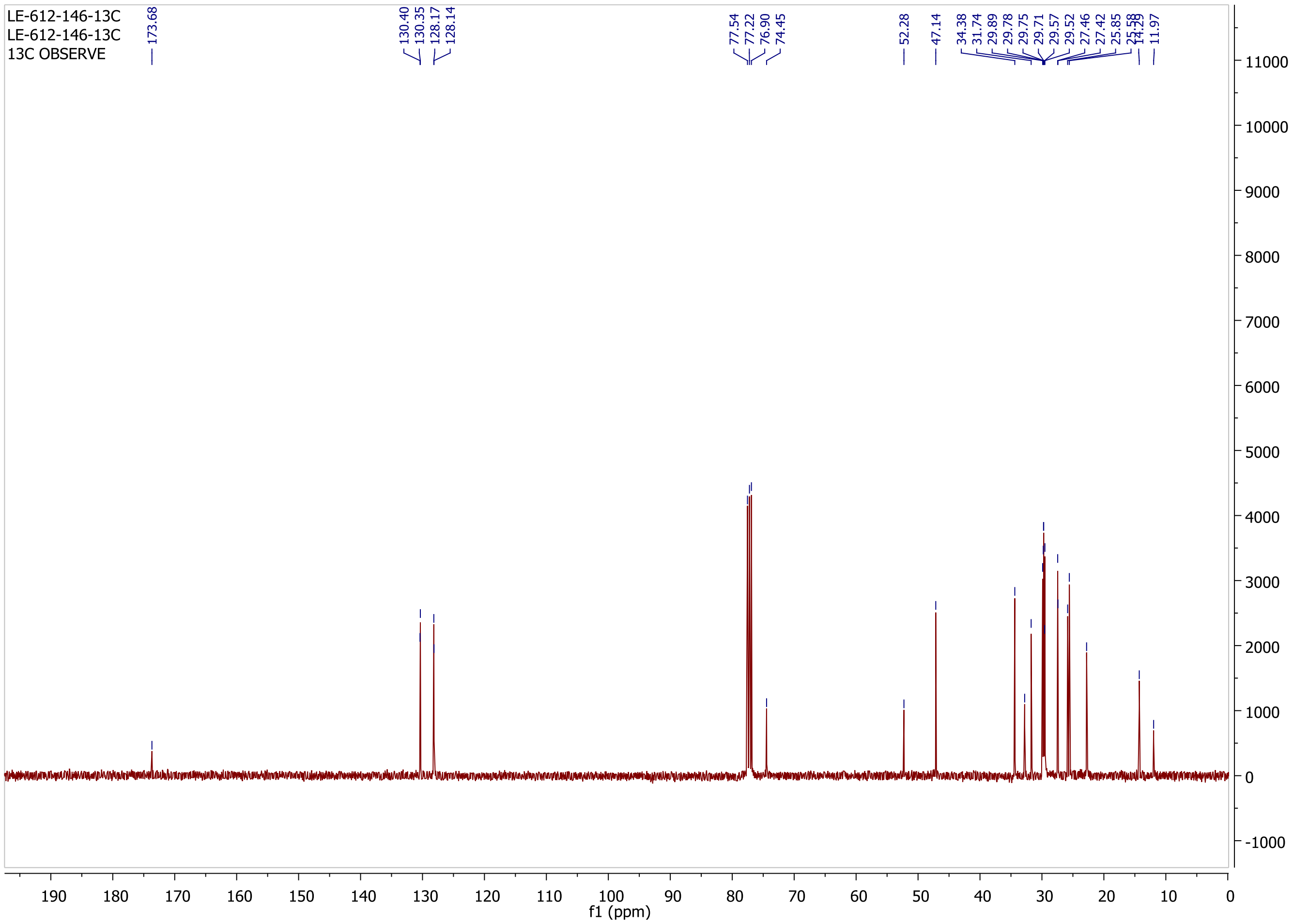

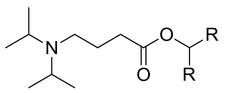

R = linoleyl

31

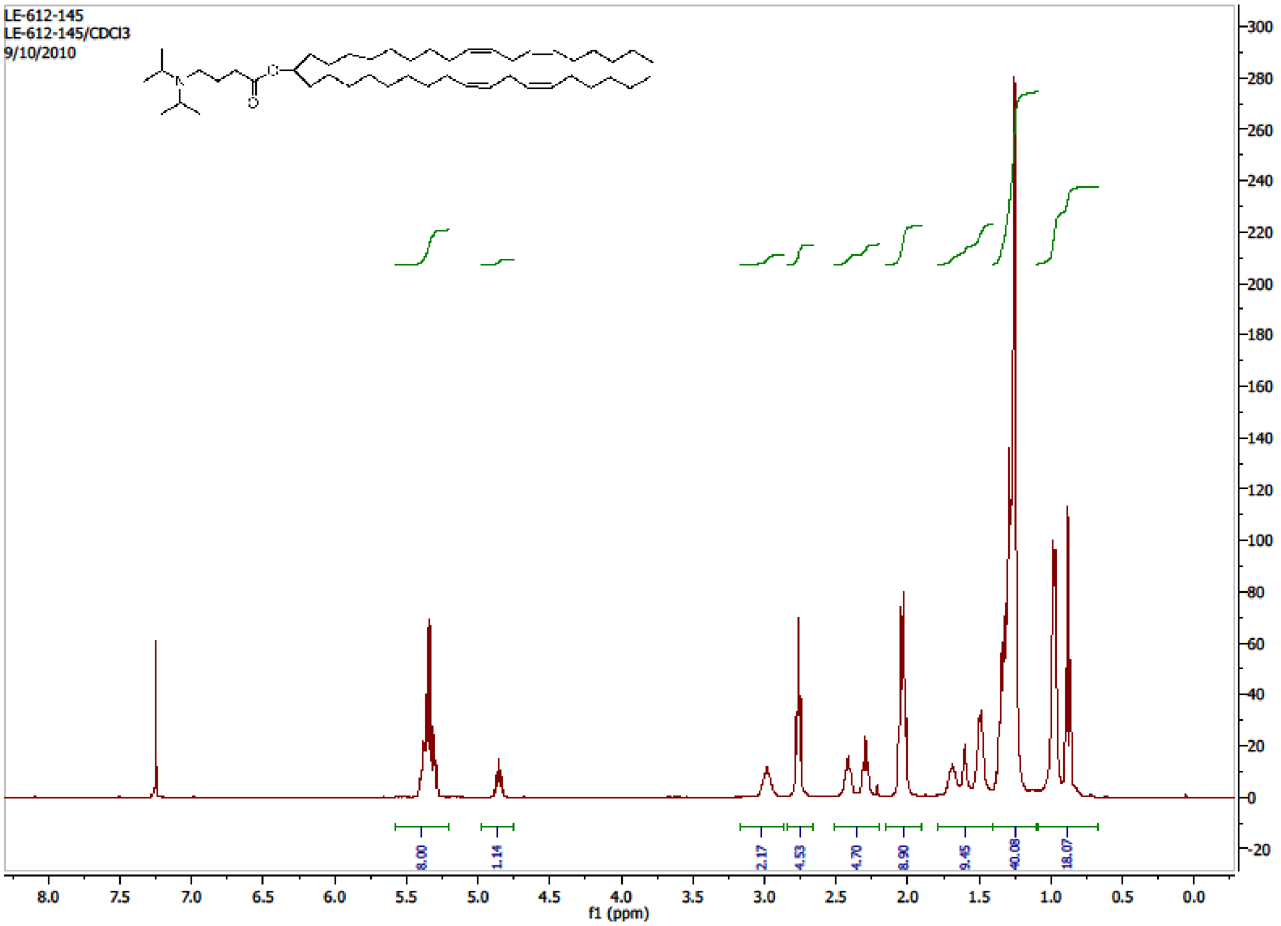

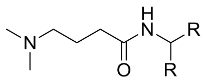

R = linoleyl

32

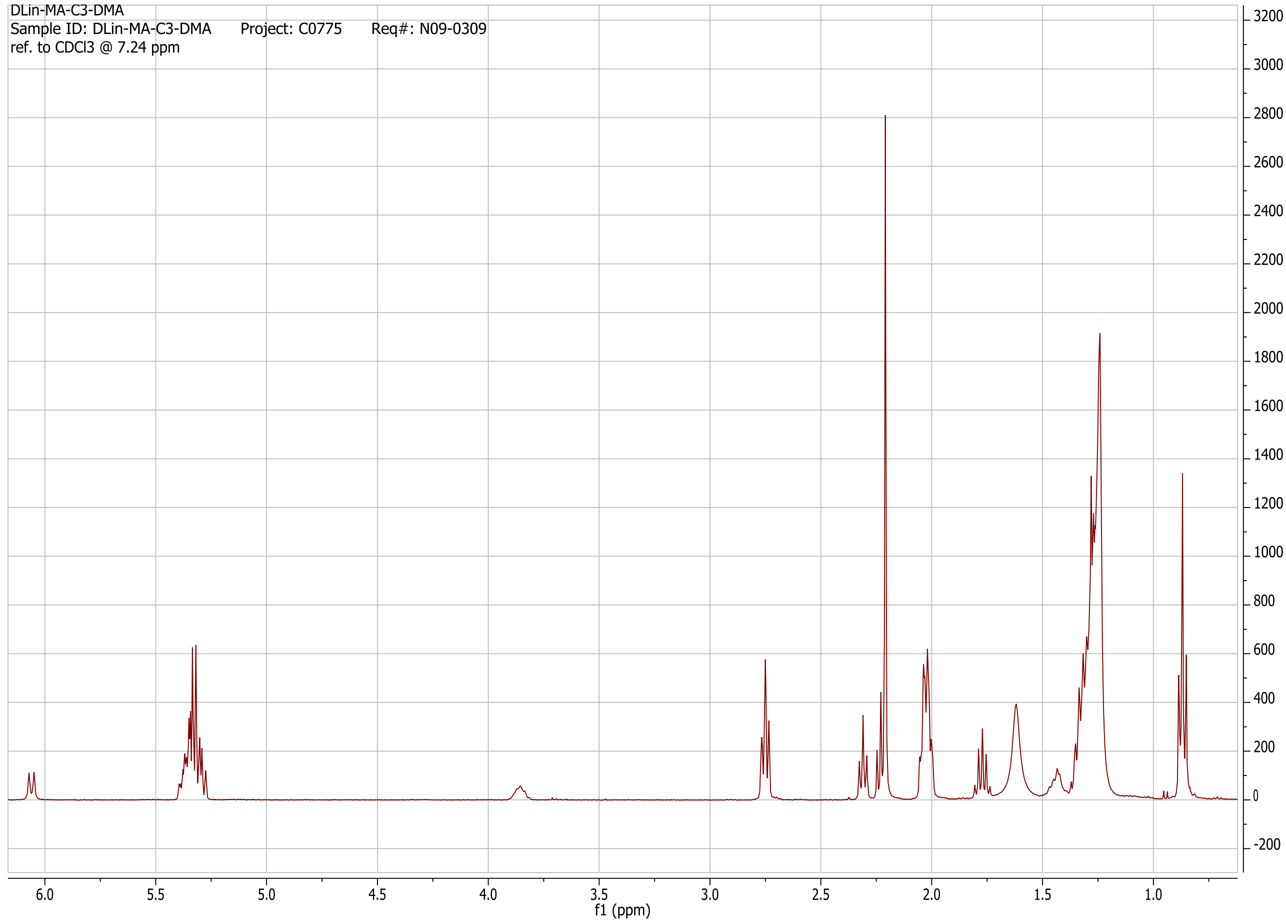

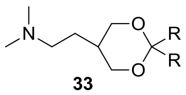

33

R = linoleyl

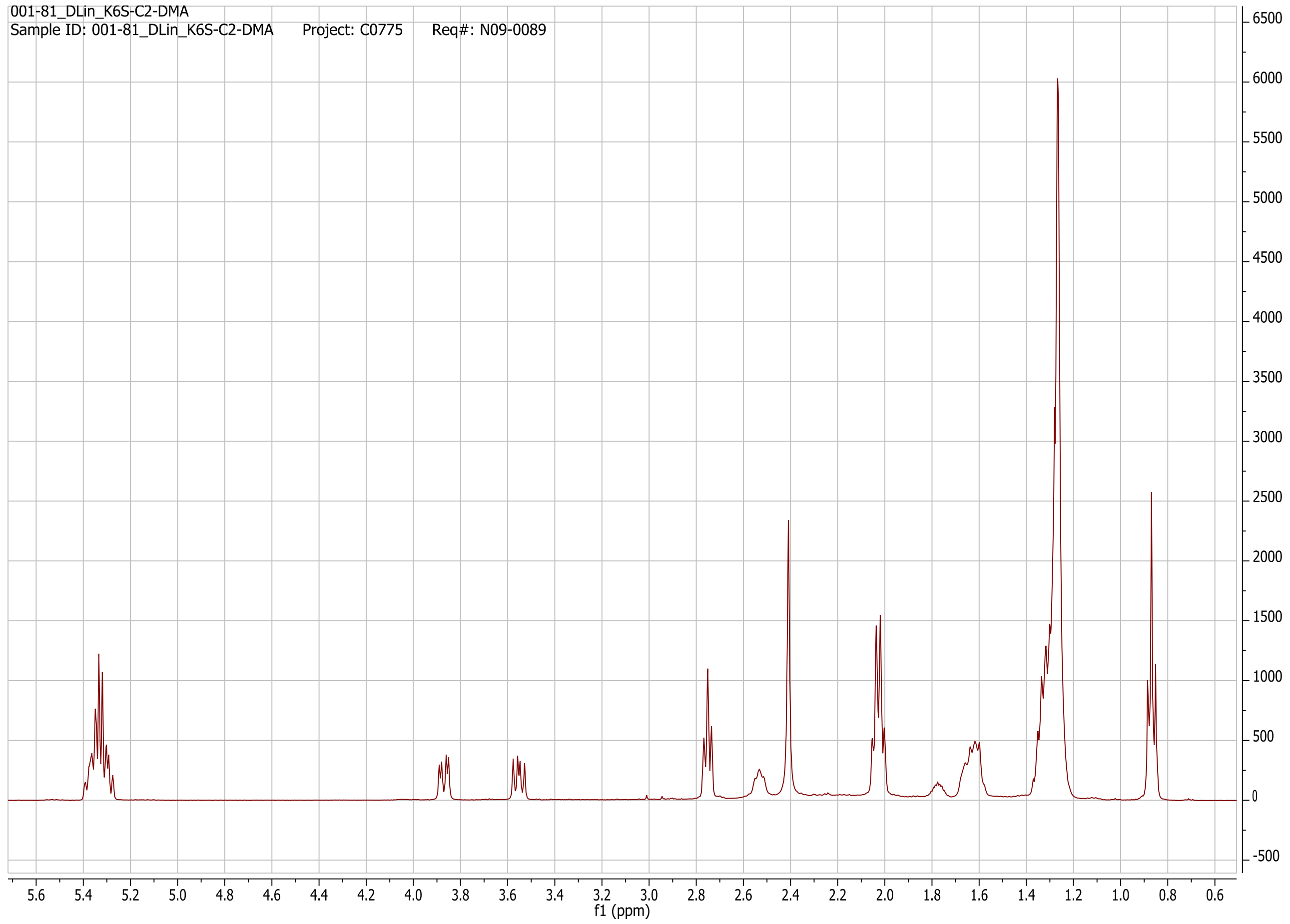

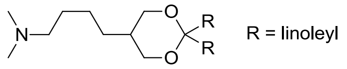
N
O
O
R
R
R = linoleyl

34

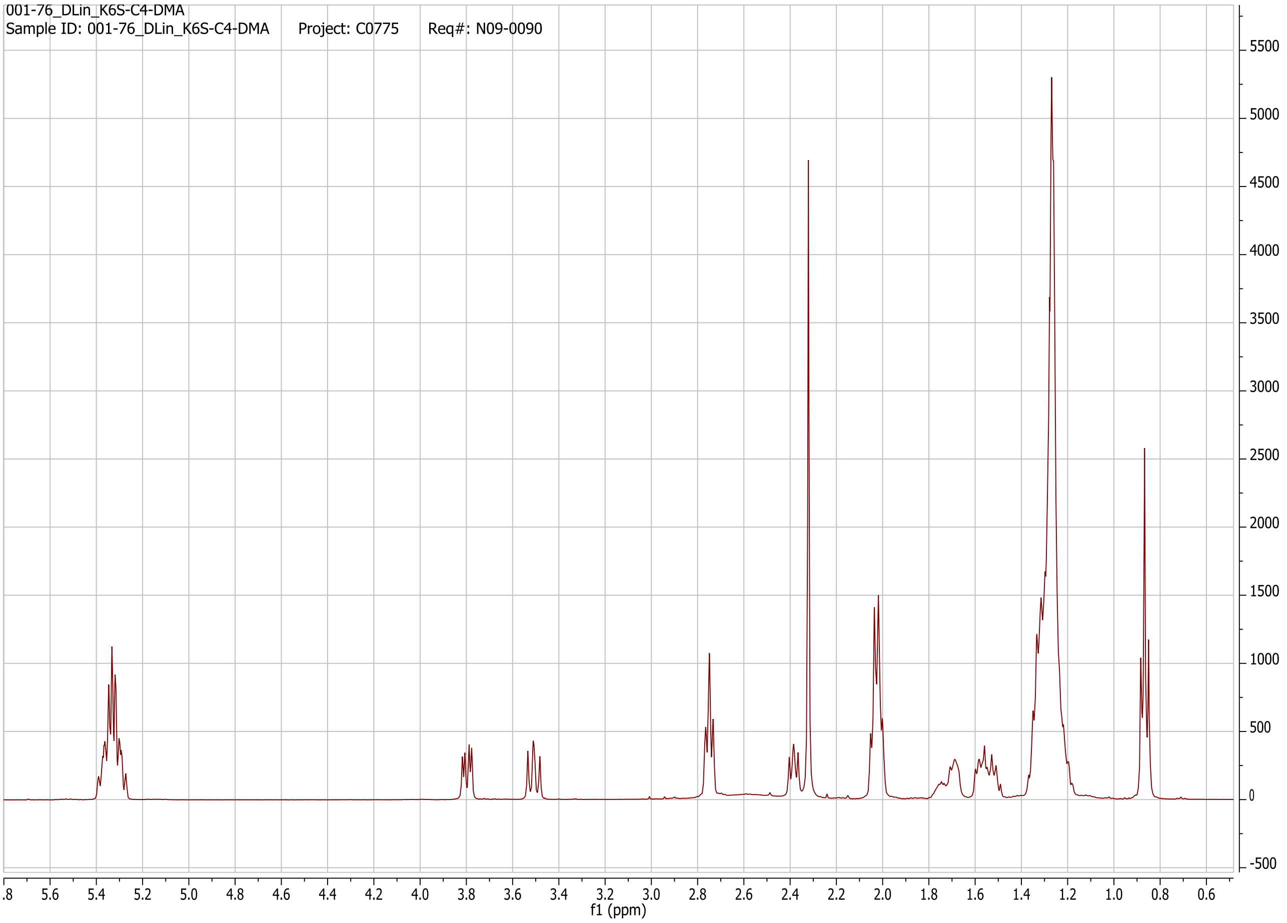
001-76_DLin_K6S-C4-DMA
Sample ID: 001-76_DLin_K6S-C4-DMA
Project: C0775
Req#: N09-0090
5500
5000
4500
4000
3500
3000
2500
2000
1500
1000
500
0
-500
.8
5.6
5.4
5.2
5.0
4.8
4.6
4.4
4.2
4.0
3.8
3.6
3.4
3.2
3.0
2.8
2.6
2.4
2.2
2.0
1.8
1.6
1.4
1.2
1.0
0.8
0.6
f1 (ppm)

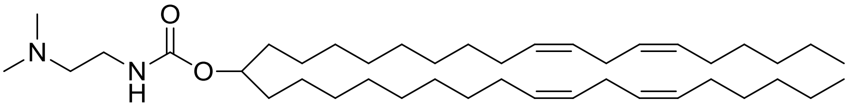

35

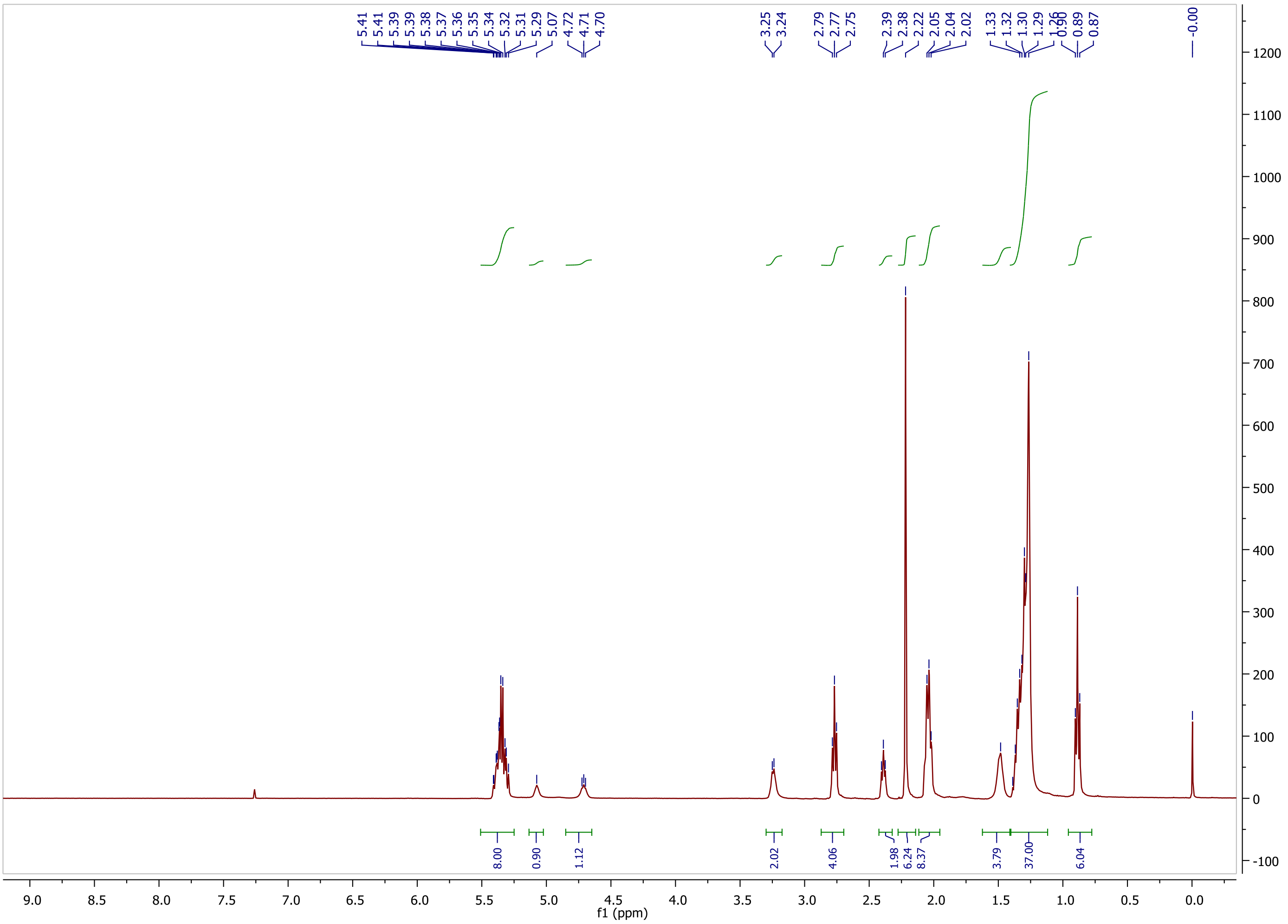

5.41
5.41
5.39
5.39
5.38
5.37
5.36
5.35
5.34
5.32
5.31
5.29
5.07
4.72
4.71
4.70
3.25
3.24
2.79
2.77
2.75
2.39
2.38
2.22
2.05
2.04
2.02
1.33
1.32
1.30
1.29
1.26
0.90
0.89
0.87
-0.00
8.00
0.90
1.12
2.02
4.06
1.98
6.24
8.37
3.79
37.00
6.04
f1 (ppm)

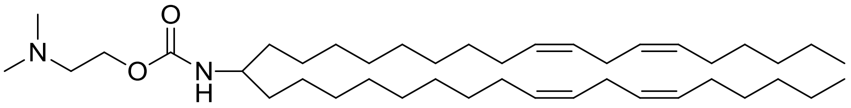
O
N
O
N
H

36

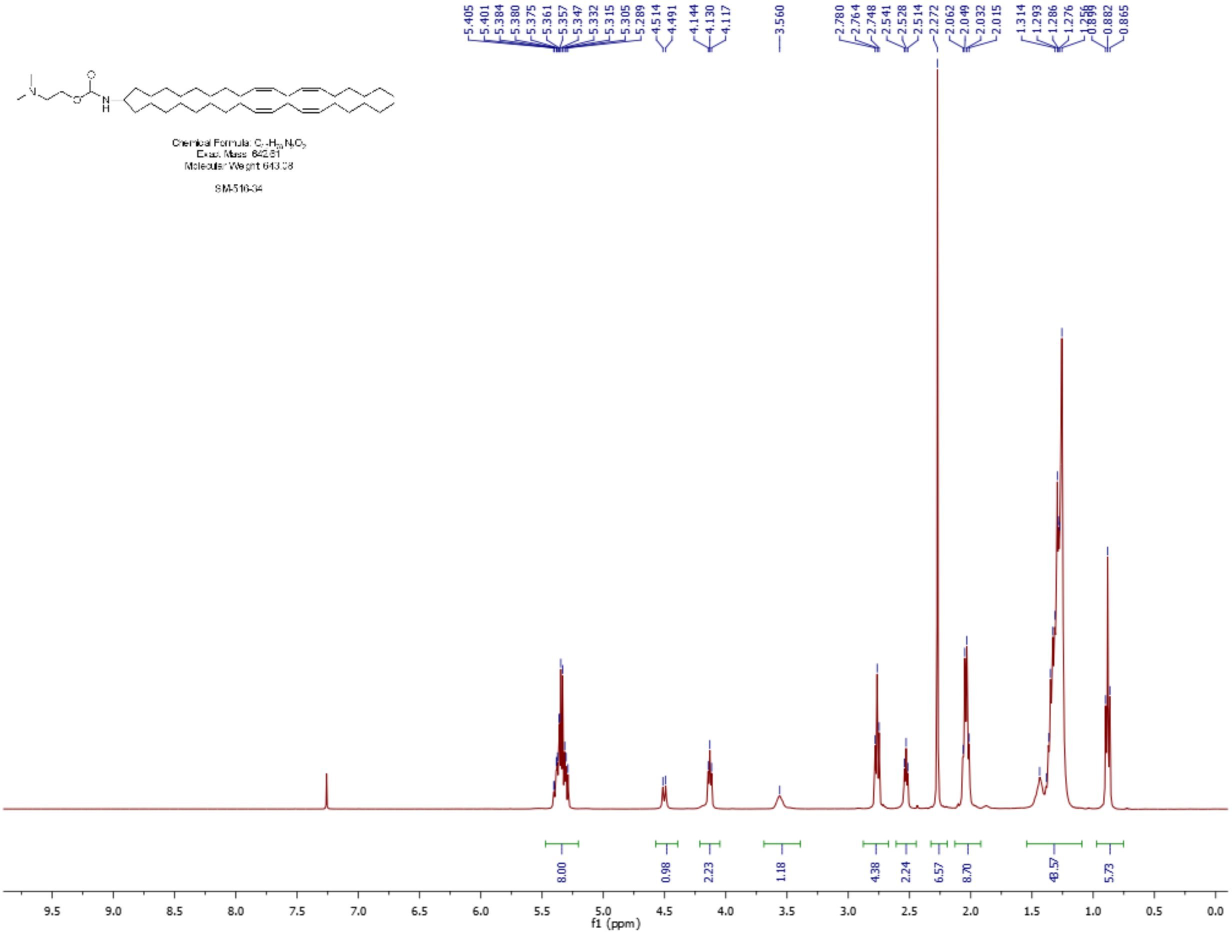
5.405 5.401 5.384 5.380 5.375 5.361 5.357 5.347 5.332 5.315 5.305 5.289 4.514 4.491 4.144 4.130 4.117 3.560 2.780 2.764 2.748 2.541 2.528 2.514 2.272 2.062 2.049 2.032 2.015 1.314 1.293 1.286 1.276 0.882 0.865
Exact Mass: 642.61
Molecular Weight: 643.08
SM-516-34
8.00
0.98
2.23
1.18
4.38
2.24
6.57
8.70
43.57
5.73
9.5 9.0 8.5 8.0 7.5 7.0 6.5 6.0 5.5 5.0 4.5 4.0 3.5 3.0 2.5 2.0 1.5 1.0 0.5 0.0
f1 (ppm)

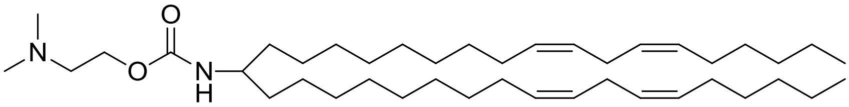

36

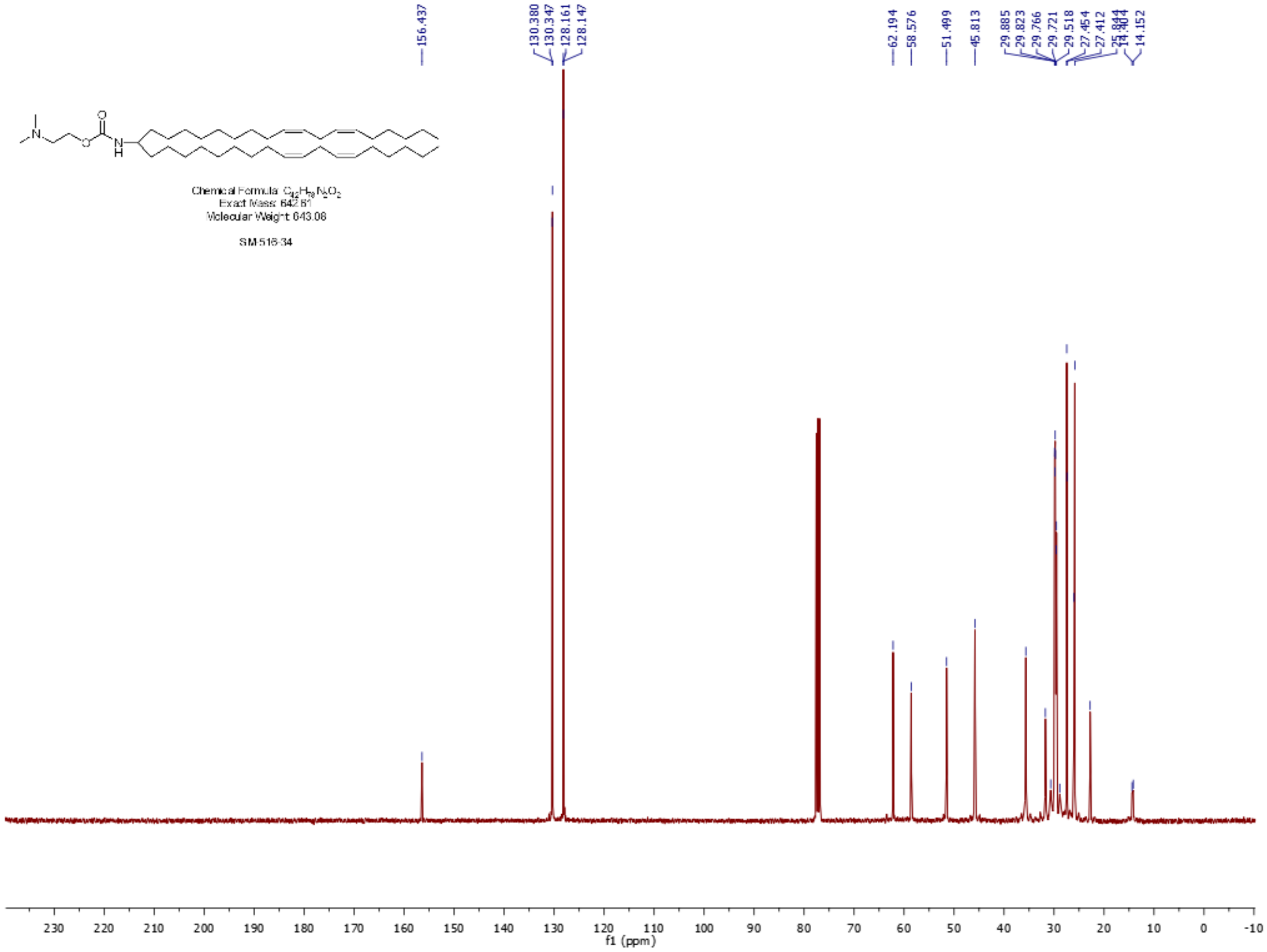
156.437
130.380
130.347
128.161
128.147
62.194
58.576
51.499
45.813
29.885
29.823
29.766
29.721
29.518
27.454
27.412
14.152
Chemical Formula: C42H78N2O2
Exact Mass: 642.61
Molecular Weight: 643.08
SM-516-34
f1 (ppm)

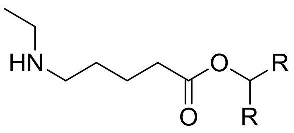

R = linoleyl

37

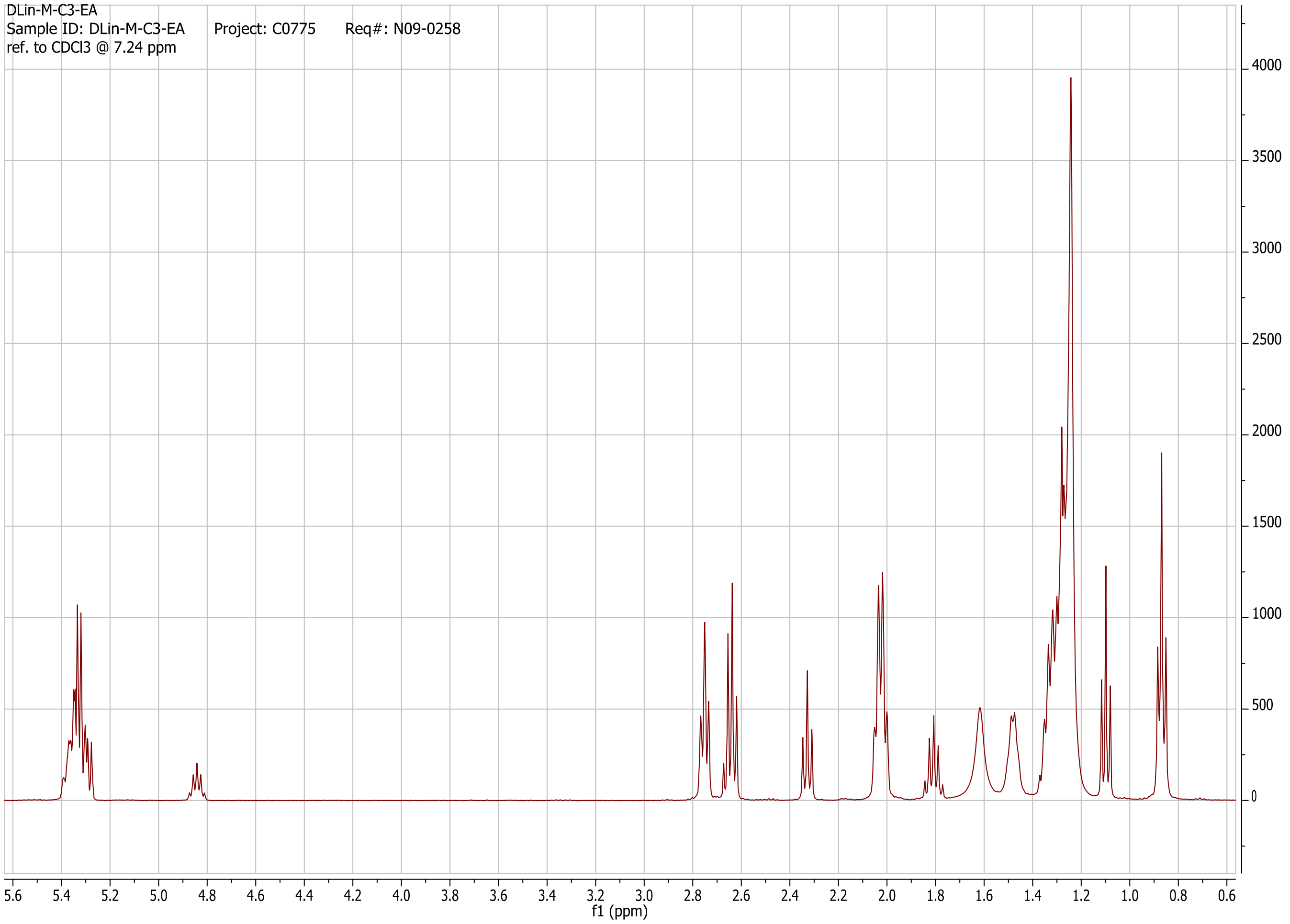

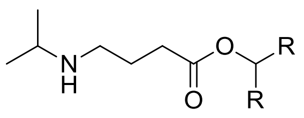

R = linoleyl

38

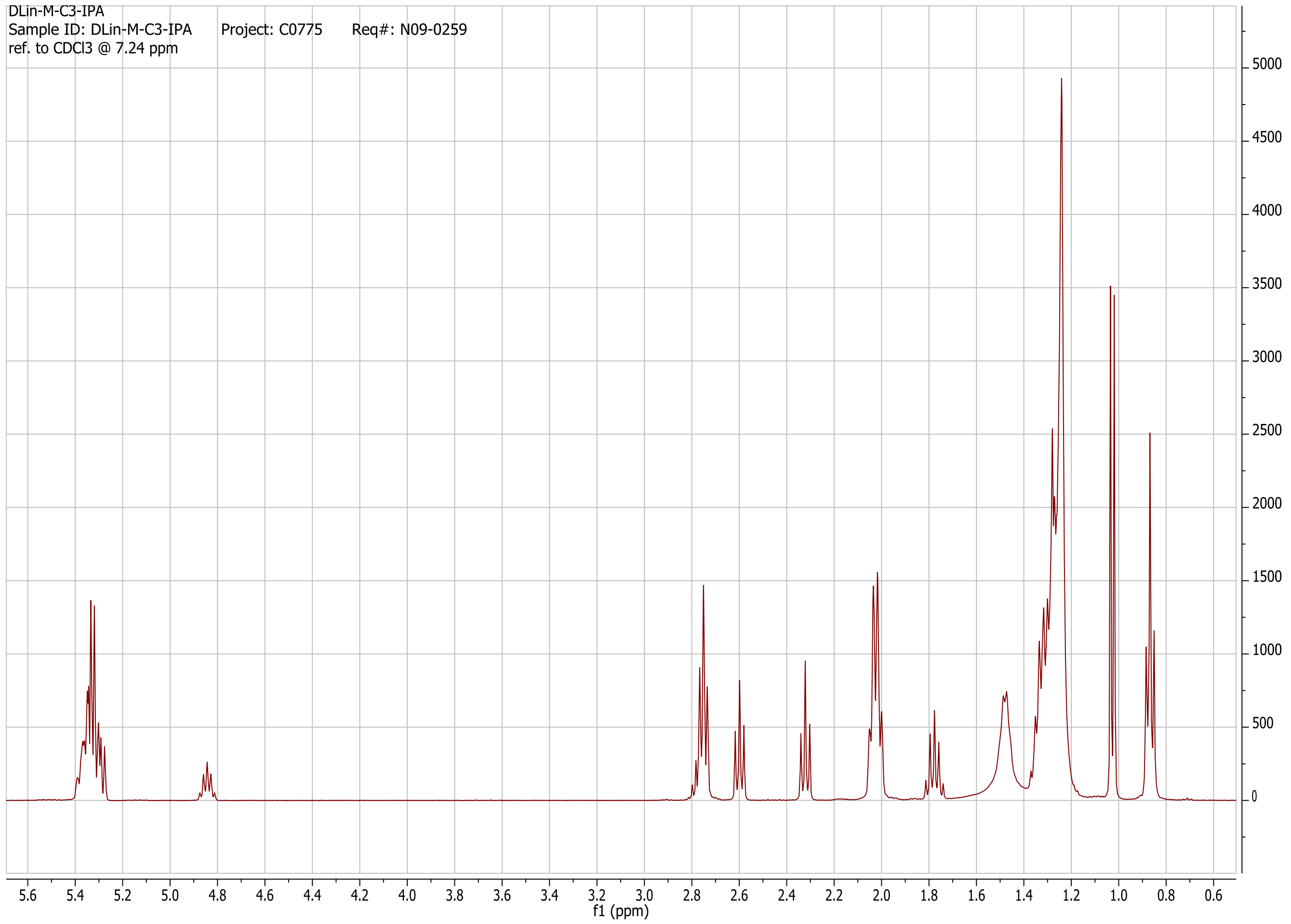

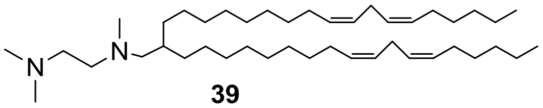

39

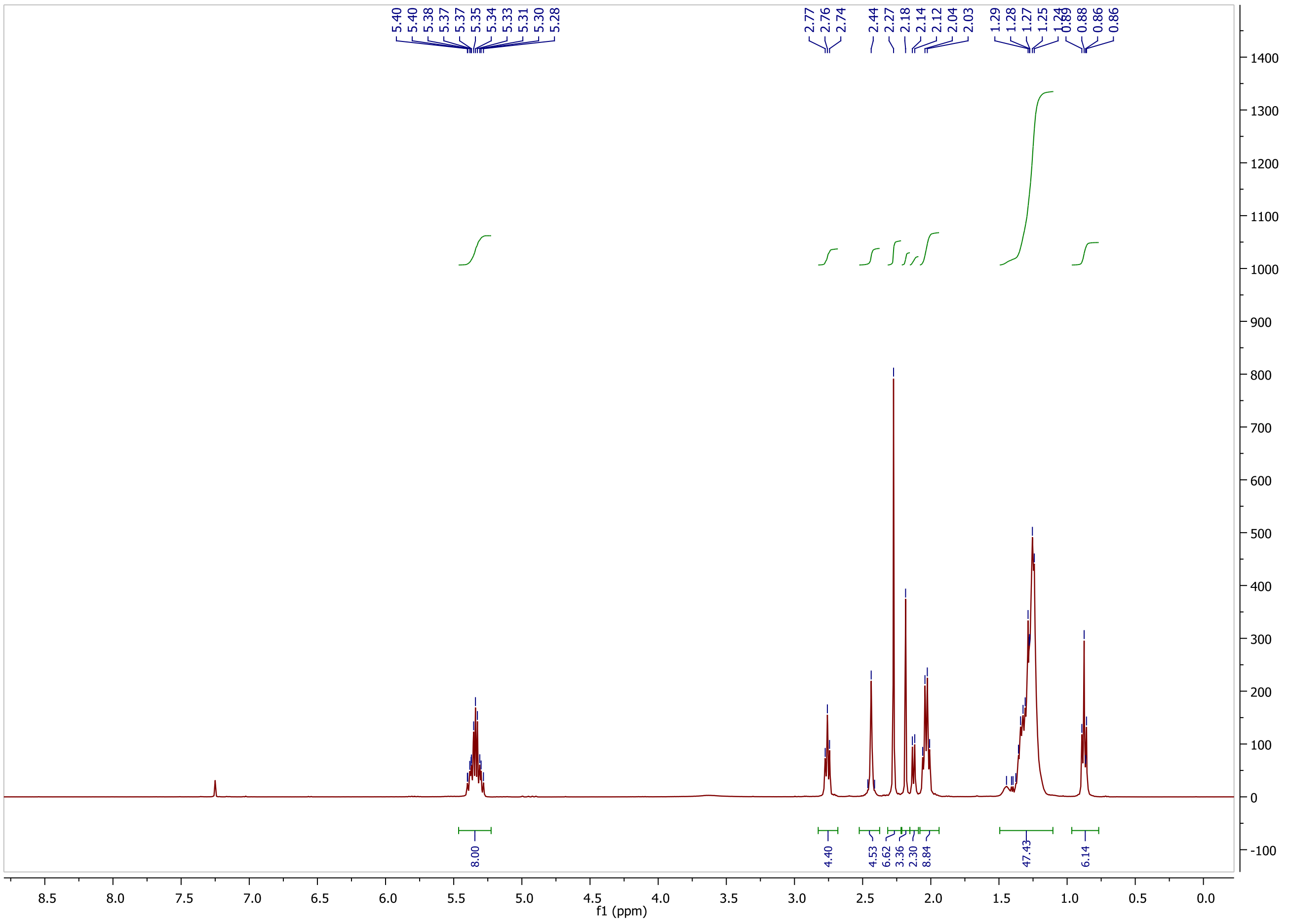

5.40 5.40 5.38 5.37 5.37 5.35 5.34 5.33 5.31 5.30 5.28
2.77 2.76 2.74
2.44 2.27 2.18 2.14 2.12 2.04 2.03
1.29 1.28 1.27 1.25 1.24 0.89 0.88 0.86 0.86
8.00
4.40
4.53 6.62 3.36 2.30 8.84
47.43
6.14
8.5 8.0 7.5 7.0 6.5 6.0 5.5 5.0 4.5 4.0 3.5 3.0 2.5 2.0 1.5 1.0 0.5 0.0
f1 (ppm)
1400 1300 1200 1100 1000 900 800 700 600 500 400 300 200 100 0 -100

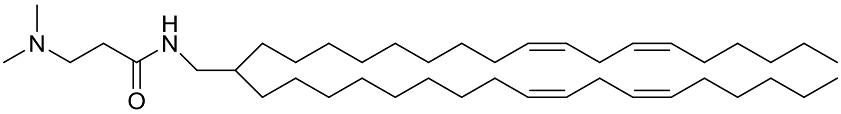

H
N
N
O

40

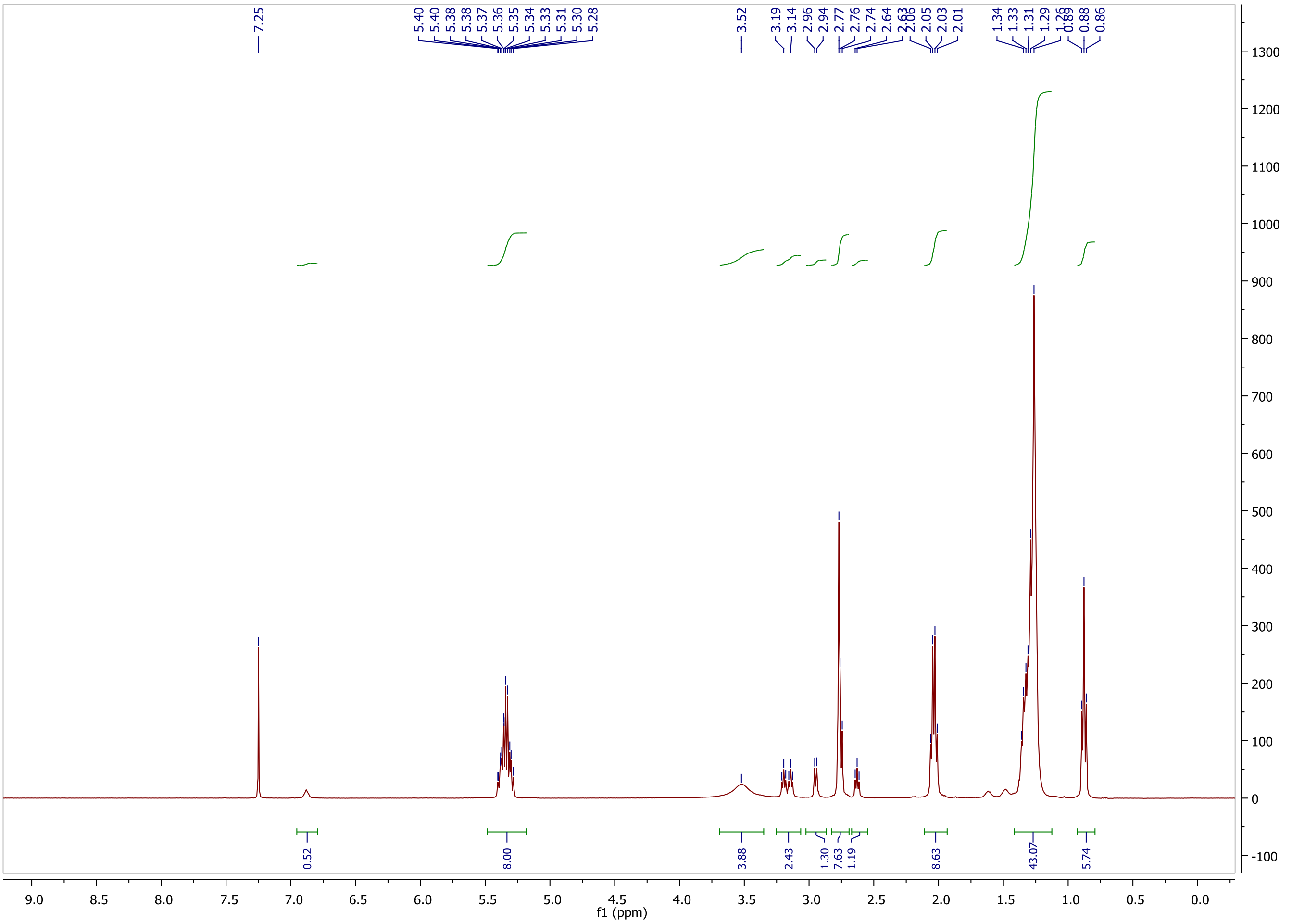

7.25
5.40
5.40
5.38
5.38
5.37
5.36
5.35
5.34
5.33
5.31
5.30
5.28
3.52
3.19
3.14
2.96
2.94
2.77
2.76
2.74
2.64
2.63
2.06
2.05
2.03
2.01
1.34
1.33
1.31
1.29
1.26
0.89
0.88
0.86
0.52
8.00
3.88
2.43
1.30
7.63
1.19
8.63
43.07
5.74
9.0 8.5 8.0 7.5 7.0 6.5 6.0 5.5 5.0 4.5 4.0 3.5 3.0 2.5 2.0 1.5 1.0 0.5 0.0
f1 (ppm)
1300 1200 1100 1000 900 800 700 600 500 400 300 200 100 0 -100

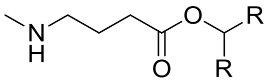

R = linoleyl

41

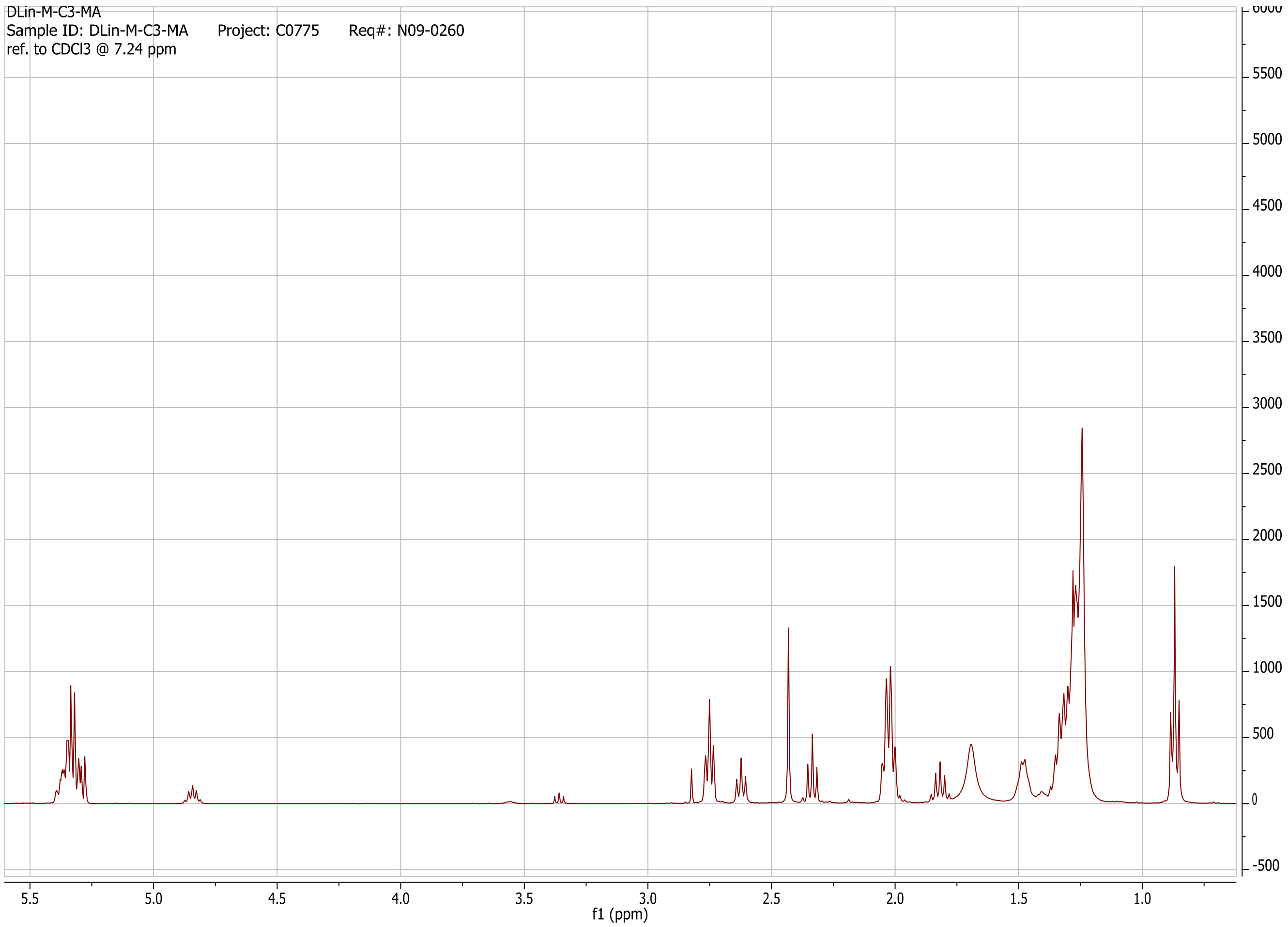

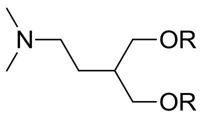

R = linoleyl

42

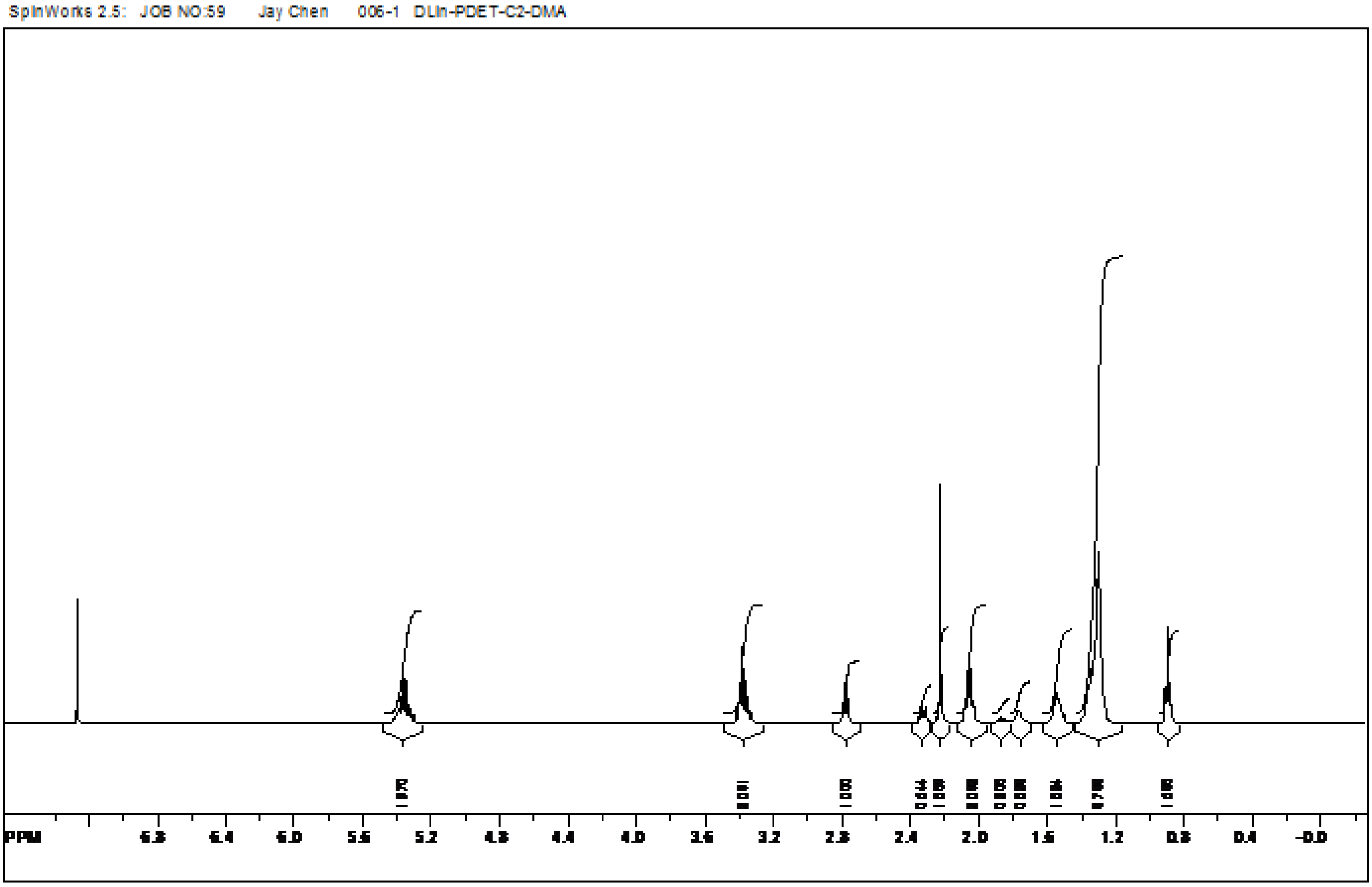

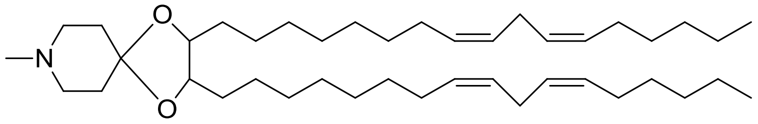

43

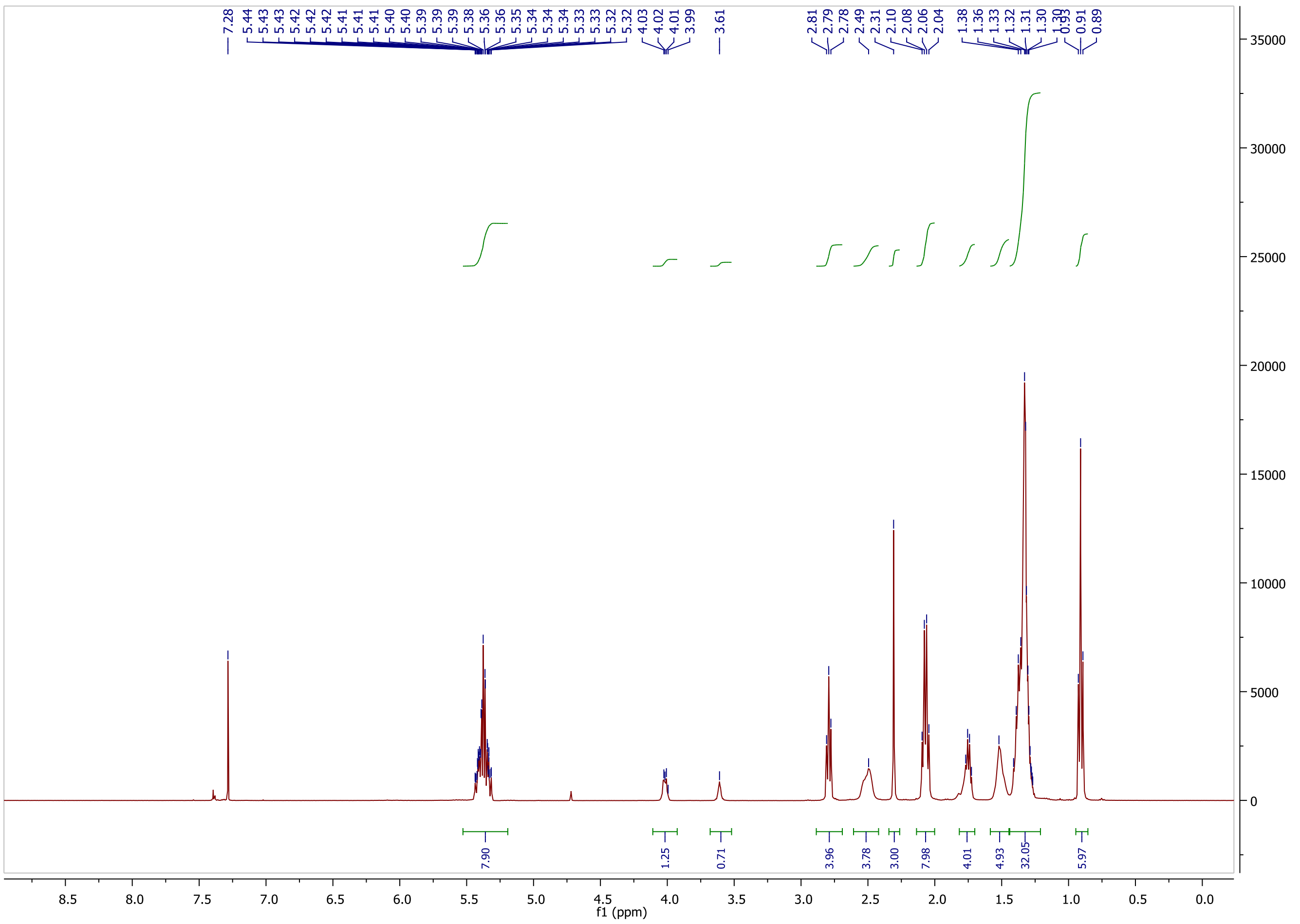

7.28
5.44
5.43
5.43
5.42
5.42
5.42
5.41
5.41
5.41
5.40
5.39
5.39
5.39
5.38
5.36
5.35
5.34
5.34
5.34
5.33
5.33
5.32
5.32
4.03
4.02
4.01
3.99
3.61
2.81
2.79
2.78
2.49
2.31
2.10
2.08
2.06
2.04
1.38
1.36
1.33
1.32
1.31
1.30
0.91
0.89
7.90
1.25
0.71
3.96
3.78
3.00
7.98
4.01
4.93
32.05
5.97
f1 (ppm)

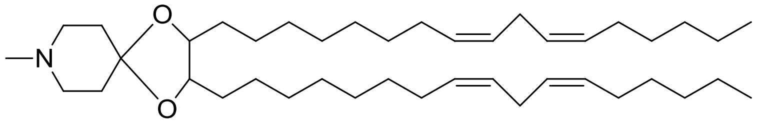

43

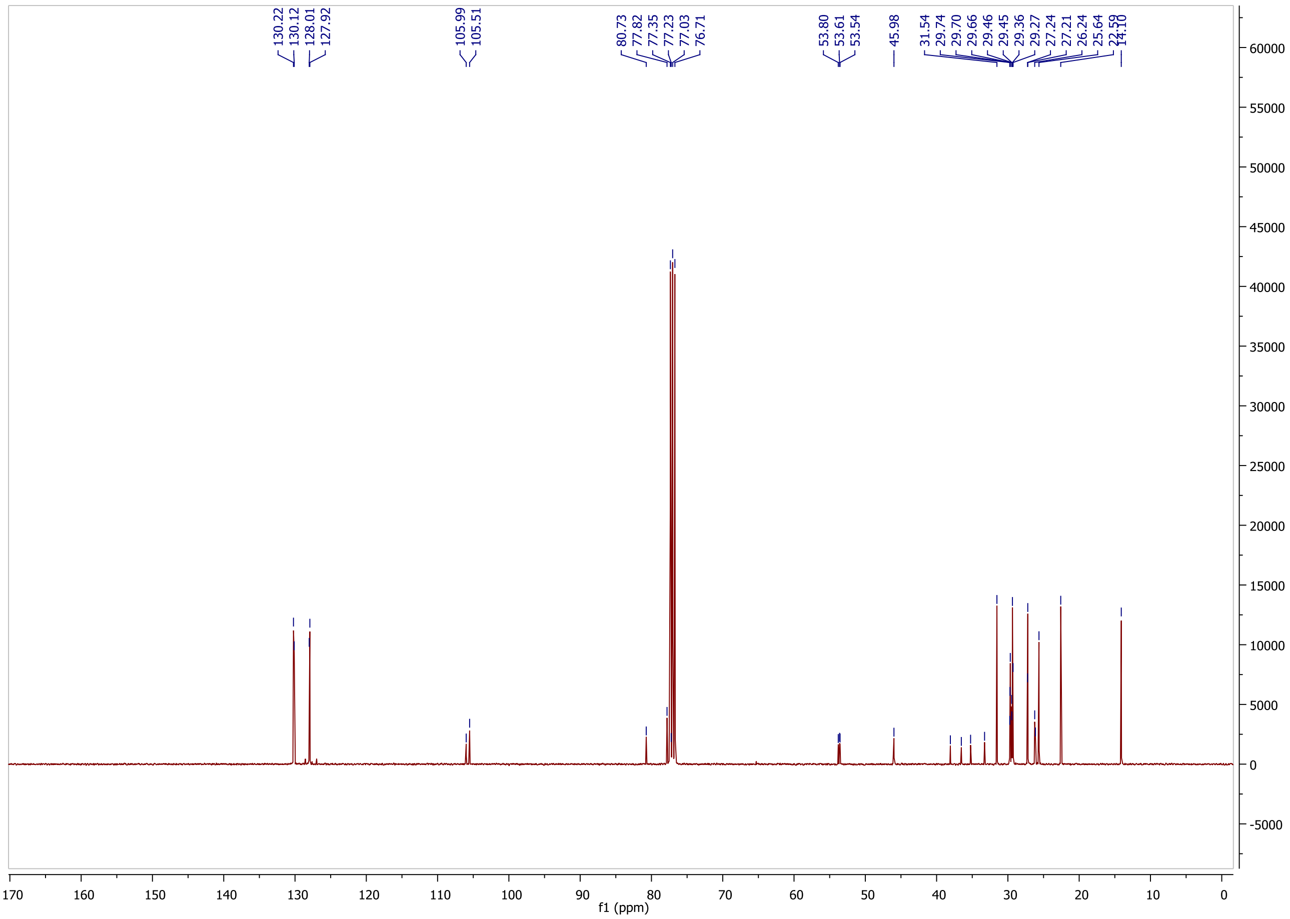

130.22
130.12
128.01
127.92
105.99
105.51
80.73
77.82
77.35
77.23
77.03
76.71
53.80
53.61
53.54
45.98
31.54
29.74
29.70
29.66
29.46
29.45
29.36
29.27
27.24
27.21
26.24
25.64
22.59
14.10
f1 (ppm)

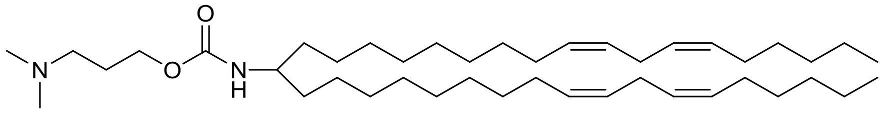

44

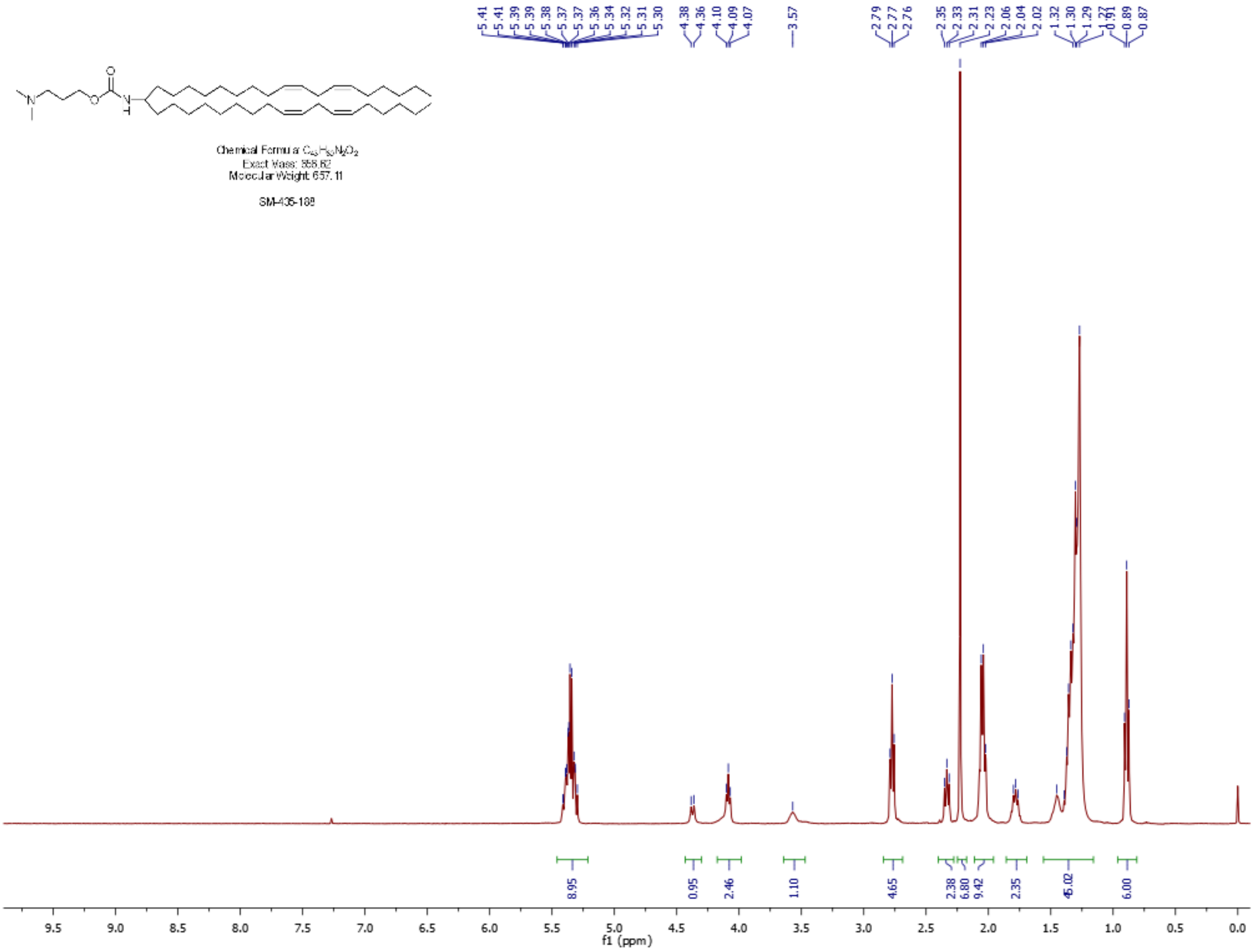

5.41 5.41 5.39 5.39 5.38 5.37 5.37 5.36 5.34 5.32 5.31 5.30 4.38 4.36 4.10 4.09 4.07
3.57
2.79 2.77 2.76 2.35 2.33 2.31 2.23 2.06 2.04 2.02 1.32 1.30 1.29 1.27 0.91 0.89 0.87
Molecular Weight 657.11
8.95
0.95
2.46
1.10
4.65
2.38
6.80
9.42
2.35
45.02
6.00
9.5 9.0 8.5 8.0 7.5 7.0 6.5 6.0 5.5 5.0 4.5 4.0 3.5 3.0 2.5 2.0 1.5 1.0 0.5 0.0
f1 (ppm)

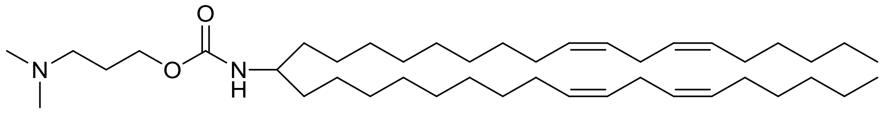

44

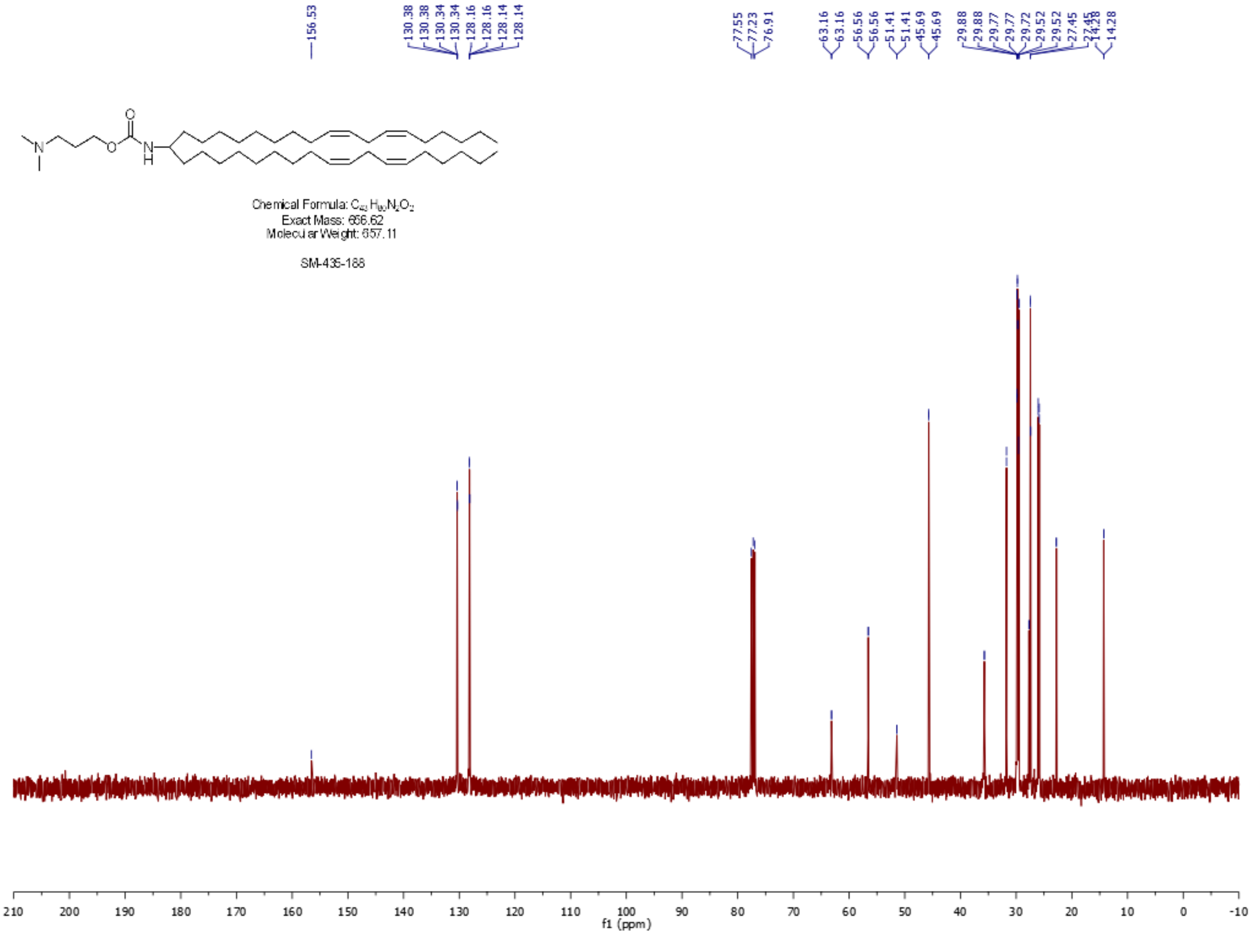

156.53
130.38
130.38
130.34
130.34
128.16
128.16
128.14
128.14
77.55
77.23
76.91
63.16
63.16
56.56
56.56
51.41
51.41
45.69
45.69
29.88
29.88
29.77
29.77
29.72
29.52
29.52
27.45
14.28
14.28
Chemical Formula: $C_{43}H_{80}N_2O_2$
Exact Mass: 656.62
Molecular Weight: 657.11
SM-435-188
210 200 190 180 170 160 150 140 130 120 110 100 90 80 70 60 50 40 30 20 10 0 -10
f1 (ppm)

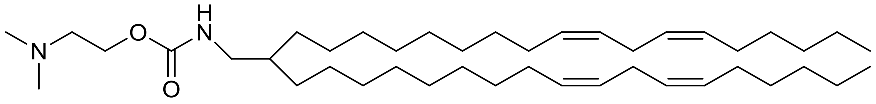

45

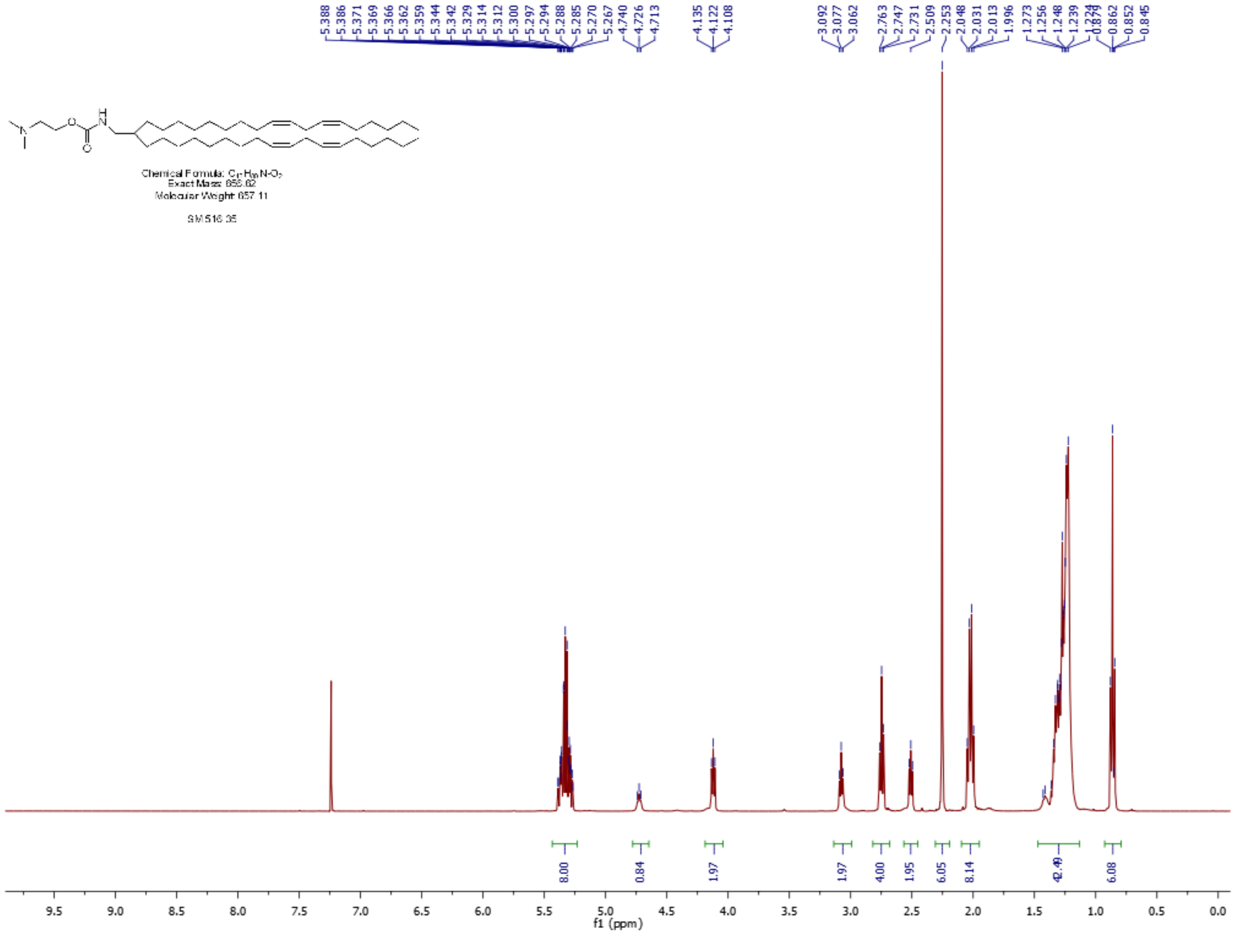

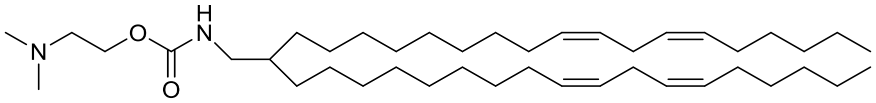
N
O
H
N
O

45

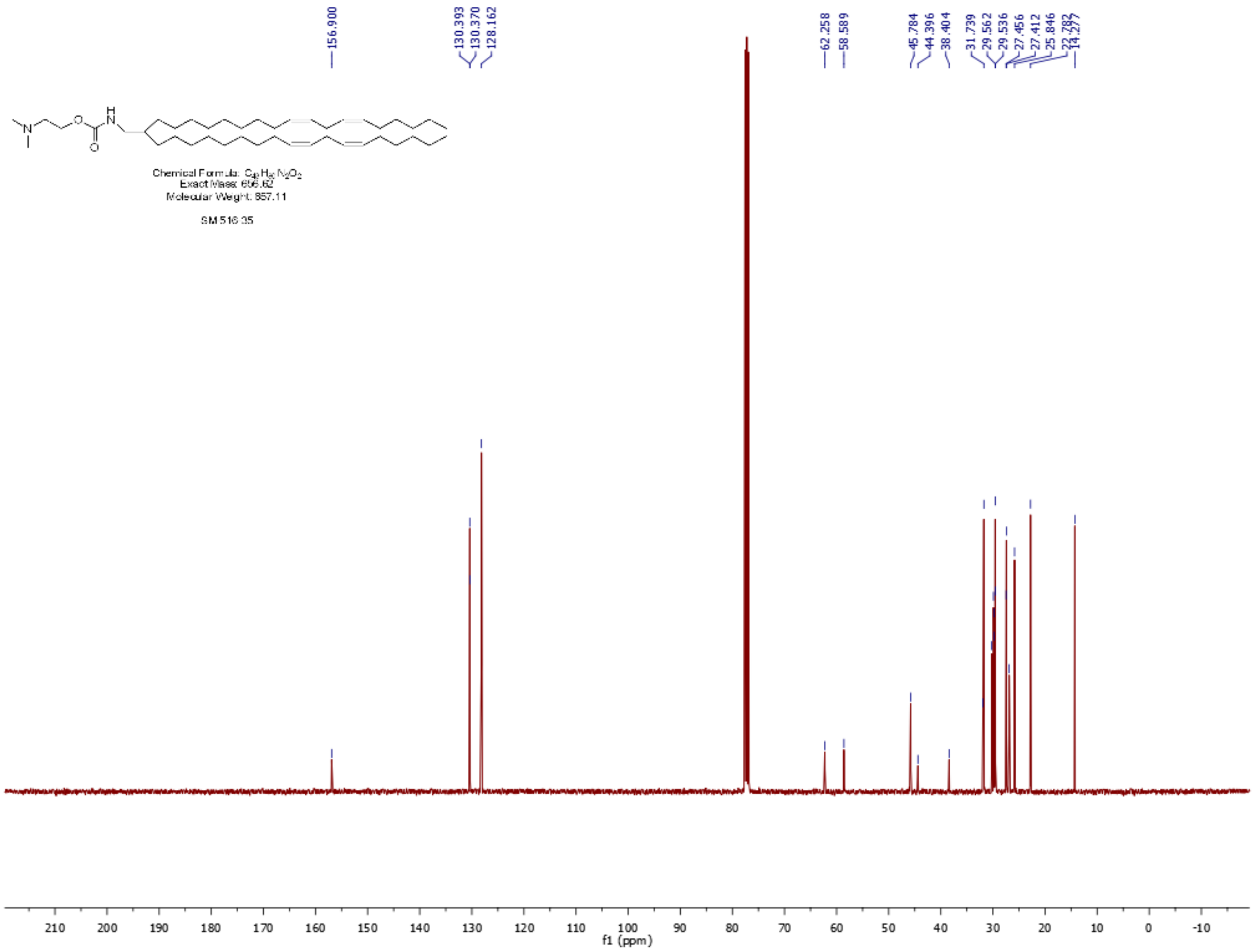
156.900
130.393
130.370
128.162
62.258
58.589
45.784
44.396
38.404
31.739
29.562
29.536
27.456
27.412
25.846
22.782
14.277
Molecular Weight: 857.11
210
200
190
180
170
160
150
140
130
120
110
100
90
80
70
60
50
40
30
20
10
0
-10
f1 (ppm)

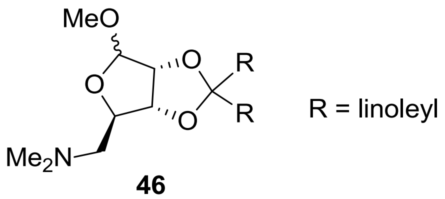

46

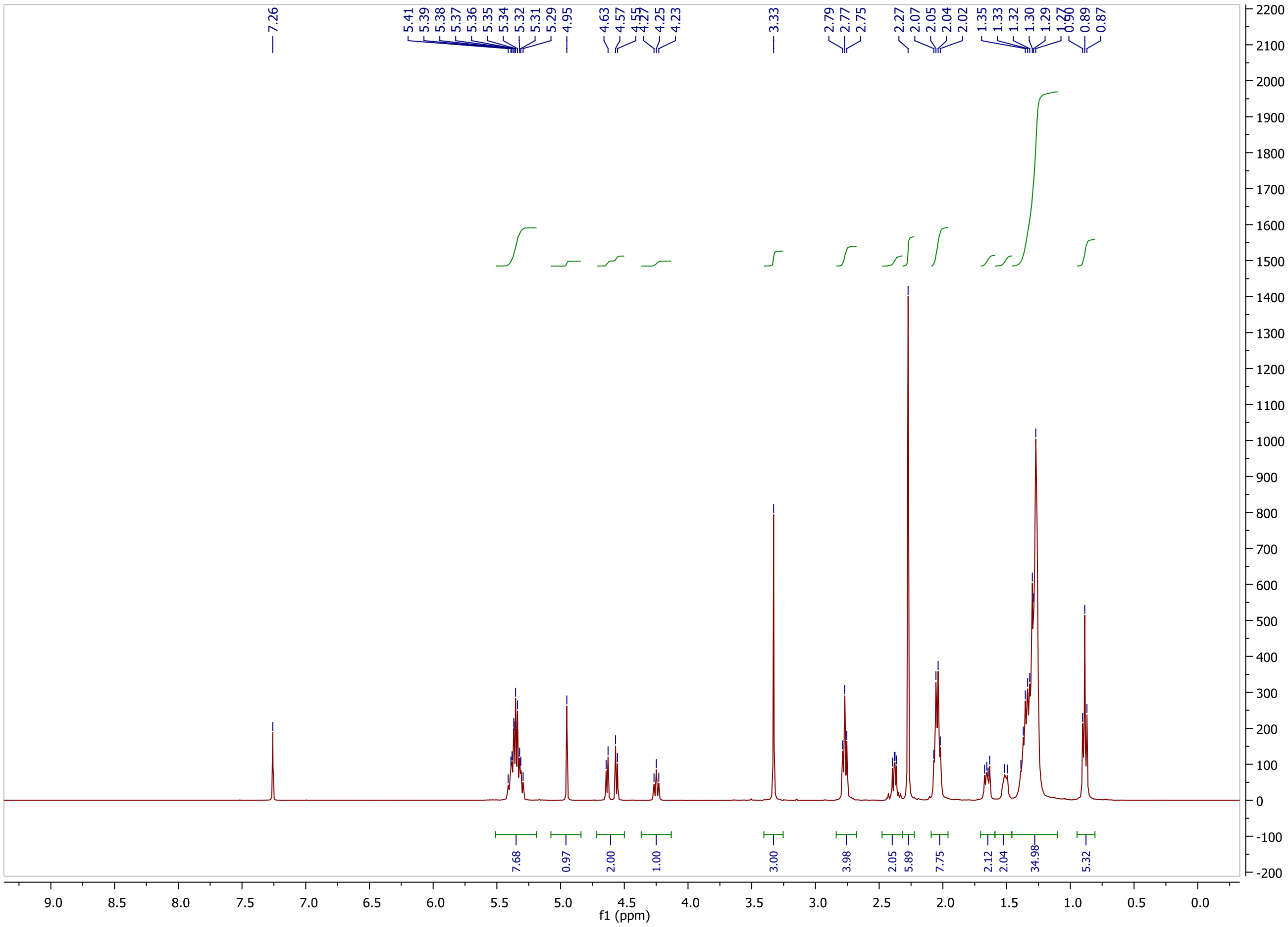

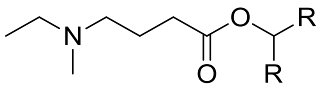

47

R = linoleyl

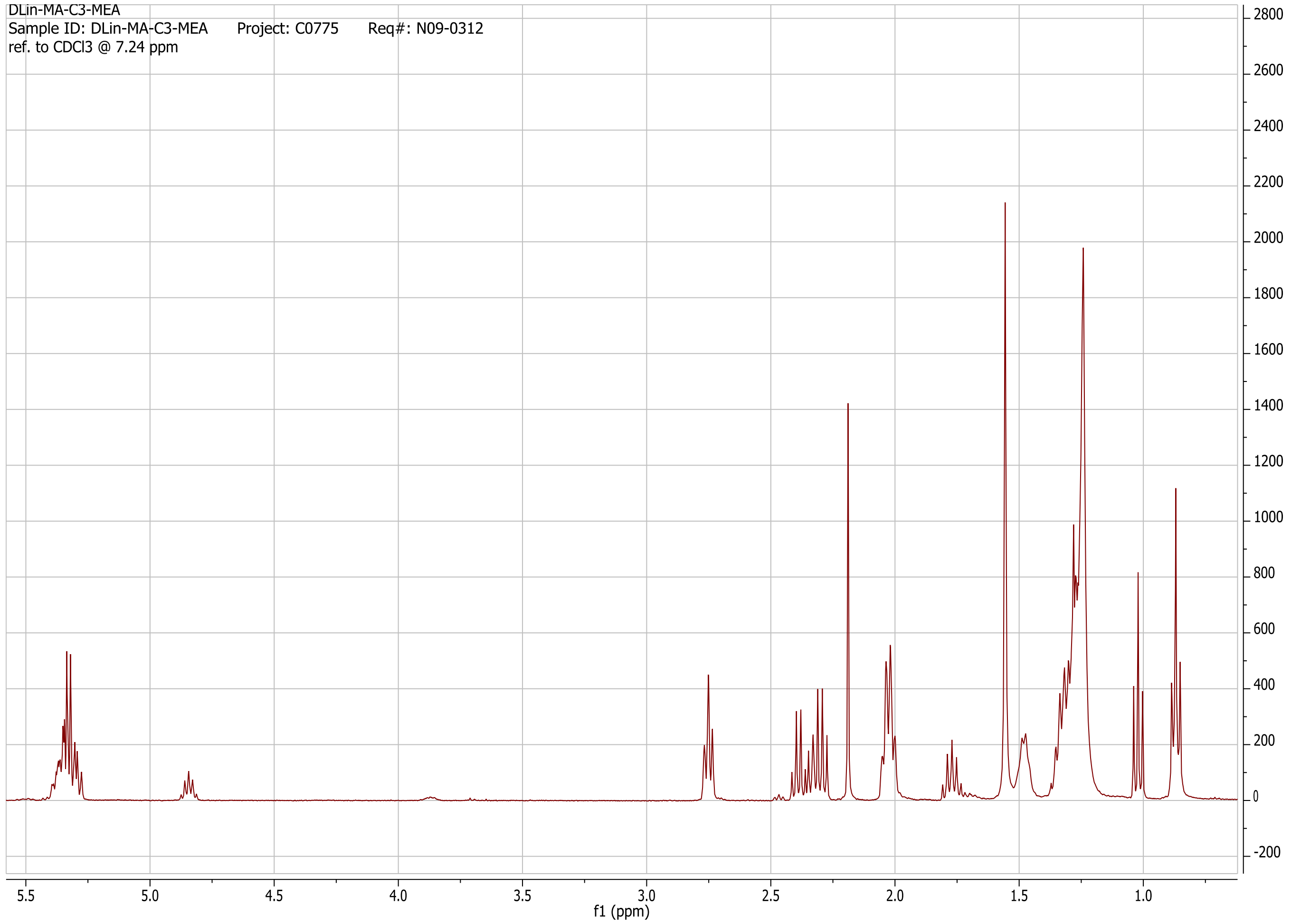

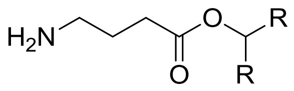

R = linoleyl

48

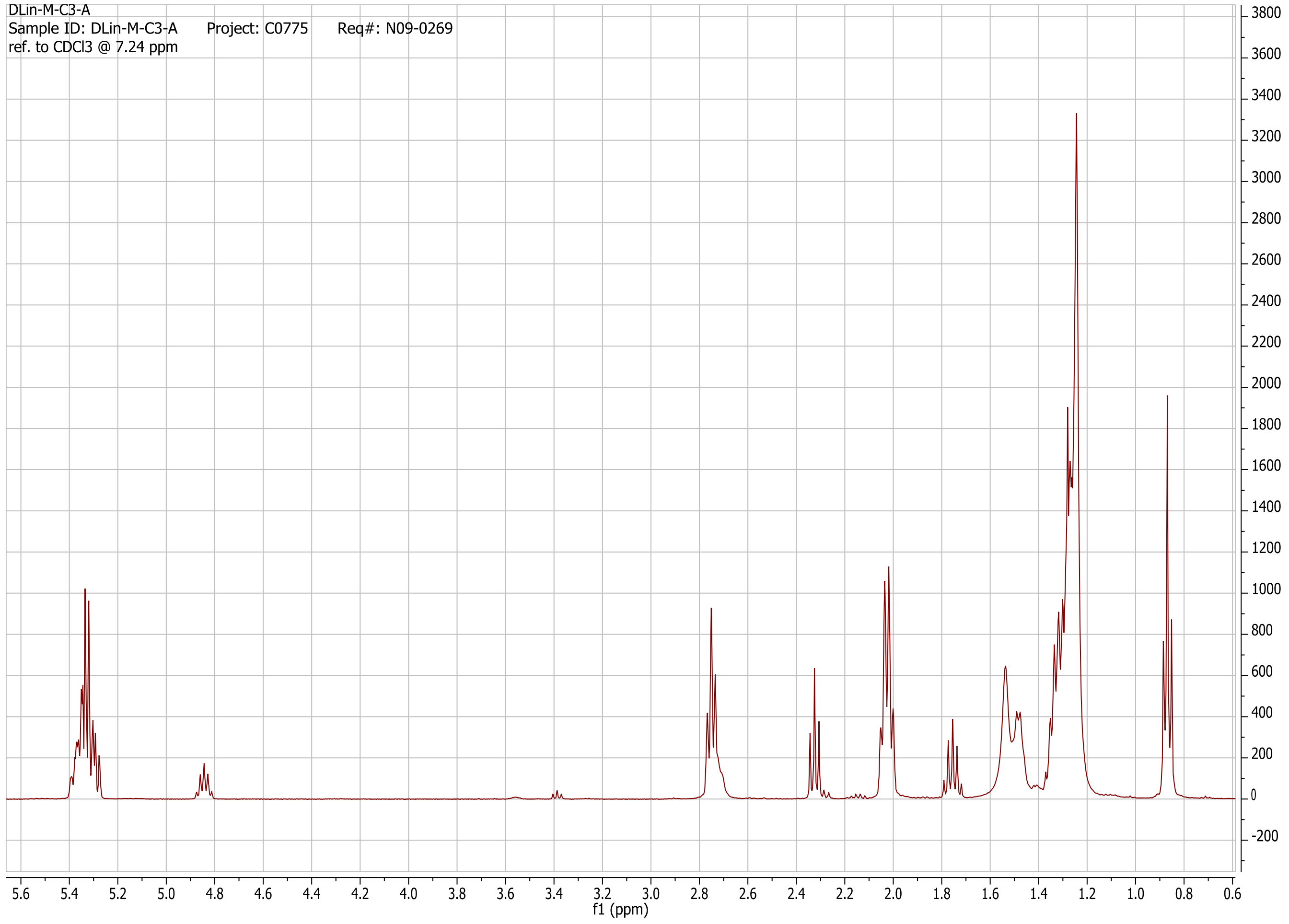

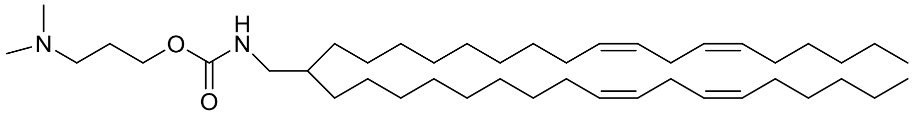

49

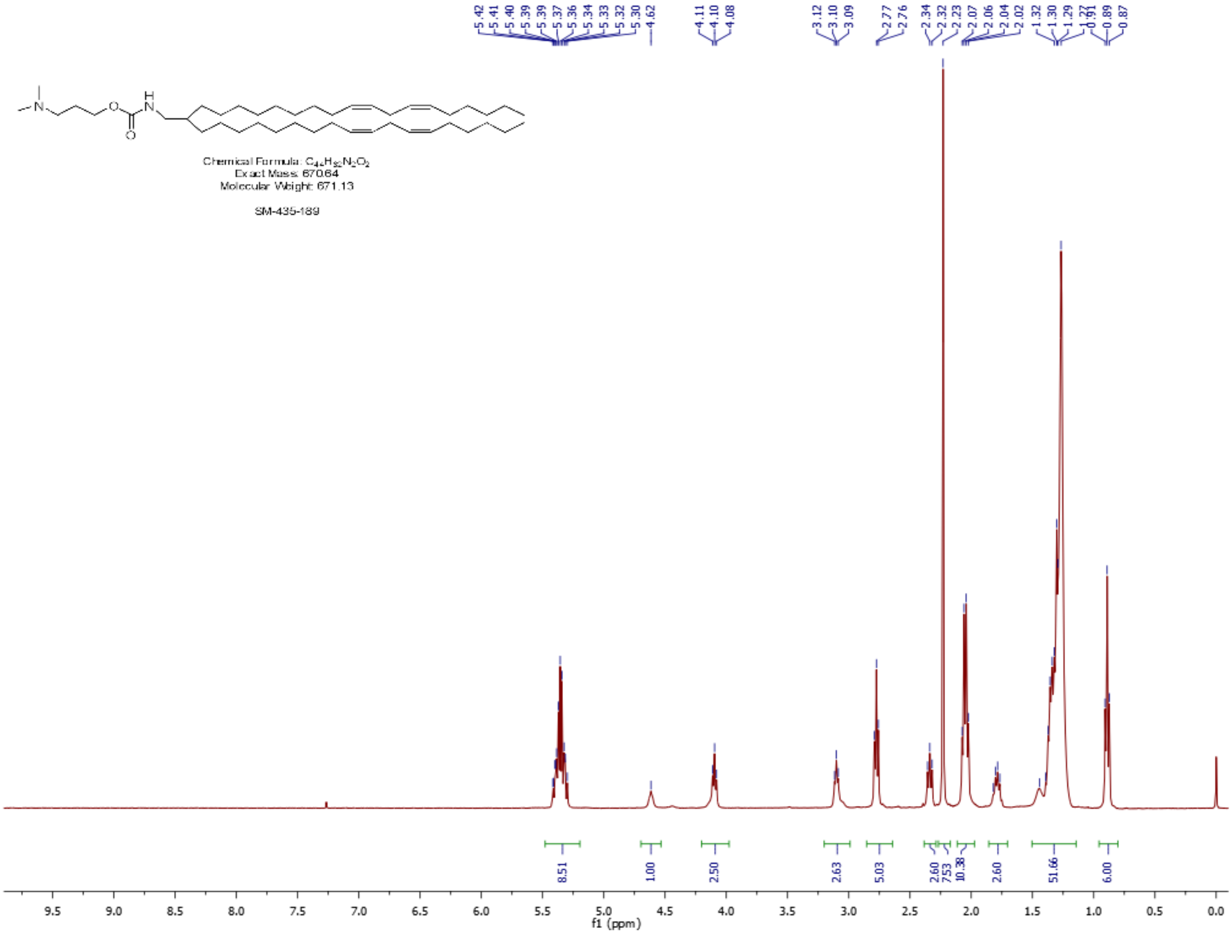
Chemical Formula: C44H82N2O2
Exact Mass: 670.64
Molecular Weight: 671.13
SM-435-189
f1 (ppm)

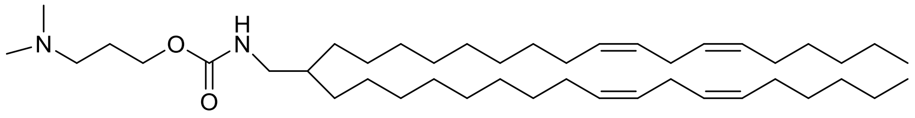

49

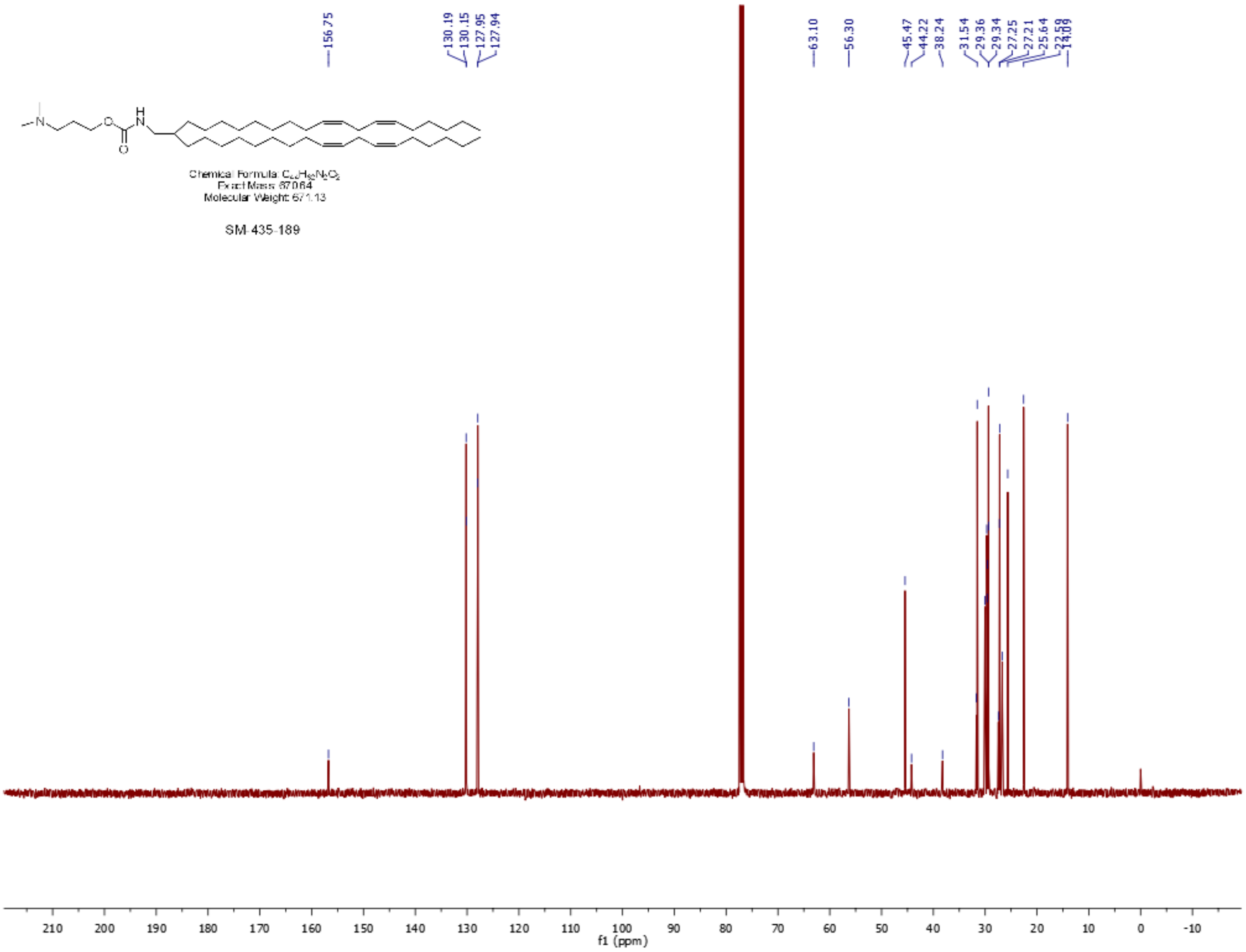
156.75
130.19
130.15
127.95
127.94
63.10
56.30
45.47
44.22
38.24
31.54
29.36
29.34
27.25
27.21
25.64
23.59
14.09
Exact Mass: 670.64
Molecular Weight: 671.13
SM-435-189
210 200 190 180 170 160 150 140 130 120 110 100 90 80 70 60 50 40 30 20 10 0 -10
f1 (ppm)

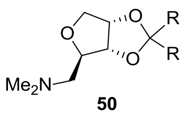

50

R = linoleyl

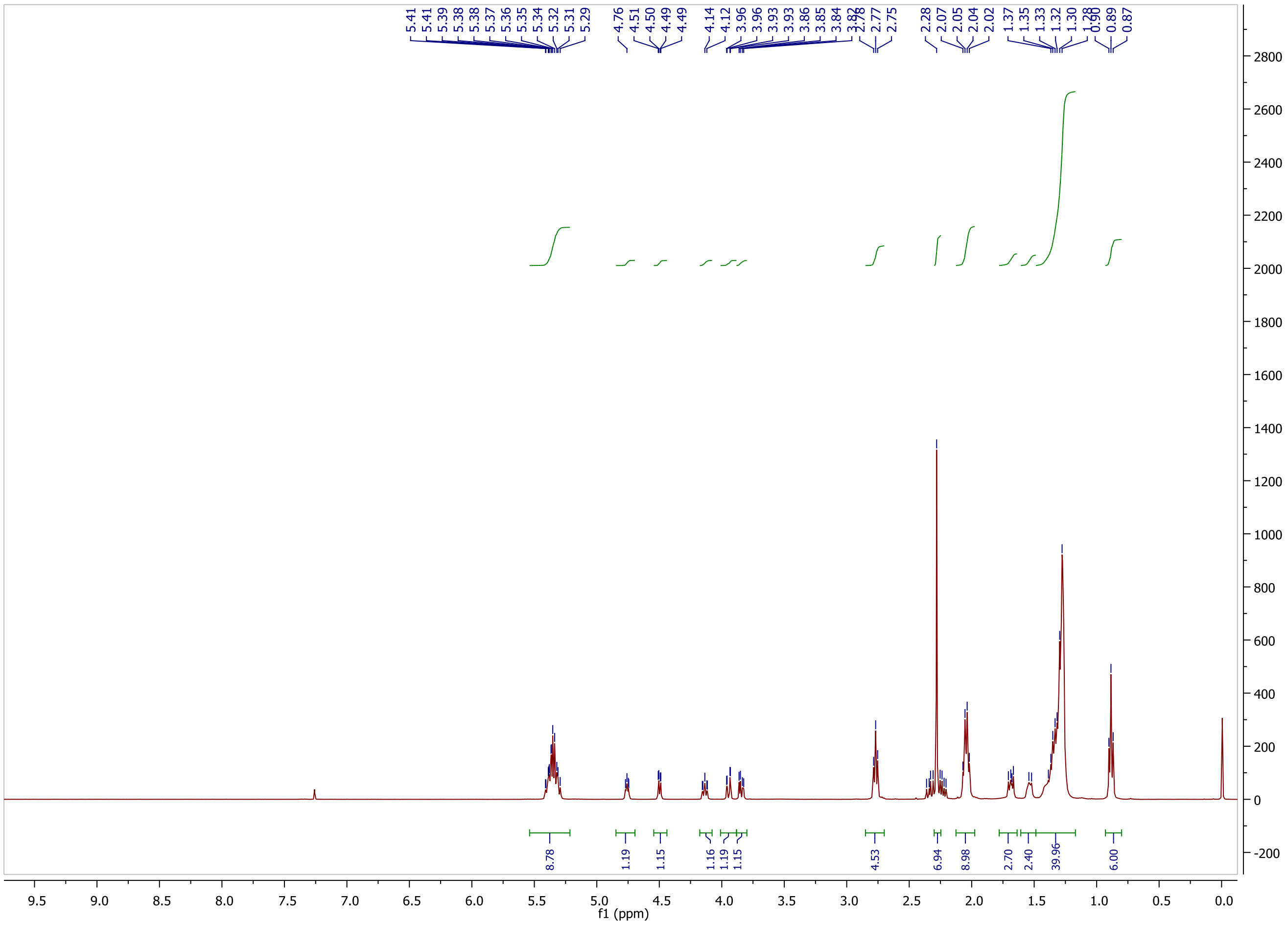

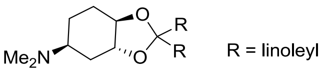

51

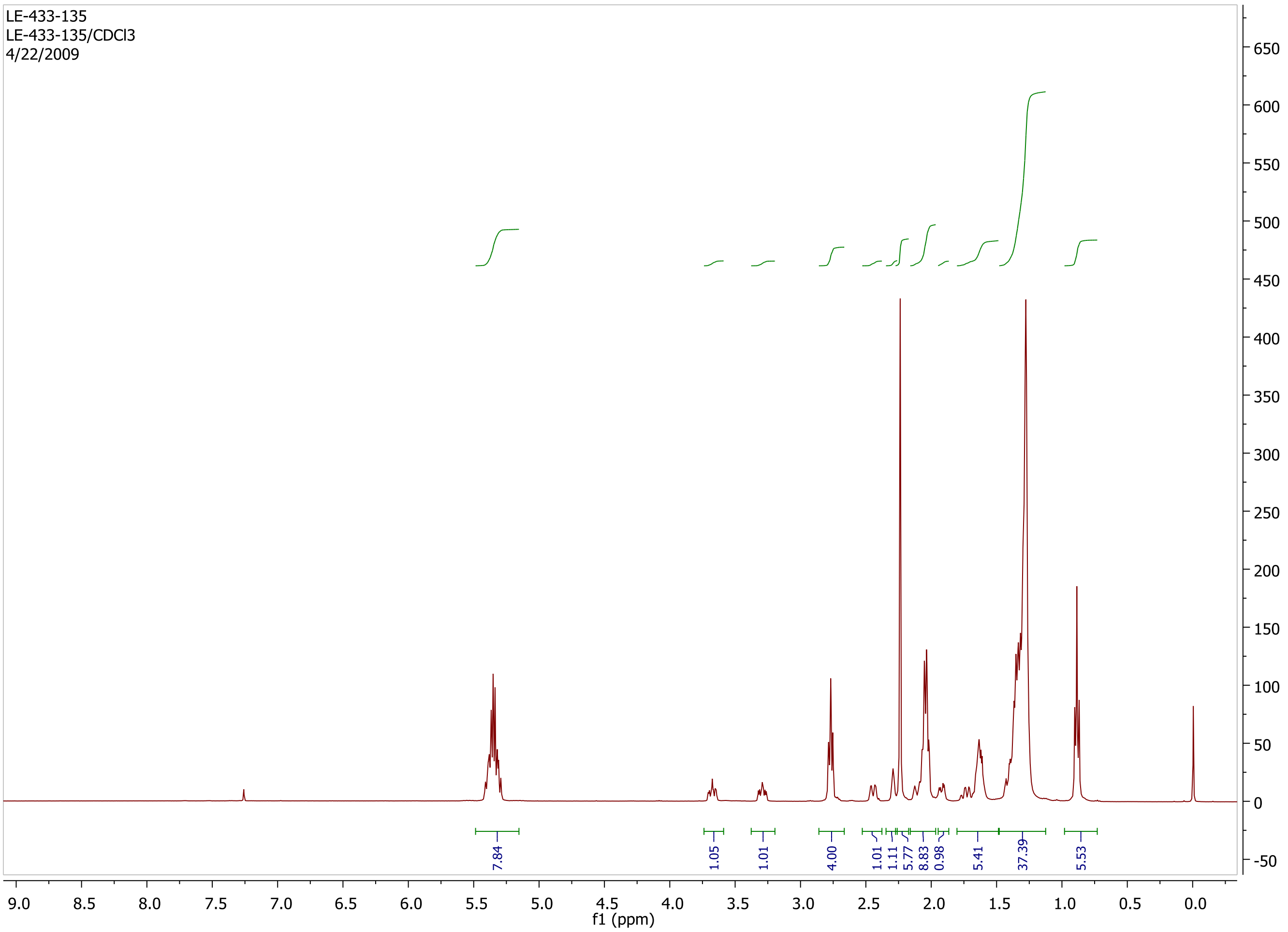

LE-433-135
LE-433-135/CDCl3
4/22/2009
7.84
1.05
1.01
4.00
1.01
1.11
5.77
8.83
0.98
5.41
37.39
5.53
f1 (ppm)

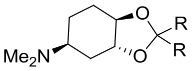

R = linoleyl

51

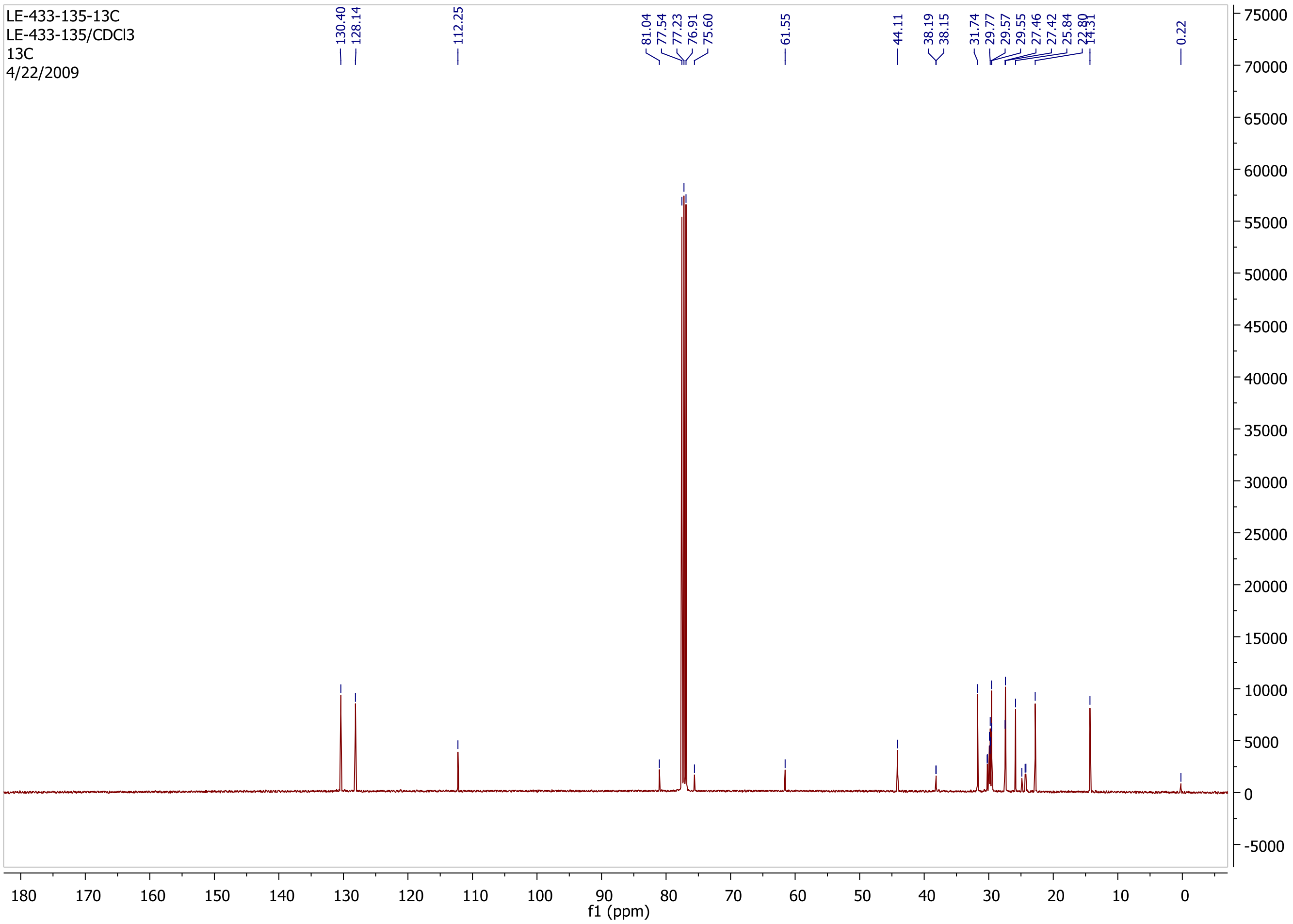

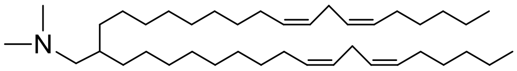

52

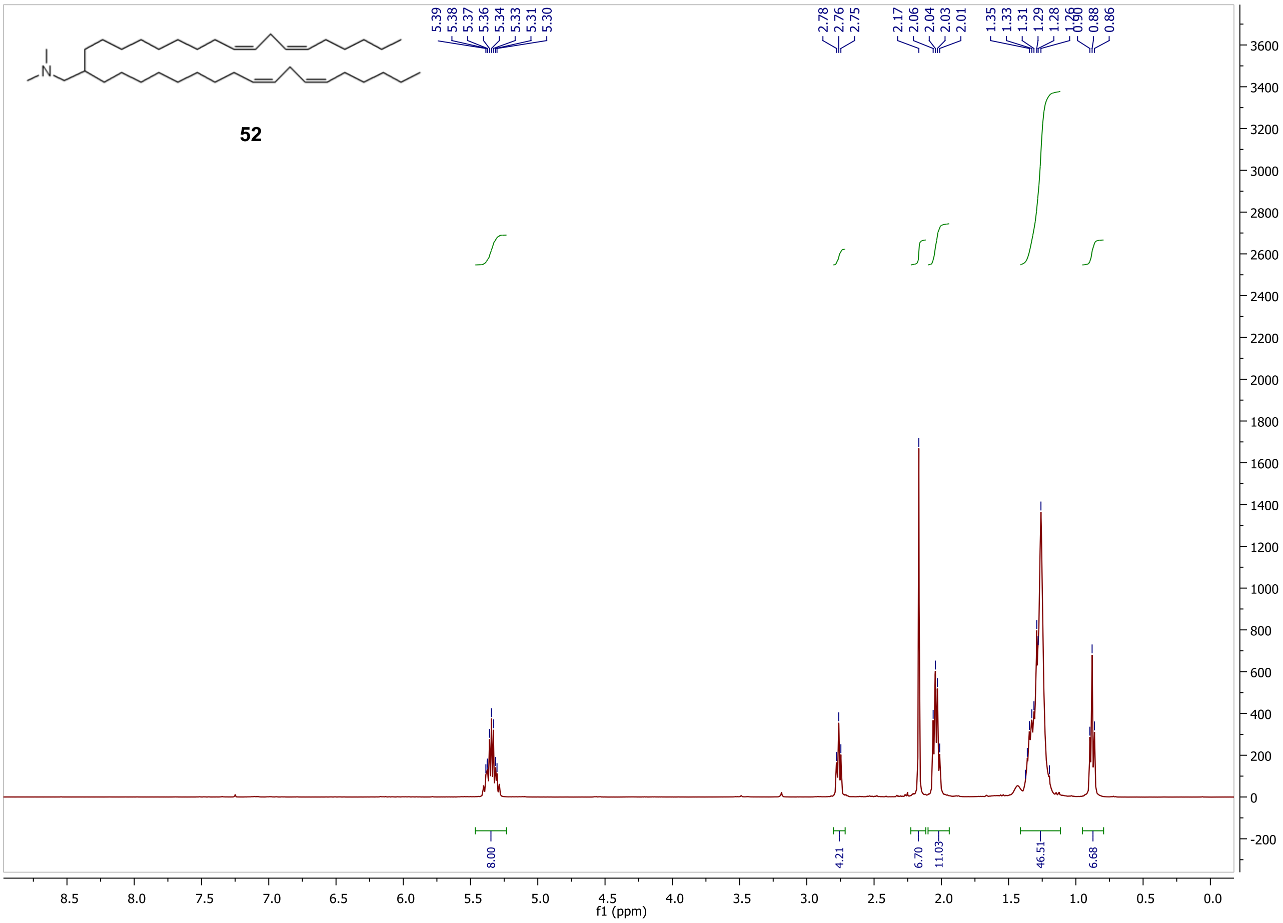

52
5.39
5.38
5.37
5.36
5.34
5.33
5.31
5.30
2.78
2.76
2.75
2.17
2.06
2.04
2.03
2.01
1.35
1.33
1.31
1.29
1.28
1.26
0.90
0.88
0.86
8.00
4.21
6.70
11.03
46.51
6.68
f1 (ppm)

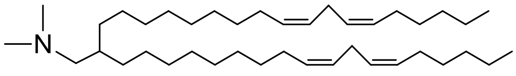

52

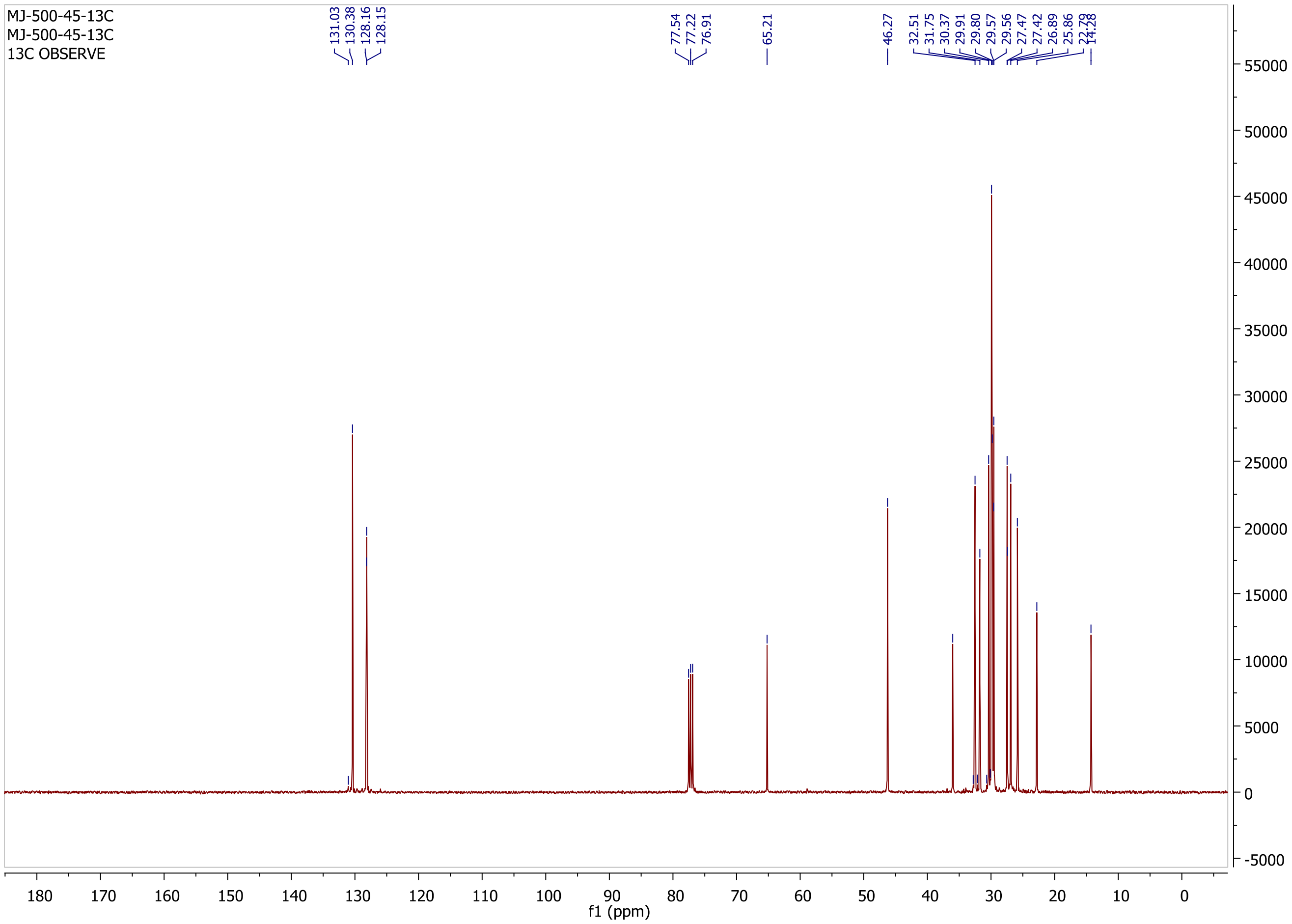

MJ-500-45-13C
MJ-500-45-13C
13C OBSERVE
131.03
130.38
128.16
128.15
77.54
77.22
76.91
65.21
46.27
32.51
31.75
30.37
29.91
29.80
29.57
29.56
27.47
27.42
26.89
25.86
22.79
14.28
f1 (ppm)

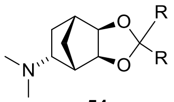

R = linoleyl

54

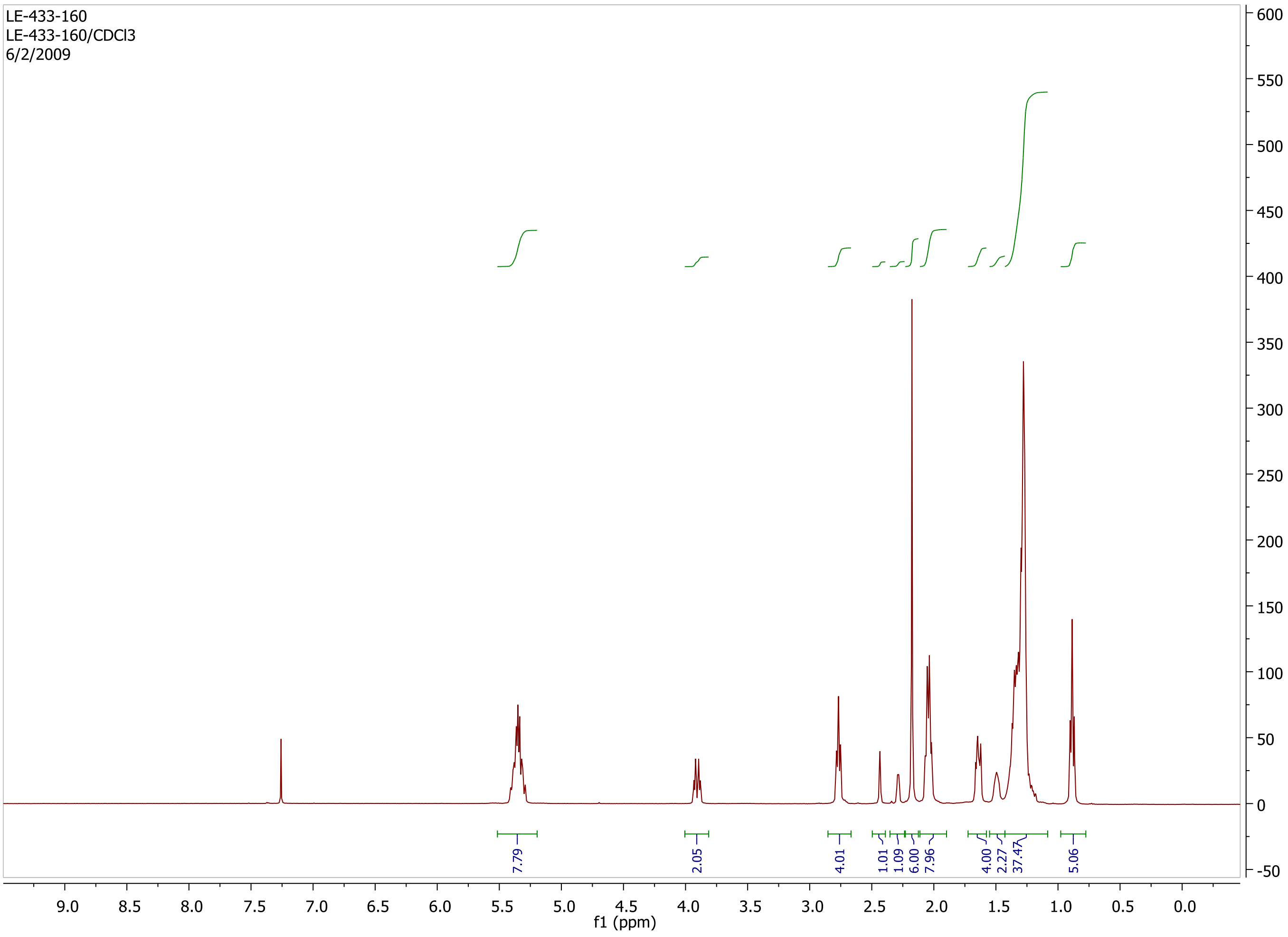

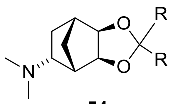

54

R = linoleyl

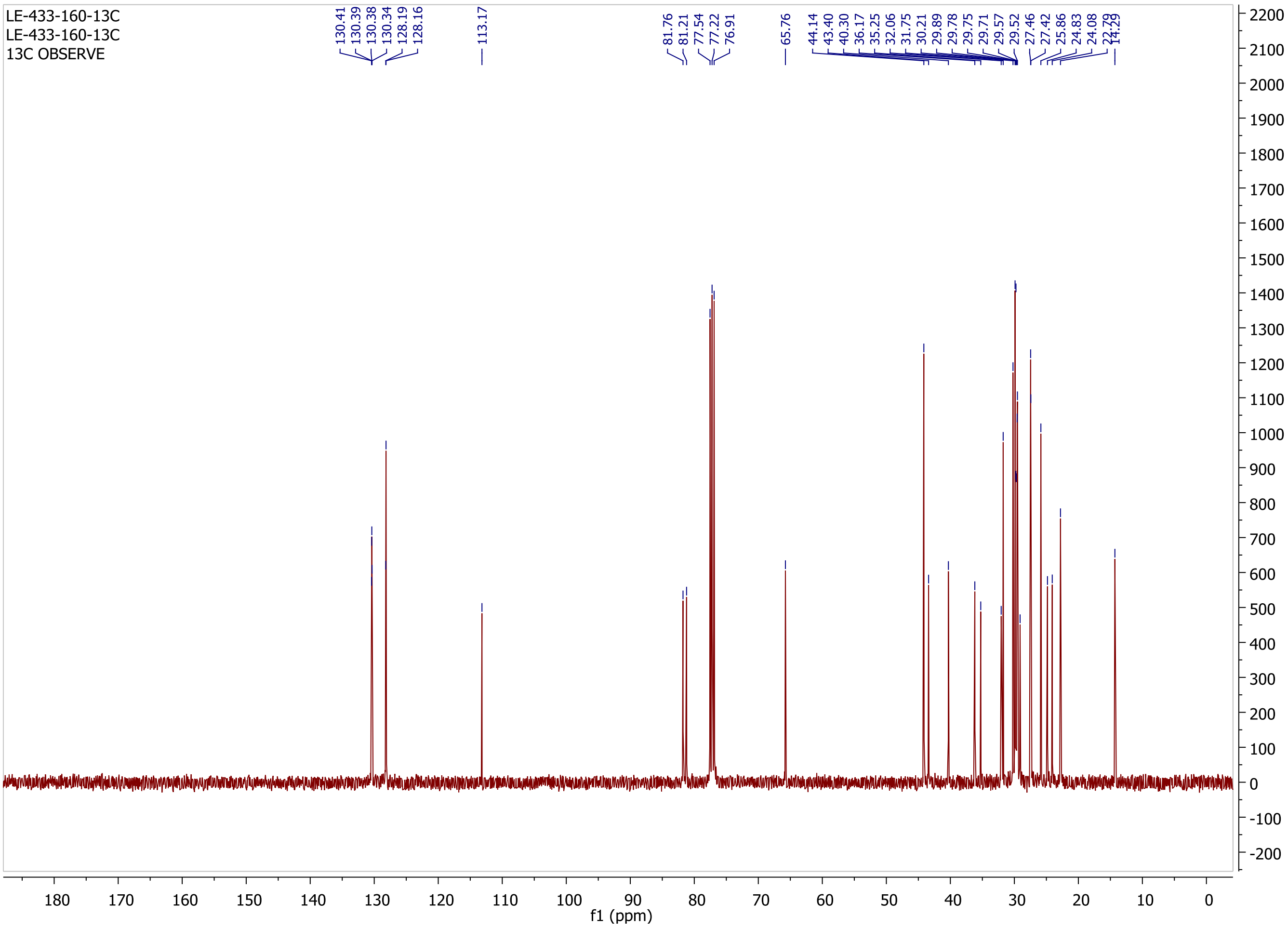

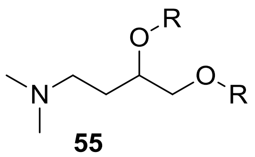

55

R = linoleyl

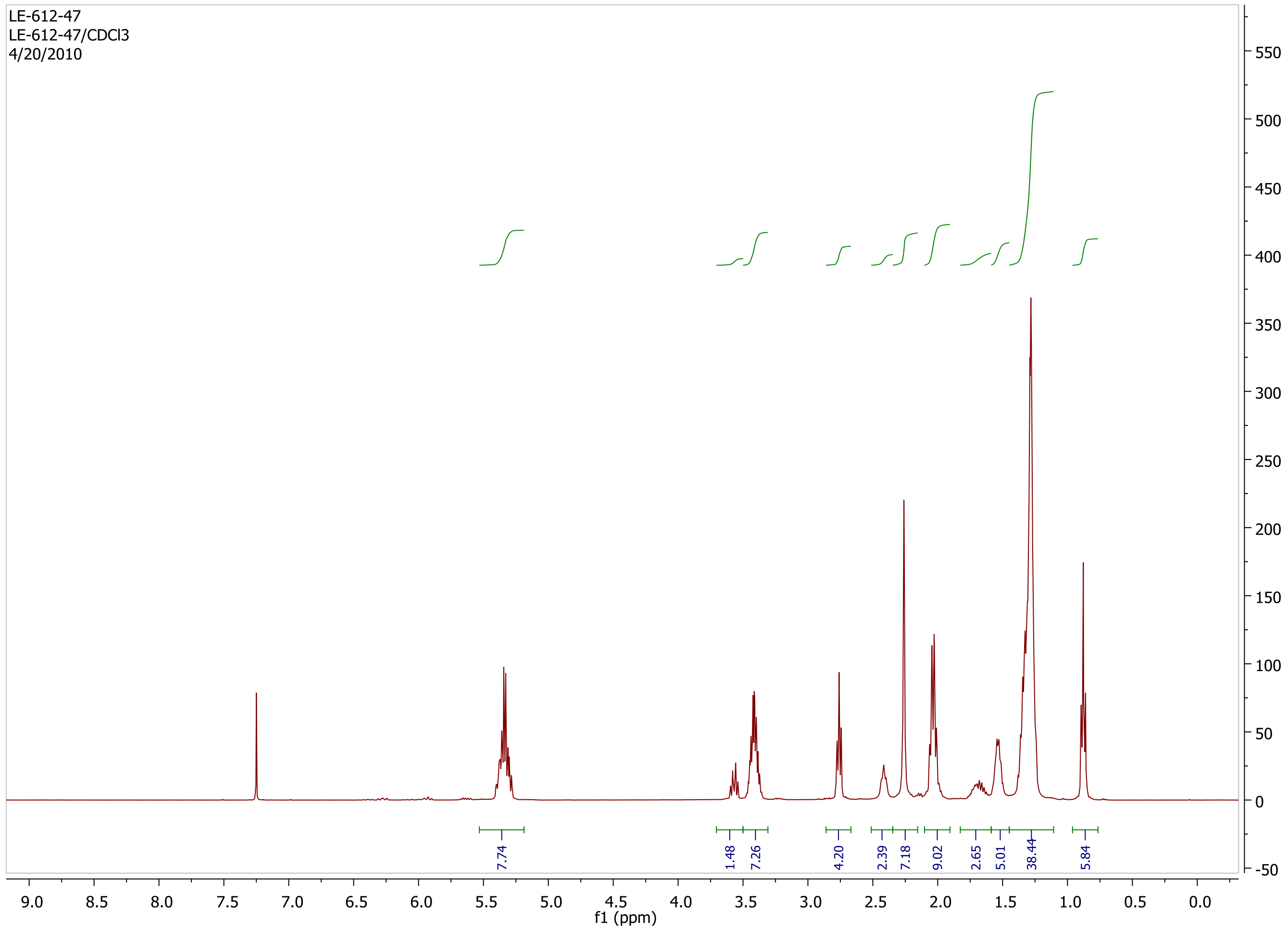

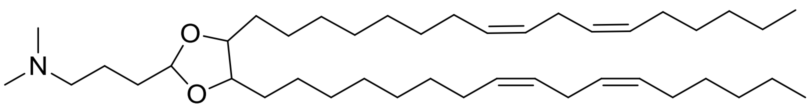

56

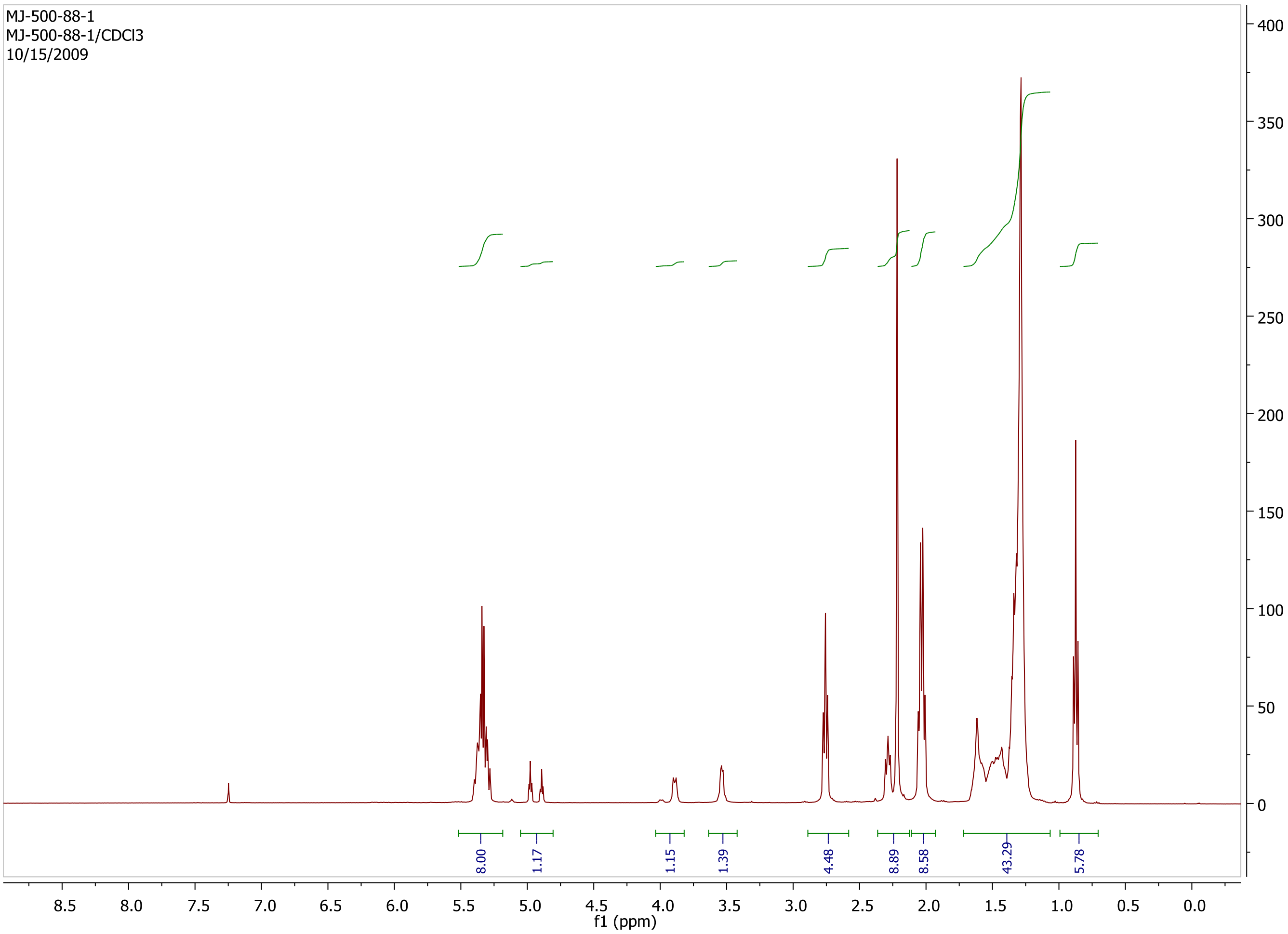
MJ-500-88-1
MJ-500-88-1/CDCl3
10/15/2009
8.00
1.17
1.15
1.39
4.48
8.89
8.58
43.29
5.78
f1 (ppm)
